# Historical Reconstruction Reveals Recovery in Hawaiian Coral Reefs

**DOI:** 10.1371/journal.pone.0025460

**Published:** 2011-10-03

**Authors:** John N. Kittinger, John M. Pandolfi, Jonathan H. Blodgett, Terry L. Hunt, Hong Jiang, Kepā Maly, Loren E. McClenachan, Jennifer K. Schultz, Bruce A. Wilcox

**Affiliations:** 1 Department of Geography, University of Hawai‘i at Mānoa, Honolulu, Hawai‘i, United States of America; 2 Centre for Marine Science, School of Biological Sciences, Australian Research Council Centre of Excellence for Coral Reef Studies, The University of Queensland, Brisbane, Queensland, Australia; 3 Department of Anthropology, University of Hawai‘i at Mānoa, Honolulu, Hawai‘i, United States of America; 4 Kumu Pono Associates, LLC, Lāna‘i City, Hawai‘i, United States of America; 5 Earth to Ocean Research Group, Biological Sciences, Simon Fraser University, Burnaby, British Columbia, Canada; 6 Hawai‘i Institute of Marine Biology, School of Ocean and Earth Science and Technology, University of Hawai‘i, Kāne‘ohe, Hawai‘i, United States of America; 7 Office of Public Health Studies, Global Health Program, University of Hawai‘i, Honolulu, Hawai‘i, United States of America; 8 Program in Ecology, Conservation and Pathogen Biology, National Science Foundation Integrated Graduate Education, Research and Training (IGERT), University of Hawai‘i at Mānoa, Honolulu, Hawai‘i, United States of America; University of California San Diego, United States of America

## Abstract

Coral reef ecosystems are declining worldwide, yet regional differences in the trajectories, timing and extent of degradation highlight the need for in-depth regional case studies to understand the factors that contribute to either ecosystem sustainability or decline. We reconstructed social-ecological interactions in Hawaiian coral reef environments over 700 years using detailed datasets on ecological conditions, proximate anthropogenic stressor regimes and social change. Here we report previously undetected recovery periods in Hawaiian coral reefs, including a historical recovery in the MHI (∼AD 1400–1820) and an ongoing recovery in the NWHI (∼AD 1950–2009+). These recovery periods appear to be attributed to a complex set of changes in underlying social systems, which served to release reefs from direct anthropogenic stressor regimes. Recovery at the ecosystem level is associated with reductions in stressors over long time periods (decades+) and large spatial scales (>10^3^ km^2^). Our results challenge conventional assumptions and reported findings that human impacts to ecosystems are cumulative and lead only to long-term trajectories of environmental decline. In contrast, recovery periods reveal that human societies have interacted sustainably with coral reef environments over long time periods, and that degraded ecosystems may still retain the adaptive capacity and resilience to recover from human impacts.

## Introduction

Coral reefs are among the most diverse and productive ecosystems worldwide, but the abundance of key species and habitat builders have declined globally due to human activities [1], [2], [3], [4]. Though reefs generally face common threats, different patterns of human activities and environmental conditions have led to regional differences in the trajectories, timing and extent of degradation over time [3]. These regional differences highlight the need for in-depth case studies to understand the factors that contribute to either ecosystem resilience and sustainability or decline and collapse [3], [5], [6], [7].

Restoring ecological resilience requires understanding long-term trends (decades to centuries) in resource and ecosystem conditions and characterizing the complex ways that societies have mediated environmental outcomes in the past [5], [6], [7], [8], neither of which is well known for any coral reef ecosystem in the world. It is generally held that societies alter ecosystem conditions directly through proximate human activities, which are in turn determined by the underlying economic, demographic, technological and institutional social systems that mediate social-ecological interactions [9], [10], [11], [12]. For reefs, studies of cumulative change at the global scale have primarily focused on the timing, intensity and effects of different proximate human stressors including overexploitation, land-based pollution, disease, invasive species and threats associated with climate change [2], [3], [13], [14], [15]. Considerably less attention, however, has focused on how underlying changes in social systems indirectly shape social-ecological interactions in coral reefs. This gap obscures the potential linkage and interdependence of the different responsible factors (direct and indirect) associated with sustainable human systems and disturbance levels for coral reefs, which must be unravelled for successful ecosystem restoration and management [3], [16].

Using coral reefs and island societies as a model social-ecological system [17], [18], we reconstructed human-environment relationships to test for sustainable levels of anthropogenic disturbance in human-dominated seascapes. Our reconstruction spans the past 700 years and is based on independent datasets on ecological conditions and social system change, which together provided the basis for reconstructing long-term social-ecological relationships in Hawaiian coral reef systems. Here we report previously undetected periods of ecosystem recovery, including a historical recovery in the Main Hawaiian Islands (MHI) (∼AD 1400–1820) and an ongoing recovery in the Northwestern Hawaiian Islands (NWHI) (∼AD 1950–2009+). Coral reefs recovered from human impacts when the intensity of anthropogenic stressors and the number of ecological guilds affected were reduced over long time periods (decades+) and large spatial scales (> entire island systems or regions [>10^3^ km^2^]), which limited direct human stressors of reef systems to sustainable levels. Reductions in proximate human stressors were mediated indirectly by a complex set of historical events and consequent changes in social systems, which altered social-ecological relationships in reef environments.

## Materials and Methods

**Figure 1:**
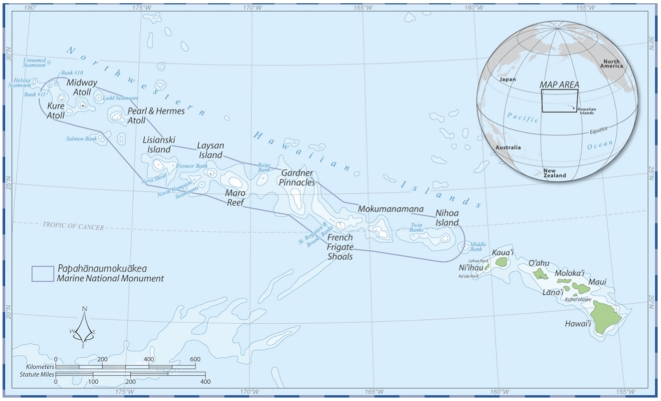
Map of the Hawaiian Archipelago, comprised of the inhabited high islands of the main Hawaiian Islands (from Kaua‘i/Ni‘ihau to Hawai‘i Island) and the uninhabited reefs, banks, and atolls of the Northwestern Hawaiian Islands (NWHI). The boundary for the Papahānaumokuākea Marine National Monument in the NWHI is indicated in black. Map courtesy of the NOAA Papahānaumokuākea Marine National Monument Office.

We reconstructed ecological changes at the guild level, relying on a diversity of data types to assess changes in the degradation and depletion of coral reef biota through an intensive review and assessment of archaeological deposits, ethnohistoric and anecdotal descriptions and modern ecological and fishery data. This type of historical analysis is limited in precision but assessing change through multiple data types provides a valid method for reconstructing long-term ecological trends and characterizing the social factors that have shaped ecosystem conditions [2], [6], [19], [20], [21]. The time period considered includes over 700 years of human occupation of the Hawaiian Archipelago, from AD 1250–2009. Recent research in high-precision radiocarbon dating places the date of colonization of Hawai‘i at approximately AD 1250 [22], [23]. Thus AD 1250 provides a pristine (pre-human) baseline for coral reef ecosystems. For consistency, the NWHI baseline also starts at AD 1250, but unpublished results from Th-230 dating on coral recovered from NWHI archaeological sites suggests human settlement did not pre-date the mid-15^th^ century [24]. Coral reef ecosystems were grouped into two major regions in the Hawaiian Archipelago, including the populated MHI and the primarily uninhabited atolls, reefs and banks of the NWHI (Figure 1). These regions were selected based on social factors, including level of human habitation, and also for comparability with regional coral reef ecosystems assessed in a global review [3].

Coral reef ecosystem conditions were reconstructed using a modified version of a method developed for assessing long-term ecological change in marine environments [2], [3], [4], [25]. This method consists of: (a) Determining the ecological status (“EcoState”) of organisms comprising seven ecological functional groups, or guilds, for each reef at distinct points in time using multiple types of data; (b) Performing an indirect gradient analysis on the guild status database; and, (c) Estimating the location of each reef at each time along a gradient of degradation from pristine (pre-human) to extinct to determine historical trajectories of changes in ecological state. Coral reef biota were grouped into seven ecological guilds (functional groups), defined by their mode of nutrition (herbivore, carnivore), life habit (mobile/free-living or sessile/architectural), and size (for free-living species large >1 m, small <1 m) (Table S1). Species were considered to be part of coral reef ecosystems if they were reef-associated for a significant part of their life history and were functionally connected to coral reef foodwebs. This determination can be construed as broad in that it includes deep-reef demersal piscivores (bottomfish) and estuarine/nearshore species.

Data were extracted directly or derived from archaeological studies and midden analyses (N = 46; 17 total sites), ethnographic and archival anecdotal accounts (N = 990), contemporary ecological studies (N = 228), and annually published fisheries datasets (N = 55) (Table S3). Archaeological sources included datasets and reports on 17 sites distributed throughout the MHI and two sites in the NWHI (Table S4). Citations for all sources reviewed for this research are listed in the Supporting Information S1. The high number of observations for each guild and the diverse types of data utilized in the study (Table S3) allowed for a reconstruction of coral reef conditions at the level of decades to centuries over the past 700 years (Figure S1).

The status of ecological guilds was determined through analysis and quantitative scoring of data sources (Table S3). Quantitative “EcoState” scores were based on criteria established to assess the scale of human impact on an ordinal scale of ecological state (Table S2), and scores were conservatively assigned based on a review of multiple types of data and evidence available for each set of taxa comprising a given guild. Net determinations of guild status through time were estimated by reviewing all the information and data for a particular guild. Aggregate trends are discussed herein and more detailed summaries of data used to reconstruct changes in ecological status by guild are included in the Supporting Information S2.

Transitions between ordinal EcoState scores in individual guilds were scored as to whether the changes were gradual or sudden based on the evidence available from different data types. For example, the transition from subsistence use to mass harvesting of a marine resource for a newly developed export market constituted a sudden transition (e.g. from EcoState 2→3), whereas if evidence revealed a gradual decrease in the abundance of an organism then the transition was scored as a gradual shift (e.g. from EcoState 1→1.25→1.5→1.75→2). To determine the overall trajectory of the entire coral reef ecosystem, EcoState scores for each guild were averaged. Averages were not weighted, which assumes that each guild is equally important in maintaining an intact coral reef. Ordinal EcoState scores (Table S2) were converted to percentage depletion-degradation, where 1 (pristine)  = 0% depletion-degradation and 6 (globally extinct)  = 100% depletion-degradation.

Proximate human stressors to coral reef ecosystems were quantified for each guild through time, and were classified into five major types of disturbance: 1. Overexploitation; 2. Invasive Species; 3. Land-Based Pollution, 4. Disease; and, 5. Climate Change. Proximate stressors were quantified on an ordinal scale that included small (1), medium (2) or large (4), and the magnitude of proximate stressors was determined based on a determination of severity of the effect through reviewing all the available evidence. For example, overexploitation by subsistence or artisanal fishing was determined to be small (1), whereas evidence of large-scale commercial or industrial harvesting was classified as large (4). To quantify proximate stressors at the ecosystem level, individual stressors were summed for each guild. Cumulative human impacts were then represented graphically on an intensity scale as a proportion of the total stressor load by region (MHI & NWHI). Underlying social factors were determined by reconstructing detailed timelines of social change in demography, economic systems, technologies and human institutions related to coral reef environments. Changes in these social systems were described in terms of impacts to each ecological guild, and linkages between social change, proximate human stressors, and ecological conditions were assessed based on the available evidence from multiple sources.

## Results

**Figure 2:**
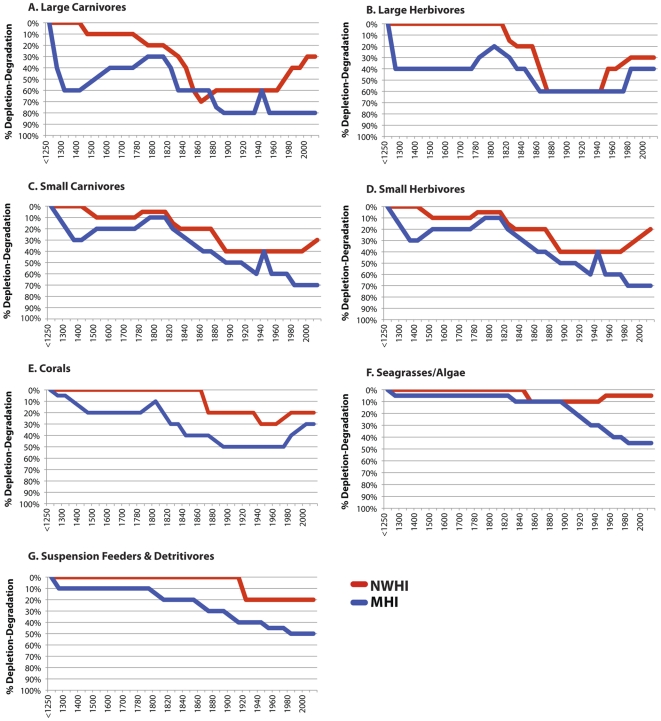
Changes in depletion and degradation of seven trophic guilds of coral reef biota in the Hawaiian Archipelago. Guild trendlines span the period AD 1250–2009 for both the Main Hawaiian Islands (MHI, blue) and the Northwestern Hawaiian Islands (NWHI, red) and were reconstructed using archaeological midden data (N = 42), ethnographic and archival anecdotal accounts (N = 990), contemporary ecological studies (N = 228), and annually published fisheries data (N = 55) (Figure S1). Guilds include free-living organisms (A–D: large carnivores; large herbivores; small carnivores; small herbivores) and architectural/sedentary species (E–G: corals; seagrasses/algae; suspension feeders & detritivores).

**Figure 3:**
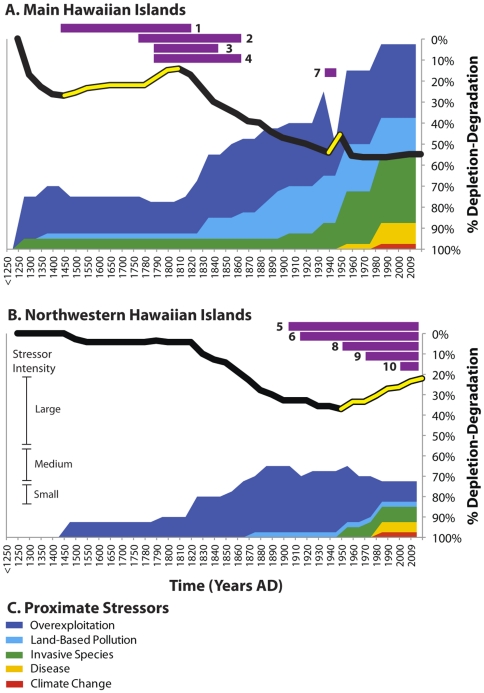
Trajectories of change in coral reef ecosystems in the Main Hawaiian Islands (MHI) (A) and the Northwestern Hawaiian Islands (NWHI) (B). Ecosystem trendlines (in black) represent averages of changes in relative abundance in 7 trophic guilds (Figure 2). Periods of reef recovery in the MHI (AD 1450–1800) and the NWHI (AD 1950–2009) are indicated where the trendline is yellow. Proximate human stressors (C), indicated as colored regions, are quantified on a scale of intensity (see Methods). Recovery factors are represented by purple bars and reference column D on Table 1. Proximate stressors exhibit an inverse relationship with ecosystem condition on an axis of depletion-degradation. Time is represented on the horizontal axes (AD 1250–2009); 50-year increments are used in the prehistoric period (AD 1250–1778) and 10-year increments are used for the historic and modern period (AD 1778–2009).

Our reconstruction reveals that trajectories of change in ecological condition differed among coral reef guilds and between regions of the Hawaiian archipelago (Figure 2). Generally, free-living guilds experienced earlier and more drastic declines than architectural/sedentary guilds, but free-living biota were also primarily responsible for periods of ecosystem recovery (Figures 2, S2, S3, S4). Proximate factors impacting reef species also varied by guild and among regions (Figures S2, S3). Overexploitation, land-based pollution and invasive species constituted the earliest and longest-lasting proximate human stressors affecting free-living guilds, and climate change impacted only architectural guilds. Though guilds exhibited different trajectories based on proximate stressor regimes and underlying social change, our analysis uncovered recovery periods operating at the ecosystem level (Figure 3, Table 1). Below, we discuss in more detail the diverse datasets used to reconstruct ecosystem conditions, and our interpretation of the social factors associated with ecosystem change in the MHI and NWHI.

**Table 1:** Timeline of major historical events and the underlying social factors associated with coral reef decline and recovery over 700 years in Hawaiian coral reefs.

Date (Years AD)	A. Major Historical Events	B. Decline Factor	C. Event	D. Recovery Factor
<1250	Pristine coral reef ecosystem			
1250–1450	Voyaging Polynesians settle in coastal areas adjacent coral reef fisheries	Human harvesting and invasive species reduce vulnerable marine megafauna		
1450–1700	Rise of large chiefdoms, large-scale fishpond aquaculture, agricultural complexes and animal husbandry	Human population reaches its zenith (∼1450) then stabilizes	1	Imposition of social consumption controls on some marine fauna; Reef conservation strategies implemented (∼1400–1819)
1778	Discovery by western explorers (1778)		2	Indigenous depopulation due to disease epidemics (1778–1860+)
1819–1840s	Abolishment of indigenous religious system; Arrival of Christian missionaries and whalers	First commercial fisheries develop; Indigenous cultural practices discouraged; Traditional restrictions on some reef species removed; Indigenous depopulation continues	3	Chiefly re-direction of labor away from traditional fishing practices (1790–1840+)
			4	Attrition of able-bodied men to foreign commercial enterprises (1790–1860+)
1848–1852	Transition from feudal, common property land ownership system to fee simple ownership in the Great Māhele	Customary marine tenure systems seriously eroded with many indigenous claims to fisheries resources lost, unrecorded or stolen		
1860s+	Sugar and ranching economy develops	Sedimentation in reef environments, loss of fishponds; Dynamite fishing introduced; Shark and bêche-de-mer fisheries develop		
1893–1930s	US overthrow of Hawaiian monarchy, US annexation; Tourism economy initiates	Widespread overfishing documented in MHI; Harvesting of juveniles for commercial markets; Japanese fishermen further develop pelagic and deep-reef fisheries; Introduction of exotic species for marine cultivation; Major construction at some atolls in NWHI; Private fisheries serially condemned	5	President Roosevelt declares the NWHI a biological refuge to protect resources from foreign commercial harvest (1903–2009)
			6	Exclusion of foreign commercial operations from NWHI (1915–2009); Limited marine resource protections enacted in MHI
1941–1946	Hawaiian Islands placed under Marshall Law during WWII		7	Marshall law restricts access to reef areas in MHI
1950s–1970s	Hawai‘i becomes 50th US state; Rapid growth in human population, coastal development and tourism industry	Statehood abolishes private fishery rights	8	Constriction in NWHI fisheries (1915–2009); Human depopulation in NWHI occurs after WWII (1945–2009); First MHI MPAs established, but are small and isolated
1970s–2009	Major coastal development occurs; Native Hawaiian cultural renaissance	NWHI bottom fishery expands; Lobster fishery boom and bust in NWHI; MHI reefs are depleted due to overfishing and coastal development; Invasive species proliferate	9	Terrestrial ecological restoration in NWHI (1970–2009); No-take MHI MPAs are small and few; Native Hawaiian principles and traditions of stewardship reinvigorated
			10	Major environmental protections are put in place for reefs in the NWHI (2000–2009)

Major historical events (A); underlying social factors leading to decline (B) and recovery (D) for Hawaiian coral reef ecosystems; Recovery events (C) for the MHI (1–4, 7) and the NWHI (5, 6, 8–10) are represented as purple bars on Figure.

### The Main Hawaiian Islands

Voyaging Polynesians arrived in the Hawaiian archipelago sometime around AD 1250 [22], [23], and archaeological midden remains and later recorded ethnohistoric accounts reveal that coral reef species were commonly exploited by Polynesian colonizers [26], [27], [28]. Early prehistoric Hawaiian societies developed around alluvial valleys in the MHI that provided freshwater, potential for irrigated agriculture, and reef environments that were a focus for fishing and foraging [28], [29].

**Figure 4:**
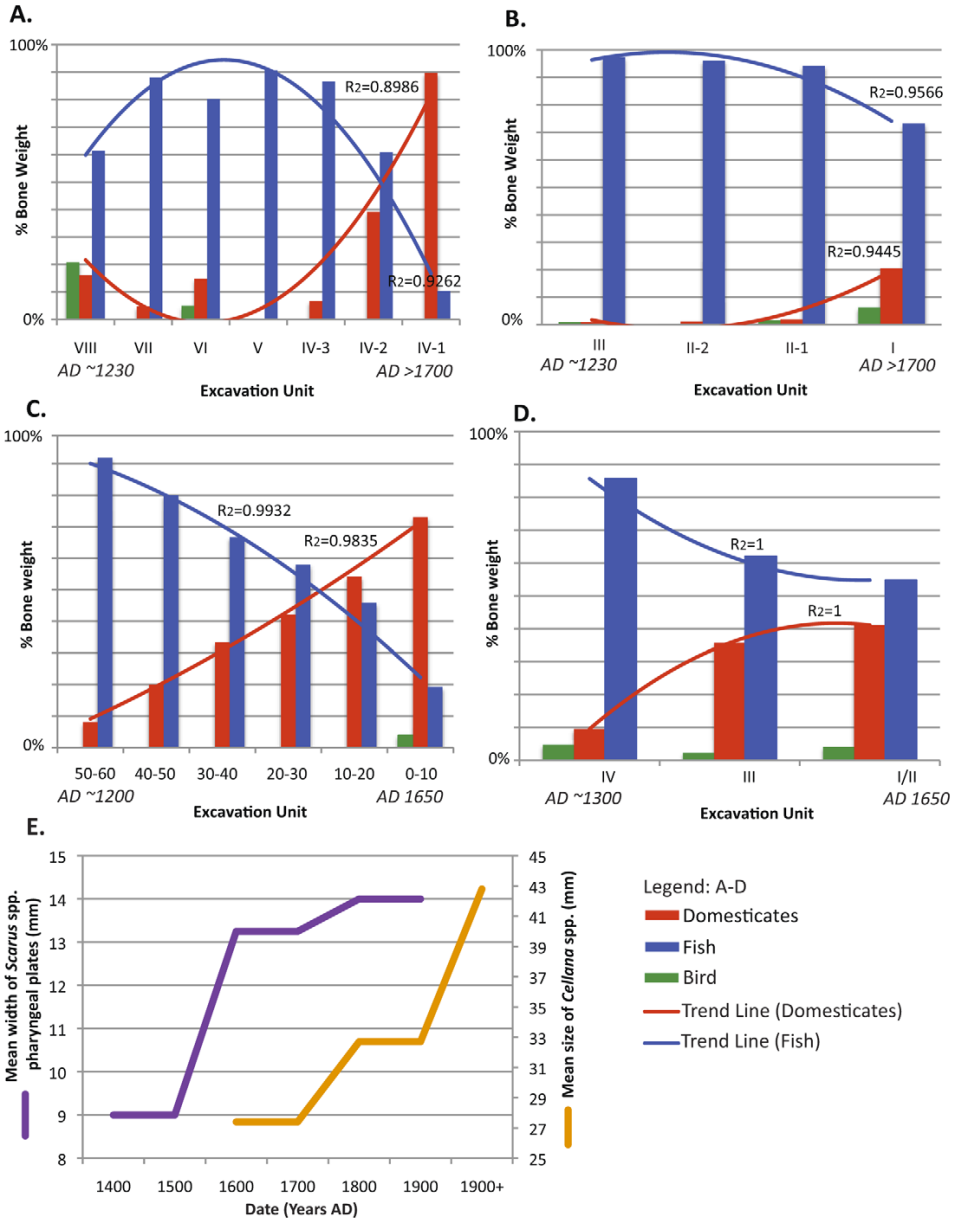
Trends in faunal bone remains in four coastal middens (A–D) and size changes for *Scarus* spp. (parrotfish) and *Cellana* spp. (intertidal limpets) (E) recovered from archaeological excavations in the Hawaiian archipelago. Midden remains show decreases in marine fish and increases in domesticated mammals (pigs [*Sus scrofa]* and dogs [*Canis familiaris*]) through time, suggesting a change in basic subsistence patterns in Hawaiian prehistory with a decreased reliance on fish versus domesticates. Increases parrotfish and limpet species occurred from the late prehistoric and early historic period (∼AD 1400–1900+) suggesting release of these populations from exploitation pressure (E). Middens analyzed include the following sites: (A) Beach profile 1, and (B) Beach profile 4, Ke‘e Beach, Hā‘ena, Kaua‘i [36], [104], (C) Hālawa Dune Site, Moloka‘i [105], [106]; and, (D) Kuakini cave sites, Kailua-Kona, Hawai‘i Island [107]. Data for *Scarus* spp. are from Site 4853-1, Bellows Beach, Waimanalo, O‘ahu [39]; *Cellana* spp. data are from the Kalaupapa Peninsula, Moloka‘i [50].

To analyse archaeological data, we relied on well-established models derived from foraging theory to infer ecological impact on the basis of loss in abundance, size or diversity of taxa found in midden deposits [30], [31], [32]. This framework was used to interpret both general trends in a comprehensive review of middens across the MHI (Table S5) and in a more in-depth analysis of a few well-described middens that we used as representative sites (Figure 4). Together, these datasets were used to infer temporal changes in ecological conditions at the regional level in Hawaiian prehistory.

The archaeological record in Hawai‘i exhibits considerable variation in patterns of marine resource exploitation [27], [33] but despite these place-specific differences, some general patterns are detectable (Table S5). First, nearshore and reef-associated biota comprise the major marine source of protein and are more prevalent in the archaeological record than pelagic, riparian, estuarine and aquacultured species. Second, some studies show consistent evidence of overexploitation, but these patterns appear to be most consistent for nearshore shellfish species only (e.g. intertidal gastropods and mollusks) [34], [35], [36]. Overexploitation in these cases is evidenced in taxa size reductions and changes in the dominant taxa of species recovered through time, and these trends appear to be primarily associated with geographic factors. For example, faunal remains recovered at intermittent fishing camps where subsistence strategies relied more heavily on marine resources reflect more intensive exploitation than inland sites where agricultural and animal husbandry activities were developed (Table S5). In several well-described sites, fish remains recovered from archaeological deposits suggest a shift from a predominance of inshore carnivorous reef fish to inshore herbivorous reef fish [37], [38], [39]. This may have been caused by shifts in fishing methods through time to achieve better yields due to the higher prevalence of herbivorous versus carnivorous fish species in Hawaiian reef environments. An alternative explanation is that carnivores were overfished, but changes in fishing gears (e.g. fishhook type and function) recovered at these sites suggest that fishing methods changed as an adaptive strategy to local environmental conditions and resource availability. Finally, at some late prehistoric sites, differences in patterns of excavated marine resources have been interpreted as a reflection of social status (elite vs. commoner classes), by gender, and between habitation sites and special ritual sites [40], [41]. These studies draw on ethnohistoric accounts of these practices [26], [42], [43], which limited social access in late prehistory (>∼AD 1400) to some reef biota, particularly large carnivores and herbivores.

The considerable intra-site variability led us to examine a few well-described midden assemblages as representative sites to assess temporal patterns in subsistence strategies and estimate the ecological impact of human exploitation on reef ecosystems in prehistory (AD 1250–1778). We purposefully selected sites capable of supporting both hunter-gatherer activities and agricultural activities (‘mixed-use’ sites), which we felt were the most appropriate as representative sites for assessing regional-level changes in subsistence patterns. Considerable care must be taken in extrapolating ecological conditions from patterns of recovered faunal remains in archaeological sites [44], [45]. Temporal trends in faunal remains can be the result of human exploitation patterns, environmental changes affecting species, or shifts in social preferences or cultural traditions that influence the collection and disposal of a species. Trends may also be an artefact of sampling, site characteristics and data analyses (e.g. variable deposition rates, preservation processes, excavation techniques and differences in data reporting). To establish rigor and reliability in our analysis, we chose sites where all faunal remains had been identified by excavated strata, the chronology of the strata was well established and spanned a sufficient period of Hawaiian prehistory, and where excavation methodology and data reporting allowed for comparison. We also excluded sites with a low density of midden materials recovered to minimize the effects of sample size bias [44], [45]. Our use of percent bone weight as a comparative metric necessitated the exclusion of nearshore shellfish that are commonly found in middens and which probably comprised a significant portion of marine fauna consumed [35], [46]. Notably, our review of existing published data reveals a paucity of archaeological studies where these criteria for valid intra-site comparisons are met.

Our detailed analysis of faunal remains from these representative sites reveals a pattern of decline in reef fish relative to domesticated mammals as a primary source of meat-derived protein in Hawaiian prehistory (Figure 4). This analysis corroborates trends observed from other archaeological sites (Table S5) suggesting that marine exploitation was highest in the early period after Polynesian settlement and subsequently decreased through time (Figure 4). In the early prehistoric period (<AD 1400), decreases in the size of shellfish species and declines in high-value versus lower-value prey suggest that free-living reef guilds may have been depressed due to exploitation pressure by human foragers (Table S5, Figure 2). Similar impacts have been documented for other taxa, particularly terrestrial avifauna, and are consistent with a historical pattern of ecological transformation as the result of human arrival in pristine island environments [23], [34], [47], [48]. Though evidence is equivocal, it is likely that highly vulnerable marine megafauna (e.g. sea turtles, monk seals) may have experienced early and rapid population reductions during this period due to the synergist effects of human hunting and invasive species [49], including potential rat predation on turtle eggs and juveniles and deterrence of monk seals from utilizing haul out beaches by introduced pigs and dogs (Supporting Information S2).

By the late prehistoric period (> AD 1400), however, reef-derived protein sources are less prevalent than domesticates, suggesting a shift in the basic modes of subsistence and exploitation patterns in prehistoric Hawaiian societies (Figure 4). The trend of decreasing reef- versus land-derived protein can be explained by one of two competing (but non-exclusive) hypotheses. Either early Hawaiian societies overexploited reef biota and turned to land-based sources as a primary protein source or, alternatively, animal husbandry was developed as a primary mode of subsistence irrespective of reef resource condition. The archaeological record can provide an independent test of these competing hypotheses through examination of changes in the size of bones in archaeological deposits. Unfortunately, little research has been directed at quantifying temporal changes in the size of marine taxa recovered in archaeological deposits. No data are readily available for the early prehistoric period (< AD 1400), and only a few taxa have been studied in the late prehistoric and historic periods. Among these taxa, trends show that intertidal limpets and reef parrotfish bones recovered in midden deposits at two mixed-use sites [39], [50] increased in size in the late prehistoric and early historic periods (∼AD 1400–1800) (Figure 4E). These examples are limited in scope and sample size and should be interpreted with caution. Nonetheless, these patterns are consistent with the hypothesis that some reef populations may have been released from harvesting pressure due to displacement of effort from the sea to the land. More research is needed to determine if these trends hold across a wider array of taxa and sites and to further test hypotheses explaining the shift from reef- to land-based sources of protein.

In sum, intra-site trends in faunal remains suggest partial resource recovery as a result of decreased and differential exploitation of reef ecosystems from AD 1400–1800. Competing, but we suggest less likely, hypotheses that may serve to explain these trends include environmental changes affecting the availability of certain species, prey release due to reduction of higher trophic level predators, sampling artefacts, or changes in preferences or cultural traditions that influenced the collection of reef species. We conservatively estimate this recovery as not exceeding 20% for free-living guilds (Figure 2), which translates to a 10% recovery at the ecosystem level (Figure 3). Though some patterns in the archaeological record remain equivocal, the observed pattern of changes in subsistence and modes of production is consistent with other research documenting the intensification of agricultural activities in the late prehistoric period (AD 1400+) and later recorded ethnographic information documenting the agrarian subsistence focus of Hawaiian society at the time of western contact [51], [52], [53].

We also conducted a comprehensive review of observations made by explorers, traders and merchants, whaling and sealing crew members and captains, missionaries and Native Hawaiians about Hawaiian coral reef environments in the early historic period after western contact (AD 1778–1850+). Such observational data are often limited in precision but nonetheless can be used to derive valid information about the condition of marine ecosystems and social relationships with these environments [2], [19], [54], [55], [56], [57].

Though many westerners visited Hawai‘i after Cook's discovery of the archipelago in 1778, comments about the marine environment are limited to items mentioned in accounts of ship provisioning through trade with Hawaiian communities. Fish and shellfish were an object of minor trade with foreigners during this period, but there is no indication that this slight increase in demand could or did affect species abundance, and the demand for other provisions, including salt, pigs, and fresh water was much greater. Existing observations in published accounts about the archipelago, however, describe high abundances of invertebrates, reef fish, turtles, and predators in Hawaiian coral reef ecosystems (Table S6). For example, James Cook's ships purchased both fresh and salted fish in the 1770s including “cavalla” (reef jacks/trevally [*Carangidae*]), and in the late 1780s Nathaniel Portlock purchased “snappers, rock-cod, [reef piscivores]” from Ni‘ihau. In the mid-1780s, Colnett noted a high abundance of both fish and sea turtle at Ni‘ihau and described that Hawaiians from the southwest shore of O‘ahu had only “great quantitys” [sic] of fish to sell. Members of Vancouver's expedition in the early 1790s also described the large quantity of salted fish available at Ni‘ihau, and reef fish were so plentiful that they were traded to the people of Kaua‘i for cloth and mats. Quimper purchased “excellent snails” [intertidal/reef invertebrates] at both Kealekekua and at Waimea on Kaua‘i. Early visitors also described high abundances of predators in Hawaiian reef environments (Table S6). For example, Portlock observed an abundance of “very large sharks” throughout the archipelago and both Meares (1789) and Colnett (1786) observed abundant turtles, some of which were captured and stored in fishponds.

Early descriptions by foreigners are also corroborated by observations by Native Hawaiian historians known to have been experts in Hawaiian history, environments and traditions [58]. These accounts uniformly describe high abundances of reef species in nearshore environments during the early post-contact period (Table S6). For example, an observation of the west coast of O‘ahu during the 1790s described extremely high abundances of reef fish: “On this trip, there were so much fish caught that a stench rose up on the shore. People went from Ewa, Waianae and Waialua [districts of O‘ahu island] to get some fish but the supply was inexhaustible. The fish kept coming to the same place for several days” [59] (Table S6). The Native Hawaiian historian Samuel Kamakau also described incredible abundances of catches that are estimated to date to the same period (Table S6). Finally, legends from the preserved folklore of Native Hawaiian traditional accounts also describe high abundances of fish catches (Table S6). Though the literal truth of these legends is impossible to know, these accounts nonetheless describe an abundant marine environment.

In sum, multiple independent observations of high abundances of reef taxa in ethnohistoric and observational data indicate further recovery of free-living reef biota after western contact (Table S6). Coupled with archaeological analysis, these trends suggest a period of sustained recovery spanning the late prehistoric to the early historic period (∼AD 1400–1820) (Figure 3). Reef recovery ended in the early to mid-1800s as observations describe declining abundances in populations of the reef biota across all guilds (Figure 2) (Supporting Information S2). The anthropogenic stressors associated with these declines include overexploitation, land-based pollution (e.g., sedimentation) and a suite of other proximate stressors, which increased in scale and intensity due to changes in underlying economic systems, changes in population and demography, technological introductions and institutions in Hawai‘i (Figure 3, Table 1). These impacts intensified over the past 150+ years, with only a brief rebound in the 1940s due to the closing of nearshore marine areas during WWII (Figure 3).

### The Northwestern Hawaiian Islands

Coral reefs in the NWHI have functioned as a geographic refuge since prehistoric times due to the region's isolation, limited human habitability, and the dispersed geography of NWHI reefs and atolls, but they have not been free from human impact. The NWHI were also culturally protected as a sacred ancestral homeland from which life arises and spirits return after death [60]. Though prehistoric impacts appear to be minimal, reefs in the NWHI suffered impacts in the post-contact period from the same historical activities affecting MHI reefs, as fishers and maritime industries moved from locally accessible MHI reefs to resource pools further afield in the NWHI. The NWHI figured prominently in 19^th^ century colonial economies in the Pacific, as whaling, trading, sealing, and other commercial ventures resulted in heavy exploitation of marine resources, including monk seals, reef fish, turtles, sharks, pearl oysters and sea cucumbers.

Ecosystem protections were enacted in the early 1900s in the NWHI [61] and had the early effect of limiting foreign fleets (principally the Japanese) from accessing the NWHI for commercial extraction in coral reef habitats. Large-scale reef removal activities for facilities construction coupled with resource exploitation by domestic fleets, however, largely prevented those protections from having a discernable effect on the marine environment until after WWII. For example, several atolls were highly modified for the construction of runways or other facilities such as guano extraction operations. Though these activities were somewhat spatially limited, the impacts were not inconsiderable and resulted in permanent loss of coral reef habitat and alteration of circulation patterns in fragile atoll environments [62]. For example, at French Frigate Shoals over 500,000 m^3^ of dredged coral were used to build the runway, quadrupling the size of Tern Island; similar activities were undertaken at Midway Atoll.

Following WWII, commercial fishing operations continued but the domestic fleet remained fairly small over the past century (<12 vessels) [63], [64]. Over the past few decades, reef fisheries have narrowly focused on a few species, including demersal deep-reef piscivores (bottomfish) and lobsters, both of which have been intensively exploited. Recovery in the NWHI is indicated by numerous historical anecdotal accounts and contemporary ecological studies that have documented the healthy status of predator-dominated reef ecosystems in this region [65], [66], [67], [68]. Many reef species that were historically exploited have recovered. For example, green sea turtle nesting abundances have increased more than 500% in a 30-year time span following protection from exploitation [69]. Similarly, fisheries data show that the NWHI commercial fishery for predatory reef jacks and trevally (*Carangidae*) declined precipitously in the 1970s–80s due in part to concerns about ciguatera poisoning, and these species are now abundant in the NWHI [68]. Despite these positive trends, some species have never recovered from intensive commercial exploitation (e.g. monk seals, pearl oysters, spiny lobsters). The lack of recovery in these species has been attributed to a number of factors, including allee effects, interspecific interactions, time lags and large-scale shifts in climate and reef ecosystem productivity [70]. Despite lack of recovery in these specific populations, most populations are healthy and ecological guilds are intact in the NWHI [65], [66], [67], [68].

## Discussion

Coral reefs remained a focus for resource extraction throughout Hawaiian prehistory, but the development of large-scale agricultural and aquaculture complexes and increases in animal husbandry prevented societies from relying exclusively on reef environments for food resources. Our research suggests that reef recovery in the MHI during the late prehistoric period was probably attributed to shifts in the basic patterns of subsistence (Figure 4). These shifts appear to be related to the development and implementation of socio-cultural institutions in late prehistory (∼AD 1400–1500+) [71], which imposed hierarchical controls on resources and production systems. Though human population size remains poorly understood in the pre-censal period (before AD 1853), population likely increased exponentially during the early prehistoric period and then remained fairly stable during the late prehistoric period (Figure S6). Social institutions for reef management were probably codified during the rise of complex chiefdoms, which are associated with increased complexity in socio-political structures at a time in history when pre-contact human populations were believed to be at their zenith [58], [72], [73], [74] (Figure 3, Table 1, Figure S6). The ethnohistoric and archaeological record confirm that these systems included gender- and class-specific consumption restrictions on many marine species and a suite of coral reef ecosystem conservation strategies which may have enabled resource extraction while preventing overuse and collapse [26], [40], [75]. These strategies included direct measures such as time/area closures, size and species restrictions, and protection of spawning cycles, and indirect measures including large-scale aquaculture and restrictions on fishing effort through chiefly sponsorship and regulation of a professional fishing class. Resource protection measures were apparently robust due to incentives and draconian punishments for rule breakers and the efficacy of these institutions is evidenced by ethnographic information, anecdotal accounts by early observers and analysis of archaeological deposits (Supporting Information S2) [26], [40], [75].

The pattern observed in Hawaiian prehistory is similar to other island systems where the success of human colonizers occurred at the expense of vulnerable taxa [76], but active ecosystem engineering with conserving mechanisms occurred as part of longer-term cultural adaptation to island environments [29], [77]. Socio-cultural institutions and other adaptations (e.g. large-scale aquaculture) probably minimized risk in a stochastic environment subject to unpredictable disturbances such as droughts, floods, tsunamis, hurricanes and large storms. However, the causal mechanisms giving rise to these social institutions remain unclear. These systems may have arisen as an adaptive response to environmental drivers (e.g., diminishing returns on marine resource exploitation), to social drivers (e.g., the need for increased cooperation or the rise of large-scale chiefdoms), or as a result of some complex interaction of these factors [78], [79], [80].

Subsistence exploitation in prehistory altered the structure of coral reef ecosystems through the removal of large predators and high-value prey, similar to changes observed in modern contexts [81], [82]. However, exploitation of reef resources appears to have been sustainable in late prehistory even though MHI reef ecosystems were heavily modified from pre-exploitation states. For select marine species, the development of conservation-like sanctions appears to be a result of reciprocal interactions, whereby the most vulnerable taxa were reduced faster than the development of cultural controls, and high-value resources that were more resistant to initial impacts become subject to sanctions [75], [80]. For example, socio-cultural practices restricted access to vulnerable taxa such as sea turtles and sharks, while invertebrates and most reef fish populations were protected primarily via periodic time/area closures [26].

After western contact, reef recovery in the MHI is attributed to extensive indigenous depopulation due to disease epidemics and changes in labor and the modes of production (Figures 3, Table 1). The introduction of western diseases, to which Native Hawaiians exhibited little immunity, resulted in widespread and catastrophic depopulation among the indigenous population, which continued until the late 1800s [83], [84] (Figure S6). The decline in human population during this period is associated with reef recovery, however, other underlying social factors, including changes in economic systems also serve to explain reef recovery patterns. During this period, Hawaiian chiefs were re-directing labor away from traditional reef fishing practices and towards emerging commodities markets and foreign commercial enterprises including whaling, trading and other ventures. As a result, chiefly sponsorship of the trades that supported the professional fishing class declined significantly [52], [85], [86]. These shifts in the economic modes of production and depopulation of the labor force led to the abandonment of large-scale agricultural complexes within decades of European contact [52], [53] and probably reduced pressure on natural resources and the ecosystems in which they were embedded. Independent observations from western observers also corroborate how changes in population and labor patterns impacted food production and subsistence, resulting in some cases in famine [87], [88], [89], [90], [91]. For example, the Native Hawaiian historian Samuel Kamakau described food scarcity in the early 1800s as a result of changes in how chiefs redistributed labor away from food production systems and towards the harvesting of trade commodities such as sandalwood: “The rush of labor to the mountains brought about a scarcity of cultivated food throughout the whole group. The people were forced to eat herbs and tree ferns…The chief [Kamehameha] immediately declared all sandalwood to be the property of the government and ordered the people to devote only part of their time to its cutting and to return to the cultivation of the land” [92∶204]. Several investigators have commented that these changes are likely to have reduced pressure on natural resources through reduced exploitation effort in both marine and terrestrial systems [50], [85], [86], [88], which is consistent with uniform descriptions of high abundances of reef biota during this period (Table S6). The environmental re-wilding of Hawaiian coral reefs during the post-contact era echoes similar changes observed in terrestrial ecosystems after indigenous population collapse [93], [94].

In the NWHI, reef recovery is associated with 100+ years of environmental protections, human depopulation, ecological restoration efforts and decreases in the extractive capacity, spatial extent, and range of species targeted by commercial operations (Figure 3, Table 1). After WWII, reef fisheries in inshore reef zones were discontinued by the late 1950s, and economic and geographic remoteness have served to limit the fleet size and impact on NWHI reefs [95]. Exploitation of reef bottomfish and lobsters continued after WWII, but both fisheries are now closed and there is little evidence that these fisheries impacted ecosystem structure and function at the guild level. More research, however, is needed to fully document and understand the historical legacies of fisheries in the NWHI. Human depopulation after WWII and further environmental protections extended to reef ecosystems further limited human impacts to these atoll ecosystems.

Long-term trajectories of change in reef ecosystems in the Hawaiian archipelago reveal that coral reefs may be resilient to human activities if the intensity and ecological breadth (number of guilds affected) of proximate stressors are reduced over long time periods (decades+) and large spatial scales (>10^3^ km^2^). Though the underlying social factors responsible for recovery differ among regions (Table 1), both recovery periods appear to be initiated by cumulative and overlapping factors (Figure 3), which acted in concert to limit proximate human stressors of reef systems to sustainable levels. Recovery at the ecosystem level was driven primarily by population increases in free-living/mobile guild species rather than biota comprising the architectural/sedentary guilds (Figures 2, S4). At the species level, reef taxa exhibit variable recovery responses, which appear to be based on species vulnerability to disturbance, recovery potential and the prior intensity of exploitation.

As in studies in terrestrial ecosystems [9], [10], [96], our findings negate simplistic cause-consequence relationships that are often advanced to explain environmental degradation. Instead, a complex set of historical events has shifted the underlying demographic, economic, technological and institutional systems that structure social-ecological relationships in Hawaiian coral reef systems over the past 700 years (Table 1). For example, recovery in the MHI was first initiated through changes in subsistence patterns and the implementation of social institutions for reef management, but was later mediated through effects of depopulation due to disease epidemics and shifts in economies and labor patterns. In the NWHI, recovery is associated with changes in human institutions for ecosystem protection, enduring remoteness from economic markets, and human depopulation after WWII. Social factors mediating ecosystem recovery and decline had differential impacts among guilds (Figure 2) but cumulative trajectories of change at the ecosystem level (Figure 3) reflect the complexity and diversity of drivers (+/−) affecting reef species (Table 1).

**Figure 5:**
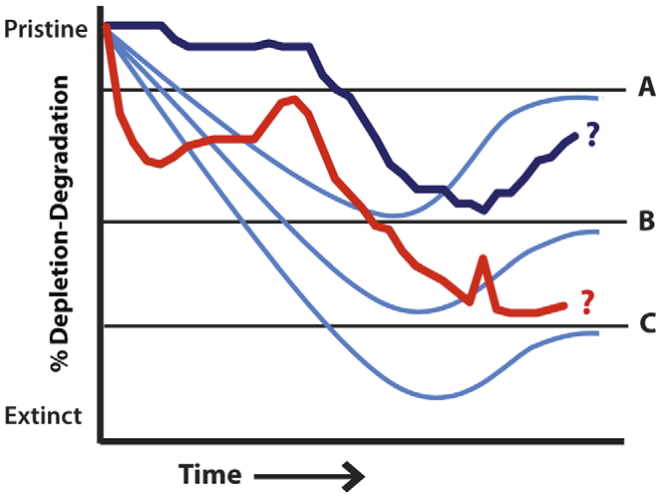
A model showing hypothesized trajectories of ecosystem conditions and future recovery potential available (light blue) under different thresholds (A–C) of degradation. Long-term ecosystem trajectories for the MHI (red) and the NWHI (dark blue) are superimposed on the model.

Our results indicate that while coral reef ecosystems in the MHI are highly degraded, NWHI reefs are healthy and in good condition by global standards (Figure S5). Reefs in the MHI have been declining over the past 150+ years and deleterious phase shifts observed in other regional reef ecosystems [97], [98] point to the existence of degradation thresholds beyond which recovery is doubtful (Figure 5). Historical recoveries suggest that reversing trajectories of decline will require major steps to protect a large range of habitat types over large spatial scales in order to hedge against further declines as the intensity of global stressors ratchets upward. In coastal environments like Hawai‘i that exhibit socio-cultural and ecological heterogeneity, place-based conservation strategies developed in a participatory process with resource users are most likely to be effective. However, the mechanisms enabling historical recovery periods also support the establishment of holistic marine conservation planning targets to facilitate recovery (e.g. 20–30% placed in no-take reserves [99], [100], [101]) and robust social institutions with the capacity to enforce regulations and build social adaptive capacity [102].

In the NWHI, where protections are in place and reefs are in a recovery mode, reef ecosystems provide an opportunity to understand the role of historical disturbance legacies in determining the limits to recovery (Figure 5). Achieving ecological restoration goals in the NWHI and elsewhere will require explicit consideration of whether future, desired resource conditions are realistic or achievable given past human disturbances that have forever altered ecosystem trajectories [30], [70], [103]. Controlled manipulations to rehabilitate exploited and vulnerable populations, for example, may be warranted for species that have undergone severe reductions in the past.

Integrated approaches to understanding the co-evolution of human and natural systems are necessary to understand the complex interactions between societies and the ecosystems upon which they rely. Long-term social-ecological reconstructions allow us to address contemporary environmental challenges by providing baselines of previous ecosystem conditions and reciprocal cultural responses, the states and transformations these linked systems have undergone, and the factors associated with sustainability, degradation or collapse [6], [8]. Our reconstruction reveals that human agency is partly responsible for environmental recovery periods and that not all human-environment interactions lead to irreversible deleterious outcomes. Understanding environmental challenges of the past provides promise for contemporary efforts to manage ecosystems and societies toward social-ecological sustainability.

## Supporting Information

Figure S1 Data summary by guild through time used to reconstruct coral reef ecosystem conditions in the Hawaiian archipelago. Number of observations (N) are displayed on the right side of the graph, and observations are plotted by each year for each guild on the horizontal axis. Archaeological data are displayed as one observation per earliest reliable date of the excavated site, but most sites spanned longer time series than are graphically indicated (see Dates, Table S5).
(TIFF)

Figure S2 Changes in ecological conditions through time for free-living guilds (large carnivores; large herbivores; small carnivores; small herbivores) for the Main Hawaiian Islands (A–D) and the Northwestern Hawaiian Islands (E–H). The vertical axis represents ecological condition as a percentage of depletion-degradation, with 0% = pristine and 100% = globally extinct. Time is represented on the horizontal axis (AD 1250–2009); 100-year increments are used in the prehistoric period (AD 1250–1778) and 20-year increments are used for the historic and contemporary period (AD 1778–2009).
(TIFF)

Figure S3 Changes in ecological conditions through time for architectural guilds (corals; seagrasses/algae; suspension feeders & detritivores) for the Main Hawaiian Islands (A–C) and the Northwestern Hawaiian Islands (D–F). The vertical axis represents ecological condition as a percentage of depletion-degradation, with 0% = pristine and 100% = globally extinct. Time is represented on the horizontal axis (AD 1250–2009); 100-year increments are used in the prehistoric period (AD 1250–1778) and 20-year increments are used for the historic and contemporary period (AD 1778–2009).
(TIFF)

Figure S4 Trajectories of change in free-living (large carnivores; large herbivores; small carnivores; small herbivores) and architectural/sedentary guilds (corals; seagrasses/algae; suspension feeders & detritivores) from AD 1250–2009 for the Main Hawaiian Islands (MHI) (pink, red lines) and the Northwestern Hawaiian Islands (NWHI) (dark & light blue lines). Reef recovery in the MHI (∼AD 1450–1800) and the NWHI (∼AD 1950–2009) was driven primarily by free-living guilds.
(TIFF)

Figure S5 Comparison of Hawaiian coral reef ecosystem trajectories with global estimates of reef conditions. Trajectories include the Main Hawaiian Islands (MHI, red) and the Northwestern Hawaiian Islands (NWHI, blue). Periods of reef recovery in the MHI (AD 1400–1820) and the NWHI (AD 1950–2009) are indicated where the trend line is yellow. Current ecosystem conditions for the Hawaiian Islands are compared with global assessments of coral reefs reported by Pandolfi et al. (2003), which shows that regions in the Hawaiian archipelago (MHI, NWHI) occupy the distal ends of the global spectrum of observed conditions in coral reef ecosystems on an axis of depletion-degradation. Time is represented on the horizontal axis (AD 1250–2009); on the horizontal axis 100-year increments are used in the prehistoric period (AD 1250–1778 AD) and 20-year increments are used for the historic and contemporary period (AD 1778–2009).
(TIFF)

Figure S6 Human population in Hawai‘i, AD 1250–2010. A demographic model developed by Dye and Komori (1992) (as reviewed by Kirch 2007b), was used for estimating pre-censal population, which remains poorly understood and the subject of some controversy (Kirch 2007b). High and low bounds (grey lines) and an estimated mean (black line) are presented for this period (AD 1250–1832). Census data were used for the period 1853–2010 (Schmitt 1977; US Census Bureau 2010) (black line).
(TIFF)

Table S1 Descriptions and examples of biota comprising of coral reef guilds.
(DOCX)

Table S2 Ecological states (‘EcoStates’) and criteria used to assess the condition of coral reef guilds for different data types on an ordinal scale. General criteria are presented as headings in each row of the second column. General criteria developed by Jackson et al. (2001) and Pandolfi et al. (2003, 2005) were further refined to evaluate the level of impact by data type. These criteria were developed in recognition that the quantitative scoring of different studies and data types required a common methodology for evaluation, but that specific criteria were needed in order to evaluate each data type. For example, corals are rated by the total amount that have declined or are at risk, with 0–10% at risk being pristine, 11–29% abundant/common, 30–59% depleted/uncommon, 60–89% rare, 90–99% ecologically extinct, and 100% globally extinct. For states 2–5, more specific criteria used for each type of data (in italics) are described.
(DOCX)

Table S3 Summary of data types and number of records used to reconstruct ecological conditions through time. Archaeological records include published studies on marine fauna from archaeological sites (See Table S4, below). For qualitative and ethnographic accounts, one record is one description from one publication; multiple descriptions from reports are extracted in many cases. Records for annual fisheries reports include one published report or dataset, which contained catch data for multiple species for one year.
(DOCX)

Table S4 Archaeological sites in the MHI and studies that report marine fauna in midden deposits. Citation references are in Appendix A. NWHI sites (not listed in table) include sites on Nihoa and Mokumanamana (Necker) Islands (Emory 1928; Cleghorn 1988; Kikiloi 2010; K. Kikiloi, pers. comm.). No midden remains have been discovered in other NWHI sites (Apple 1973; Ziegler 1990).
(DOCX)

Table S5 Summary of trends in faunal remains recovered from excavated middens in archaeological sites in the main Hawaiian Islands, sorted by date. Changes in ecological state (EcoState) are denoted as positive (up arrow), negative (down arrow), or not able to be determined (−) for specific time periods based on summary of major findings. Abbreviations for ecological guilds as follows: SC  =  small carnivores; SH  =  small herbivores; LH  =  large herbivores; LC  =  large carnivores. *indicates sites that have not been re-dated with modern methods; as such dates must be viewed with caution. Numbered citations are listed below the table.
(DOCX)

Table S6 Representative observations of the ecological condition of reef biota in the Main Hawaiian Islands during the prehistoric and early historic period. *exact dates for these observations are not described; estimates are derived from descriptions of authors or commentators.
(DOCX)

Supporting Information S1 Bibliography of sources.
(DOCX)

Supporting Information S2 Summary narratives of the primary data used to reconstruct ecological conditions and anthropogenic impacts are presented by guild. At the beginning of each guild subsection, tables summarize a timeline specific to the guild and include descriptions of major events, intensity of proximate stressors and the quantitative EcoState scores assigned to different time periods (Tables A–F). In the summary narratives that follow these tables, a chronological overview synthesizes primary data from multiple data sources and types. These syntheses by guild justify the quantitative scores determined for guild EcoStates and proximate stressor regimes. Ecological changes are reconstructed from multiple lines of evidence. Guild summary narratives are supplemented with additional data in the form of endnotes. In the narratives, terms are used to refer to specific periods in Hawaiian history; these include: 1) prehistoric: AD 1250–1778; 2) historic: AD 1778–1900; and, 3) modern: AD 1900+.
(DOCX)
