# Supplementary figures and images for: Historical Reconstruction Reveals Recovery in Hawaiian Coral Reefs
Source: PLoS One. 2011 Oct 3;6(10):e25460. doi: 10.1371/journal.pone.0025460 (PMC3184997; doi:10.1371/journal.pone.0025460)

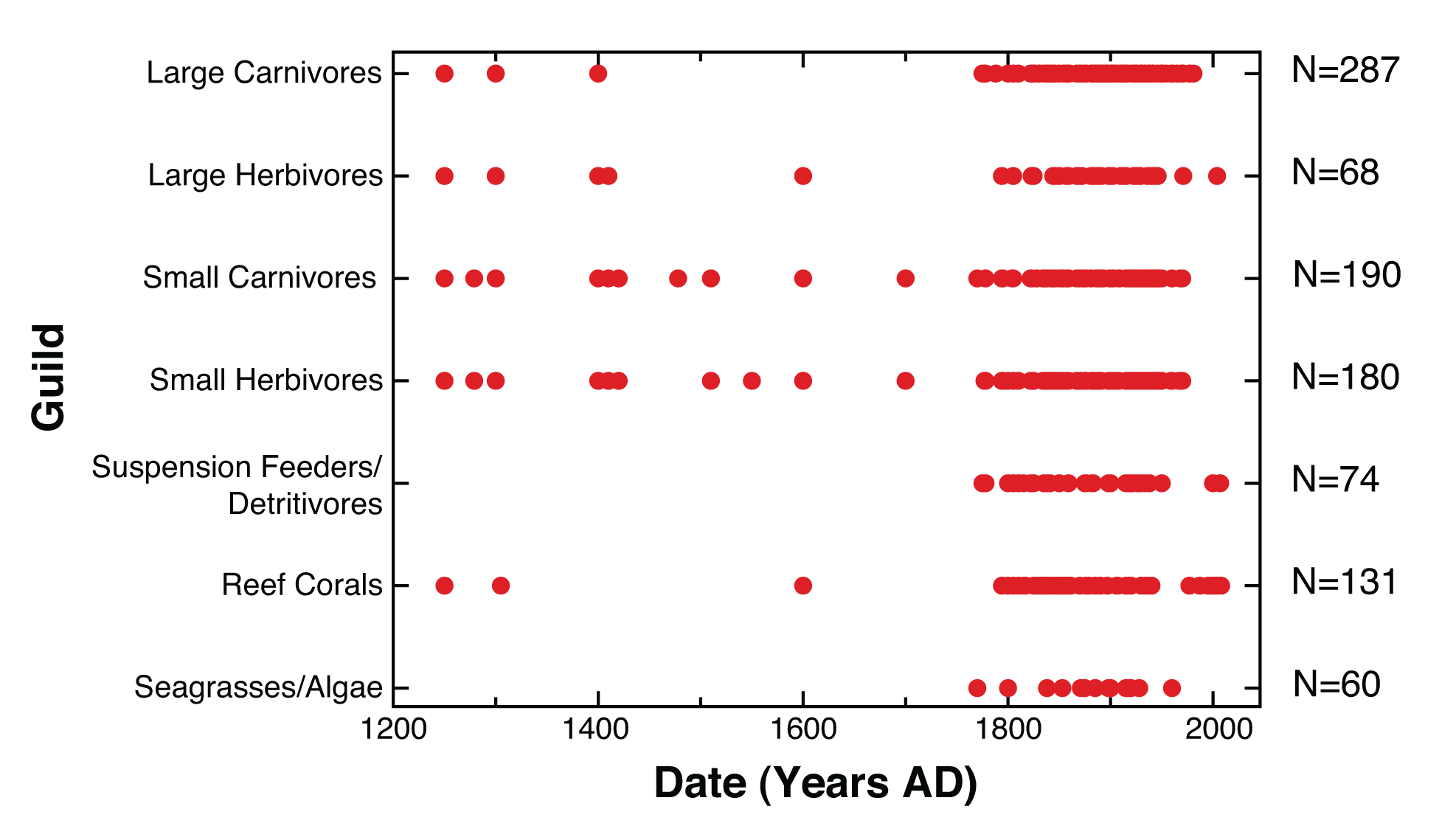

Supplement: Figure S1 — Data summary by guild through time used to reconstruct coral reef ecosystem conditions in the Hawaiian archipelago. Number of observations (N) are displayed on the right side of the graph, and observations are plotted by each year for each guild on the horizontal axis. Archaeological data are displayed as one observation per earliest reliable date of the excavated site, but most sites spanned longer time series than are graphically indicated (see Dates, Table S5). (TIFF) [file pone.0025460.s001.tiff]

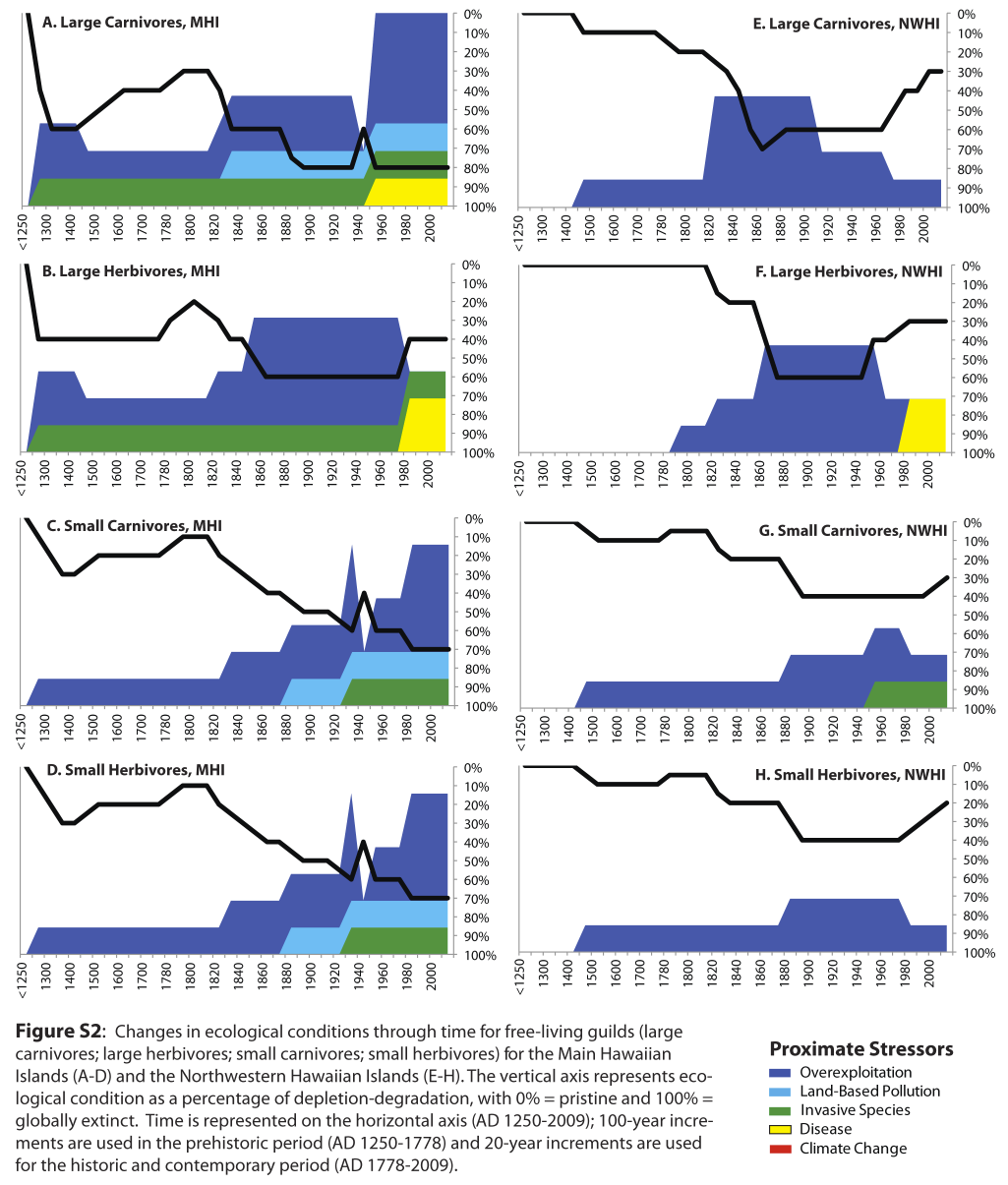

Supplement: Figure S2 — Changes in ecological conditions through time for free-living guilds (large carnivores; large herbivores; small carnivores; small herbivores) for the Main Hawaiian Islands (A–D) and the Northwestern Hawaiian Islands (E–H). The vertical axis represents ecological condition as a percentage of depletion-degradation, with 0% = pristine and 100% = globally extinct. Time is represented on the horizontal axis (AD 1250–2009); 100-year increments are used in the prehistoric period (AD 1250–1778) and 20-year increments are used for the historic and contemporary period (AD 1778–2009). (TIFF) [file pone.0025460.s002.tiff]

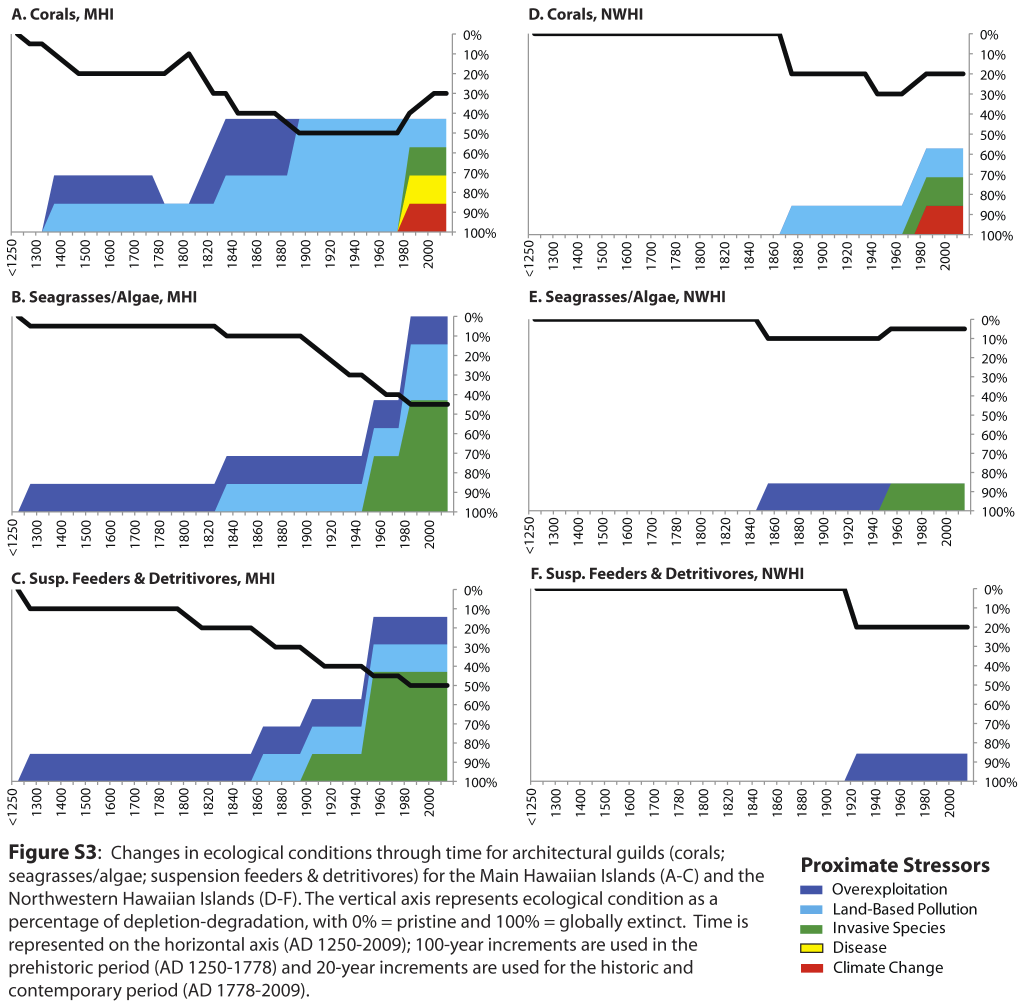

Supplement: Figure S3 — Changes in ecological conditions through time for architectural guilds (corals; seagrasses/algae; suspension feeders & detritivores) for the Main Hawaiian Islands (A–C) and the Northwestern Hawaiian Islands (D–F). The vertical axis represents ecological condition as a percentage of depletion-degradation, with 0% = pristine and 100% = globally extinct. Time is represented on the horizontal axis (AD 1250–2009); 100-year increments are used in the prehistoric period (AD 1250–1778) and 20-year increments are used for the historic and contemporary period (AD 1778–2009). (TIFF) [file pone.0025460.s003.tiff]

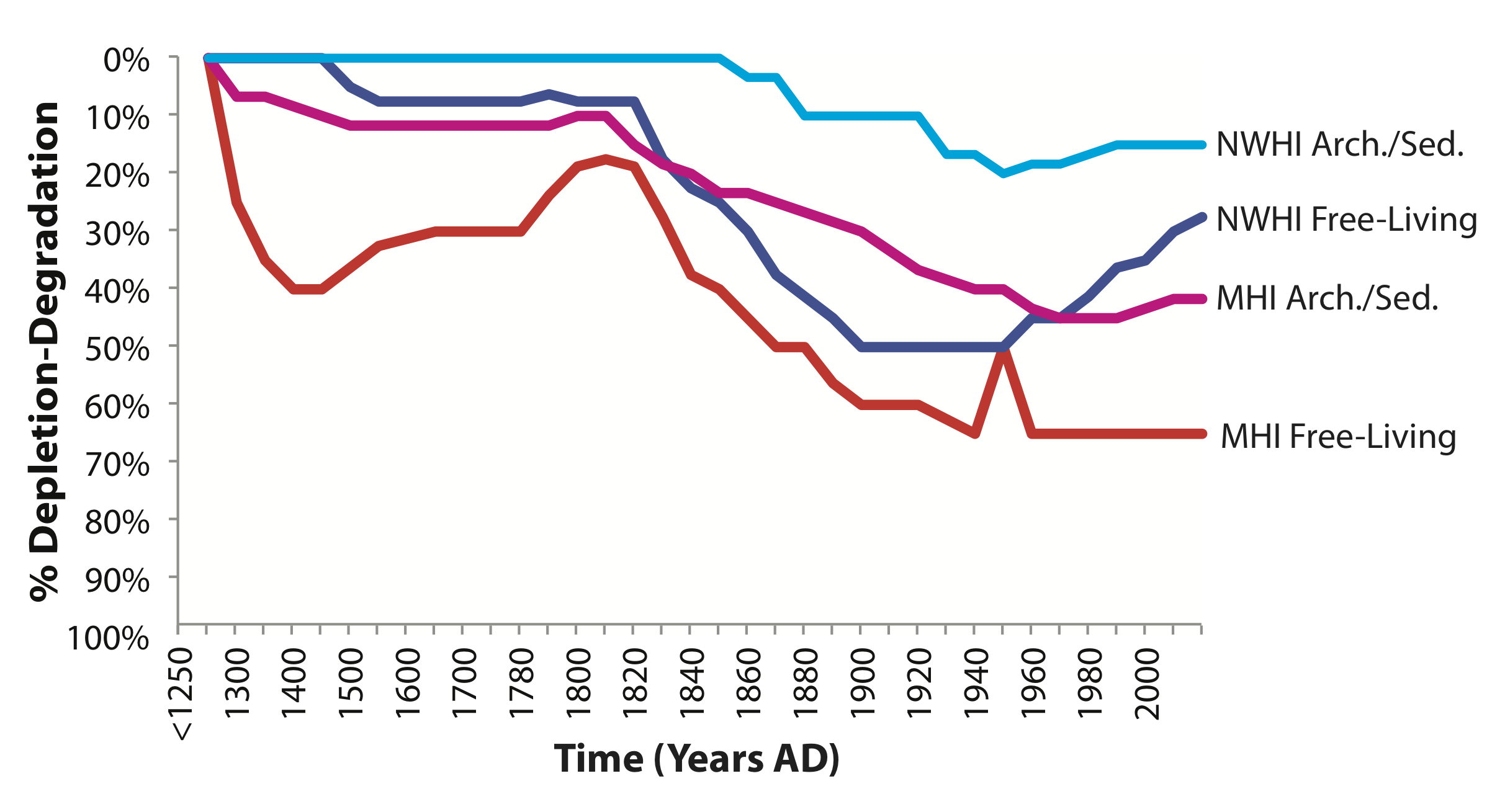

Supplement: Figure S4 — Trajectories of change in free-living (large carnivores; large herbivores; small carnivores; small herbivores) and architectural/sedentary guilds (corals; seagrasses/algae; suspension feeders & detritivores) from AD 1250–2009 for the Main Hawaiian Islands (MHI) (pink, red lines) and the Northwestern Hawaiian Islands (NWHI) (dark & light blue lines). Reef recovery in the MHI (∼AD 1450–1800) and the NWHI (∼AD 1950–2009) was driven primarily by free-living guilds. (TIFF) [file pone.0025460.s004.tiff]

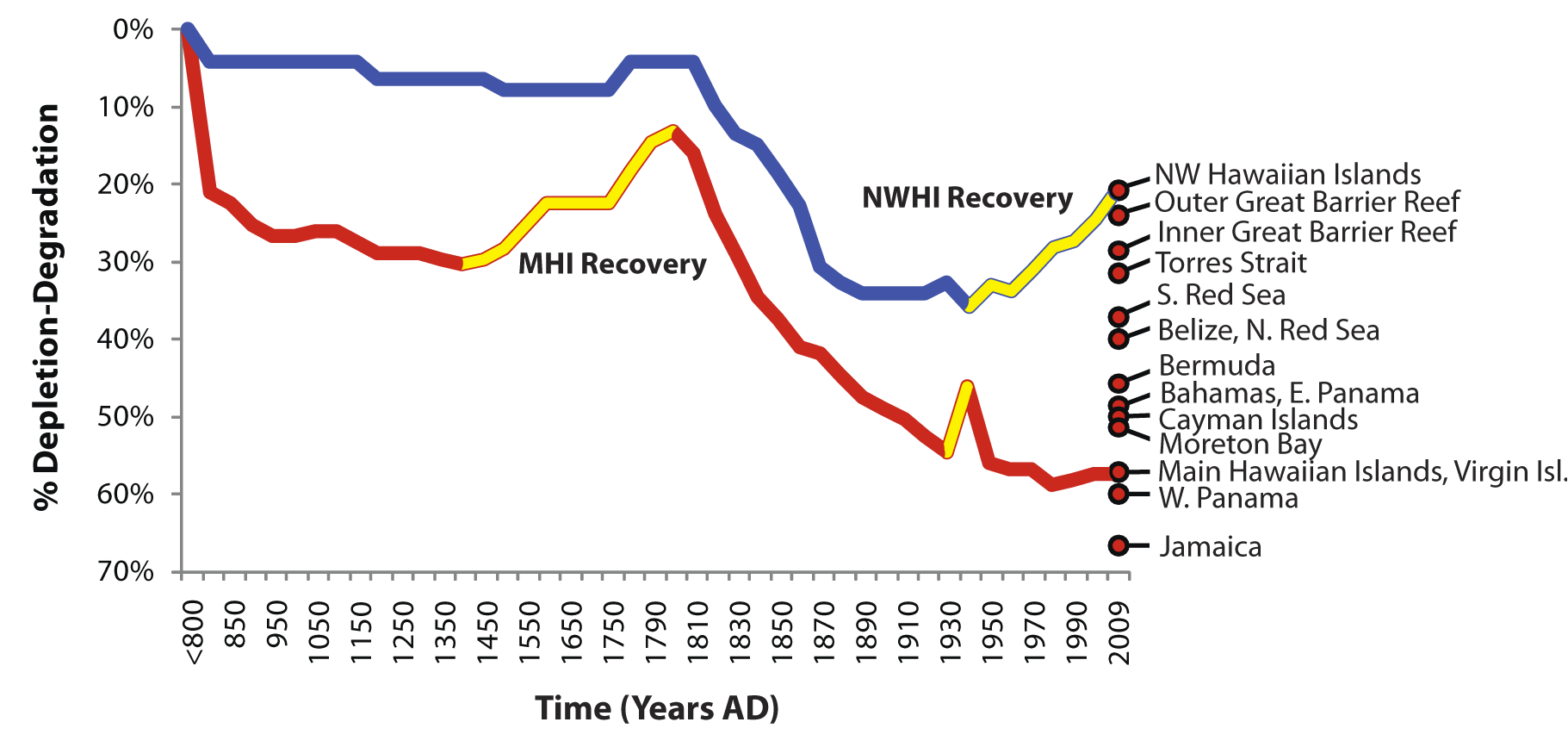

Supplement: Figure S5 — Comparison of Hawaiian coral reef ecosystem trajectories with global estimates of reef conditions. Trajectories include the Main Hawaiian Islands (MHI, red) and the Northwestern Hawaiian Islands (NWHI, blue). Periods of reef recovery in the MHI (AD 1400–1820) and the NWHI (AD 1950–2009) are indicated where the trend line is yellow. Current ecosystem conditions for the Hawaiian Islands are compared with global assessments of coral reefs reported by Pandolfi et al. (2003), which shows that regions in the Hawaiian archipelago (MHI, NWHI) occupy the distal ends of the global spectrum of observed conditions in coral reef ecosystems on an axis of depletion-degradation. Time is represented on the horizontal axis (AD 1250–2009); on the horizontal axis 100-year increments are used in the prehistoric period (AD 1250–1778 AD) and 20-year increments are used for the historic and contemporary period (AD 1778–2009). (TIFF) [file pone.0025460.s005.tiff]

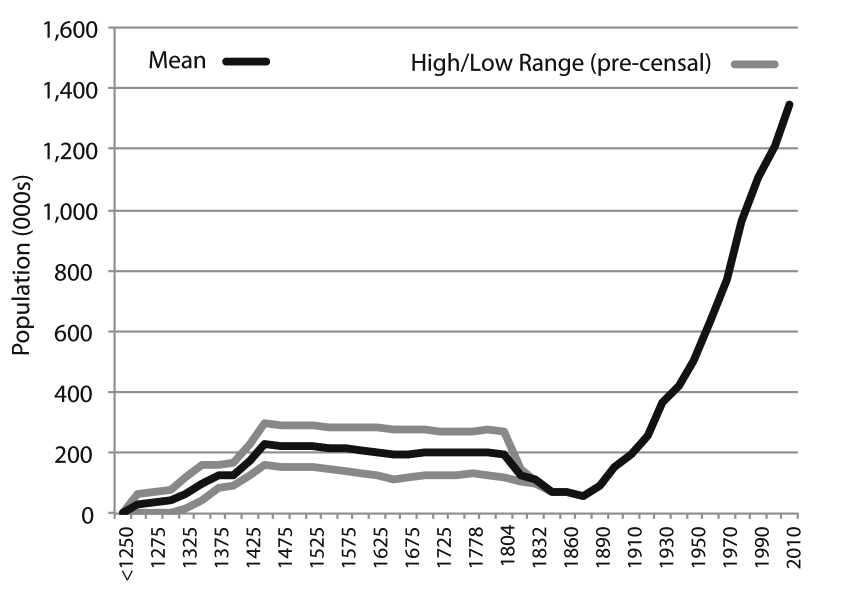

Supplement: Figure S6 — Human population in Hawai‘i, AD 1250–2010. A demographic model developed by Dye and Komori (1992) (as reviewed by Kirch 2007b), was used for estimating pre-censal population, which remains poorly understood and the subject of some controversy (Kirch 2007b). High and low bounds (grey lines) and an estimated mean (black line) are presented for this period (AD 1250–1832). Census data were used for the period 1853–2010 (Schmitt 1977; US Census Bureau 2010) (black line). (TIFF) [file pone.0025460.s006.tiff]
