# Supplementary material for: Historical Reconstruction Reveals Recovery in Hawaiian Coral Reefs
Source: PLoS One. 2011 Oct 3;6(10):e25460. doi: 10.1371/journal.pone.0025460 (PMC3184997; doi:10.1371/journal.pone.0025460)
Supplement: Table S1 — Descriptions and examples of biota comprising of coral reef guilds. (DOCX) [file pone.0025460.s007.docx]

Table S1: Descriptions and examples of biota comprising of coral reef guilds.

| **Guilds** | **Type** | **Example** |
| --- | --- | --- |
| Large Carnivores | Free-Living | Sharks, Monk Seals, Barracuda, Large Groupers |
| Large Herbivores | Free-Living | Green Sea Turtle |
| Small Carnivores | Free-Living | Most Reef Fish & Invertebrates |
| Small Herbivores | Free-Living | Parrotfish & other Reef Fish; Sea Urchins |
| Reef Corals | Sessile/Architectural | Pocillipora, Montipora, Porites stony coral spp. |
| Seagrasses/Algae | Sessile/Architectural | Macroalgae, Seagrasses |
| Suspension Feeders & Detritivores | Sessile/Architectural | Sponges, Pearl Oysters |
