# Supplementary material for: Historical Reconstruction Reveals Recovery in Hawaiian Coral Reefs
Source: PLoS One. 2011 Oct 3;6(10):e25460. doi: 10.1371/journal.pone.0025460 (PMC3184997; doi:10.1371/journal.pone.0025460)
Supplement: Table S2 — Ecological states (‘EcoStates’) and criteria used to assess the condition of coral reef guilds for different data types on an ordinal scale. General criteria are presented as headings in each row of the second column. General criteria developed by Jackson et al. (2001) and Pandolfi et al. (2003, 2005) were further refined to evaluate the level of impact by data type. These criteria were developed in recognition that the quantitative scoring of different studies and data types required a common methodology for evaluation, but that specific criteria were needed in order to evaluate each data type. For example, corals are rated by the total amount that have declined or are at risk, with 0–10% at risk being pristine, 11–29% abundant/common, 30–59% depleted/uncommon, 60–89% rare, 90–99% ecologically extinct, and 100% globally extinct. For states 2–5, more specific criteria used for each type of data (in italics) are described. (DOCX) [file pone.0025460.s008.docx]

Table S2: Ecological states (‘EcoStates’) and criteria used to assess the condition of coral reef guilds for different data types on an ordinal scale. General criteria are presented as headings in each row of the second column. General criteria developed by Jackson et al. (2001) and Pandolfi et al. (2003, 2005) were further refined to evaluate the level of impact by data type. These criteria were developed in recognition that the quantitative scoring of different studies and data types required a common methodology for evaluation, but that specific criteria were needed in order to evaluate each data type. For example, corals are rated by the total amount that have declined or are at risk, with 0-10% at risk being pristine, 11-29% abundant/common, 30-59% depleted/uncommon, 60-89% rare, 90-99% ecologically extinct, and 100% globally extinct. For states 2-5, more specific criteria used for each type of data (in italics) are described.

| **EcoState** | **Criteria for Assessment** |
| --- | --- |
| 1. Pristine | No evidence of human presence or use. |
| 2. Abundant/Common | Human use with no evidence of reduction of marine resource.  *Archaeological*: No shifts in abundance of organisms or size of organisms harvested  *Anecdotal & Ethnohistoric*: Observations lack any evidence of significant reduction at the population level; individual accounts describe taxa as common  *Contemporary Ecological*: Evidence of use or harvest shows increasing catch; most economically valuable and highest-value species are major focus of harvesting efforts |
| 3. Depleted/Uncommon | Human use and evidence of reduced abundance.  *Archaeological*: Observed shift in assemblages to smaller sized organisms; decrease in abundance of marine organisms; or changes in the proportional representation of species (diet breadth)  *Anecdotal & Ethnohistoric*: Observations of consistent harvesting or use of resource with some indication of use beyond the subsistence scale. Persistent use in construction activities  *Contemporary Ecological*: Persistent use of resource; catches are level, with no major increases or decreases |
| 4. Rare | Evidence of severe human impact.  *Archaeological*: Observations of decreases in size and abundance in assemblages over larger geographic ranges (e.g. over whole coastlines or islands); Significant reductions in sizes, abundances and increasing diversity of organisms in assemblages  *Anecdotal & Ethnohistoric*: Observations of commercial or industrial scale harvesting, large-scale removal, or verbal accounts of significant declines in species availability for subsistence fishing  *Contemporary Ecological*: Rate of resource extraction exceeds natural replenishment; catch records are declining |
| 5. Ecologically extinct | Rarely observed & further reduction would have no further environmental effect.  *Archaeological*: Local extirpation of populations; Decreases in size or abundance followed by absence in archaeological assemblages; Presence  absence observed in the majority of midden deposits reviewed  *Anecdotal & Ethnohistoric*: Rarely observed or described as having disappeared; observation of individual species worthy of publication  *Contemporary Ecological*: Closure of fishery due to overfishing; fishery not economically viable; species considered worthy of special protection via statute |
| 6. Globally extinct | No longer in existence. |
