# Supplementary material for: Historical Reconstruction Reveals Recovery in Hawaiian Coral Reefs
Source: PLoS One. 2011 Oct 3;6(10):e25460. doi: 10.1371/journal.pone.0025460 (PMC3184997; doi:10.1371/journal.pone.0025460)
Supplement: Table S3 — Summary of data types and number of records used to reconstruct ecological conditions through time. Archaeological records include published studies on marine fauna from archaeological sites (See Table S4, below). For qualitative and ethnographic accounts, one record is one description from one publication; multiple descriptions from reports are extracted in many cases. Records for annual fisheries reports include one published report or dataset, which contained catch data for multiple species for one year. (DOCX) [file pone.0025460.s009.docx]

Table S3: Summary of data types and number of records used to reconstruct ecological conditions through time. Archaeological records include published studies on marine fauna from archaeological sites (See Table S4, below). For qualitative and ethnographic accounts, one record is one description from one publication; multiple descriptions from reports are extracted in many cases. Records for annual fisheries reports include one published report or dataset, which contained catch data for multiple species for one year.

| **Data Type** | **Number of Records** |
| --- | --- |
| 1. Archaeological Studies | 46 (17 total sites) |
|  |  |
| 2. Qualitative Anecdotes & Ethnographic Accounts |  |
| Guild |  |
| Reef Corals | 131 |
| Seagrasses/Algae | 60 |
| Suspension Feeders & Detritivores | 74 |
| Small Herbivores | 180 |
| Small Carnivores | 190 |
| Large Herbivores | 68 |
| Large Carnivores | 287 |
| Sub-Total | 990 |
| 3. Annual Fisheries Reports |  |
| US Bureau of Fisheries (1900, 1903) | 2 |
| Territory of Hawai‘i, Division of Fish & Game (1926-1938) | 13 |
| State of Hawai‘i, Division of Aquatic Resources (1948-2008) | 40 |
| Sub-Total | 55 |
|  |  |
