# Supplementary material for: Historical Reconstruction Reveals Recovery in Hawaiian Coral Reefs
Source: PLoS One. 2011 Oct 3;6(10):e25460. doi: 10.1371/journal.pone.0025460 (PMC3184997; doi:10.1371/journal.pone.0025460)
Supplement: Table S4 — Archaeological sites in the MHI and studies that report marine fauna in midden deposits. Citation references are in Appendix A. NWHI sites (not listed in table) include sites on Nihoa and Mokumanamana (Necker) Islands (Emory 1928; Cleghorn 1988; Kikiloi 2010; K. Kikiloi, pers. comm.). No midden remains have been discovered in other NWHI sites (Apple 1973; Ziegler 1990). (DOCX) [file pone.0025460.s010.docx]

Table S4: Archaeological sites in the MHI and studies that report marine fauna in midden deposits. Citation references are in Appendix A. NWHI sites (not listed in table) include sites on Nihoa and Mokumanamana (Necker) Islands (Emory 1928; Cleghorn 1988; Kikiloi 2010; K. Kikiloi, pers. comm.). No midden remains have been discovered in other NWHI sites (Apple 1973; Ziegler 1990).

| **Island** | **Region/Area** | **Areas Excavated** | **Citations** |
| --- | --- | --- | --- |
| Hawai‘i | NW Hawai‘i/ Kohala | Koai‘e coastal village & Lapakahi upland | Pearson 1969; Newman 1970;Tuggle & Griffin 1973; Rosendahl 1994 |
| Hawai‘i | South Point/ Ka‘u | Pakini-Nui & Pakini-Iki, Wai‘ahukini | Sinoto & Kelly 1975; Goto 1984, 1986, 1990; Dye 1992 |
| Hawai‘i | W. Hawai‘i | Kalahuipua‘a rockshelter, Makaiwa Bay | Kirch 1979b, 1982a; Hommon 1983 |
| Hawai‘i | W. Hawai‘i/ N. Kona | Kahalu‘u | Kirch 1973a |
| Hawai‘i | W. Hawai‘i/ Kailua-Kona | Kuakini Cave Sites D7-27 & D8-33 | Schilt 1984 |
| Kaua‘i | N. Kaua‘i/ Nā Pali | Nu‘alolo Kai | Soehren n.d.; Gordon 1993; Morrison & Hunt 2007 |
| Kaua‘i | S. Kaua‘i/ Māhā‘ulepū | Makauwahi Cave | Kikuchi & Burney 1998; Burney et al. 2001; Burney & Kikuchi 2006; Burney, pers. comm., 2009 |
| Kaua‘i | N. Kaua‘i/ Halele‘a | Hā‘ena, Ke‘e Beach | Griffin et al. 1977; Hammatt et al. 1978; Griffin 1984 |
| Lana‘i | S. Lana‘i | Hulopo‘e | Tomonari-Tuggle et al. 2000 |
| Maui | E. Maui/Hāna | Kahikinui; Kipapa Rockshelter | Chapman & Kirch 1979; O'Day 2002, 2004; Kirch & O'Day 2003; Jones & Kirch 2007 |
| Maui | S. Maui/ Wailea | Southern Acreage & Lot 15 | Gosser et al. 1993 |
| Maui | W. Maui | Hawea Point | Kirch 1973b, 1982a |
| Moloka‘i | E. Moloka‘i/ Hālawa Valley | Hālawa Valley | Kirch & Kelly 1975; Kirch 1982a; Kirch & McCoy 2007 |
| Moloka‘i | N. Moloka‘i | Kalama‘ula | Athens 1985 |
| Moloka‘i | N. Moloka‘i | Kalaupapa Peninsula | Hirata & Potts 1971; Kirch et al. 2003; McCoy 2008 |
| O‘ahu | N. O‘ahu/ Waialua | Anahulu Valley rockshelters | Kirch 1979a, 1989; Kirch & Spriggs 1993 |
| O‘ahu | SE O‘ahu/ Waimanalo | Bellows Beach | Pearson et al. 1971; Cordy & Tuggle 1976; Tuggle & Spriggs 2000; Chui 2002; Dye & Pantaleo 2010 |
