# Supplementary material for: Historical Reconstruction Reveals Recovery in Hawaiian Coral Reefs
Source: PLoS One. 2011 Oct 3;6(10):e25460. doi: 10.1371/journal.pone.0025460 (PMC3184997; doi:10.1371/journal.pone.0025460)
Supplement: Table S5 — Summary of trends in faunal remains recovered from excavated middens in archaeological sites in the main Hawaiian Islands, sorted by date. Changes in ecological state (EcoState) are denoted as positive (up arrow), negative (down arrow), or not able to be determined (−) for specific time periods based on summary of major findings. Abbreviations for ecological guilds as follows: SC = small carnivores; SH = small herbivores; LH = large herbivores; LC = large carnivores. *indicates sites that have not been re-dated with modern methods; as such dates must be viewed with caution. Numbered citations are listed below the table. (DOCX) [file pone.0025460.s011.docx]

**Table S5**: Summary of trends in faunal remains recovered from excavated middens in archaeological sites in the main Hawaiian Islands, sorted by date. Changes in ecological state (EcoState) are denoted as positive (up arrow), negative (down arrow), or not able to be determined (-) for specific time periods based on summary of major findings. Abbreviations for ecological guilds as follows: SC = small carnivores; SH = small herbivores; LH = large herbivores; LC = large carnivores. *indicates sites that have not been re-dated with modern methods; as such dates must be viewed with caution. Numbered citations are listed below the table.

| **Island** | **Areas Excavated** | **Dates** (Years AD) | **EcoState** (Years) | **Summary of Results/Major Findings** | **Guilds** | **Citation** |
| --- | --- | --- | --- | --- | --- | --- |
| Kaua‘i | Makauwahi Cave | 1039-1900+ | 🡪 (~1039-1778) | Midden remains includes fish, mollusks and echinoderms; Evidence from the site indicates that human overexploitation resulted in size changes in marine mollusks in the prehistoric period | SC, SH, LC | 1 |
| O‘ahu | Bellows Beach | 1040-1800+ | 🡨 (1400+) | Midden remains include fish and mammal bone, fish scales, and mollusks; Analysis of fish bones suggests that parrotfish increased in size in late prehistory; Subsistence patterns suggest a switch in focus from inshore reef carnivores to herbivorous fish; Low midden density recovered | SH, SC | 2 |
| Moloka‘i | Hālawa Valley | 1200-1650+ | 🡪 (~1200-1400)  🡨 (~1400+) | The concentration of domesticates (pig and dog) and fish fauna recovered from controlled excavation shows an initial phase of high marine resource exploitation gradually replaced by a reliance on domestic food (pig and dog); this transition appears to have taken place “well after the initial human impact on the endemic fauna and corresponds to the advent of intensive pondfield agriculture” | SC, SH, LC | 3 |
| Kaua‘i | Hā‘ena, Ke‘e Beach | ~1230-1700+* | 🡪 (1230-1494)  🡨 (~1500-1700+) | Midden remains show: 1) a sharp decrease in size of shellfish and availability of some shellfish species during early periods (~AD 1230-1494); 2) a high percentage of fish and bird in the earlier strata shifting to a primary focus on mammal (primarily pig); and, 3) shifts in habitat utilization from easily accessed intertidal surge zone to the shallow inshore reef area; Increased dependence on taro production but less emphasized exploration of marine resources continues until the 1700-1800s - decreasing density of cultural material in this period indicates depopulation or changing settlement patterns | SC, SH | 4 |
| O‘ahu | Anahulu Valley rockshelters | 1245-1810+ | _ | Midden analysis from several excavations at this inland site reveal reliance on marine exploitation strategies, with fish, invertebrates, and marine turtle remains recovered and evidence of animal husbandry; Faunal patterns for rockshelters at this inland site suggest repeated, shorter-term use; Some sites indicate no temporal change in faunal remains, but some show higher density of mollusks in earlier strata | SC, SH, LH | 5 |
| Hawai‘i | Kalahuipua‘a rockshelter | 1279+* | _ | Midden remains include shellfish and reef fish species; A general trend was observed of an increase in frequency of parrotfish with an attendant decrease in the frequency of labrids through time; Site probably used as an intermittent fishing camp | SC, SH, LC | 6 |
| Maui | Wailea, Maui | 1280-1800+* | _ | Very little faunal material recovered but remains include shellfish; Lack of mammal bones indicate that the region was not able to support a domesticated mammal population; Lack of fish bone in midden is problematic given the artifactual evidence that inhabitants “were primarily fishers” | SC, SH | 7 |
| Hawai‘i | Koai‘e coastal village & Lapakahi upland | ~1300-1800 | 🡪 (1300-1500)  🡨 (~1500-1800+) | The coastal fishing site was probably inhabited first, possibly as early as AD 1300; shellfish comprised the largest component of the midden, along with fish, mammal (pig & dog) and a small percentage from turtle bone and Hawaiian monk seal; Initial focus on exploitation of marine sources, followed by increased agricultural development first with seasonal swidden agriculture and then permanent field systems (AD 1500+) | SC, SH, LH, LC | 8 |
| Lana‘i | Hulopo‘e | ~1300-1800+* | 🡨 (~1400+) | Midden remains include negligible quantities of marine shell, fish bone, and mammal bone; Inland occupation followed by later intensification in AD 1400 associated with agricultural activities; Analysis suggests coastal sites as temporarily inhabited until the protohistoric period | SC, SH, LH, LC | 9 |
| Hawai‘i | Kuakini Cave Sites D7-27 & D8-33 | 1300-1820* | 🡨 (~1300+) | Midden remains show that fish decrease markedly through time and mammals increase dramatically; The consumption of domestic animals appear to increase through time | SC, SH, LH | 10 |
| Moloka‘i | Kalama‘ula | 1405-1765+ | 🡨 (~1400-1600) | Very low density of midden; Subsequent occupations from AD 1400-1600 indicate a primary focus on agricultural activities, and a decreased focus on marine exploitation | SC, SH, LC | 11 |
| Kaua‘i | Nu‘alolo Kai | 1410+ | 🡪 (~1400+) | Analysis of midden remains suggests that heavy exploitation by humans resulted in declines in the abundance of mollusks (resource depression) at the site, evidenced by shifts from larger, more desirable mollusks to smaller species in the intertidal zone | SC, SH, LH, LC | 12 |
| Maui | Hawea Point | 1420-1715* | _ | Analysis of excavated materials from this area indicate that the site was used as a temporary base for marine resource exploitation; Species recovered from excavations included gastropods, urchins, and small amounts of fish bone; Fish may have been preserved for transport to permanently inhabited upland agricultural sites | SC, SH | 13 |
| Hawai‘i | Ka‘u: Pakini-Nui & Pakini-Iki, Wai‘ahukini (Ka‘u/S. Point) | 1432-1850 | _ | Midden remains decrease in birds and increased in domesticated animals with a continued reliance on marine fauna throughout the sequence; No marked temporal change in density of fish remains has been noticed in stratified sites, but a gradual decrease in the prevalence of small carnivores through time, with an increased focus on herbivores is attributed to changes in fishing strategies supported by interpretation of the portable artifacts recovered | SC, SH, LC | 14 |
| Maui | Kahikinui; Kipapa Rockshelter | 1470-1800+ | _ | Site believed to be fishing camp paired with agricultural and animal husbandry activities in the adjacent upland zone; Midden analyses indicate a high reliance on littoral invertebrates early in the record; Fish and invertebrate remains made up the largest portion of the assemblage, and a minor component of the assemblage was attributed to pig, dog and rat; Midden remains also reveal differences in patterns of reef biota exploitation based on social rank (commoner vs. elite), gender and use in ritual activities - larger carnivorous fish appear to be reserved for males of high social status | SC, SH, LH, LC | 15 |
| Hawai‘i | Kahalu‘u | 1550* | _ | Archaeological surveys suggest a paired subsistence strategy with marine exploitation along the coasts and agricultural and animal husbandry activities in the uplands (>150 m elev.); Coastal midden remains include shellfish, reef fish, bird and domesticated mammals (likely from uplands sites); Upland settlement associated with agricultural activitiy by AD 1340, settlement in environmentally marginal areas near the coast by AD 1550 | SC, SH, LC | 16 |
| Moloka‘i | Kalaupapa Peninsula | >1650 | 🡨 (~1650+) | The size of shells of intertidal limpets increased from the late prehistoric period to the historic period, which suggests release of harvesting pressure during the historic period; Midden analyses also suggest a marked reduction in the overall density of invertebrates in the younger cultural deposits, and reliance on domesticated mammals in this late prehistoric site | SC, SH, LC | 17 |

**Citations**

1 Kikuchi & Burney 1998; Burney et al. 2001; Burney & Kikuchi 2006; Burney, pers. comm., 2009

2 Pearson et al. 1971; Cordy & Tuggle 1976; Tuggle & Spriggs 2000; Chui 2002; Dye & Pantaleo 2010

3 Kirch & Kelly 1975; Kirch 1982a; Kirch & McCoy 2007

4 Griffin et al. 1977; Hammatt et al. 1978; Griffin 1984

5 Kirch 1979a, 1989; Kirch & Spriggs 1993

6 Kirch 1979b, 1982a; Hommon 1983

7 Gosser et al. 1993

8 Pearson 1969; Newman 1970;Tuggle & Griffin 1973; Rosendahl 1994

9 Tomonari-Tuggle et al. 2000

10 Schilt 1984

11 Athens 1985

12 Soehren n.d.; Gordon 1993; Morrison & Hunt 2007

13 Kirch 1973b, 1982a

14 Sinoto & Kelly 1975; Goto 1984, 1986, 1990; Dye 1992

15 Chapman & Kirch 1979; O'Day 2002, 2004; Kirch & O'Day 2003; Jones & Kirch 2007

16 Kirch 1973a

17 Hirata & Potts 1971; Kirch et al. 2003; McCoy 2008
