# Supplementary material for: Historical Reconstruction Reveals Recovery in Hawaiian Coral Reefs
Source: PLoS One. 2011 Oct 3;6(10):e25460. doi: 10.1371/journal.pone.0025460 (PMC3184997; doi:10.1371/journal.pone.0025460)
Supplement: Table S6 — Representative observations of the ecological condition of reef biota in the Main Hawaiian Islands during the prehistoric and early historic period. *exact dates for these observations are not described; estimates are derived from descriptions of authors or commentators. (DOCX) [file pone.0025460.s012.docx]

**Table S6**: Representative observations of the ecological condition of reef biota in the Main Hawaiian Islands during the prehistoric and early historic period. *exact dates for these observations are not described; estimates are derived from descriptions of authors or commentators.

| **Island(s)** | **Date** | **Observations** | **Source** |
| --- | --- | --- | --- |
| MHI | prehistoric* | Legend of Nihooleki, a chief and famous fisherman who ultimate became the paramount chief on Kaua‘i: "The wife proceeded to give away [fish], feed the pigs, give some to the loafers, sell some, and salt some, but a large number were still left over, there being so many. The people from the uplands came down with food, sugar-cane, bananas and everything else good to eat and all went home with fish, even those that came down without anything went home with their share. Those who were still in the uplands when they heard of the fish came down and returned with their share." | Fornander 1916-1920:Vol. 4:494 |
| MHI | prehistoric* | Legend of Puniakaia, who released his famous decoy parrotfish Uhumakaia, which led vast numbers of fish to shore: "The fish reached from way down deep in the sea to the surface, and they were driven clear up onto the sand. Upon seeing this the people began taking up the fish, some were salted, some were given away to the people…from the Makapuu point to the Kaoio point at Kualoa." People came from all over the district of Koolau to obtain fish, but there were still fish left to feed to the dogs and pigs. | Fornander 1916-1920:Vol 5:154-162 |
| MHI | late prehistoric* | Large quantitites of hina‘i kala [reef fish] were obtained by fishing with traps, forming "a pile as big as a house" which might contain forty thousand fish | Kamakau 1976:84-85 |
| MHI | late prehistoric* | Enough octopus might be obtained to "fill forty or fifty canoes" and were piled on the shore. They were then apportioned out to the community. | Kamakau 1976:70-71 |
| MHI | late prehistoric* | Describes bag net fishing, which could fill ten to twenty canoes with fish | Kamakau 1976:64 |
| Kaua‘i | 1778, 1779 | Observed that diet was primarily comprised of vegetables and domesticated mammals; Commented on the great numbers of fishing gears and the practice of salting fish | Cook 1842 |
| O‘ahu | 1790s | “On this trip, there were so much fish caught that a stench rose up on the shore. People went from Ewa, Waianae and Waialua [districts] to get some fish but the supply was inexhaustible. The fish kept coming to the same place for several days” | Kalakaua n.d. |
| MHI | 1794 | Saw "great numbers taken" that were "exceedingly good to eat when fresh, and being caught in abundance…"; observed fish and turtle as commonly caught | Vancouver 1967: Vol. II:168 |
| Hawai‘i | 1804 | "the coast of [Hawai‘i] abounds in fish" | Lisiansky 1814 |
| O‘ahu | 1805? | Described practices of stocking fishponds seasonally by harvesting schools of juveniles; Attributed the abundance of fish to fishponds | Schaler 1935:88 |
| O‘ahu | 1809-1810 | "Sharks are numerous in these seas" | Campbell 1825:144 |
| O‘ahu | 1815 | "In the morning, the nets were run out and set on the [reef] flat…by this method they catch 50 or 60 canoe-loads" | Corney 1965:207 |
| O‘ahu | 1817 | Recorded large catches from inshore fishing of reefs near Honolulu: "Many canoes were outside the breakers in depths of ten to fifteen feet engaged in fishing. They were using long drag nets with which they caught a great variety of fish, particularly [butterfly] fish that shimmered in the most wonderful colors" | Chamisso 1939:77 |
| Hawai‘i | 1819 | Observed "an enormous quantity of fish nets," and remarked on the types of reef fish consumed | Freycinet 1978 |
| O‘ahu, Hawai‘i | 1825 | "[Kaneohe] bay is open and exposed and full of rocks in many places above water... It is full of fish"; "The sea here, like at other parts of the islands we touched at, abounds with a variety of excellent fish of many colours." | Macrae 1922:26, 71 |
| MHI | 1826 | Described abundant sharks throughout the archipelago | Bingham 1849 |
| Hawai‘i, Kaua‘i | 1830 | "Fish were so plentiful, especially at Waialua and Wai‘anae, that pigs and dogs feasted on those that rotted. On Hawaii and Kauai there was the same abundance. The fish caught were [reef fish]...At Wailua the kahala fishing grounds were so rich a man could catch as many as twenty to forty fish at one haul." | Kamakau 1961:301 |
| Lana‘i | 1840 | Described high abundance of reef fish observed by members of the US Exploring Expedition | Wilkes 1845:117 |
| Moloka‘i, MHI | early 1800s* | Describes "abundant" fish surround the Hawaiian Islands | Nakuina 1904:11 |
| O‘ahu | 1800s* | Described "a sea swarming with fish, taken by exploding a stick of dynamite in the water, an expedient that half-filled a boat with fish" | Farrell 1928:219 |
