# Supplementary material for: Historical Reconstruction Reveals Recovery in Hawaiian Coral Reefs
Source: PLoS One. 2011 Oct 3;6(10):e25460. doi: 10.1371/journal.pone.0025460 (PMC3184997; doi:10.1371/journal.pone.0025460)
Supplement: Supporting Information S1 — Bibliography of sources. (DOCX) [file pone.0025460.s013.docx]

SUPPORTING INFORMATION S1: Bibliography of sources.

Abbott, I. A. 1978. The uses of seaweed as food in Hawaii. Economic Botany 32:409-412.

Abbott, I. A. 1984. Limu: An ethnobotanical study of some edible Hawaiian seaweeds. Pacific Tropical Botanical Garden, Lawai, Hawaii.

Adams, T. 1998. The interface between traditional and modern methods of fishery management in the Pacific Islands. Ocean and Coastal Management 40:127-142.

Adams, T., P. Dalzell, and R. Farman. 1996. Status of Pacific Island coral reef fisheries. Paper presented at the 8th International Coral Reef Symposium, Panama. [online] http://www.spc.int/coastfish/Reports/ICFMAP/statreef.htm

Aeby, G. S. 2005. Outbreak of coral disease in the Northwestern Hawaiian Islands. Coral Reefs 24:481-481.

Aeby, G. S. 2006. Baseline levels of coral disease in the Northwestern Hawaiian Islands. Atoll Research Bulletin 543:471-488.

Aeby, G. S., J. C. Kenyon, J. E. Maragos, and D. C. Potts. 2003. First record of mass coral bleaching in the Northwestern Hawaiian Islands. Coral Reefs 22:256-256.

Aeby, G. S., T. Work, T. Lewis, S. Coles, and J. Kenyon. 2008. Coral disease across the Hawaiian Archipelago. Paper presented at the 16th Annual Hawai‘i Conservation Conference, 29-31 July 2008, Honolulu, Hawaii, USA.

Agassiz, A. 1889. The coral reefs of the Hawaiian Islands. Bulletin of the Museum of Comparative Zoology at Harvard College 17:121-170.

Agassiz, A. 1903. The coral reefs of the tropical Pacific. Harvard University Museum of Comparative Zoology. University Press: John Wilson and Son, Cambridge, USA.

Aguirre, A. A., G. H. Balazs, B. Zimmerman, and T. R. Spraker. 1994. Evaluation of Hawaiian green turtles (Chelonia mydas) for potential pathogens associated with fibropapillomas. Journal of Wildlife Diseases 30:8-15.

Aguirre, A. A., J. S. Reif, and G. A. Antonelis. 1999. Hawaiian monk seal epidemiology plan: health assessment and disease status studies. NOAA technical memorandum NOAA-TM-NMFS-SWFSC 280. U.S. Dept. of Commerce, National Oceanic and Atmospheric Administration, National Marine Fisheries Service, Southwest Fisheries Science Center, La Jolla, CA, USA. 63 pgs.

Aguirre, A. A., T. R. Spraker, G. H. Balazs, and B. Zimmerman. 1998. Spirorchidiasis and fibropapillomatosis in green turtles from the Hawaiian Islands. Journal of Wildlife Diseases 34:91-98.

Alexander, M. C. 1912. The story of Hawaii. American Book Company, New York, Cincinnati.

Alexander, W. D. 1899. A brief history of the Hawaiian people. American Book Company, New York.

Alexander, W. D. 1904. Early trading in Hawaii. Papers of the Hawaiian Historical Society 11:22-24.

Alexander, W. D. 1911. The story of the trans-Pacific cable. 18th Annual Report of the Hawaiian Historical Society.

Allen, G. E. 1950. Hawaii's War Years. University of Hawai‘i Press, Honolulu.

Allen, G. M. 1942. Extinct and vanishing mammals of the Western Hemisphere. Publication of the American Committee on International Wild Life Protection. The Intelligencer Printing Co., Lancaster, PA, USA.

Allen, J. A. 1918. The Laysan seal. Natural History 18:399-400.

Allen, J. A. 1998. Mangroves as alien species: the case of Hawaii. Global Ecology and Biogeography Letters 7:61-71.

Allen, M. S. 1984. A review of archaeobotany and palaeoethnobotany in Hawaii. Hawaiian Archaeology 1:19-30.

Allen, M. S. 2002. Resolving long-term change in Polynesian marine fisheries. Asian Perspectives 41:195-213.

Allen, M. S. 2003. Human impact on Pacific nearshore marine ecosystems. Pages 317-325 in C. Sand, editor. Pacific Archaeology: Assessments and Prospects, Le Cahiers de l'Archéologie en Nouvelle-Calédonie 15. Services des Musées et du Patrimoine, Nouméa.

Allen, M. S. 2004. Bet-hedging strategies, agricultural change, and unpredictable environments: historical development of dryland agriculture in Kona, Hawaii. Journal of Anthropological Archaeology 23:196-224.

Allen, M. S. 2007. Three millennia of human and sea turtle interactions in remote Oceania. Coral Reefs 26:959-970.

Allen, M. S., E. Matisoo-Smith, and A. Horsburgh. 2001. Pacific 'Babes': issues in the origins and dispersal of Pacific pigs and the potential of mitochondrial DNA analysis. International Journal of Osteoarchaeology 11:4-13.

Amerson Jr., A. B. 1971. The natural history of French Frigate Shoals, Northwestern Hawaiian Islands. Atoll Research Bulletin 150. [online] http://www.botany.hawaii.edu/faculty/duffy/atoll.htm

Amerson Jr., A. B., R. B. Clapp, and W. O. Wirtz III. 1974. The natural history of Pearl and Hermes Reef, Northwestern Hawaiian Islands. Atoll Research Bulletin 174. [online] http://www.botany.hawaii.edu/faculty/duffy/atoll.htm

Anders, G. C. 1987. Native Hawaiian Rights in a regulated fishery: an exploratory analysis. East-West Center, Pacific Islands Development Program, Honolulu, Hawaii, USA. 19 pgs.

Anderson, A. 1991. The chronology of colonization in New Zealand. Antiquity 65:767-795.

Anderson, A. 1995. Current approaches in East Polynesian colonisation research. Journal of the Polynesian Society 104:110-132.

Anderson, A. 1996. Was Rattus exulans in New Zealand 2000 years ago? AMS radiocarbon ages from Shag River Mouth. Archaeology in Oceania 31:178-184.

Anderson, A. 1997. Uniformity and regional variation in marine fish catches from prehistoric New Zealand. Asian Perspectives 36:1-26.

Anderson, A. 2001. No meat on that beautiful shore: the prehistoric abandonment of subtropical Polynesian islands. International Journal of Osteoarchaeology 11:14-23.

Anderson, A. 2002. Faunal collapse, landscape change and settlement history in remote Oceania. World Archaeology 33:375-390.

Anderson, A. 2008. The rat and the octopus: initial human colonization and the prehistoric introduction of domestic animals to Remote Oceania. Biological Invasions 11:1503-1519.

Anderson, A., and I. W. G. Smith. 1996. The transient village in southern New Zealand. World Archaeology 27:359-371.

Anderson, A. J., and B. F. Leach. 2001. Zooarchaeology of oceanic coasts and islands - Papers from the 8th International Congress of the International Council for Archaeozoology, 23-29 August 1998, British Columbia, Canada. International Journal of Osteoarchaeology 11:1–171.

Anderson, W. G. 1944. A complete and accurate description of fishing in the islands. Freeman Lang, Honolulu.

Andrade, C. 2008. Hā‘ena: Through the Eyes of the Ancestors. University of Hawai‘i Press, Honolulu.

Anonymous. 1857. Arrival of the Manuokawai-interesting account of her exploration. The Polynesian, Honolulu. 6 June 1857, Vol 40:2-3.

Anonymous. 1859a. Chart of Midway. Hawaii State Archives.

Anonymous. 1859b. Report of a sealing and exploring voyage of Hawaiian Bark Gambia. The Polynesian. 13 August 1859, Honolulu.

Anonymous. 1867. Loss of the Bark Daniel Wood. Pacific Commercial Advertiser, 27 April 1867, Honolulu.

Anonymous. 1886a. Dunnottar Castle Relief Expedition! Hawaiian Gazette, 5 October 1886, pg. 8, Honolulu.

Anonymous. 1886b. Ocean Island, Return of the steamer Waialeale. 30 September 1886, Honolulu.

Anonymous. 1897. Hawaiian Epidemics. Pages 95-101 in T. G. Thrum, editor. Thrum's Hawaiian Almanac 1897, Honolulu.

Anonymous. 1900a. Laysan Island's Story of Blood. Pacific Commercial Advertiser, 8 Sept. 1900, Honolulu.

Anonymous. 1900b. Well publicized shark expedition fails to find sharks. Pacific Commercial Advertiser, 24 Sept 1900, p.10 (13), Honolulu.

Anonymous. 1901a. Bird life seriously diminished by raid of Japanese on Midway Island. Pacific Commercial Advertiser, 22 Aug. 1901, p.6, Honolulu.

Anonymous. 1901b. Ku'ula the Fish God of Hawaii. Page 114. Translation from Moke Manu. Hawaiian Annual for 1901. Identified on pg. 197: Thrum, T. G. 1907. Hawaiian Almanac and Annual for 1908. The Reference Book of Information and Statistics relating to the Territory of Hawaii, of Value to Merchants, Tourists and Others. Thos G. Thrum, Honolulu.

Anonymous. 1901c. PCA quotes opponents to a bill that would legalize use of dynamite in fishing. Pacific Commercial Advertiser, 27 March 1901, p.5 (4), Honolulu.

Anonymous. 1905. Bystander column reminisces about fishing with dynamite. Pacific Commercial Advertiser, 22 Jan 1905, p.4 (2), Honolulu.

Anonymous. 1908a. Fish ponds, Pearl River and Locks. Paradise of the Pacific, May 1908, p. 10, Honolulu.

Anonymous. 1908b. Dynamiting for fish practiced inshore waters of these islands for nearly 40 years. Paradise of the Pacific, Jan. 1908, p.22 c.2, Honolulu.

Anonymous. 1917. Representative Wilder wins protection the female lobster; House Bill 26 to provide a closed season for the ama‘ama, or mullet, is defeated. Honolulu Advertiser, 13 March 1917, p.7 c.7, Honolulu.

Anonymous. 1918. New reduced scale of fish prices to go into effect until the end of April. Honolulu Star-Bulletin, 25 February 1918, pg. 1 (1), Honolulu.

Anonymous. 1924. Sampan Ebisu Maru arrives from Midway with $2,000 fish cargo. Honolulu Advertiser, 23 Feb 1924, Honolulu.

Anonymous. 1925. History of Hawaii’s fisheries, by Comm. V. S. Houston. Honolulu Advertiser, 30 April 1925, Honolulu.

Anonymous. 1925. The fish question in Hawaii. Honolulu Advertiser, 26 July 1925, Honolulu.

Anonymous. 1927. Newly formed Lanikai Fishing Company. Honolulu Advertiser, 16 Jan. 1927, Honolulu.

Anonymous. 1934. Fish yields for territory. Honolulu Star-Bulletin, 13 Sept. 1934, p.8 c.4, Honolulu.

Anonymous. 1937a. Swarms of akule. Paradise of the Pacific, Aug. 1937, p.13, Honolulu.

Anonymous. 1937b. Reefs in French Frigate Shoals dead, says diver. Honolulu Star-Bulletin, 1 Oct. 1937, p.3 c.3, Honolulu.

Anonymous. 1937c. Midway, Wake Island waters said fishermen's paradise. Honolulu Advertiser, 14 Sept. 1937, p.1 c.3, Honolulu.

Anonymous. 1938. Barracuda catch seen as record. Yeiko Oshiro hooks 57 pound fighter of Mahukona, Hawaii. Honolulu Star-Bulletin, 31 Dec. 1938, p.1 c.6, Honolulu.

Anonymous. 1938. Midway Island and Japanese - 1900. Paradise of the Pacific, May 1938, p.19, Honolulu.

Anonymous. 1939. Leases of Pearl Harbor ponds studied. Honolulu Star-Bulletin, 18 Dec. 1939, p. 3 c.4, Honolulu.

Anonymous. 1940. Sportsmen complain of depletion by Japanese commercial fishing. Honolulu Advertiser, 11 Aug. 1940, p.1 c.1, Honolulu.

Anonymous. 1943a. Revival of fishing industry urged. Honolulu Advertiser, 9 July 1943, p.1 c.4, Honolulu.

Anonymous. 1943b. Molokai fishponds to be inspected. Honolulu Star-Bulletin, 14 Aug. 1943, p.8 c.4, Honolulu.

Anonymous. 1943c. Rehabilitation of Molokai ponds discussed. Honolulu Star-Bulletin, 18 Aug. 1943, p.6 c.3, Honolulu.

Anonymous. 1944. No fishing permits will be issued to persons who would only fish for fun. Honolulu Advertiser, 23 July 1944, p.10 c.2, Honolulu.

Anonymous. 1945a. Kure Island is fishing paradise of Midway navy men taking rest. Honolulu Advertiser, 13 July 1945, p.4 c.8, Honolulu.

Anonymous. 1945b. Large fish industry organized on Guam. Honolulu Star-Bulletin, 17 July 1945, p.1 c.5, Honolulu.

Anonymous. 1945c. Project to fill and reclaim Wailupe Fish Pond land on the shore of Oahu, near Diamond Head, to begin December 15. Honolulu Star-Bulletin, 13 Dec. 1945, p.8 c.1, Honolulu.

Anonymous. 1945d. Hearing on compensation for Damon Estate fisheries, condemned by the Territory in 1941, nears close. Honolulu Advertiser, 16 Dec. 1945, p.5 c.6, Honolulu.

Anonymous. 1946a. High prices and no restrictions increase fishing. Honolulu Star-Bulletin, 30 Jan. 1946, p.4 c.6, Honolulu.

Anonymous. 1946b. Urges rebuilding of Hawaii’s inshore fisheries. Honolulu Star-Bulletin, 28 June 1946, p.8 c.3, Honolulu.

Anonymous. 1946c. Conservation of fisheries. Honolulu Star-Bulletin, 13 July 1946, p.12 c.3, Honolulu.

Anonymous. 1946d. Vernon Brock compiles state of Territory of Hawaii fishery resources. Honolulu Advertiser, 10 Nov. 1946, p.18 c.1, Honolulu.

Anonymous. 1946e. Seaside Fishing Company establishes camp on Tern Island, French Frigate Shoals, flies catch to Honolulu. Honolulu Star-Bulletin, 16 Dec. 1946, p.13 c.4, Honolulu.

Anonymous. 1947a. French Frigate Shoals may be used as a public airport and fishing base for commercial fishermen. Honolulu Star-Bulletin, 1 Jan. 1947, p.4 c.2, Honolulu.

Anonymous. 1947b. Saving Hawaii's natural resources. Honolulu Star-Bulletin, 4 Jan. 1947, p.7 c.4, Honolulu.

Anonymous. 1947c. 48 more fisheries may be condemned. Honolulu Advertiser, 13 June 1947, p.3 c.7, Honolulu.

Anonymous. 1947d. Fish ponds disappearing. Honolulu Advertiser, 26 Oct. 1947, p.14 c.7, Honolulu.

Anonymous. 1949. Bait license, Kaneohe Bay. Honolulu Star-Bulletin, 16 Feb. 1949, p.24 c.4, Honolulu.

Anonymous. 1950. Recalling 1880: Molokai brig brings boat-load of o‘io to Honolulu market. Honolulu Advertiser, 23 March 1950, Ed. p. c.4, Honolulu.

Anonymous. 1950. Inshore fisheries. Honolulu Star-Bulletin, 7 June 1950, p.12 c.4, Honolulu.

Anonymous. 1953. Leeward isle fishing rules now in effect. The Honolulu Advertiser, 26 July 1953, pg. 12 (3), Honolulu.

Anonymous. 1956. Terr. Fish & Game wants to rehabilitate HI’s decreasing fish Honolulu Star-Bulletin, 4 July 1956, p.12 c.1, Honolulu.

Anonymous. 1959. Map of Pearl Harbor showing locations of fishponds and other old landmarks. Compiled from maps dating from 1873 to 1915. Bernice P. Bishop Museum, Honolulu, Hawaii, USA.

Anonymous. 1959. Hawaii's shark control program. Honolulu Advertiser, 11 Sept 1959, pg. A8 c.3, Honolulu.

Anonymous. 1961. Kauai's shark eradication program. Honolulu Star-Bulletin, 24 July 1961, p.3, c.3, Honolulu.

Anonymous. 1962. County supervisors request additional funds for shark eradication. Honolulu Star-Bulletin, 22 June 1962, p.4 c.5, Honolulu.

Anonymous. 1965. Anti-shark committee appointed. Honolulu Star-Bulletin, 10 May 1965, p.1 c.3, Honolulu.

Anonymous. 1965. City-state to join shark eradication. Honolulu Advertiser, 9 July 1965, p.1 c.4, Honolulu.

Anonymous. 1965. Shark control program shapes up. Honolulu Advertiser,13 July 1965, p.6 c.1, Honolulu.

Anonymous. 1965. Specific shark control program promised soon. Honolulu Star-Bulletin, 13 July 1965, pg. 1B, Honolulu.

Anonymous. 1965. Senator Hiram Fong report Navy to aid in shark control. Honolulu Star-Bulletin, 1 Sept 1965, p.B1 c.1, Honolulu.

Anonymous. 1969. Shark population around Oahu sharply reduced. Honolulu Star-Bulletin, 11 March 1969, p.9 c.1, Honolulu.

Anonymous. 1969. State Dept. of Land and Natural Resources to propose shark control legislation. Honolulu Advertiser, 27 Nov 1969, p.17 c.1, Honolulu.

Anonymous. 1970. Shark control bill becomes law. Honolulu Advertiser, 20 June 1970, p. 8 c.1.

Anonymous. 1970. State soon to launch a shark control fishing program. Honolulu Star-Bulletin, 13 July 1970, p.3 c.1, Honolulu.

Anonymous. 1970. State to resume Isle hunts in February; $50,000 appropriated by the State Legislature. Honolulu Star-Bulletin, 23 June 1971, p.B16 c.1, Honolulu.

Anonymous. 1971. State Bd. of Land and Natural Resources to hire Robert P. Kellog and his boat Machias fora six-month shark control fishing operation. Honolulu Advertiser, 23 Jan 1971, p.B1 c.1, Honolulu.

Anonymous. 1971. Dept. of Land and Natural Resources asks the Legislature for $200,000 to continue its shark control and research activities. Honolulu Advertiser, 4 March 1971, p.16 c.1, Honolulu.

Anonymous. 1971. State ready to shark hunt again; reports catching 120 sharks on a recently-completed circuit of bait sites around the state. Honolulu Advertiser, 2 June 1971, p.4 c.1.

Anonymous. 2009. Deep coral reef exploration reveals more ocean wonders. NWHI MNM Announcement List, PMNM-Recent News from Papahānaumokuākea Marine National Monument, Honolulu.

Anonymous. N.D. Hawaii's Sugar Industry. Hawaii Agriculture Research Center (HARC), Honolulu, HI.

Apfelbaum, S. I., J. P. Ludwig, and C. E. Ludwig. 1983. Ecological problems associated with disruption of dune vegetation dynamics by Casuarina equisetifolia L. at Sand Island, Midway Atoll. Atoll Research Bulletin 261. [online] http://www.botany.hawaii.edu/faculty/duffy/atoll.htm

Apple, R. A. 1973. Prehistoric and historic sites and structures in the Hawaiian Islands National Wildlife Refuge. U.S. National Park Service, Honolulu.

Apple, R. A., and W. K. Kikuchi. 1975. Ancient Hawaii shore zone fishponds: an evaluation of survivors for historical preservation. Office of the State Director, National Park Service, United States Dept. of the Interior, Honolulu. [online] http://www.nps.gov/history/history/online_books/hawaii/fishponds.pdf

Arago, J. 1823. Narrative of a voyage around the world in the Uranie and Physcienne corvettes, commanded by Captain Freycinet, during the years 1817, 1818, 1819, and 1820. Treuttel and Wurtz, London.

Arago, J. 1971. Narrative of a voyage around the world. N. Israel and Da Capo Press, Amsterdam and New York.

Armstrong, R. W., and J. A. Bier, editors. 1973. Atlas of Hawaii. The University Press of Hawaii, Honolulu.

Arthur, K. E., and G. H. Balazs. 2008. A comparison of immature green turtle (Chelonia mydas) diets among seven sites in the main Hawaiian Islands. Pacific Science 62:205-217.

Asoh, K., T. Yoshikawa, R. Kasaki, and E. A. Marschall. 2004. Damage to cauliflower coral by monofilament fishing lines in Hawaii. Conservation Biology 18:1645-1650.

Athens, J. S. 1985. Prehistoric investigations at an upland site on the leeward slopes of central Moloka'i. International Archaeological Research Institute, Inc., Honolulu.

Athens, J. S. 1988. Archaeological Survey and Testing for Airfield Perimeter Fence Project: Bellows Air Force Station, Oahu, Hawaii. International Archaeological Research Institute.

Athens, J. S. 1997. Hawaiian native lowland vegetation in prehistory. Pages 248-270 in P. V. Kirch, and T. L. Hunt, editors. Historical Ecology in the Pacific Islands. Yale University Press, New Haven, CT.

Athens, J. S. 2009. Rattus exulans and the catastrophic disappearance of Hawai‘i’s native lowland forest. Biological Invasions 11:1489-1501.

Athens, J. S., D. W. Blinn, M. J. Tomonari-Tuggle, J. V. Ward, I. International Archaeological Research, and E. S. Hawaii 1995. Paleoenvironmental Investigations at Uko‘a Pond, Kawailoa Ahupua‘a, O‘ahu, Hawai‘i. International Archaeological Research Institute.

Athens, J. S., H. D. Tuggle, D. J. Welch, and J. V. Ward. 2002. Avifaunal extinctions, vegetation change, and Polynesian impacts in prehistoric Hawai‘i. Archaeology in Oceania 37:57-78.

Athens, J. S., and J. V. Ward. 1994. Paleoenvironmental investigations at Kekaulike, Nu'uanu Ahupua'a, Honolulu, O‘ahu, Hawai‘i Report prepared for Archaeological Consultants of Hawaii. International Archaeological Research Institute, Inc., Honolulu, Hawaii, USA.

Atkinson, A. L. C., and W. A. Bryan. 1914. A rare seal (quotations from a letter). New York Zoological Bulletin 16:1050-1051.

Bailey, A. M. 1918. The monk seal of the Southern Pacific. Natural History 18:396-399.

Bailey, A. M. 1952. Museum pictorial: the Hawaiian monk seal. Denver Museum of Natural History, Denver, CO, USA.

Baker, J. D., and T. C. Johanos. 2004a. Abundance of the Hawaiian monk seal in the main Hawaiian Islands. Biological Conservation 116:103-110.

Baker, J. D., C. L. Littnan, and D. W. Johnston. 2006. Potential effects of sea level rise on the terrestrial habitats of endangered and endemic megafauna in the Northwestern Hawaiian Islands. Endangered Species Research 4:1-10.

Balazs, G. H. 1973. Status of marine turtles in the Hawaiian Islands. Elepaio 33:127-132.

Balazs, G. H. 1976. Green turtle migrations in the Hawaiian archipelago. Biological Conservation 9:125-140.

Balazs, G. H., and G. C. Whittow. 1979. Revised bibliography of the Hawaiian monk seal: Monachus schauinslandi Matschie 1905. Sea Grant Miscellaneous Report UNIHI-SEAGRANT-MR-79-03, University of Hawai‘i, Sea Grant College Program, Honolulu..

Balazs, G. H. 1980. Synopsis of Biological Data on the Green Turtle in the Hawaiian Islands. NOAA Technical Memorandum NMFS. National Oceanic and Atmospheric Administration, Southwest Fisheries Science Center, National Marine Fisheries Service, Honolulu.

Balazs, G. H. 1983. Sea turtles and their traditional usage in Tokelau. Atoll Research Bulletin 279:1-29.

Balazs, G. H., and M. Chaloupka. 2004. Thirty-year recovery trend in the once depleted Hawaiian green sea turtle stock. Biological Conservation 117:491-498.

Balazs, G. H., L. K. Katahira, and D. M. Ellis. 2000. Satellite tracking of hawksbill turtles nesting in the Hawaiian Islands. Pages 279-281 in F. A. Abreu-Grobois, R. Briseño, R. Márquez, and L. Sarti, editors. Proceedings of the Eighteenth International Sea Turtle Symposium (Supplement, 16th Symposium Addendum). U.S. Dept. Commerce, NOAA Technical Memorandum NMFS-SEFSC436. [online] http://www.pifsc.noaa.gov/library/pubs/Balazs_etal_ConfProc_p279_2000.pdf

Balazs, G. H., W. Puleloa, E. Medeiros, S. K. K. Murakawa, and D. M. Ellis. 1997. Growth rates and incidence of fibropapillomatosis in Hawaiian green turtles utilizing coastal foraging pastures at Palaau, Molokai. Pages 141-143. Proceedings 17th Annual Sea Turtle Symposium, 4-8 March 1997. U.S. Dept. of Commerce, NOAA Technical Memorandum NMFS-SEFSC-415. [online] http://aquacomm.fcla.edu/2227/1/nmfs-sefsc-tm415.pdf#page=153

Baldwin, P. R. 1937. Sea-fishing of Kauai. Paradise of the Pacific, July 1937, Vol. 49, No. 7, p. 13; 29-30, Honolulu.

Banish, L. D., and W. G. Gilmartin. 1992. Pathological findings in the Hawaiian monk seal. Journal of Wildlife Diseases 28:428-434.

Banks, J., Sir 1963. The Endeavour journal of Joseph Banks, 1768-1771 / edited by J. C. Beaglehole. Trustees of the Public Library of New South Wales in association with Angus and Robertson, Sydney.

Banner, A. H. 1974. Kaneohe Bay, Hawaii: urban pollution and a coral reef ecosystem. Pages 685-702. Proceedings of the Second International Symposium on Coral Reefs. Vol.2. Great Barrier Reef Committee, Brisbane, AU.

Barber, I. 2004. Sea, land and fish: spatial relationships and the archaeology of South Island Maori fishing. World Archaeology 35:434-448.

Bates, G. W. 1854. Sandwich Island notes by a Haole. Harper & Brothers, New York.

Beaglehole, J. C., editor. 1967. The Journals of Captain James Cook on his voyages of discovery. The voyage of the Resolution and Discovery 1776-1780. Part I. Cambridge University Press, Cambridge, UK.

Beamer, B. K. 2005. Huli Ka Palena. MA Thesis, Dept of Geography, University of Hawai‘i at Mānoa, Honolulu.

Beamer, B. K., and T. K. Duarte. 2006. Mapping the Hawaiian Kingdom: a colonial venture? Hawaiian Journal of Law and Politics 2:34-52.

Beamer, B. K. 2008. Na Wai Ka Mana? Native Agency and European Imperialism in the Hawaiian Kingdom. PhD Dissertation, Dept of Geography, University of Hawai‘i at Mānoa, Honolulu.

Beckley, E. M. 1883. Hawaiian fisheries and methods of fishing with an account of the fishing implements used by the natives of the Hawaiian Islands. Advertiser Steam Print, Honolulu.

Beckwith, M. 1917. Hawaiian shark aumakua. American Anthropologist 19:503-517.

Beckwith, M. 1970. Hawaiian mythology. University of Hawai‘i Press, Honolulu.

Beckwith, M. W. 1981. The Kumulipo: a Hawaiian creation chant. University of Hawai‘i Press, Honolulu, Hawaii, USA.

Beckwith, M. W., editor. 2007. Kepelino's traditions of Hawaii. Bernice P. Bishop Museum Bulletin 95. Bishop Museum Press, Honolulu.

Beckwith, M. W. 2008. The Kumulipo, translated by Queen Liliuokalani 1897. Forgotten Books.

Beechert, E. D. 1991. Honolulu: Crossroads of the Pacific. University of South Carolina Press, Columbia, SC.

Beemiller, R. M., and M. T. Wells. 1999. Gross state product by industry, 1995-97. Survey of Current Business 79:24-45.

Bell, F. T., and E. Higgens. 1939. A Plan for the Development of the Hawaiian Fisheries. U.S. Bureau of Fisheries Investigational Report 42.

Bennett, W. C. 1931. Archaeology of Kauai. Bernice P. Bishop Museum, Honolulu, HI, USA.

Bennett, C. C. 1869. Honolulu Directory and Historical Sketch of the Hawaiian or Sandwich Islands. C.C. Bennett, Publisher, Stationer and News Agent, No. 44 Fort Street, Honolulu.

Bergquist, P. R. 1967. Additions to the sponge fauna of the Hawaiian Islands. Micronesica 3:159-173.

Bingham, H. 1849. Residence of twenty-one years in the Sandwich Islands. Hezekiah Huntington, Hartford.

Bird, I. L. 1906. Hawaiian Archipelago: six months amongst palm groves, coral reefs, and volcanoes of the Sandwich Islands. Mutual Publishing, Honolulu.

Bishop, S. E. 1840. Journal book. Journal kept by Sereno Edwards Bishop in the passage from the Sandwich Island [sic] to Newport R.I. in the ship William Lee..Oct. 29, 1839-April 28, 1840.

Bishop, S. E. 1888. Why are the Hawaiians dying out? Or, Elements of disability for survival among the Hawaiian people. Read to Honolulu Social Science Association, November 1888, Honolulu.

Bishop, S. E. 1916. Reminiscences of old Hawaii. Hawaiian Gazette Company, Honolulu.

Blackman, T. M. 1941. Rarest seal. Natural History 47:138-139.

Blodgett, J. H. 2007. The impact of humans on the ecological history of the coral reefs in the Main Hawaiian Islands. Masters Thesis, Centre for Marine Studies. University of Queensland, Brisbane, QLD, AU.

Bloxam, A. 1925. Diary of Andrew Bloxam, Naturalist of the "Blonde" on her trip from England to the Hawaiian Islands, 1824-25. Bishop Museum Publication, Special Publication 10, Honolulu.

Board of Agriculture and Forestry. 1928. Report of the Board of Agriculture and Forestry to the Governor for the Fiscal Year Ended June 30, 1928. Board of Agriculture and Forestry, Territory of Hawaii, Honolulu.

Board of Fish and Game Commissioners. 1927. Annual Report for Year Ending June 30, 1927. Board of Fish and Game Commissioners, Territory of Hawaii, Honolulu.

Boehlert, G. W. 1993. Fisheries and marine resources of Hawaii and the U.S.-associated Pacific Islands: an introduction. Marine Fisheries Review 55:3-19.

Boggs, C. H., and R. Y. Ito. 1993. Hawaii's pelagic fisheries. Marine Fisheries Review 55:69-82.

Boggs, C. H., and B. S. Kikkawa. 1993. The development and decline of Hawaii's skipjack tuna fishery. Marine Fisheries Review 55:61-68.

Braden, W. E. 1976. On the probability of pre-1778 Japanese drifts to Hawaii. Hawaiian Journal of History 10:75-89. [online] http://hdl.handle.net/10125/16375

Bradley, H. W. 1939. The Hawaiian Islands and the Pacific fur trade, 1785-1813. Pacific Northwest Quarterly 30:275-299.

Bradshaw, C. J. A., L. S. Davis, M. Purvis, Q. Zhou, and G. L. Benwell. 2002. Using artificial neural networks to model the suitability of coastline for breeding by New Zealand fur seals (Arctocephalus forsteri). Ecological Modeling 148:111-131.

Brainard, R., A. Friedlander, D. Gulko, C. Hunter, R. Kelty, and J. Maragos. 2002. Status of coral reefs in the Hawaiian Archipelago in C. Wilkinson, editor. Status of Coral Reefs of the World. Australian Institute of Marine Science, Townsville, AU.

Brassey, A. A. 1881. A Voyage in the 'Sunbeam': Our Home on the Ocean for Eleven Months. Belford, Clarke & Co., Chicago.

Brigham, W. 1908. The Roosevelt fish. The Paradise of the Pacific, 21(5):17., Honolulu.

Brigham, W. T. 1909. The volcanoes of Kilauea and Mauna Loa on the island of Hawaii, their variously recorded history to the present time. Memoirs of the Bernice P. Bishop Museum v. 2, no. 4. Millwood, N.Y., Kraus Reprint, 1974, Honolulu.

Brigham, W. T. 1974. The ancient Hawaiian house. Kraus Reprint [Reprint of 1908 ed.], Millwood, N. Y.

Brock, V. 1960. The introduction of aquatic animals into Hawaiian water. International Revue Hydrobiology (Internationale Revue der gesamten Hydrobiologie) 45:463-480.

Brooke, G. M., editor. 1986. John M. Brooke's Pacific cruise and Japanese adventure, 1858-1860. University of Hawai‘i Press, Honolulu, HI, USA.

Brooks, N. C. 1859. Cruise of the Gambia. Pacific Commercial Advertiser, August 11 & 18, Honolulu.

Brooks, N. C. 1936. Middlebrook Islands discovered. Paradise of the Pacific, vol. 48, no. 10, p. 23, October 1936, Honolulu.

Broughton, W. R. 1804. A voyage of discovery to the north Pacific ocean...in the years 1795, 1796, 1797, 1798. T. Cadell & W. Davies, London.

Brower, K. 1974. With their islands around them. Holt, Rinehart, and Winston, New York.

Bryan Jr., E. H. 1938. Fish and fishing in Hawaii. Castle and Cooke, Limited, Booklets on Hawaii, No. 3, Honolulu.

Bryan Jr., E. H. 1938. Ancient Hawaiian life. Honolulu Advertiser Publishing Company, Honolulu.

Bryan Jr., E. H. 1942. American Polynesia and the Hawaiian chain. Tongg Publishing Company, Honolulu.

Bryan Jr., E. H. 1978. The Northwestern Hawaiian Islands: an annotated bibliography. U.S. Fish and Wildlife Service, Honolulu.

Bryan Jr., E. H., and K. P. Emory. 1986. The natural and cultural history of Honaunau, Kona, Hawai‘i. Departmental Report Series 86-2, Bernice P. Bishop Museum, Honolulu.

Bryan, W. A. 1906. A visit to Midway Island. Bishop Museum Occasional Papers, Vol. II. No. 4, Box 8., Honolulu.

Bryan, W. A. 1915. Natural history of Hawaii: Being an account of the Hawaiian people, the geology and geography of the islands, and the native and introduced plants and animals of the group. The Hawaiian Gazette Co., ltd., Honolulu.

Buck, P. H. 1957. Arts and crafts of Hawaii. Bernice P. Bishop Museum Special Publication 45, Bishop Museum Press, Honolulu, Hawaii.

Buck, P. H. 1959. Vikings of the Pacific. University of Chicago Press, Chicago.

Buckland, C. R. 1908. The fish and fisheries of Hawaii. The Pacific Monthly 19:196-199.

Buggeln, R. G. 1965. A preliminary list of the algal flora of the Midway Islands. Atoll Research Bulletin 109. [online] http://www.botany.hawaii.edu/faculty/duffy/atoll.htm

Burney, D. 2009. Personal communication to J. N. Kittinger. Kaua‘i, Hawai‘i.

Burney, D. A., H. F. James, L. P. Burney, S. L. Olson, W. Kikuchi, W. L. Wagner, M. Burney, D. McCloskey, D. Kikuchi, F. V. Grady, R. G. Ii, and R. Nishek. 2001. Fossil evidence for a diverse biota from Kaua‘i and its transformation since human arrival. Ecological Monographs 71:615-641.

Burney, D. A., and W. K. P. Kikuchi. 2006. A millennium of human activity at Makauwahi Cave, Māhā'ulepū, Kaua‘i. Human Ecology 34:219-247.

Burney, L. P., and D. A. Burney. 2003. Charcoal stratigraphies for Kaua‘i and the timing of human arrival. Pacific Science 57:211-226.

Butler Jr., G. D., and R. L. Usinger. 1963. Insects and other invertebrates from Laysan Island. Atoll Research Bulletin 98. [online] http://www.botany.hawaii.edu/faculty/duffy/atoll.htm

Butler, V. L. 2001. Changing fish use on Mangaia, southern Cook Islands: resource depression and the prey choice model. International Journal of Osteoarchaeology 11:88-100.

Byron, L. G. A. 1826. Voyages of H.M.S. Blonde to the Sandwich Islands...1824-1825. Murray, London.

Cachola-Abad, C. K. 1993. Evaluating the orthodox dual settlement model for the Hawaiian Islands: an analysis of artifact distribution and Hawaiian oral traditions. Pages 13-32 in M. Graves, and R. Green, editors. The Evolution and Organization of Prehistoric Society in Polynesia. New Zealand Archaeological Association Monograph No. 19.

Callcott, L. M., editor. 1827. Voyage of H.M.S. Blonde to the Sandwich Islands, in the Years 1824-1825. John Murray, Albemarle-Street, London.

Calkin, M. 1833-1842. Milo Calkin’s journal [1833-1842]. Typescript copy of original owned by great grandnephew George Boland Eckhart.

Calkin, M. 1953. The last voyage of the Independence, the story of a shipwreck and South Sea sketches, 1833-36. [Edited by Walter A. Starr]. Weiss Print Co., San Francisco, CA.

Callan, D. 1973. Some dietary preferences of a Hawaiian population: a statistical study of covariation of shell midden. Pages 115-126 in H. D. Tuggle, and P. B. Griffin, editors. Lapakahi Hawaii: archaeological studies. Asian and Pacific Archaeology Series No. 5, Social Science Research Institute, University of Hawai‘i, Honolulu, Hawaii, USA.

Callcott, L. M., editor. 1827. Voyage of H.M.S. Blonde to the Sandwich Islands, in the Years 1824-1825. John Murray, Albemarle-Street, London.

Campbell, A. 1825. A voyage round the world from 1806 to 1812. Allen & Watte, Roxbury, MA.

Cartwright, B. 1929. The Legend of Hawaii-loa. Journal of the Polynesian Society 38:150.

Cartwright, B. 1930. Rule and regulation prohibiting the taking of any species of pearl oyster. Territory of Hawaii, Board of Commissioners of Agriculture and Forestry, Honolulu.

Casserley, T. R. 1998. A Maritime History of the Northwestern Hawaiian Islands from Laysan to Kure. Marine Option Program, School of Ocean and Earth Science and Technology, University of Hawai‘i at Manoa.

Caut, S., E. Angulo, and F. Courchamp. 2008. Dietary shift of an invasive predator: rats, seabirds and sea turtles. Journal of Applied Ecology 45:428-437.

Caut, S., E. Angulo, and F. Courchamp. 2009. Avoiding surprise effects on Surprise Island: alien species control in a multitrophic level perspective. Biological Invasions 11:1689-1703.

Cesar, H. S. J., and P. J. H. van Beukering. 2004. Economic valuation of the coral reefs of Hawai‘i. Pacific Science 58:231-242.

Chaloupka, M., and G. Balazs. 2007. Using Bayesian state-space modeling to assess the recovery and harvest potential of the Hawaiian green sea turtle stock. Ecological Modeling 205:93-109.

Chaloupka, M., G. H. Balazs, and T. M. Work. 2009. Rise and fall over 26 years of a marine epizootic in Hawaiian green sea turtles. Journal of Wildlife Diseases 45:1138-1142.

Chamisso, A. v. 1939. Chamisso in Hawaii. (translator C.K. Houston). Annual Report of the Hawaiian Historical Society for the year 1939 48:52-82.

Chapin, H. G. 1994a. The First Hotels at Waikiki. Hawaiian Historical Society, Honolulu, HI, USA.

Chapin, H. G. 1994b. The Royal Hawaiian Hotel. Hawaiian Historical Society, Honolulu, HI, USA.

Chapin, M. H., K. R. Wood, S. P. Perlman, and M. Maunder. 2004. A review of the conservation status of the endemic Pritchardia palms of Hawaii. Oryx 38:273-281.

Chapman, M. D. 1985. Environmental influences on the development of traditional conservation in the South Pacific region. Environmental Conservation 12:217-230.

Chapman, M. D. 1987. Women's fishing in Oceania. Human Ecology 15:267-288.

Chapman, P. S., and P. V. Kirch 1979. Archaeological Excavations at Seven Sites, Southeast Maui, Hawaiian Islands. Dept. of Anthropology, Bernice P. Bishop Museum, Honolulu, Hawaii.

Chave, K. E., and S. V. Smith. 1973. Atlas of Kaneohe Bay: a reef ecosystem under stress. University of Hawai‘i, Honolulu.

Chiu, M. 2002. Fish remains from site 4853-1 at Bellows Beach, Waimanalo, Oíahu, Hawaiian Islands. New Zealand Journal of Archaeology 24:61-76.

Chun, M. N. 1986. A preliminary cultural study of primary sources concerning the islands of Nihoa, Necker, and the Leeward (Northwest) Chain. Western Pacific Regional Fishery Management Council, Honolulu.

Chun, M. N., editor. 1994. Must we wait in despair: the 1867 report of the ‘Ahahui La‘au Lapa‘au of Wailuku, Maui on Native Hawaiian health. First People’s Productions, Honolulu.

Cinner, J. E., and S. Aswani. 2007. Integrating customary management into marine conservation. Biological Conservation 140:201-216.

Clapp, R. B. 1972. The natural history of Gardner Pinnacles, Northwestern Hawaiian Islands. Atoll Research Bulletin 163. [online] http://www.botany.hawaii.edu/faculty/duffy/atoll.htm

Clapp, R. B., and E. Kridler. 1977. The natural history of Necker Island, Northwestern Hawaiian Islands. Atoll Research Bulletin 206. [online] http://www.botany.hawaii.edu/faculty/duffy/atoll.htm

Clapp, R. B., E. Kridler, and R. T. Fleet. 1977. The natural history of Nihoa Island, Northwestern Hawaiian Islands. Atoll Research Bulletin 207. [online] http://www.botany.hawaii.edu/faculty/duffy/atoll.htm

Clapp, R. B., M. D. F. Udvardy, and A. K. Kepler. 1996. An Annotated Bibliography of Laysan Island, Northwestern Hawaiian Islands. Atoll Research Bulletin 434. [online] http://www.pwrc.usgs.gov/infobase/laysan4.pdf

Clapp, R. B., and W. O. Wirtz III. 1975. The natural history of Lisianski Island, Northwestern Hawaiian Islands. Atoll Research Bulletin 186. [online] http://www.botany.hawaii.edu/faculty/duffy/atoll.htm

Clark, A. H. 1908. Descriptions of new species of crinoids, chiefly from the collections made by the U.S. Fisheries steamer "Albatross" at the Hawaiian Islands in 1902. Proceedings of the United States Natural Museum 34:209-244.

Clark, A. H. 1949. Ophiuroidea of the Hawaiian Islands. Bernice P. Bishop Museum Bulletin 195:1-133.

Clark, G. R. 1997. Maori subsistence change: zooarchaeological evidence from the prehistoric dog of New Zealand. Asian Perspectives 36:200–219.

Clark, J. T., and J. Terrell. 1978. Archaeology in Oceania. Annual Review of Anthropology 7:293-319.

Clay, H. F. 1961. Narrative report of botanical fieldwork on Kure Island, 3 October 1959 to 9 October 1959. Atoll Research Bulletin 78: [online] http://www.botany.hawaii.edu/faculty/duffy/atoll.htm

Cleghorn, P. L. 1988. The settlement and abandonment of two Hawaiian outposts: Nihoa and Necker Islands. Bernice P. Bishop Museum, Occasional Papers 28:35-49.

Clifford, D. M. 1991. Loko I'a O Hawai‘i: Ancient Hawaiian Fishponds and Their Changing Role in Society. B.A. thesis, Princeton University.

Cobb, J. N. 1902. Commercial fisheries of the Hawaiian Islands. Extracted from the U. S. Fish Commission Report for 1901. Pages 353-499, Plates 21-27. Washington, Government Printing Office.

Cobb, J. N. 1902. Queer legends of Hawaiian fishermen. New York Times, New York, 22 June 1902.

Cobb, J. N. 1905a. The commercial fisheries. Pages 715-765 in D. S. Jordan, and B. W. Evermann, editors. Bulletin of the United States Fish Commission, Vol. XXIII for 1903. The aquatic resources of the Hawaiian Islands. U. S. Fish Commission, Washington, Government Printing Office.

Cobb, J. N. 1905b. The Commercial Fisheries of the Hawaiian Islands in 1903. Appendix to the report of the commissioner of fisheries to the secretary of commerce and labor for the year ending June 30, 1904. Pages 433-512. Department of Commerce and Labor, Bureau of Fisheries, Washington, DC, Government Printing Office.

Coffman, M. and K. Kim. 2009. The economic impacts of banning commercial bottomfish fishing in the Northwestern Hawaiian Islands. Ocean & Coastal Management 52:166-172.

Coles, S. L., R. C. DeFelice, L. G. Eldredge, and J. T. Carlton. 1999. Historical and recent introductions of non-indigenous marine species into Pearl Harbor, Oahu, Hawaiian Islands. Marine Biology 135:147-158.

Collins, S. 1984. Analysis of vertebrate faunal remains. Pages 333-353 in R. Schilt, editor. Subsistence and conflict in Kona, Hawaii: An archaeological study of the Kuakini Highway realignment corridor. Hawaii Historic Preservation Report 84-1. Department of Anthropology, Bernice P. Bishop Museum, Honolulu.

Collins, S. and F. Watanabe. 1983. Analysis of faunal remains. Pages 371-383 in J. T. Clark and P. V. Kirch, editors. Archaeological Investigations of the Mudlane-Waimea-Kawaihae Road Corridor, Island of Hawaii: An Interdisciplinary Study of an Environmental Transect. Departmental Report Series 83-1. Bishop Museum Press, Honolulu.

Colnett, J., and R. M. Galois (editor) 2004. A voyage to the northwest side of America: The journals of James Colnett, 1786-89. UBC Press, Vancouver.

Colnett, J. 1959. The journal of Captain James Colnett aboard The Prince of Wales and Princess Royal from 16 Oct. 1786 to 7 Nov. 1788. Manuscript in Hawaiian Collection, Hamilton Library, University of Hawaii at Manoa, Honolulu.

Cook, J. 1842. The voyages of Captain James Cook round the world. William Smith, 113, Fleet Street, London.

Cook, J. 1999. The journals of Captain James Cook on his voyages of discovery (edited by J.C. Beaglehole). Boydell Press and Hordern House, Sydney, AU, Woodbridge, UK and Rochester, NY.

Cook, J., and J. King. 1784. A voyage to the Pacific Ocean... for making discoveries in the Northern Hemisphere ... in the years 1776, 1777, 1778, 1779 and 1780. W. and A. Strahan, London.

Cook, J. M. 1905. Midway. Pacific Commercial Advertiser, Feb. 11 1905, Honolulu.

Cooper G, and Daws G (1990). Land and power in Hawai‘i. University of Hawai‘i Press, Honolulu.

Cordy, R. 1974. Cultural adaptation and evolution in Hawaii: a suggested new sequence. Journal of the Polynesian Society 83:180-191.

Cordy, R., and M. W. Kaschko. 1980. Prehistoric archaeology in the Hawaiian Islands: Land units associated with social groups. Journal of Field Archaeology 7:403-416.

Cordy, R. H. 1981. A study of prehistoric social change: the development of complex societies in the Hawaiian Islands. Academic Press, New York.

Cordy, R. H. 2007. Reconstructing Hawaiian population at European contact. Pages 108-128 in P. V. Kirch, and J.-L. Rallu, editors. The growth and collapse of Pacific Island societies. University of Hawai‘i Press, Honolulu.

Cordy, R. H., and H. D. Tuggle. 1976. Bellows, Oahu, Hawaiian Islands: new work and new interpretation. Archaeology and Physical Anthropology in Oceania 11:207-235.

Corney, P. 1965. Early voyages in the North Pacific, 1813-1818. Ye Galleon Press, Fairfield, Wash.

Corney, P., and W. D. Alexander 1896. Voyages in the Northern Pacific. Thos G. Thrum, Honolulu.

Cornish, A. 2004a. Epinephelus quernus. 2004 IUCN Red List of Threatened Species. IUCN 2004.

Cornish, A. 2004b. Stereolepis gigas. IUCN 2009. IUCN Red List of Threatened Species. Version 2009.1.

Costa-Pierce, B. A. 1987. Aquaculture in ancient Hawaii. BioScience 37:320-331.

Costanza, R., L. Graumlich, W. Steffen, C. Crumley, J. Dearing, K. Hibbard, R. Leemans, C. Redman, and D. Schimel. 2007. Sustainability or collapse: What can we learn from integrating the history of humans and the rest of nature? Ambio 36:522-527.

Coulter, J. W. 1931. Population and utilization of land and sea in Hawaii, 1853. Bernice P. Bishop Museum Press.

Coulter, J. W. 1964. Great Britain in Hawaii: The Captain Cook Monument. The Geographical Journal 130.

Couthouy, J. P. 1842. Remarks upon coral formations in the Pacific. Boston Journal of Natural History IV:142 (also pp. 166, 137).

Creighton, T. H. 1978. The lands of Hawaii: their use and misuse. University Press of Hawaii, Honolulu.

Crossley, R. 1930. Fishing industry is important to Hawaii. Honolulu Star-Bulletin, 21 June 1930, p.2 c.3, Honolulu.

Cuddihy, L. W., and C. P. Stone. 1990. Alteration of native Hawaiian vegetation. University of Hawai‘i Cooperative National Park Resources Studies Unit, Honolulu, HI.

Dalzell, P. 1998. The role of archaeological and cultural-historical records in long-range coastal fisheries resources management strategies and policies in the Pacific Islands. Ocean and Coastal Management 40:237-252.

Dalzell, P. 2000. Fishing, Turtles and the Law: Recent Events in the Hawaii-based Longline Industry. SPC Fisheries Information Newsletter 93:1. [online] http://www.spc.int/coastfish/News/Fish_News/93/Paul_Dalzell.htm

Dalzell, P. 2001. Fishing, Turtles and the Law: Recent Events in the Hawaii-Based Longline Fishery. SPC Fisheries Information Newsletter 98:34. [online] http://www.spc.int/coastfish/News/Fish_News/98/Fish_News_98.pdf

Dalzell, P., J. H. Adams, and N. V. C. Polunin. 1996. Coastal fisheries in the Pacific Islands. Oceanography and Marine Biology: An Annual Review 34:395-531.

DAR. 2009. Hawaii Annual Reported Landings (Pounds) Table (1948-2008). State of Hawaii, Department of Land and Natural Resources, Division of Aquatic Resources (DAR), Honolulu, Hawaii, USA.

Davidson, J. M., K. Fraser, B. F. Leach, and Y. H. Sinoto. 1999. Prehistoric fishing at Hane, Ua Huka, Marquesas Islands, French Polynesia. New Zealand Journal of Archaeology 21:5-28.

Davidson, J. M., B. F. Leach, and C. Sand. 2002. Three thousand years of fishing in New Caledonia and the Loyalty Islands. Pages 153-164 in S. Bedford, C. Sand, and D. Burley, editors. Fifty Years in the Field - Essays in honour and celebration of Richard Shutler Jr's archaeological career. New Zealand Archaeological Association Monograph 25, Auckland, NZ.

Davis, B. D. 1990. Human settlement in pristine insular environments: a Hawaiian case study from Barbers Point, Southwestern Oahu. PhD Dissertation, Department of Anthropology, University of Hawai‘i, Honolulu.

Davis, B. D. 1991. In Memory of Pearl Harbor: The Losses Gone Unsung. Environment Hawai‘i.

Daws, G. 1968. Shoal of time: A history of the Hawaiian Islands. University of Hawai‘i Press, Honolulu.

De Laubenfels, M. W. 1950. The sponges of Kaneohe Bay, Oahu. Pacific Science 4:3-36.

De Laubenfels, M. W. 1951. The sponges of the island of Hawaii. Pacific Science 5:256-271.

De Laubenfels, M. W. 1957. New sponges and records of Hawaiian sponges. Pacific Science 11:236-251.

De Leo, G. A., and S. Levin. 1997. The multifaceted aspects of ecosystem integrity. Conservation Ecology 1:3. [online] http://www.consecol.org/vol1/iss1/art3/

de Varigny, C. V. C., and A. L. Korn 1981. Fourteen years in the Sandwich Islands, 1855-1868. University Press of Hawaii, Hawaiian Historical Society, c1981, Honolulu.

Deering, M. C. C. 1899. Hawaii Nei. Godfrey A. S. Wieners, New York.

Denham, T., F. J. Elbe, B. Winsborough, and J. V. Ward. 1999. Paleoenvironmental and archaeological investigations at 'Ohi'apilo Pond, leeward cost of Moloka‘i. Hawaiian Archaeology 7:35-60.

Derrickson, S. A. K., M. P. Robotham, S. G. Olive, and C. I. Evensen. 2002. Watershed management and policy in Hawaii: Coming full circle. Journal of the American Water Resources Association 38:563.

Desha, F. 2009. Cover-hundred poundahs never left! Hana hou! Hawaii Fishing News, March 2009, p. 3-4, Honolulu, HI.

Devaney, D. M., M. Kelly, P. J. Lee, and L. S. Motteler 1982. Kane'ohe, a history of change. The Bess Press, Honolulu.

Diamond, J. M. 1986. The environmentalist myth. Nature 324:19-20.

Diamond, J. M. 2005. Collapse: How societies choose to fail or succeed. Viking, New York.

Dill, H. R., and W. A. Bryan. 1912. Report of an expedition to Laysan Island in 1911 under the joint auspices of the United States Department of agriculture and University of Iowa. Government Printing Office, U.S. Dept. of agriculture. Biological survey. Bulletin no. 42, Washington, DC.

DiNardo, G. T., W. R. Haight, and J. A. Wetherall. 1998. Status of lobster stocks in the Northwestern Hawaiian Islands, 1995-97, and outlook for 1998. Admin. Rep. Page 35. H-98-05. Southwest Fisheries Science Center, National Marine Fisheries Service, Honolulu.

DiNardo, G. T., and R. Marshall. 2001. Status of lobster stocks in the Northwestern Hawaiian Islands, 1998-2000. Southwest Fisheries Science Center Administrative Report H-01-04. National Marine Fisheries Service, Honolulu.

DiNardo, G. T., and F. A. Parrish, editors. 2006. Northwestern Hawaiian Islands third scientific symposium, November 2-4, 2004. The Atoll Research Bulletin, No. 543, Washington, D.C.

Division of Fish and Game. 1927-1936. Annual and Biennial Reports. Territory of Hawaii, Board of Commissioners of Agriculture and Forestry, Honolulu.

Dixon, B., A. Carpenter, F. Eble, C. Mitchell, and M. Major. 1995. Community growth and heiau construction: possible evidence of political hegemony at the site of Kaunolu, Lana‘i, Hawai‘i. Asian Perspectives 34:229-255.

Dixon, B., P. J. Conte, V. Nagahara, and W. K. Hodgins. 1999. Risk minimization and the traditional ahupua‘a in Kahikinui, Island of Maui, Hawai‘i. Asian Perspectives 38:229-255.

Dollar, S., and R. W. Grigg. 2004. Anthropogenic and natural stresses on selected coral reefs in Hawai‘i: a multidecade synthesis of impact and recovery. Pacific Science 58:281-304.

Dollar, S. J. 1982. Wave stress and coral community structure in Hawaii. Coral Reefs 1:71-81.

Dollar, S. J., and G. W. Tribble. 1993. Recurrent storm disturbance and recovery: a long-term study of coral communities in Hawaii. Coral Reefs 12:223-233.

Doty, M. S., and B. C. Stone. 1966. Two new species of Halophila (Hydrocharitaceae). Brittonia 18:303-306.

Drake, D. R., and T. L. Hunt. 2008. Invasive rodents on islands: integrating historical and contemporary ecology. Biological Invasions 11:1483-1487.

Druett, J., editor. 1992. She was a sister sailor: the whaling journals of Mary Brewster, 1845-1851. Mystic Seaport Museum, Inc., Mystic, CT, USA.

Dunmore, J., editor. 1994. The Journal of Jean-Francois de Galaup de la Perouse. The Hakluyt Society, London.

Dwight, E. W. 1990. Memoir of Henry Obookiah: a native of the Sandwich Islands. Woman’s Board of Missions for the Pacific Islands, Honolulu, HI.

Dye, T. 1992. The south point radiocarbon dates thirty years later. New Zealand Journal of Archaeology 14:89-97.

Dye, T. 1994a. Apparent ages of marine shells: implications for archaeological dating in Hawai‘i. Radiocarbon 36:51-57.

Dye, T. 1994b. Population trends in Hawai‘i before 1778. The Hawaiian Journal of History 28:1-20.

Dye, T., and E. A. Gordon. 1994. Perspectives: comment on Gordon, "Screen size and differential faunal recovery: a Hawaiian example". Journal of Field Archaeology 21:391-394.

Dye, T., and D. W. Steadman. 1990. Polynesian ancestors and their animal world. American Scientist 78:207-215.

Dye, T. S., and T. R. Graham. 2004. Review of archaeological and historical data concerning reef fishing in Hawaii and American Samoa. T.S. Dye & Colleagues, Archaeologists, Inc., Honolulu.

Dye, T. S., and E. Komori. 1992. A pre-censal population of Hawaii. New Zealand Journal of Archaeology 14:113-128.

Dye, T. S. and J. Pantaleo. 2010. Age of the O18 site, Hawai‘i. Archaeology in Oceania 45:113-119.

Earle, T. K. 1973. Control hierarchies in the traditional irrigation economy of Halelea district, Kauai, Hawaii. PhD Dissertation, University of Michigan.

Earle, T. K. 1978. Economic and social organization of a complex chiefdom: the Halelea District, Kaua'i, Hawaii. Anthropological Paper, Museum of Anthropology, University of Michigan. No. 63, Ann Arbor, Michigan, USA.

Earle, T. K. 1987. Chiefdoms in Archaeological and Ethnohistorical Perspective. Annual Review of Anthropology 16:279-308.

Edmondson, C. H. 1946. Reef and shore fauna of Hawaii. Bernice P. Bishop Museum, Honolulu, HI.

Edmondson, C. H., and W. M. Ingram. 1939. Fouling organisms in Hawaii. Occasional Papers of Bernice P. Bishop Museum 14:251-300.

Edmondson, C. H., and I. H. Wilson. 1940. The shellfish resources of Hawaii. Pages 241-243. Sixth Pacific Science Congress. University of California Press, Berkeley, CA.

Ellis, W. 1825. Journal of a Tour Around Hawaii, the Largest of the Sandwich Island. Boston: Crocker & Brewster, No. 50, Cornhill and New York: John P. Haven, 182, Broadway.

Ellis, W. 1826. Narrative of a Tour Through Hawaii, or Owhyhee; with Remarks on the History, Traditions, Manners, Customs, and Language of the Inhabitants of the Sandwich Island. For the author, by H. Fiser, son, and P. Jackson.

Ellis, W. 1836. Polynesian researchers, during a residence of nearly eight years in the Society and Sandwich Islands. Fisher, Son, & Jackson, London.

Ellis, W. 1979. Journal of William Ellis: narrative of a tour of Hawaii, or Owhyhee : with remarks on the history, traditions, manners, customs, and language of the inhabitants of the Sandwich Islands. Charles E. Tuttle Company, Rutland, VT.

Elschner, C. 1915. The leeward islands of the Hawaiian Group. Honolulu Advertiser, Honolulu.

Else, I. R. N. 2004. The breakdown of the kapu system and its effect on Native Hawaiian health and diet. Hulili: Multidisplinary Research on Hawaiian Well-Being 1:241-255.

Ely, C. A., and R. B. Clapp. 1973. The natural history of Laysan Island, Northwestern Hawaiian Islands. Atoll Research Bulletin 171. [online] http://www.botany.hawaii.edu/faculty/duffy/atoll.htm

Emory, K. P. 1928. Archaeology of Nihoa and Necker islands. Bishop Museum, Honolulu, HI.

Emory, K. P. 1940. Hawaii, Kailua, Chart of fishing grounds off Kailua: data furnished by father of Thomas K. Maunupau, 1933. Manuscript Collection. Bernice P. Bishop Museum, Honolulu, Hawaii, USA.

Emory, K. P. 1959. Origin of the Hawaiians. The Journal of the Polynesian Society 68:29-35.

Emory, K. P., W. J. Bonk, and Y. H. Sinoto 1959. Fishhooks. Bishop Museum Press, Bernice P. Bishop Museum Special Publication 47, Honolulu.

Emory, K. P., and Y. H. Sinoto. 1961. Hawaiian archaeology: Oahu excavations. Bernice P. Bishop Museum Bulletin 53, Honolulu.

Emory, K. P., and Y. H. Sinoto. 1969. Age of sites in the South Point area, Ka'u, Hawaii. Pacific Anthropological Records 8, Department of Anthropology, Bernice P. Bishop Museum, Honolulu, Hawaii, USA.

Englund, R. A. 2002. The loss of native biodiversity and continuing nonindigenous species introductions in freshwater, estuarine, and wetland communitites of Pearl Harbor, Oahu, Hawaiian Islands. Estuaries 25:418-430.

Erlandson, J. M., M. H. Graham, B. J. Bourque, D. Corbett, J. A. Estes, and R. S. Steneck. 2007. The kelp highway hypothesis: marine ecology, the coastal migration theory, and the peopling of the Americas. The Journal of Island and Coastal Archaeology 2:161-174.

Erlandson, J. M., and T. C. Rick. 2010. Archaeology meets marine ecology: the antiquity of maritime cultures and human impacts on marine fisheries and ecosystems. Annual Review of Marine Science 2:231-251.

Esh, K. S. 2005. Human impacts on Pacific seabirds: an analysis of resource use at Nu‘alolo Kai, Kaua‘i. Masters Thesis, Department of Anthropology, University of Hawai‘i at Mānoa, Honolulu, Hawaii, USA.

Etnier, M. A. 2007. Defining and identifying sustainable harvests of resources: Archaeological examples of pinniped harvests in the eastern North Pacific. Journal for Nature Conservation 15:196-207.

Eveleth, E. 1829. History of the Sandwich Islands with an account of the American Mission established in 1820. American Sunday School Union, Philadelphia.

Evermann, B. W., and D. S. Jordan. 1973. The shore fishes of Hawaii: these fishes are found throughout the Pacific Ocean. First printed in Washington in 1905 as ’Part I: The shore fishes’ of a report entitled ’The aquatic resources of the Hawaiian Islands,’ Bulletin of the United States Fish Commission for 1903, Vol. 23. C. E. Tuttle Company, Rutland, VT.

Executive Order 199A. Placing Midway Atoll under control of the U.S. Navy. 1903, Washington, DC.

Executive Order 1019. Establishing the Hawaiian Islands Reservation. February 3, 1909, Washington, DC.

Executive Order 13022. Administration of the Midway Islands. U. S. Fish and Wildlife Service. 61 Fed. Reg. 56875-56876, November 4, 1996, Washington, DC.

Executive Order 13089. Coral reef protection. 63 Fed. Reg. 32701-32703, June 11, 1998, Washington, DC.

Executive Order 13158. Marine protected areas. 65 Fed. Reg. 34909-34911, May 31, 2000, Washington, DC.

Executive Order 13178. Northwestern Hawaiian Islands coral reef ecosystem reserve. 65 Fed. Reg. 76903-76910, December 4, 2000, Washington, DC.

Executive Order 13196. Final Northwestern Hawaiian Islands coral reef ecosystem reserve. 66 Fed. Reg. 7395-7397, January 18, 2001, Washington, DC.

Farrell, A., editor. 1928. John Cameron's odyssey. The MacMillan Co., New York.

Field, H. G. 1917. Game fishing in Hawaiian waters. Pages 87-93 in T. G. Thrum, editor. Hawaiian Annual. Thrum, Honolulu.

Fisher, W. K. 1903. Birds of Laysan and the Leeward Islands, Hawaiian group. Government Printing Office, Washington.

Fletcher, C. 2009. Personal communication on beaches in Hawaii 2000 years ago, to J. N. Kittinger. Honolulu, HI.

Fornander, A., and J. F. G. Stokes 1880. An account of the Polynesian race: its origins and migrations, and the ancient history of the Hawaiian people to the times of Kamehameha I. Vols I-III. Trübner & Co., Ludgate Hill, London.

Fornander, A. 1916-1920. Fornander Collection of Hawaiian Antiquities and Folk-Lore. Vol. 4-6. Memoirs of the Bernice P. Bishop Museum. Bernice P. Bishop Museum Press, Honolulu.

Franchère, G. 2007. Narrative of a voyage to the northwest coast of America in the years 1811, 1812, 1813, and 1814, or, The first American settlement on the Pacific. IndyPublish.com, Boston.

Fraser, K. L. 2001. Variation in tuna fish catches in Pacific prehistory. International Journal of Osteoarchaeology 11:127-135.

Frazier, F. N. 1989. The "Battle of Kalalau," as Reported in the Newspaper Kuokoa. The Hawaiian Journal of History 23:108-118.

Frazier, J. 2002. Marine turtles of the past: a vision for the future? Pages 103-116 in R. C. G. M. Lauwerier, and I. Plug, editors. 9th ICAZ Conference, The Future from the Past, Durham. [online] http://www.seaturtle.org/PDF/Frazier_2002_ICAZ.pdf

Freycinet, L. C. D. d. 1978. Hawaii in 1819: a narrative account by Louis Claude Desaulses de Freycinet (Ella L. Wiswell, translater; notes and comments by Marion Kelly). Pacific Anthropological Records No. 26, Department of Anthropology, B. P. Bishop Museum, Honolulu.

Friedlander, A., G. Aeby, S. Balwani, B. Bowen, R. Brainard, A. Clark, J. Kenyon, J. Maragos, C. Meyer, P. Vroom, and J. Zamzow. 2008a. The state of coral reef ecosystems of the Northwestern Hawaiian Islands. Pages 263-306 in J. E. Waddell, and A. M. Clarke, editors. The State of Coral Reef Ecosystems of the United States and Pacific Freely Associated States: 2008. NOAA Technical Memorandum NOS NCCOS 73. NOAA/NCCOS Center for Coastal Monitoring and Assessment’s Biogeography Team, Silver Spring, MD.

Friedlander, A., G. Aeby, R. Brainard, E. Brown, K. Chaston, A. Clark, P. McGowan, T. Montgomery, W. Walsh, I. Williams, and W. Wiltse. 2008b. The state of coral reef ecosystems of the Main Hawaiian Islands. Pages 219-261 in J. E. Waddell, and A. M. Clarke, editors. The State of Coral Reef Ecosystems of the United States and Pacific Freely Associated States: 2008. NOAA Technical Memorandum NOS NCCOS 73. NOAA/NCCOS Center for Coastal Monitoring and Assessment’s Biogeography Team, Silver Spring, MD.

Friedlander, A., G. Aeby, R. Brainard, E. K. Brown, A. Clark, S. Coles, E. DeMartini, S. J. Dollar, S. Godwin, C. Hunter, P. L. Jokiel, J. Kenyon, R. Kosaki, J. Maragos, P. Vroom, B. Walsh, I. Williams, and W. Wiltse. 2004. Status of coral reefs in the Hawaiian Archipelago. Pages 411-430 in C. Wilkinson, editor. Status of Coral Reefs of the World: 2004.

Friedlander, A. M., editor. 2004. Status of Hawaii's coastal fisheries in the new millennium: Proceedings of the 2001 fisheries symposium. The American Fisheries Society, Hawaii Chapter, Honolulu, Hawaii.

Friedlander, A. M., and E. E. DeMartini. 2002. Contrasts in density, size, and biomass of reef fishes between the northwestern and the main Hawaiian Islands: the effects of fishing down apex predators. Marine Ecology Progress Series 230:253-264.

Friedlander, A. M., and J. D. Parrish. 1997. Fisheries harvest and standing stock in a Hawaiian Bay. Fisheries Research 32:33-50.

Furneaux, C. 1922. Hawaiian canoe and fish traps, CA 1890-1900. Bernice P. Bishop Museum, Honolulu, Hawaii, USA.

Gaffney, R. 2000. Tourism and jaws. Shark Conference, Honolulu, HI, 21-24 Feb 2000. [online] http://www.pacfish.org/sharkcon/documents/gaffneyr.html

Gagne, W. C. 1988. Conservation priorities in Hawaiian natural systems: Increased public awareness and conservation action are required. BioScience 38:264-271.

Galtsoff, P. S. 1930. Preliminary report of the U.S.S. Whippoorwill to Pearl & Hermes reef. To George I. Brown, President, Board of Commissioners of Agricultur and Forestry, 16 Sept 1930, Hawaii State Archives, Honolulu.

Galtsoff, P. S. 1933. Pearl and Hermes Reef, Hawaii hydrographical and biological observations. Bernice P. Bishop Museum Bulletin 107:49.

Glazier, E. W. 2007. Hawaiian fishermen. G. Spindler, and J. Stockard, editors. Case Studies in Cultural Anthropology. 145 pgs. Thomson/Wadsworth.

Godwin, S., S. R. Ku‘ulei, and P. L. Jokiel. 2006. Reducing potential impact of invasive marine species in the Northwestern Hawaiian Islands Marine National Monument. Northwest Hawaiian Islands Marine National Monument Administration, Honolulu, HI.

Gollop, J. H., and E. W. Pon. 1992. Ciguatera: a review. Hawaii Medical Journal 51:91-99.

Gosser, D. C., S. D. Clark, and B. Dixon. 1993. Na Lawai‘i O ‘Ao‘ao Kona O Ka Moku: Excavations at the Southern Acreage and Lot 15, Wailea, Maui. Public Archaeology Section, Applied Research Group, Bishop Museum, Honolulu.

Gordon, E. A. 1993. Screen size and differential faunal recovery: a Hawaiian example. Journal of Field Archaeology 20:453-460.

Goto, A. 1984. Marine exploitation at South Point, Hawaii Island: An aspect of adaptive diversity in Hawaiian prehistory. Hawaiian Archaeology 1:44-63.

Goto, A. 1986. Prehistoric ecology and economy of fishing in Hawaii: an ethnoarchaeological approach. PhD Dissertation, Department of Anthropology, University of Hawai‘i, Honolulu.

Goto, A. 1990. Prehistoric Hawaiian fishing lore: An integrated approach. Man and Culture in Oceania 6:1-34.

Gowans, A. 1993. Fruitful fields: American missionary churches in Hawaii. Department of Land & Natural Resources, State Historic Preservation Division, Honolulu, HI.

Graves, M. W., and D. J. Addison. 1995. The Polynesian settlement of the Hawaiian Archipelago: integrating models and methods in archaeological interpretation. World Archaeology 26:380-399.

Graves, M. W., and R. C. Green, editors. 1993. The evolution and organisation of prehistoric society in Polynesia. New Zealand Archaeological Association Monograph 19, Auckland, N.Z.

Graves, M. W., and M. Sweeney. 1993. Ritual behaviour and ceremonial structures in Eastern Polynesia: Changing perspectives on archaeological variability. Pages 106–125 in M. W. Graves, and R. C. Green, editors. The Evolution and Organisation of Prehistoric Society in Polynesia. New Zealand Archaeological Association Monograph 19, Auckland, N.Z.

Grayson, D. K. 1981. A critical view of the use of archaeological vertebrates in paleoenvironmental reconstruction. Journal of Ethnobiology 1:28-38.

Grayson, D. K. 1984. Quantitative zooarchaeology: topics in the analysis of archaeological faunas. Academic Press, New York.

Grayson, D. K. 2001. The archaeological record of human impacts on animal populations. Journal of World Prehistory 15:1-68.

Green, L. S., and M. Beckwith. 1928. Hawaiian household customs. American Anthropologist 30:1-17.

Green, T. H. n.d. Martial law in Hawaii: December 7, 1941 - April 4, 1943. Personal papers of Major General Thomas H. Green. [online] http://www.loc.gov/rr/frd/Military_Law/Martial-Law-Hawaii_Green.html

Green, R. C., and M. I. Weisler. 2004. Prehistoric introduction and extinction of animals in Mangareva, Southeast Polynesia. Archaeology in Oceania 39:34-41.

Griffin, P. B. 1984. Where Lohiau ruled: excavations at Ha‘ena, Halele‘a, Kaua‘i. Hawaiian Archaeology 1:1-18.

Griffin, P. B., R. M. Bordner, H. H. Hammatt, M. E. Morgenstein, and C. Stauder. 1977. Preliminary archaeological investigations at Ha‘ena, Halele‘a, Kaua‘i Island. Archaeological Research Center Hawaii, Lawa‘i.

Grigg, R. W. 1993. Precious coral fisheries of Hawaii and the U.S. Pacific Islands. Marine Fisheries Review 55:50-60.

Grigg, R. W. 1994. Effects of sewage discharge, fishing pressure and habitat complexity on coral ecosystems and reef fishes in Hawaii. Marine Ecology Progress Series 103:25-34.

Grigg, R. W. 1995. Coral reefs in an urban embayment in Hawaii: a complex case history controlled by natural and anthropogenic stress. Coral Reefs 14:253-266.

Grigg, R. W. 1997. Paleoceanography of coral reefs in the Hawaiian-Emperor Chain - revisited. Coral Reefs 16:S33-S38.

Grigg, R. W., and R. T. Pfund, editors. 1980. Proceedings of the symposium on status of resource investigations in the northwestern Hawaiian Islands, 24-25 April 1980. University of Hawai‘i Sea Grant College Program, UNIHI-SEAGRANT-MR 80-04, University of Hawai‘i. Honolulu.

Grigg, R. W., and K. Y. Tanoue, editors. 1984. Proceedings of the second symposium on resource investigations in the northwestern Hawaiian Islands, 25-27 May 1983. University of Hawai‘i Sea Grant College Program, UNIHI-SEAGRANT-MR 84-01, University of Hawai‘i. Honolulu.

Gulko, D., J. Maragos, A. Friedlander, C. Hunter, and R. Brainard. 2000. Status of coral reefs in the Hawaiian Archipelago. Pages 219–238 in C. Wilkinson, editor. Status of coral reefs of the world. Australian Institute of Marine Science, Cape Ferguson, Queensland, AU.

Haan, A., and A. L. Tester. 1949. Hawaii’s fishing industry. Hawaii Educational Review 38:60-61, 70, 76, 82.

Hadden, F. C. 1941. Midway Islands. Hawaiian Sugar Planters’ Association, Honolulu. Reprint from The Hawaiian Planters' Record, Vol XLV, No. 3, 1941 (Pages 179-221).

Haight, W. R., D. R. Kobayashi, and K. E. Kawamoto. 1993. Biology and Management of Deepwater Snappers of the Hawaiian Archipelago. Marine Fisheries Review 55:20-27.

Hall, E. O. 1839. Notes of a tour around Oahu. Hawaiian Spectator 2:94-112.

Hamamoto, H. 1928. The fishing industry of Hawaii. Theses for the degree of Bachelor of Arts, University of Hawai‘i, no. 10, Honolulu. 45 pgs.

Hammatt, H. H., M. J. Tomonari-Tuggle, and C. Streck. 1978a. Archaeological investigations at Ha‘ena State Park, Halele‘a, Kaua‘i Island. Phase II: Excavations of beach localities and visitors facilities area. Archaeological Research Center Hawaii, Lawa‘i, Kaua‘i.

Handy, E. S. C., and E. G. Handy 1972. Native planters in old Hawaii: Their life, lore and the environment. Bishop Museum Press, Honolulu.

Harbors Division Planning Section. 1997. History of Oahu's harbors. Harbors Division, Dept. of Transportation, Honolulu, HI.

Harwood, P. J. 1929. Description of Midway Islands, historical and commercial comments by P. J. Harwood, Cable Company official. Honolulu Advertiser, 2 Jan. 1929, Ed. page, Honolulu.

Hawaii Cooperative Fishery Research Unit. 2008. Biology of parrotfish in Hawaii. Western Pacific Regional Fishery Management Council, Honolulu, Hawaii, USA.

Hawaii State Department of Business Economic Development & Tourism (DBEDT). 2004. The state of Hawaii data book, Honolulu.

Hawaiian Roots. 2006. Ships to Hawaii before 1819. Hawaiian Roots: Genealogy for Hawaiians, Honolulu.

HawaiiHistory.org. N.D. Hawaii timeline. Info Grafik Inc., Honolulu.

Hays, G. C. 2004. Good news for sea turtles. Trends in Ecology & Evolution 19:349-351.

He, X., K. A. Bigelow, and C. H. Boggs. 1997. Cluster analysis of longline sets and fishing strategies within the Hawaii-based fishery. Fisheries Research 31:147-158.

Helen, F. J., and P. P. Jonathan. 2008. Integration of palaeontological, historical, and geographical data on the extinction of koa-finches. Diversity and Distributions 14:441-451.

Herman, L. M. 1979. Humpback whales Megaptera-novaeangliae in Hawaiian USA waters: A study in historical ecology. Pacific Science 33:1-16.

Higham, T. F. G., and A. G. Hogg. 1997. Evidence for late Polynesian colonization of New Zealand: University of Waikato radiocarbon measurements. Radiocarbon 39:149-192.

Hirata, J., L. Potts, and R. Pearson. 1971. Preliminary report on the excavation of Cave 1, Kalaupapa Peninsula, Molokai, Hawaii. Department of Anthropology and Sociology, University of British Columbia, Vancouver, B.C.

Holdaway, R. N. 1999. A spatio-temporal model for the invasion of the New Zealand archipelago by the Pacific rat Rattus exulans. Journal of the Royal Society of New Zealand 29:91-105.

Holzwarth, S. R., E. E. DeMartini, R. E. Schroeder, B. J. Zgliczynski, and J. L. Laughlin. 2006. Sharks and jacks in the Northwestern Hawaiian Islands from towed-diver surveys 2000-2003. Pages 257-280 in G. T. DiNardo, and F. A. Parrish, editors. Northwestern Hawaiian Islands third scientific symposium. Atoll Research Bulletin, Vol. 543, National Museum of Natural History, Smithsonian Institute, Honolulu, HI, 2-4 November 2004.

Hommon, R. J. 1975. Use and control of Hawaiian inter-island channels Polynesian Hawaii: A.D. 1400-1794, Unpublished Document, Office of the Governor, Hawaii, 1975.

Hommon, R. J. 1976. The formation of primitive states in pre-contact Hawaii. PhD Dissertation. University of Arizona, Tucson.

Hommon, R. J. 1980. National register of historic places inventory - nomination form: Kaho'olawe, U.S. Department of the Interior, National Park Service.

Hommon, R. J. 1983. Archaeological data recovery at site 342, Kalahuipuaʻa, Hawaii. Science Management, Inc., Honolulu.

Hommon, R. J. 1993. Through volcanic glass, darkly. Hawaiian Archaeology 2:86-94.

Hoover, J. P. 2006. Hawaii's Sea Creatures, a Guide to Hawaii's Marine Invertebrates. Mutual Publishing, Honolulu, Hawaii.

Horcajo, K. 2003. Native Hawaiian History Timeline. Office of Hawaiian Affairs, Honolulu.

Huber, D. R. 1986. Ancient Hawaiian Fishponds of O‘ahu: A Bibliography of Map Resources, Honolulu.

Hudgins, L. L. 1980. Per capita annual utilization and consumption of fish and shellfish in Hawaii, 1970-77. Marine Fisheries Review 42:16-20.

Hughes, T. P., A. H. Baird, D. R. Bellwood, M. Card, S. R. Connolly, C. Folke, R. Grosberg, O. Hoegh-Guldberg, J. B. C. Jackson, J. Kleypas, J. M. Lough, P. Marshall, M. Nystrom, S. R. Palumbi, J. M. Pandolfi, B. Rosen, and J. Roughgarden. 2003. Climate change, human impacts, and the resilience of coral reefs. Science 301:929-933.

Huisman, J. M., I. A. Abbott, and C. M. Smith. 2008. Hawaiian Reef Plants. University of Hawai‘i Sea Grant College Program, Honolulu.

Hunnewell, J. F., W. T. Brigham, and S. B. Dole. 1869. Bibliography of the Hawaiian Islands. Printed for James F. Hunnewell. Based on "A catalogue of works published at, or relating to, the Hawaiian islands" comp. by W. T. Brigham, Sanford B. Dole, and James F. Hunnewell, and published as one of the "Hawaiian club Papers," Oct. 1868. Preceded by an essay on the "Civilization of the Hawaiian islands" (p. 4-17), Boston.

Hunt, T. L. 2005. Archaeological stratigraphy and chronology and Nu‘alolo Kai, Na Pali District, Kaua‘i in M. T. Carson, and M. G. Graves, editors. Na Mea Kahiko o Kaua‘i: Archaeological studies in Kaua‘i. Special Publication No. 2, Society for Hawaiian Archaeology, Honolulu.

Hunt, T. L., and R. M. Holsen. 1991. An early radiocarbon chronology for the Hawaiian Islands: a preliminary analysis. Asian Perspectives 30:147-161.

Hunt, T. L., and C. P. Lipo. 2006. Late colonization of Easter Island. Science 311:1603-1606.

Hunt, T. L., and C. P. Lipo. 2001. Cultural elaboration and environmental uncertainty in Polynesia. Pages 103-115 in C. M. Stevenson, G. Lee, and F. Morin, editors. Pacific 2000: Proceedings of the Fifth International Conference on Easter Island and the Pacific. Easter Island Foundation, Los Osos.

Hunt, T. L. 2007. Rethinking Easter Island’s ecological catastrophe. Journal of Archaeological Science 34:485–502.

Hunt, T. L., and C. P. Lipo. 2007. Chronology, deforestation, and “collapse:” Evidence vs. faith in Rapa Nui prehistory. Rapa Nui Journal 21:85-97.

Hunt, T. L., and C. P. Lipo. 2009. Ecological catastrophe, collapse, and the myth of "ecocide" on Rapa Nui (Easter Island). Pages 21-44 in P. A. McAnany, and N. Yoffee, editors. Questioning Collapse: Human Resilience, Ecological Vulnerability and the Aftermath of Empire. Cambridge University Press, New York.

Hunter, C. L., and C. W. Evans. 1995. Coral reefs in Kaneohe Bay, Hawaii: two centuries of western influence and two decades of data. Bulletin of Marine Science 57:501-515.

IARII. 2008. IARII Report List. International Archaeological Research Institute, Inc., Honolulu, HI.

Ii, J. P. 1993. Fragments of Hawaiian History. Translated Hawaiian to English by Mary Kawena Pukui, Dorothy B. Barrère, ed. Bishop Museum Press, Honolulu.

Ingram, W. M. 1939. Endemic Hawaiian cowries. Bernice P. Bishop Museum, Occasional Papers 14:327-333.

Ingram, W. M. 1947. Hawaiian Cypraeidae. Bernice P. Bishop Museum, Occasional Papers 19:1-23.

Inouye, S. 1931. Honolulu’s sampan fishers brave perils and hardships to garner finny harvest. The Honolulu Advertiser, 9 Sept. 1931, Honolulu.

Inouye, S. 1933. Honolulu’s sampan fishers. Mid-Pacific Magazine, Honolulu.

Iversen, R. T. B., T. Dye, and L. M. B. Paul. 1990. Native Hawaiian fishing rights: a report. Pacific Fisheries Consultants, Honolulu.

Jackson, J. B. C. 1997. Reefs since Columbus. Coral Reefs 16:S23-S32.

Jackson, J. B. C. 2001. What was natural in the coastal oceans? Proceedings of the National Academy of Sciences of the United States of America 98:5411-5418.

Jackson, J. B. C., M. X. Kirby, W. H. Berger, K. A. Bjorndal, L. W. Botsford, B. J. Bourque, R. H. Bradbury, R. Cooke, J. Erlandson, J. A. Estes, T. P. Hughes, S. Kidwell, C. B. Lange, H. S. Lenihan, J. M. Pandolfi, C. H. Peterson, R. S. Steneck, M. J. Tegner, and R. R. Warner. 2001. Historical Overfishing and the Recent Collapse of Coastal Ecosystems. Science 293:629-637.

Jackson, J. B. C., J. C. Ogden, J. M. Pandolfi, N. Baron, R. H. Bradbury, H. M. Guzman, T. P. Hughes, C. V. Kappel, F. Micheli, H. P. Possingham, and E. Sala. 2005. Reassessing US coral reefs - Response. Science 308:1741-1742.

James, H. F., T. W. Stafford, D. W. Steadman, S. L. Olson, P. S. Martin, A. J. T. Jull, and P. C. McCoy. 1987. Radiocarbon dates on bones of extinct birds from Hawaii. Proceedings of the National Academy of Sciences 84:2350-2354.

Jarves, J. J. 1843. History of the Hawaiian or Sandwich Islands. Tappan & Dennett, Boston.

Jenkins OP (1900). Descriptions of new species of fishes from the Hawaiian Islands, belonging to the families of Labridae and Scaridae. U.S. Commission of Fish and Fisheries, Document 433. Extracted from U.S. Fish Commission Bulletin for 1899, U.S. Government Printing Office, Washington, DC.

Jenkins OP (1902). Descriptions of fifteen new species of fishes from the Hawaiian Islands. U.S. Commission of Fish and Fisheries, Document 456. Extracted from U.S. Fish Commission Bulletin for 1899, U.S. Government Printing Office, Washington, DC.

Jenkins OP (1903). Report on collections of fishes made in the Hawaiian Islands, with descriptions of new species. Extracted from U.S. Fish Commission Bulletin for 1902, U.S. Government Printing Office, Washington, DC.

Johannes, R. 2002a. Did indigenous conservation ethic exist? SPC Traditional Management Bulletin 14:3-7.

Johannes, R. E. 1978. Traditional marine conservation methods in Oceania and their demise. Annual Reviews in Ecology and Systematics 9:349-364.

Johannes, R. E. 1981. Words of the lagoon: fishing and marine lore in the Palau District of Micronesia. University of California Press, Berkeley.

Johannes, R. E. 2002b. The renaissance of community-based marine resource management in Oceania. Annual Reviews in Ecology and Systematics 33:317-340.

Johannes, R. E., and W. MacFarlane. 1991. Traditional fishing in the Torres Strait Islands. Commonwealth Scientific and Industrial Research Organisation, Hobart, AU.

Johnson, B. W., and P. A. Johnson. 1981. Estimating the Hawaiian monk seal on Laysan Island. Final Report to U.S. Marine Mammal Commission. National Technical Information Service, Bethesda, MD.

Jokiel, P., K. u. Rodgers, I. Kuffner, A. Andersson, F. Cox, and F. Mackenzie. 2009. Impact of ocean acidification on Hawaiian coral reefs in the 21st century. Paper presented at the 2009 Hawaii Conservation Conference, Honolulu.

Jokiel, P. L. 1987. Ecology, biogeography and evolution of corals in Hawaii. Trends in Ecology & Evolution 2:179-182.

Jokiel, P. L. 2008. Biology and ecological functioning of coral reefs in the main Hawaiian Islands. Pages 489-518 in B. M. Riegl, and R. E. Dodge, editors. Coral Reefs of the USA. Springer, Berlin.

Jokiel, P. L., E. K. Brown, A. Friedlander, S. K. Rodgers, and W. R. Smith. 2004. Hawaii coral reef assessment and monitoring program: spatial patterns and temporal dynamics in reef coral communities. Pacific Science 58:159-174.

Jokiel, P. L., C. L. Hunter, S. Taguchi, and L. Watarai. 1993. Ecological impact of a fresh-water reef kill in Kaneohe Bay, Oahu, Hawaii. Coral Reefs 12:177-184.

Jokiel, P. L., and K. S. Rodgers. 2007. Ranking coral ecosystem "health and value" for the islands of the Hawaiian Archipelago. Pacific Conservation Biology 13:60-68.

Jones, J. C. 1948. Dispatch of John C. Jones, U.S. Agent for Commerce, to John Quincy Adams, Secretary of State, 31 December 1821. Page 69 in T. Morgan, editor. Hawaii: A Century of Economic Change. Harvard University Press, Cambridge.

Jones, S. 2007. Human impacts on ancient marine environments of Fiji's Lau group: current ethnoarchaeological and archaeological research. The Journal of Island and Coastal Archaeology 2:239-244.

Jones, S., and P. Kirch. 2007. Indigenous Hawaiian fishing practices in Kahikinui Maui: a zooarchaeological approach. Hawaiian Archaeology 11:39-53.

Jordan, D. S. 1922. Description of deep-sea fishes from the coast of Hawaii, killed by a lava flow from Mauna Loa. Proceedings of the United States National Museum 59:643-656 (illus. 624 cm).

Jordan, D. S., and M. C. Dickerson. 1908. On a collection of fishes from Fiji, with notes on certain Hawaiian fishes. Proceedings of the United States National Museum 34:603-617.

Jordan, D. S., and B. W. Evermann. 1902. Preliminary report on an investigation of the fishes and fisheries on the Hawaiian Islands. Pages 353-499. U.S. Fish Commission Report for 1901. Pages 353-499, Plates 21-27. Government Printing Office, Washington, DC.

Jordan, D. S., and B. W. Evermann. 1903a. Descriptions of a new genus and two new species of fishes from the Hawaiian Islands. Pages 209-210. Government Printing Office, Washington, DC.

Jordan, D. S., and B. W. Evermann. 1903b. Descriptions of new genera and species of fishes from the Hawaiian Islands. Extracted from U.S. Fish Commission Bulletin for 1902. Pages 161-208. Date of publication April 10, 1903, Government Printing Office, Washington, DC.

Jordan, D. S., B. W. Evermann, and P. Fiene-Severns. 1990. Hawaiian fish: a postcard book of 30 fish drawings by ichthyologists David Starr Jordan and Barton Warren Evermann. Mutual Publishing, Honolulu, Hawaii.

Jordan, D. S., B. W. Evermann, C. H. Gilbert, J. N. Cobb, W. R. Coe, W. K. Fisher, A. G. Mayor, C. C. Nutting, A. E. Ortmann, M. J. Rathbun, H. Richardson, A. L. Treadwell, F. W. True, United States Bureau of Fisheries, Albatross (Steamer), and United States Fish Commission. 1905. The aquatic resources of the Hawaiian Islands. Bulletin of the United States Fish Commission, Government Printing Office, Washington, DC

Jordan, D. S., B. W. Evermann, and S. Tanaka. 1927. Notes on new or rare fishes from Hawaii. Proceedings of the California Academy of Sciences XVI:649-680, pl. 622-624.

Jordan, D. S., and E. K. Jordan. 1922. A list of the fishes of Hawaii, with notes and descriptions of new species. Memoirs of the Carnegie Museum X:92.

Jordan, D. S., and C. W. Metz. 1912. Descriptions of two new species of fishes, from Honolulu, Hawaii. Pages p. 525-527. pl. 571, Washington.

Jordan, D. S., and J. O. Snyder. 1904a. Description of a new species of fish (Apogon Evermann) from the Hawaiian Islands, with notes on other species. Proceedings of the United States National Museum 28:123-126.

Jordan, D. S., and J. O. Snyder. 1904b. Notes on collections of fishes from Oahu Island and Laysan Island, Hawaii, with descriptions of four new species. Proceedings of the United States National Museum XXVII:939-948.

Jordan, D. S., and J. O. Snyder. 1907. Notes on fishes of Hawaii, with descriptions of new species. Pages 207-218. Government Printing Office, Washington, DC.

Judd, L. F. 1880. Honolulu: Sketches of Life: Social, Political, and Religious, in the Hawaiian Islands from 1828-1861. With a Supplementary Sketch of Events to 1880. Anson D. F. Randolph & Company, New York.

Juvik, S. P., and J. O. Juvik, editors. 1998. Atlas of Hawai‘i. University of Hawai‘i Press, Honolulu.

Kaha‘ulelio, D. 2006. M. K. Pukui, and M. P. Nogelmeier, editors. Ka ‘Oihana Lawai‘a: Hawaiian Fishing Traditions. Bishop Museum Press and Awaiaulu Press, Honolulu.

Kalakaua, D. n.d. Concerning the fish attracting stones. Hawaiian Ethnological Notes 2 (1869): 241. Translated by Mary Kawena Pukui. Bishop Museum Library, Honolulu.

Kalakaua, D. 1888. The legends and myths of Hawaii. Charles L. Webster & Company, New York.

Kamakau, S. M. 1961. Ruling chiefs of Hawaii. Kamehameha Schools Press, Honolulu.

Kamakau, S. M. 1976. The works of the people of old: Na hana a ka po‘e kahiko. Translated by Mary Kawena Pukui, Edited by Dorothy B. Barrere. Bishop Museum Press, Honolulu.

Kamakau, S. M. 1992. The people of old. Translated from the newspaper Ke Au ‘Oko‘a by Mary Kawena Pukui; arranged and edited by Dorothy B. Barrère; illustrated by Joseph Feher. Bernice P. Bishop Museum, Special Publication No. 51, Honolulu.

Kamakau, S. M. 1993. Tales and traditions of the people of old = Na Mo‘olelo a Ka Po‘e Kahiko (Translated from the newspapers Ka Nupepa Kuokoa and Ke Au ‘Oko‘a by Mary Kawena Pukui, edited by Dorothy B. Barrère). Bishop Museum Press, Special Publication #94, Honolulu.

Kanahele, G. S. 1996. Waikiki, 100 BC to 1900 AD: an untold story. University of Hawai‘i Press, Honolulu.

Kane, H. K. 1997. Ancient Hawai‘i. The Kawainui Press, Captain Cook, HI.

Kaneshiro, K. Y., P. Chinn, K. N. Duin, A. P. Hood, K. Maly, and B. A. Wilcox. 2005. Hawai‘i’s mountain-to-sea ecosystems: social–ecological microcosms for sustainability science and practice. EcoHealth 2:349-360.

Kay, E. A., editor. 1976. A natural history of the Hawaiian Islands: selected readings. University of Hawai‘i Press, Honolulu.

Kay, E. A. 1979. Hawaiian marine shells. Bishop Museum Press, Honolulu.

Keegan, W. F., and J. M. Diamond. 1987. Colonization of islands by humans: A biogeographical perspective. Pages 49–92 in M. Schiffer, editor. Advances in Archaeological Method and Theory. Academic Press, San Diego, CA.

Keenan, E. E., R. E. Brainard, and L. V. Basch. 2006. Historical and present status of the pearl oyster, Pinctada margaritifera, at Pearl and Hermes Atoll, northwestern Hawaiian Islands. Atoll Research Bulletin 543:333-344.

Keliipio, L. D. 1900. Names of Fish Known to the Honolulu Market. Page 45 in T. G. Thrum, editor. Hawaiian Almanac and Annual for the Year 1900. Press Publishing Company, Honolulu.

Keliipio, L. D. 1901. Hawaiian fish stories and superstitions. Pages 110-114 in T. G. Thrum, editor. Hawaiian Almanac and Annual for the Year 1901. Press Publishing Company, Honolulu.

Kelly, H. L. 1925. Some aspects of Hawaii's fish problem. Pages 849-854. The Mid-Pacific Magazine, Honolulu.

Kelly, H. L. 1931. The fish situation in Hawaii. Pages 39-46 in T. Thrum, editor. The Hawaiian Annual for 1931. Thrum, Honolulu.

Kelly, M. 1956. Changes in land tenure in Hawaii. University of Hawai‘i, Theses for the degree of Masters of Arts, Pacific Islands Studies No. 346, Honolulu.

Kelly, M. 1969. Historical background of the South Point area, Ka‘u, Hawaii. Pacific Anthropological Records 6, Dept. of Anthropology, Bernice P. Bishop Museum, Honolulu, Hawaii, USA.

Kelly, M. 1989. Dynamics of production intensification in precontact Hawaii. Pages 82-106 in S. van der Leeuw, and R. Torrence, editors. What's New?: A Closer Look at the Process of Innovation. Unwin Hyman, Boston.

Kennett, D. J., and B. Winterhalder, editors. 2008. Islands of Inquiry: Colonisation, Seafaring and the Archaeology of Maritime Landscapes (Terra Australis 29). Australia National University Press, Canberra, AU.

Kenyon, J. C., and R. E. Brainard. 2006. Second recorded episode of mass coral bleaching in the Northwestern Hawaiian Islands. Atoll Research Bulletin 543:505-523.

Kenyon, J. C., M. J. Dunlap, and G. S. Aeby. 2008a. Community structure of hermatypic corals at Kure Atoll, Northwestern Hawaiian Islands: stemming the shifting baseline. Atoll Research Bulletin 559. [online] http://www.botany.hawaii.edu/faculty/duffy/atoll.htm

Kenyon, J. C., M. J. Dunlap, C. B. Wilkinson, K. N. Page, P. S. Vroom, and G. S. Aeby. 2007a. Community structure of hermatypic corals at Pearl and Hermes Atoll, Northwestern Hawaiian Islands: unique conservation challenges within the Hawaiian Archipelago. Atoll Research Bulletin 549. [online] http://www.botany.hawaii.edu/faculty/duffy/atoll.htm

Kenyon, J. C., C. B. Wilkinson, and G. S. Aeby. 2008b. Community structure of hermatypic corals at Maro Reef in the Northwestern Hawaiian Islands: a unique open atoll. Atoll Research Bulletin 558. [online] http://www.botany.hawaii.edu/faculty/duffy/atoll.htm

Kenyon, J. C., C. B. Wilkinson, M. J. Dunlap, G. S. Aeby, and C. Kryss. 2007b. Community structure of hermatypic corals at Laysan Island and Lisianski Island/Neva Shoal in the Northwestern Hawaiians: a new layer of scientific exploration. Atoll Research Bulletin 550. [online] http://www.botany.hawaii.edu/faculty/duffy/atoll.htm

Kenyon, K. W. 1972. Man versus the monk seal. Journal of Mammalogy 53:687-696.

Kenyon, K. W., and D. W. Rice. 1959. Life history of the Hawaiian monk seal. Pacific Science 13:215-252.

Kepelino, Z. K. N.D. Ka Mo‘olelo o na ia Havaii (The story of fish in Hawaii) [in Hawaiian]. Manuscript Collection. Bernice P. Bishop Museum, Honolulu, Hawaii, USA.

Kikiloi, K. 2003. A new synthesis in oceanic domestication: The symbiotic development of loko i'a aquaculture in pre-contact Oceania. SPC Traditional Marine Resource Management and Knowledge Information Bulletin 15:3-10. [online] http://www.spc.int/coastfish/News/Trad/15/Kikiloi.pdf

Kikiloi, K. 2010. Rebirth of an archipelago: sustaining a Hawaiian cultural identity for people and homeland. Hulili: Multidisplinary Research on Hawaiian Well-Being 6:73-114.

Kikiloi, K. 2010. Personal communication on archaeology of Nihoa and Necker Islands in the northwestern Hawaiian Islands, to J. N. Kittinger, Honolulu.

Kikuchi, W. K. 1976. Prehistoric Hawaiian fishponds. Science 193:295-299.

Kikuchi, W. K., and J. C. Belshé. 1971. Examination and evaluation of fishponds on the leeward coast of the island of Hawaii. Prepared for Hawaii County Planning Commission, Hilo, Hawaii.

Kikuchi, W. K., and D. A. Burney. 1998. Preliminary report on archaeological excavations at Site 50-30-10-3097, Maha‘ulepiu, District of Koloa, Kaua‘i. State of Hawaii, Historic Preservation Division, Honolulu.

Kikuchi, W. K. 1963. Archaeological Survey and Excavations on the Island of Kauai, Kona District, Hawaiian Islands. Committee for the Preservation of Hawaiian Culture.

Kingdom of Hawaii. 1848. Answers to questions: a survey of missionaries of Hawaii. Kingdom of Hawaii, Department of Foreign Affairs, Report, 1848. Hawaii State Archives, Honolulu.

Kinsey, D. W. 1986. Effects of Run-off, Siltation, and Sewage. Pages 181-189. Fringing Reef Workshop - Science, Industry, and Management. Great Barrier Reef Marine Park Authority, Arcadia Resort, Magnetic Island, AU.

Kirch, D. C., and P. V. Kirch. 1987. Impact of tourism on archaeological resources in Hawaii. Annals of Tourism Research 14:145–156.

Kirch, P., and J. Kahn. 2007. Advances in Polynesian prehistory: a review and assessment of the past decade (1993–2004). Journal of Archaeological Research 15:191-238.

Kirch, P. V. 1973a. Archaeological excavations at Kahalu‘u North Kona, Island of Hawaii. Prepared for Kamehameha Development Corporation, Honolulu.

Kirch, P. V. 1973b. Archaeological excavations at Site D13-1, Hawea Point, Maui, Hawaiian Islands. Prepared for Maui Land and Pineapple Co., Ltd. Ms. 091173, Department of Anthropology, B. P. Bishop Museum, Honolulu.

Kirch, P. V. 1975a. Excavations at Sites A1-3 and A1-4: early settlement and ecology in Halawa Valley. Pages 17-70 in P. V. Kirch, and M. Kelly, editors. Prehistory and ecology in a windward Hawaiian valley: Halawa Valley, Molokai. Pacific Anthropological Records 24, Department of Anthropology, Bernice P. Bishop Museum, Honolulu.

Kirch, P. V. 1975b. Halawa Valley in Hawaiian prehistory: discussion and conclusions. Pages 167-184 in P. V. Kirch, and M. Kelly, editors. Prehistory and ecology in a windward Hawaiian valley: Halawa Valley, Molokai. Pacific Anthropological Records No. 24, Dept of Anthropology, Bernice P. Bishop Museum, Honolulu.

Kirch, P. V. 1977. Valley agricultural systems in prehistoric Hawaii: an archaeological consideration. Asian Perspectives 20:246-280.

Kirch, P. V. 1979a. Late prehistoric and early historic settlement-subsistence patterns in the Anahulu Valley, O‘ahu. Department of Anthropology, Report Series 79-2, Bernice P. Bishop Museum, Honolulu.

Kirch, P. V. 1979b. Marine exploitation in prehistoric Hawaii: archaeological excavations at Kalahuipua‘a, Hawaii Island. Pacific Anthropological Records 29, Department of Anthropology, Bernice P. Bishop Museum, Honolulu.

Kirch, P. V. 1980. Polynesian prehistory: Cultural adaptation in island ecosystems. American Scientist 68:39-48.

Kirch, P. V. 1982a. The ecology of marine exploitation in prehistoric Hawaii. Human Ecology 10:455-476.

Kirch, P. V. 1982b. The impact of the prehistoric Polynesians on the Hawaiian ecosystem. Pacific Science 36:1-14.

Kirch, P. V. 1985. Feathered gods and fishhooks: An introduction to Hawaiian archaeology and prehistory. University of Hawai‘i Press, Honolulu.

Kirch, P. V., editor. 1989. Prehistoric Hawaiian occupation in the Anahulu Valley, Oahu Island: Excavations in three inland rockshelters. Contributions of the University of California Archaeological Research Facility No. 47. Archaeological Research Facility, Berkeley.

Kirch, P. V. 1990a. The evolution of sociopolitical complexity in prehistoric Hawaii: An assessment of the archaeological evidence. Journal of World Prehistory 4:311-345.

Kirch, P. V. 1990b. Monumental architecture and power in Polynesian chiefdoms: A comparison of Tonga and Hawaii. World Archaeology 22:206-222.

Kirch, P. V. 1997. Microcosmic histories: island perspectives on "global" change. American Anthropologist 99:30-42.

Kirch, P. V., editor. 2002a. From the ‘Cliffs of Keolewa’ to the ‘Sea of Papaloa’: an archaeological reconnaissance of portions of the Kalaupapa National Historical Park, Moloka‘i, Hawaiian Islands. Oceanic Archaeology Laboratory, Special Publication No. 2. Archaeological Research Facility, University of California, Berkeley.

Kirch, P. V. 2002b. On the road of the winds: An archaeological history of the Pacific Islands before European contact. University of California Press, Berkeley.

Kirch, P. V. 2005. From chiefdom to archaic state: social evolution in Hawaii. Grace Elizabeth Schallit Lecture, February 2005, Brigham Young University, Provo.

Kirch, P. V. 2007a. Hawaii as a model system for human ecodynamics. American Anthropologist 109:8-26.

Kirch, P. V. 2007b. "Like shoals of fish": archaeology and population in pre-contact Hawaii. Pages 52-69 in P. V. Kirch, and J.-L. Rallu, editors. The growth and collapse of Pacific Island societies: archaeological and demographic perspectives. University of Hawai‘i Press, Honolulu, HI.

Kirch, P. V. 2007c. Three islands and an archipelago: reciprocal interactions between humans and island ecosystems in Polynesia. Earth and Environmental Science Transactions of the Royal Society of Edinburgh 98:85-99.

Kirch, P. V., J. Coil, A. S. Hartshorn, M. Jeraj, P. M. Vitousek, and O. A. Chadwick. 2005. Intensive dryland farming on the leeward slopes of Haleakala, Maui, Hawaiian Islands: archaeological, archaeobotanical, and geochemical perspectives. World Archaeology 37:240-258.

Kirch, P. V., and T. S. Dye. 1979. Ethnoarchaeology and the development of Polynesian fishing strategies. Journal of the Polynesian Society 88:53-76.

Kirch, P. V., A. S. Hartshorn, O. A. Chadwick, P. M. Vitousek, D. R. Sherrod, J. Coil, L. Holm, and W. D. Sharp. 2004. Environment, agriculture, and settlement patterns in a marginal Polynesian landscape. Proceedings of the National Academy of Sciences 101:9936-9941.

Kirch, P. V., and M. Kelly 1975. Prehistory and ecology in a windward Hawaiian valley: Halawa Valley, Molokai. Dept. of Anthropology, Bernice Pauahi Bishop Museum, Honolulu.

Kirch, P. V., and M. D. McCoy. 2007. Reconfiguring the Hawaiian cultural sequence: results of re-dating the Halawa dune site (MO-A1-3), Moloka‘i Island. The Journal of the Polynesian Society 116:385-406.

Kirch, P. V., S. O'Day, J. Coil, M. Morgenstein, K. Kawelu, and M. Millerstrom. 2003. The Kaupikiawa rockshelter, Kalaupapa peninsula, Moloka‘i: new investigations and reinterpretation of its significance for Hawaiian prehistory. People and Culture in Oceania 19:1-27.

Kirch, P. V., and S. J. O'Day. 2003. New archaeological insights into food and status: a case study from pre-contact Hawaii. World Archaeology 34:484 - 497.

Kirch, P. V., and J.-L. Rallu, editors. 2007a. The growth and collapse of Pacific Island societies: Archaeological and demographic perspectives. University of Hawai‘i Press, Honolulu.

Kirch, P. V., and J.-L. Rallu. 2007b. Long-term demographic evolution in the Pacific Islands. Pages 1-14 in P. V. Kirch, and J.-L. Rallu, editors. The growth and collapse of Pacific Island societies. University of Hawai‘i Press, Honolulu.

Kirch, P. V., and M. D. Sahlins 1992. Anahulu: the anthropology of history in the Kingdom of Hawaii. University of Chicago Press, Chicago.

Kirch, P. and M. Spriggs. 1993. A radiocarbon chronology for the upper Anahulu Valley, Oíahu. Hawaiian Archaeology 2:4-9.

Kirch, P. V., and W. D. Sharp. 2005. Coral 230Th dating of the imposition of a ritual control hierarchy in precontact Hawaii. Science 307:102-104.

Kirch, P. V., and D. E. Yen. 1982. Tikopia: the prehistory and ecology of a Polynesian outlier. Bishop Museum Press, Honolulu.

Kittinger, J. N. 2009. Konohiki fishing rights. Green Magazine Hawaii Vol. 1, No. 4:44-48.

Kittinger, J. N. 2009. Our modern ahupua‘a: sustainable solutions for Hawaiian communities. Green Magazine Hawaii Vol. 1, No. 1:52-58.

Kittinger, J. N., K. N. Duin, and B. A. Wilcox. 2009. Commercial fishing, conservation and compatibility in the northwestern Hawaiian Islands. Marine Policy 34:208-217.

Kittinger, J. N., D. J. Skillings, K. K. Carvalho, L. L. N. Reeve, M. Hutchinson, K. Cullison, J. Shackeroff, M. Chow, and J. Lemus. 2009. Reconciling ecosystem-based management and focal resource conservation in the Papahānaumokuākea Marine National Monument. Marine Sanctuaries Conservation Series ONMS-09-04. U.S. Department of Commerce, National Oceanic and Atmospheric Administration, Office of National Marine Sanctuaries, Silver Spring, MD. 36 pgs.

Kobayashi, D. R., and K. E. Kawamoto. 1995. Evaluation of shark, dolphin, and monk seal interactions with Northwestern Hawaiian Island bottomfishing activity: a comparison of two time periods and an estimate of economic impacts. Fisheries Research 23:11-22.

Kolb, M. J. 1997. Labor mobilization, ethnohistory, and the archaeology of community in Hawai‘i. Journal of Archaeological Method and Theory 4:265-285.

Kolb, M. J., R. Cordy, T. Earle, G. Feinman, M. W. Graves, C. A. Hastorf, I. Hodder, J. N. Miksic, B. J. Price, and B. G. Trigger. 1994. Monumentality and the rise of religious authority in precontact Hawai‘i [and comments and reply]. Current Anthropology 34:521-547.

Konishi, O. K. 1930a. Fishing industry of Hawaii with special reference to labor. Reports of students in economics and business. University of Hawai‘i, No. 6, Honolulu.

Konishi, O. K. 1930b. Statistical survey of the fluctuation of the auction price of fish. Reports of students in Economics and Business. University of Hawai‘i, No. 4, Honolulu.

Konishi, O. K. 1933. A brief history of fishing in Hawaii. Typescript Manuscript. "Nippu Jiji. 4-9-29”.

Kosaki, R. H. 1954. Konohiki fishing rights. Legislative Reference Bureau, University of Hawai‘i, Honolulu.

Kotzebue, O. v. 1821. Voyage of discovery in the south seas and to Behring's Straits in search of a north-east passage; undertaken in the years 1815, 16, 17, and 18, in the ship Rurick. Sir Richard Phillips and Co., London.

Kraft, J. C. 1983. Impact of Coastal Geological Changes of Shoreline Environments on Man's Occupancy: 400 AD to present, NE O‘ahu Coast, Hawai‘i. Symposium on Archaeological Science in the Pacific Region. University of Otago, Dept. of Anthropology, Dunedin, New Zealand.

Krusenstern, A. J. v. 1821. Voyage autour du Monde, Fait dans les Annees 1803, 1804, 1805, et 1806... sure les Vaisseaux la Nadiejada et la Neva, Commandes par M. de Krusenstern... Librairie de Gide fils, Paris.

Kumu Pono Associates LLC. 2003. Ka Hana Lawai‘a a Me Nā Ko‘a o Na Kai‘ewalu: A history of fishing practices and marine fisheries of the Hawaiian Islands compiled from Native Hawaiian traditions, historical accounts, government communications, Kama‘aina testimony and ethnography, Lana‘i City, Lana‘i, Hawai‘i.

Kuykendall, R. S. 1923. A Northwest Trader at the Hawaiian Islands. Quarterly of the Oregon Historical Society XXIV:111-131.

Kuykendall, R. S. 1926. A history of Hawaii. The Macmillan Company, New York.

Kuykendall, R. S. 1929. Some early commercial adventures of Hawaii. Thirty-Seventh Annual Report of the Hawaiian Historical Society, pp. 15-33, Honolulu.

Kuykendall, R. S. 1938. The Hawaiian Kingdom, Vol. I: 1778-1854 foundation and transformation. University of Hawai‘i Press, Honolulu, Hawaii, USA.

Kuykendall, R. S. 1957. The Hawaiian Kingdom, Vol. II: 1854-1874, Twenty Critical Years. University of Hawai‘i Press, Honolulu, Hawaii, USA.

Kuykendall, R. S. 1967. The Hawaiian Kingdom, Vol. III: The Kalakaua Dynasty, 1874-1893. University of Hawai‘i Press, Honolulu, Hawaii, USA.

La Croix, S. 2001. Economic History of Hawaii in R. Whaples, editor. EH.Net Encyclopedia.

La Pérouse, J.-F. d. G. 1994. The journal of Jean-François de Galaup de la Pérouse, 1785-1788, translated and edited by John Dunmore. Works issued by the Hakluyt Society, 2nd ser., no. 179, 180, London.

La Pérouse, J.-F. o. d. G. 1807. A voyage round the world, performed in the years 1785, 1786, 1787 and 1788, by the Boussole and Astrolabe. Lackington, Allen, London.

Ladd, E. J., editor. 1973. Makaha Valley historical project: Interim Report No. 4. Pacific Anthropological Records 19, Department of Anthropology, Bernice P. Bishop Museum, Honolulu.

Ladd, E. J., and G. F. Somers. 1987. Excavations at site A-27: archaeology at Pu‘uhonua o Honaunau National Historical Park. Publications in anthropology, Western Archeological and Conservation Center No. 43, National Park Service, U.S. Dept. of the Interior.

Ladd, E. J., and D. E. Yen, editors. 1972. Makaha Valley historical project: Interim Report No. 3. Pacific Anthropological Records 18, Department of Anthropology, Bernice P. Bishop Museum, Honolulu.

Ladefoged, T. N., and M. W. Graves. 2006. The formation of Hawaiian territories. Pages 259–283 in I. Lilley, editor. Archaeology of Oceania: Australia and the Pacific Islands. Blackwell Press, New York.

Ladefoged, T. N., M. W. Graves, and M. D. McCoy. 2003. Archaeological evidence for agricultural development in Kohala, Island of Hawai‘i. Journal of Archaeological Science 30:923-940.

Lamb, W. K., editor. 1984. A voyage of discovery to the North Pacific Ocean and round the world 1791-1795. The Hakluyt Society, London.

Lamoureux, C. H. 1961. Botanical observations on leeward Hawaiian Atolls. Atoll Research Bulletin 79. [online] http://www.botany.hawaii.edu/faculty/duffy/atoll.htm

Lamoureux, C. H. 1963. The flora and vegetation of Laysan Island. Atoll Research Bulletin 97. [online] http://www.botany.hawaii.edu/faculty/duffy/atoll.htm

Langdon, R. 1984. Where the Whalers Went. The Australian National University, Canberra.

Langsdorft, G. H. v. 1817. Voyages and Travels in Various Parts of the World, 1803-7. Colburn, London.

Lau, R. 1936. Iron men on wooden ships. The Paradise of the Pacific 48(8):22-24, Honolulu.

Leach, F., and J. Davidson. 2000. Pre-European catches of snapper (Pagrus auratus) in northern New Zealand. Journal of Archaeological Science 27:509-522.

Leach, F., and J. Davidson. 2001. The use of size-frequency diagrams to characterize prehistoric fish catches and to assess human impact on inshore fisheries. International Journal of Osteoarchaeology 11:150-162.

Ledyard, J. 1781. Journal of Captain Cook's Last Voyage to the Pacific Ocean on Discovery...1776-9. Newbery, London.

Lightfoot, K. G., A. Martinez, and A. M. Schiff. 1998. Daily practice and material culture in pluralistic social settings: An archaeological study of culture change and persistence from Fort Ross, California. American Antiquity 63:199-222.

Lisiansky, U. 1814. A voyage round the world, in the years 1803, 4, 5, 6. S. Hamilton, Weybridge, Surrey, London.

Loope, L. L., O. Hamann, and C. P. Stone. 1988. Comparative conservation biology of oceanic archipelagoes: Hawaii and the Galapagos. BioScience 38:272-282.

Lord, P. B. 2002. Can Ancient Hawaii Guide Modern Rhode Island? 41 degrees N. Online 1. [online] http://seagrant.gso.uri.edu/41N/Vol41No43/hawaii.pdf

Lotze, H. K., H. S. Lenihan, B. J. Bourque, R. H. Bradbury, R. G. Cooke, M. C. Kay, S. M. Kidwell, M. X. Kirby, C. H. Peterson, and J. B. C. Jackson. 2006. Depletion, degradation and recovery potential of estuaries and coastal seas. Science 312:1806-1809.

Lotze, H. K., and B. Worm. 2009. Historical baselines for large marine animals. Trends in Ecology & Evolution 24:254-262.

Lowry, K. 1989. Coastal area management: a Hawaii case study. Coastal area management in Southeast Asia: policies, management strategies and case studies, Manila, Philippines

Lowry, K., C. Jarman, and S. Maehara. 1990. Ocean management in Hawaii. Coastal Management 18:233-254.

Luomala, K. 1958. Polynesian myths about Maui and the dog. Manuscript, University of Hawai‘i, Hawaiian-Pacific Collection.

Luomala, K. 1960a. A history of the binominal classification of the Polynesian native dog. Pacific Science 14:193-223.

Luomala, K. 1960b. The native dog in the Polynesian system of values. Pages 190-240 in S. Diamond, editor. Culture in History, Essays in Honor of Paul Radin. Columbia University Press, New York.

Luomala, K. 1962. Additional eighteenth-century sketches of the Polynesian native dog, including the Maori. Pacific Science 16:170-180.

MacCaughey, V. 1916a. Coral reefs of the Hawaiian Islands. Journal of Geography 14:252-255.

MacCaughey, V. 1916b. The seaweeds of Hawaii. Reprinted from the American journal of botany, v.8, Oct. 1916. 8:474-479.

MacCaughey, V. 1917. The physique of the ancient Hawaiians. Reprinted from the Scientific monthly, August 1917.

MacCaughey, V. 1918a. Algae of the Hawaiian Archipelago. Part I. Botanical Gazette 65:42-57.

MacCaughey, V. 1918b. Algae of the Hawaiian Archipelago. Part II. Botanical Gazette 65:121-148.

MacCaughey, V. 1918c. A survey of the Hawaiian coral reefs. The American Naturalist 52:409-438.

Macintyre, I. G., editor. 1996. Laysan Island and other northwestern Hawaiian islands: early science reports with a Laysan Island bibliography. National Museum of Natural History, Smithsonian Institution, Washington, DC. [online] http://www.botany.hawaii.edu/faculty/duffy/atoll.htm

MacKaye, A. L. 1915. Coral of Kaneohe Bay. Pages 135-139 in T. Thrum, editor. Hawaiian Almanac and Annual for 1916. Thrum, Honolulu.

MacKaye, A. L. 1916. Where the coral blossoms bloom. Pages 117-121. Mid-Pacific Magazine, vol. 11, no. 2 (Feb).

Mackenzie, M. K., editor. 1991. Native Hawaiian rights handbook. Native Hawaiian Legal Corporation, Office of Hawaiian Affairs, Distributed by University of Hawai‘i Press, Honolulu.

MacKenzie, M. K. 2003. Native Hawaiian claims to the lands and natural resources of the Northwestern Hawaiian Islands. Report to the Office of Hawaiian Affairs, Honolulu.

Macrae, J. 1922. With Lord Byron at the Sandwich Islands in 1825, being extracts from the MS Diary of James Macrae, Scottish Botanist (William F. Wilson, editor), Honolulu.

Malo, D. 1951. Hawaiian Antiquities (Mo‘olelo Hawaii). Bernice P. Bishop Museum, Honolulu.

Maly, K., and O. Maly. 2003a. "Hana ka lima, ‘ai ka waha": a collection of historical accounts and oral history interviews with kama‘aina residents and fisher-people of lands in the Halele‘a-Napali region on the island of Kaua‘i. Kumu Pono Associates. Prepared for The Nature Conservancy, Honolulu.

Maly, K., and O. Maly. 2003b. He wahi mo‘olelo no na lawai‘a ma Kapalilua, Kona hema, Hawai‘i a collection of historical accounts and oral history interviews with elder kama‘aina fisher-people from the Kapalilua region of south Kona, island of Hawai‘i. Kumu Pono Associates. Prepared for The Nature Conservancy, Honolulu.

Maly, K., and O. Maly. 2003c. Volume 1: Ka hana lawai‘a a me na ko‘a o na kai ‘ewalu: a history of fishing practices and marine fisheries of the Hawaiian Islands, compiled from Native Hawaiian traditions, historical accounts, government communications, kama‘aina testimony and ethnography. Kumu Pono Associates. Prepared for The Nature Conservancy, Honolulu.

Maly, K., and O. Maly. 2003d. Volume 2: Oral History Interviews. Ka hana lawai‘a a me na ko‘a o na kai ‘ewalu: a history of fishing practices and marine fisheries of the Hawaiian Islands, compiled from oral history interviews with kupuna and kama‘aina. Kumu Pono Associates. Prepared for The Nature Conservancy, Honolulu.

Maly, K., and O. Maly. 2004. Ka hana lawai‘a a me na ko‘a o na kai ‘ewalu: Summary of detailed findings from research on the history of fishing practices and marine fisheries of the Hawaiian Islands, compiled from Native Hawaiian traditions, historical accounts, government communications, kama‘aina testimony and ethnography. Kumu Pono Associates. Prepared for The Nature Conservancy, Honolulu.

Manby, T. 1929. Journal of Vancouver's voyage to the Pacific Ocean (1791-1793). Honolulu Mercury, June 1929:11-25; July 1929:33-47; Aug. 1929:39-55.

Mann, H., and J. Remy. 1864-68. H. Mann collection on Hawaiian Islands from the Clements Library. William L. Clements Library.

Maragos, J. 1998. Status of coral reefs of the southwest and east Pacific: Melanesia and Polynesia in C. Wilkinson, editor. Status of coral reefs of the world: 1998. Australian Institute of Marine Science, Townsville, AU.

Maragos, J. E., D. C. Potts, G. Aeby, D. Gulko, J. Kenyon, D. Siciliano, and D. VanRavenswaay. 2004. 2000-2002 Rapid ecological assessment of corals (Anthozoa) on shallow reefs of the Northwestern Hawaiian Islands. Part 1: species and distribution. Pacific Science 58:211-230.

Marean, C. W., L. M. Spencer, R. J. Blumenschine, and S. D. Capaldo. 1992. Captive hyena bone choice and destruction, the schlepp effect and Olduvai archaeofaunas. Journal of Archaeological Science 19:101-121.

Mariner, W., and J. M. Martin 1818. An Account of the Natives of the Tong Islands, in the South Pacific Ocean. John Murray, Albemarle-Street, London.

Marsden, J., J. F. Brown, P. Alexander, and A. T. Atkinson 1896. The Hawaiian Islands: their resources agricultural, commercial and financial. Coffee, the coming staple product. Issued under the auspices of the Department of Foreign Affairs. Hawaiian Gazette Print, Honolulu.

Masse, W. B., and H. D. Tuggle. 1998. The date of Hawaiian colonization. Pages 229–235 in C. M. Stevenson, G. Lee, and F. J. Morin, editors. Easter Island in Pacific context: South Seas symposium: Proceedings of the 4th International Conference on Easter Island and East Polynesia. Easter Island Foundation Occasional Paper 4. Bearsville and Cloud Mountain Presses, Los Osos, California.

Mathison, G. F. 1825. Narrative of a visit to Brazil, Chili, Peru and the Sandwich Islands, during the years 1821-22. Charles Knight, Pall Mall East, London.

Matschie, G. F. P. 1905. Sitzb. Ges. Naturf. Freunde. Page 258, Berlin.

Mazzuca, L., S. Atkinson, and E. Nitta. 1998. Deaths and entanglements of humpback whales, Megaptera novaeangliae, in the Main Hawaiian Islands, 1972-1996. Pacific Science 52:1-13.

McAllister, J. G. 1933a. Archaeology of Kahoolawe. Bernice P. Bishop Museum Bulletin 115, Honolulu (New York: Kraus Reprint, 1971).

McAllister, J. G. 1933b. Archaeology of Oahu. Bernice P. Bishop Museum Bulletin 104, Honolulu.

McAvoy, A. 2009. Ancient objects found on remote Mokumanamana 'an archaeological mystery'. HonoluluAdvertiser.com, Honolulu.

McCoy, M. D. 2005. The development of the kalaupapa field system, Moloka‘i Island, Hawai‘i. Journal of the Polynesian Society 114:339-358.

McCoy, M. D. 2006. Landscape, social memory, and society: An ethnohistoric-archaeological study of three Hawaiian communities. University of California, Berkeley.

McCoy, M. D. 2007. Revised late Holocene culture history for Moloka‘i Island, Hawai‘i. Radiocarbon 49:1273-1322.

McCoy, M. D. 2008. Hawaiian limpet harvesting in historical perspective: a review of modern and archaeological data on Cellana spp. from the Kalaupapa Peninsula, Moloka‘i Island. Pacific Science 62:21-38.

McCoy, M. D., and A. S. Hartshorn. 2007. Wind erosion and intensive prehistoric agriculture: a case study from the Kalaupapa field system, Moloka‘i Island, Hawai‘i. Geoarchaeology 22:511-532.

McCoy, P. C., and R. Gould. 1977. Alpine archaeology in Hawaii. Archaeology 30:234-243.

McDermid, K. J., M. C. Gregoritza, J. W. Reeves, and D. W. Freshwater. 2003. Morphological and genetic variation in the endemic seagrass Halophila hawaiiana (Hydrocharitaceae) in the Hawaiian Archipelago. Pacific Science 57:199-209.

McDermid, K. J., B. Stuercke, and G. H. Balazs. 2007. Nutritional composition of marine plants in the diet of the green sea turtle (Chelonia mydas) in the Hawaiian Islands. Bulletin of Marine Science 81:55-71.

McMullin, J. 2005. The call to life: revitalizing a healthy Hawaiian identity. Social Science & Medicine 61:809-820.

McNeill, J. R. 1994. Of rats and men: a synoptic environmental history of the island Pacific. Journal of World History 5:299-349.

Meares, J. 1790. Voyages made in the years 1788 and 1789, from China to the north west coast of America. Lopographic Press, London.

Meller, N. 1985. Indigenous Ocean Rights in Hawaii. University of Hawai‘i, Sea Grant College Program, Sea Grant Marine Policy and Law Report, Honolulu.

Menzies, A. 1829. Some account of an ascent and barometrical measurement of Wha-ra-rai, a mountain in Owhyhee. London's Magazine of Natural History, Vol. I, pp. 201-208, and vol. II, pp, 435-442, London.

Menzies, A. 1920. Hawai‘i Nei 128 Years Ago: Journal of Archibald Menzies. The New Freedom, Honolulu.

Meyer, C. G., K. N. Holland, B. M. Wetherbee, and C. G. Lowe. 2001. Diet, resource partitioning and gear vulnerability of Hawaiian jacks captured in fishing tournaments. Fisheries Research 53:105-113.

Milberg, P., and T. Tyrberg. 1993. Naive birds and noble savages: A review of man-caused prehistoric extinctions of island birds. Ecography 16:229-250.

Miller, G. M. 1989. America, Hawai‘i and the Sea: The Impact of America on the Hawaiian Maritime Mode of Production, 1778-1850. PhD Dissertation, University of Hawai‘i at Mānoa, American Studies Department, Honolulu.

Mills, P. R. 2002. Hawaii's Russian Adventure: A New Look at Old History. University of Hawai‘i Press, Honolulu.

Mills, P. R. 2003. Neo in Oceania: Foreign vessels owned by Hawaiian chiefs before 1830. The Journal of Pacific History 38:53-67.

Mills, P. R. 2009. Folk housing in the middle of the Pacific: lime architecture, cultural power and ideology in 19th century Hawaii. Pages 75-91 in C. White, editor. Materiality of Individuality. Springer Press.

Minerbi, L. 1999. Indigenous management models and protection of the ahupua‘a. The Ethnic Studies Story: Politics and Social Movements in Hawai‘i: Essays in Honor of Marion Kelly. 208 pgs.

Minister of the Interior. 1890. Biennial Report (1890). Hawaii State Archives, Honolulu.

Minister of the Interior. 1895. Minister of the Interior, Report, 1895. Hawaii State Archives, Honolulu.

Minson, W. H. J. 1952. The Hawaiian journal of Manuel Quimper. Thesis, University of Hawaii, Honolulu.

Mitchell, C., C. Ogura, D. Meadows, A. Kane, L. Strommer, S. Fretz, D. Leonard, and A. McClung. 2005. Hawaii's Comprehensive Wildlife Conservation Strategy. State of Hawaii, Department of Land and Natural Resources, Honolulu, Hawaii, USA.

Moniz, J. 1997. The role of seabirds in Hawaiian subsistence: implications for interpreting avian extinction and extirpation in Polynesia. Asian Perspectives 36:27-50.

Moniz, J. 1999. The archaeology of human foraging and bird resources on the island of Hawaii: the evolutionary ecology of avian predation, resource intensification, extirpation, and extinction. PhD Dissertation, University of Hawai‘i, Honolulu.

Monsarratt Survey. 1897. Honolulu and the harbor, 1897. Hawaii State Archives, Honolulu.

Morell, B. 1832. A narrative of four voyages to the South Sea, North and South Pacific Ocean, Chinese Sea, Ethiopic and Southern Atlantic Ocean, Indian and Antartic Ocean from the year 1822 to 1831. J and J Harper, New York.

Morgan, T. 1948. Hawaii: a century of economic change, 1778-1876. Harvard University Press, Cambridge.

Morgenstein, M. 1978. Geoarchaeological analysis of field remnants, Kawainui Marsh, Kailua, Oahu. Prepared for U.S. Army Corps of Engineers. Hawaii Marine Research, Honolulu, Hawaii.

Mori, R. 1989. Hawaii State Records, Seabass, giant (Hapu‘upu‘u). Hawaii Fishing News, Honolulu. [online] http://www.hawaiifishingnews.com/records_d.cfm?ID=22

Morris, P. C. 1934. How the territory of Hawaii grew and what domain it covers. Pages 13-39. Forty-second annual report of the Hawaiian Historical Society for the year 1933, Honolulu, Hawaii, USA.

Morrison, A. E., and D. J. Addison. 2009. Examining causes and trends in marine trophic level change: 1500 Years of fish exploitation at Fatu-ma-Futi, Tutuila Island, American Samoa. Journal of Island & Coastal Archaeology 4:177-194.

Morrison, A. E., and T. L. Hunt. 2007. Human impacts on the nearshore environment: an archaeological case study from Kaua‘i, Hawaiian Islands. Pacific Science 61:325-345.

Mortimer, G. 1791. Observations and remarks made during a voyage to the Islands of Teneriffe, Amsterdam, Maria's Island near Van Diemen's Land, Otaheite, Sandwich Islands, Owhyhee, the Fox Islands on the north west coast of America, Tinian, and from thence to Canton in the brig Mercury commanded by John Henry Cox. Cadell, London.

Mulder, C., M. Grant-Hoffman, D. Towns, P. Bellingham, D. Wardle, M. Durrett, T. Fukami, and K. Bonner. 2009. Direct and indirect effects of rats: does rat eradication restore ecosystem functioning of New Zealand seabird islands? Biological Invasions 11:1671-1688.

Mulrooney, M. A., and T. N. Ladefoged. 2005. Hawaiian heiau and agricultural production in the Kohala dryland field system. Journal of the Polynesian Society 114:45–67.

Munro, G. C. 1942. Birds of Hawaii and adventure in bird study. Elepaio 3:7.

Munro, G. C. 1942. Midway Island fifty years ago. Paradise of the Pacific, July 1942, p. 17, Honolulu.

Musick, J. R. 1898. Hawaii: Our New Possessions. Funk & Wagnalls Company, New York and London.

Nagaoka, L. 2001. Using diversity indices to measure changes in prey choice at the Shag River Mouth site, southern New Zealand. International Journal of Osteoarchaeology 11:101-111.

Nagaoka, L. 2002. The effects of resource depression on foraging efficiency, diet breadth, and patch use in southern New Zealand. Journal of Anthropological Archaeology 21:419-442.

Nakayama, M., and L. K. Menton. 1987. Maritime industries of Hawaii: A guide to historical resources. Humanities Program of the State Foundation on Culture and the Arts in cooperation with the Hawaiian Historical Society, Honolulu.

Nakuina, E. M. 1904. Hawaii, it's people, their legends. Hawaii Promotion Committee, Hawaiian Gazette Company, Honolulu, Territory of Hawaii.

Newman, T. S. 1970. Hawaiian fishing and farming on the island of Hawaii in A.D. 1778. State of Hawaii, Department of Land and Natural Resources, Division of State Parks, Honolulu.

Newman, T. S. 1972. Man in the prehistoric Hawaiian ecosystem. Pages 559-603 in E. A. Kay, editor. A Natural History of the Hawaiian Islands: Selected Readings. University of Hawai‘i Press, Honolulu.

Niles, W. M. O., editor. 1837. Nile's Weekly Register. Franklin Press, Baltimore.

Nitta, E. T., and J. R. Henderson. 1993. A review of interactions between Hawaii's fisheries and protected species. Marine Fisheries Review 55:83-92.

NMFS. 2007. Recovery Plan for the Hawaiian Monk Seal (Monachus schauinslandi). Page 165. Second Revision. National Marine Fisheries Service (NMFS), Silver Spring, MD. [online] www.nmfs.noaa.gov/pr/species/mammals/pinnipeds/hawaiianmonkseal.htm

Nordhoff, C. 1874. Northern California, Oregon, and the Sandwich Islands. Harper & Brothers, New York.

Nordstedt. 1876. De Algae aquae dulcis et de Characeis ex insulis Sandvicensibus a Sv. BERGGREN 1875 reportatis.

Nordyke, E. C. 1989. The peopling of Hawai‘i. University of Hawai‘i Press, Honolulu.

Norris, R. 1986. A Tide of Plastic. Audubon 88:17-23.

O'Day, S. J. 2002. Excavations at the Kipapa rockshelter, Kahikinui, Maui, Hawai‘i. Asian Perspectives 40:279-304.

O'Day, S. J. 2004. Marine resource exploitation and diversity in Kahikinui, Maui, Hawaii: bringing together multiple lines of evidence to interpret the past. Archaeofauna 13:97-108.

O'Leary, O. 2005a. Analysis of the Nu'alolo-Kai 1/4-inch Fishbone Assemblage, Na Pali Coast, Kaua‘i. SHA Special Publication No. 2, Na Mea Kahiko o Kaua‘i: Archaeological Studies in Kaua‘i, Hawaii, USA.

O'Leary, O. 2005b. Temporal changes in fishing strategies at Nu‘alolo Kai, Na Pali Coast, Kaua‘i. Masters Thesis, University of Hawai‘i, Honolulu.

Office of Hawaiian Affairs. 1999. Native Hawaiian history timeline. Office of Hawaiian Affairs, Honolulu, HI.

Oliver, D. L. 1989. The Pacific Islands. University of Hawai‘i Press, Honolulu.

Olmstead, F. A. 1841. Incidents of a whaling voyage to which are added observations on the scenery, manners and customs, and missionary stations, of the Sandwich and Society Islands, accompanied by numerous lithographic prints. D. Appleton & Co., New York.

Olson, S. L. 1996. History and ornithological journals of the Tanager Expedition of 1923 to the Northwestern Hawaiian Islands, Johnston and Wake Islands. Atoll Research Bulletin 433. [online] http://www.botany.hawaii.edu/faculty/duffy/atoll.htm

Osbun, A. G. 1966. To California and the South Seas; the diary of Albert G. Osbun, 1849-1851. Edited by John Haskell Kemble. Huntington Library, San Marino, CA.

Paishon-Duarte, M. 2008. Personal communication, a Hawaiian perspective on oceans, health and well-being, to J. N. Kittinger. Honolulu.

Pandolfi, J. M., R. H. Bradbury, E. Sala, T. P. Hughes, K. A. Bjorndal, R. G. Cooke, D. McArdle, L. McClenachan, M. J. H. Newman, G. Paredes, R. R. Warner, and J. B. C. Jackson. 2003. Global trajectories of the long-term decline of coral reef ecosystems. Science 301:955.

Pandolfi, J. M., R. H. Bradbury, E. Sala, T. P. Hughes, K. A. Bjorndal, R. G. Cooke, D. McArdle, L. McClenachan, M. J. H. Newman, G. Paredes, R. R. Warner, and J. B. C. Jackson. 2003. Causes of coral reef degradation - Response. Science 302:1502-1503.

Pandolfi, J. M., J. B. C. Jackson, N. Baron, R. H. Bradbury, H. M. Guzman, T. P. Hughes, C. V. Kappel, F. Micheli, J. C. Ogden, H. P. Possingham, and E. Sala. 2005. Are U.S. Coral Reefs on the Slippery Slope to Slime? Science 307:1725-1726.

Paty, J. 1857. Log of the Manuokawai, April and May. Hawaii State Archives, Honolulu.

Paulding, H. 1831. Journal of a cruise of the US schooner Dolphin. G. & C. & H. Carvill, New York.

Pauly, D. 1995. Anecdotes and the shifting baseline syndrome of fisheries. Trends in Ecology and Evolution 10:430.

Paxinos, E. E., H. F. James, S. L. Olson, J. D. Ballou, J. A. Leonard, and R. C. Fleischer. 2002. Prehistoric decline of genetic diversity in the nene. Science 296:1827-1827.

Peale, T. R. 1848. Mammalia and ornithology in C. Wilkes, editor. United States exploring expedition, during the years 1838, 1839, 1840, 1841, 1842. Sherman, C., Philadelphia.

Pearson, R., editor. 1969. Excavations at Lapakahi, N. Kohala, Hawaii Island - 1968. Division of State Parks, State Archaeological Journal 69-2, Honolulu.

Pearson, R., J. Hirata, L. Potts, and F. Harby. 1974. Test pitting of Cave 1, Kalaupapa Peninsula, Molokai, Hawaii. New Zealand Archaeological Society Newsletter 17:44-49.

Pearson, R. J., P. V. Kirch, and M. Pietrusewsky. 1971. An early prehistoric site at Bellows Beach, Waimanalo, Oahu, Hawaiian Islands. Archaeology and Physical Anthropology in Oceania 6:204-234.

Pearson, R. J., and SSRI 1969. Archaeology on the Island of Hawaii. Social Science Research Institute (SSRI), University of Hawai‘i.

Peterson, J. A., and M. K. Orr. 2004. Draft: IOno Ke Kole, Ia Ono Ke Kole–Sweet Conversation, Sweeet-tasting Fish: A Marine Ethnography of Kaloko-Honokohau National Historic Park, Kailua-Kona, Hawaii. International Archaeological Research Institute Inc., Honolulu.

Pfeffer, M. T. 2000. Implications of new studies of Hawaiian fishhook variability for our understanding of Polynesian settlement history. Pages 165-181 in T. D. Hurt, and G. F. M. Rakita, editors. Style and Function: Conceptual Issues in Evolutionary Archaeology. Greenwood Publishing Group, Santa Barbara, CA, USA.

Pitcher, C. R., T. D. Skewes, D. M. Dennis, and J. H. Prescott. 1992. Distribution of seagrasses, substratum types and epibenthic macrobiota in Torres Strait, with notes on pearl oyster abundance. Australian Journal of Marine and Freshwater Research 43:409-419.

Polovina, J. J. 1984. Model of a coral reef ecosystem. I. The ECOPATH model and its application to French Frigate Shoals. Coral Reefs 3:1-11.

Polovina, J. J. 1993. The lobster and shrimp fisheries in Hawaii. Marine Fisheries Review 55:28-33.

Pooley, S. G. 1993a. Economics and Hawaii's marine fisheries. Marine Fisheries Review 55:93-101.

Pooley, S. G. 1993b. Hawaii's marine fisheries: Some history, long-term trends, and recent developments. Marine Fisheries Review 55:7-20.

Portlock, N. and G. Dixon. 1789. A voyage around the world... in 1785-1788, in King George and Queen Charlott. Stockdale & Goulding, London.

Prange, G. W., M. Chibaya, and R. Barde. 1972. Miracle at Midway. Readers Digest, November 1972, Pleasantville.

Precht, W. F. 1994. The use of the term guild in coral reef ecology and paleoecology: a critical evaluation. Coral Reefs 13:135-136.

Presidential Proclamation 2416. Renaming the Hawaiian Islands Reservation as the Hawaiian Islands National Wildlife Refuge. July 25, 1940, Washington, DC.

Presidential Proclamation 8031. Establishment of the Northwestern Hawaiian Islands Marine National Monument. 71 Fed. Reg. 36443, June 26, 2006, Washington, DC.

Presidential Proclamation 8112. Amending Proclamation 8031 of June 15, 2006, To Read, “Establishment of the Papahānaumokuākea Marine National Monument.” 72 Fed. Reg. 10031, February 28, 2007, Washington, DC.

Pukui, M. K., and S. H. Elbert 1986. Hawaiian dictionary: Hawaiian–English and English–Hawaiian. University of Hawai‘i Press, Honolulu.

Pukui, M. K., S. H. Elbert, and E. T. Mo‘okini 1974. Place names of Hawai‘i. University of Hawai‘i Press, Honolulu.

Quimper Benitez del Pino, M. 1822. Islas de Sandwich: descripcion sucinta de este archipiélago, nombre que les dió su célebre descubridor, el Capitan Cook / reconocidas por el teniente de fragata de la Armada nacional D. Manuel Quimper Benitez del Pino. E. Aguado, Madrid.

Ragen, T. J. 1999. Human activities affecting the population trends of the Hawaiian monk seal. Pages 183-194 in J. A. Musick, editor. Life in the Slow Lane: Ecology and Conservation of Long-lived Marine Animals. American Fisheries Society Symposium, Bethesda, MD.

Ragen, T. J., and D. M. Lavigne. 1999. The Hawaiian monk seal: biology of an endangered species. Pages 224–245 in J. R. Twiss Jr., and R. R. Reeves, editors. Conservation and management of marine mammals. Smithsonian Institution Press, Washington, DC.

Ralston, C. 1978. Grass huts and warehouses: Pacific beach communities in the nineteenth century. University of Hawai‘i Press, Honolulu.

Ralston, C. 1984. Hawaii 1778-1854: some aspects of maka‘ainana response to rapid cultural change. The Journal of Pacific History 19:21-40.

Randall, J. E. 1998. Zoogeography of shore fishes of the Indo-Pacific region. Zoological Studies 37:227-268.

Randall, J. E. 2008. Reef and Shore Fishes of the Hawaiian Islands. University of Hawai‘i Sea Grant College Program, Honolulu.

Randall, J. E., and P. C. Heemstra. 1991. Revision of Indo-Pacific groupers (Perciformes: Serranidae: Epinephelinae), with descriptions of five new species. Indo-Pacific Fishes 20:1-332.

Rathbun, M. J. 1906. The Brachyura and Macrura of the Hawaiian Islands. Pages 827-930. The aquatic resources of the Hawaiian Islands. Bulletin of the United States fish commission. Vol. 23, for 1903. Part 3. Government Printing Office, Washington.

Rauzon, M. J. 2001. Isles of Refuge: Wildlife and History of the Northwestern Hawaiian Islands. University of Hawai‘i Press, Honolulu.

Read, A. J., and P. R. Wade. 2000. Status of marine mammals in the United States. Conservation Biology 14:929-940.

Read, G. H. 1912. The last cruise of the Saginaw. Houghton Mifflin Company, The Riverside Press Cambridge, Boston and New York.

ReefBase. 2007, [online] www.reefbase.org.

Remy, J. 1859. Récits d’un vieux sauvage pour servir a l’histoire ancienne de Havaii. Notes d’un voyageur lues à la Société d’agriculture, commerce, sciences et arts du département de la Marne, dans la séance du 15 décembre. E. Laurent, Chalons-sur-Marne.

Remy, J. 1979. Contributions of a venerable native to the ancient history of the Hawaiian Islands. translated from the French by William T. Brigham. Outbooks [reprint of 1874 publication], Reno.

Research and Economic Analysis Division. 1995. The changing structure of Hawaii's economy. State of Hawaii Dept. of Business, Economic Development and Tourism, Honolulu, HI.

Restarick, H. B. 1930. The discovery of Hawaii. Gaetano did not discover Hawaii, nor did the Spaniards know of the existence of the Hawaiian Islands before Captain James Cook discovered them in 1778. Unpublished manuscript, Honolulu.

Reynolds, J. N. 1835. Voyage of the United States Frigate Potomac Under the Command of Commodore John Downes, During the Circumnavigation of the Globe, in the Years 1831, 1832, 1833, and 1834 . . . Harper and Brothers, New York.

Rick Gaffney and Associates Inc. 2000. Evaluation of the status of the recreational fishery for ulua in Hawai‘i, and recommendations for future management. Department of Land and Natural Resources, Division of Aquatic Resources, Technical Report 20-02, Honolulu.

Rick, T. C., J. M. Erlandson, R. L. Vellanoweth, and T. J. Braje. 2005. From Pleistocene mariners to complex hunter-gatherers: The archaeology of the California Channel Islands. Journal of World Prehistory 19:169-228.

Rick, T. C., and J. M. Erlandson, editors. 2008. Human impacts on ancient marine ecosystems: A global perspective. University of California Press, Berkeley.

Rick, T. C., P. L. Walker, L. M. Willis, A. C. Noah, J. M. Erlandson, R. L. Vellanoweth, T. J. Braje, and D. J. Kennett. 2008. Dogs, humans and island ecosystems: the distribution, antiquity and ecology of domestic dogs (Canis familiaris) on California's Channel Islands, USA. Holocene 18:1077-1087.

Rick, T. C., R. L. DeLong, J. M. Erlandson, T. J. Braje, T. L. Jones, D. J. Kennett, T. A. Wake, and P. L. Walker. 2009. A trans-Holocene archaeological record of Guadalupe fur seals (Arctocephalus townsendi) on the California coast. Marine Mammal Science 25:487-502.

Rick, T. C., and J. M. Erlandson. 2009. Coastal exploitation. Science 325:952-953.

Roberts, C. 2007. The Unnatural History of the Sea. Island Press, Washington, D.C.

Roberts, C. M., and J. P. Hawkins. 2000. Fully-protected marine reserves: A guide. World Wildlife Federation, Endangered Seas Campaign, Washington, DC.

Robinson, G. 2009. A Tragedy of Democracy: Japanese Confinement in North America. Columbia University Press, New York.

Roblet, C. 1791-1792. Extrait du journal du voyage autour du monde du navire le Solide de Marseille, Capt. le Marchand. Manuscript, Archives nationales, Paris.

Rodgers, K. S., P. L. Jokiel, C. E. Bird, and E. K. Brown. 2009. Quantifying the condition of Hawaiian coral reefs. Aquatic Conservation: Marine and Freshwater Ecosystems 20:93-105.

Rolett, B. V. 1990. Archaeological excavations at site 50–80–15–3300 (Bellows Air Force Station, Oahu) conducted by the University of Hawai‘i archaeological field school. Typescript in State Historic Preservation Division Library, Kapolei.

Root, R. B. 1967. The niche exploitation pattern of the blue-grey gnatcatcher. Ecological Monographs 37:317-350.

Rosenberg, A. A. 2003. Managing to the margins: the overexploitation of fisheries. Frontiers in Ecology and the Environment 1:102-106.

Rosenberg, A. A., W. J. Bolster, K. E. Alexander, W. B. Leavenworth, A. B. Cooper, and M. G. McKenzie. 2005. The history of ocean resources: modeling cod biomass using historical records. Frontiers in Ecology and the Environment 3:84-90.

Rosenberg, A. A., M. J. Fogarty, M. P. Sissenwine, J. R. Beddington, and J. G. Shepherd. 1993. Achieving sustainable use of renewable resources. Science 262:828-829.

Rosendahl, P. H. 1972. Aboriginal agriculture and residence patterns in upland Lapakahi, Island of Hawaii. PhD Dissertation, University of Hawai‘i, Honolulu.

Rosendahl, P. H. 1994. Aboriginal Hawaiian structural remains and settlement patterns in the upland archeological zone at Lapakahi, Island of Hawaii. Hawaiian Archaeology 3:14-70.

Rosendahl, P. H., and D. E. Yen. 1971. Fossil sweet potato remains from Hawaii. Journal of the Polynesian Society 80:379-385.

Rothschild, L. W. 1893. The avifauna of Laysan and the neighbouring islands, with a complete history to date of the birds of the Hawaiian possessions. R.H. Porter, London.

Ruddle, K. 1996. Traditional management of reef fishing in N. V. C. Polunin, and C. M. Roberts, editors. Reef Fisheries. Chapman & Hall, London.

Ruddle, K., and R. E. Johannes, editors. 1985. The traditional knowledge and management of coastal systems in Asia and the Pacific. UNESCO, Indonesia.

Ruffino, L., K. Bourgeois, E. Vidal, C. Duhem, M. Paracuellos, F. Escribano, P. Sposimo, N. Baccetti, M. Pascal, and D. Oro. 2009. Invasive rats and seabirds after 2,000 years of an unwanted coexistence on Mediterranean islands. Biological Invasions 11:1631-1651.

Ruschenberger, W. S. W. 1838. Narrative of a voyage round the world, during the years 1835, 36, and 37. Richard Bentley, New Burlington-Street, Philadelphia.

Russell, D. J., and G. H. Balazs. 2000. Identification manual for dietary vegetation of the Hawaiian green turtle Chelonia mydas. U.S. Dept. of Commerce, NOAA Tech. Memo. NOAA-TM-NMFS-SWFSC-294. 49 pgs.

Russell, D. J., and G. H. Balazs. 2009. Dietary shifts by green turtles (Chelonia mydas) in the Kane‘ohe Bay region of the Hawaiian Islands: a 28-year study. Pacific Science 63:181-192.

Russell, D. J., G. H. Balazs, R. C. Phillips, and A. K. H. Kam. 2003. Discovery of the sea grass Halophila decipiens (Hydrocharitaceae) in the diet of the Hawaiian green turtle, Chelonia mydas. Pacific Science 57:393-397.

Saeki, L. A. 2005. Hawaiian Fishpond Bibliography. Pacific Regional Aquaculture Information Service for Education (PRAISE), Honolulu.

Sahlins, M. D. 1981. Historical metaphors and mythical realities: structure in the early history of the Sandwich Islands kingdom. ASAO Special Publications No. 1. University of Michigan Press, Ann Arbor.

Schilt, R., editor. 1984. Subsistence and conflict in Kona, Hawaii: An archaeological study of the Kuakini Highway realignment corridor. Hawaii Historic Preservation Report 84-1. Department of Anthropology, Bernice P. Bishop Museum, Honolulu.

Schlemmer, M. 1915. Log of the yacht Helene. Unpublished manuscript.

Schlemmer, M., and E. Schlemmer. 1904. Schlemmer Family Papers 1904-1924. Special Collections in the Smithsonian Institution Archives, Accession 05-288, Washington, DC.

Schmitt, R. C. 1968. Demographic statistics of Hawaii, 1778-1965. University of Hawai‘i Press, Honolulu.

Schmitt, R. C. 1977. Historical statistics of Hawaii. University of Hawai‘i Press, Honolulu.

Schoeffel, P. 1992. Food, health and development in the Pacific Islands: Policy implications for Micronesia. ISLA: A Journal of Micronesian Studies 1:223-250.

Schug, D. M. 2001. Hawai‘i’s commercial fishing industry: 1820-1945. The Hawaiian Journal of History 35:15-34.

Schultz, J. K., J. D. Baker, R. J. Toonen, and B. W. Bowen. 2008. Extremely low genetic diversity in the endangered Hawaiian monk seal (Monachus schauinslandi). Journal of Heredity 100:25-33.

Schultz, J. K., A. Marshall, and M. Pfunder. 2010. Genome-wide loss of diversity in the critically endangered Hawaiian monk seal. Diversity: In press.

Seaborn, G. T., M. Katherine Moore, and G. H. Balazs. 2005. Depot fatty acid composition in immature green turtles (Chelonia mydas) residing at two near-shore foraging areas in the Hawaiian Islands. Comparative Biochemistry and Physiology Part B: Biochemistry and Molecular Biology 140:183-195.

Seaton, S. 1974. The Hawaiian "kapu" abolition of 1819. American Ethnologist 1:193-206.

Setchell, W. A. 1905. Limu. University of California Publication Botany 2:91-113.

Sharp, D., editor. 1913. Fauna hawaiiensis, Cambridge.

Shauinsland, H. H., and M. D. F. Udvardy. 1996. Three months on a coral island (Laysan), by Hugo H. Schainsland [1899]. Atoll Research Bulletin 432.

[online] http://www.botany.hawaii.edu/faculty/duffy/atoll.htm

Sheppard, C. 1995. The shifting baseline syndrome. Marine Pollution Bulletin 30:766-777.

Shinsato, H. 1973. Letter dated May 9, 1973 to the Regional Director, U.S. Bureau of Sport Fisheries and Wildlife, Portland, Oregon.

Shomura, R. 2004. A historical perspective of Hawaii's marine resources, fisheries, and management issues over the past 100 years. Pages 6-11 in A. M. Friedlander, editor. Status of Hawaii's coastal fisheries in the new millennium. Proceedings of the 2001 fisheries symposium sponsored by the American Fisheries Society, Hawaii Chapter. Hawaii Audubon Society, Honolulu.

Shomura, R. S. 1987. Hawaii's marine fishery resources: yesterday (1900) and today (1986). Southwest Fisheries Center Administrative Report H-87-2. Southwest Fisheries Center, National Marine Fisheries Service, Honolulu Laboratory, Honolulu.

Sims, N. A. 1993. Pearl oysters. Pages 409-430 in A. Wright, and L. Hill, editors. Nearshore marine resources of the South Pacific: information for fisheries development and management. Forum Fisheries Agency, Institute of Pacific Studies, printed by Singapore National Printers, Honiara, Solomon Islands and Suva, Fiji.

Sinoto, A. 1978. Archaeological and Paleontological Salvage at Barbers Point, Oahu. Bernice P Bishop Museum and University of Hawai‘i Department of Anthropology, Honolulu, Hawaii, USA.

Sinoto, Y. H. 1983. An analysis of Polynesian migrations based on the archaeological assessments. Journal de la Societe des Oceanistes 39:57-67.

Sinoto, Y. H. 1995. The development and distribution of fishing gear in the Pacific: typology and distribution of fishhooks in Polynesia. Pages 143-164. Taiwan Museum.

Sinoto, Y. H., and M. Kelly 1975. Archaeological and Historical Survey of Pakini-Nui and Pakini-Iki Coastal Sites: Waiahukini, Kailikii, and Hawea, Ka'u, Hawaii. Dept. of Anthropology, Bernice P. Bishop Museum, Honolulu, HI.

Sissenwine, M. P., and A. A. Rosenberg. 1993a. Marine fisheries at a critical juncture. Fisheries 18:6-14.

Sissenwine, M. P., and A. A. Rosenberg. 1993b. United-States fisheries - status, long-term potential yields, and stock management ideas. Oceanus 36:48-54.

Skillings, D. 2009. Personal communication to J. N. Kittinger. University of Hawai‘i at Mānoa, Honolulu.

Smith, E. A. 1992. Human behavioral ecology: I. Evolutionary Anthropology 1:20-25.

Smith, I. W. G. 1979. Prehistoric sea mammal hunting in Palliser Bay. Pages 215–224 in B. F. Leach, &, and H. McLean, editors. Prehistoric man in Palliser Bay. Museum of New Zealand, Bulletin of the National Museum of New Zealand 21, Wellington, New Zealand.

Smith, I. W. G. 1985. Sea mammal hunting and prehistoric subsistence in New Zealand. PhD Dissertation, University of Otago.

Smith, I. W. G. 1989. Maori impact on the marine megafauna: pre-European distributions of New Zealand sea mammals. Pages 76–108 in D. G. Sutton, editor. Saying so doesn’t make it so. Papers in honour of B. Foss Leach. New Zealand Archaeological Association Monograph 17, Dunedin, New Zealand.

Smith, I. W. G. 2004. Nutritional perspectives on prehistoric marine fishing in New Zealand. New Zealand Journal of Archaeology 24:5-31.

Smith, I. W. G. 2005. Retreat and resilience: fur seals and human settlement in New Zealand. Pages 6-18 in G. Monks, editor. The Exploitation and Cultural Importance of Sea Mammals. Oxbow Books, Oxford.

Smith, J. E. 2003. Factors influencing algal blooms on tropical reefs with an emphasis on herbivory, nutrients and invasive species. PhD Dissertation, Dept. of Botany, University of Hawai‘i at Mānoa, Honolulu.

Smith, J. E., C. L. Hunter, and C. M. Smith. 2002. Distribution and reproductive characteristics of nonindigenous and invasive marine algae in the Hawaiian Islands. Pacific Science 56:299-316.

Smith, M. K. 1993. An ecological perspective on inshore fisheries in the main Hawaiian Islands. Marine Fisheries Review 55:34-49.

Smythe, W. R. 1960. Monk seals on Laysan Island. The Elepaio 20:78-79.

Soehren, L. S. n.d. Archaeological survey and excavations: sites K2, K3, K4, K5 Nu‘alolo Kai, Na Pali, Kauai in W. K. Kikuchi, editor. Manuscript on file, Bernice P. Bishop Museum, Honolulu.

SRGII. 2004. Working Documents: Fishing in the proposed northwestern Hawaiian Islands National Marine Sanctuary. Prepared for U.S. Department of Commerce, National Oceanographic and Atmospheric Administration, National Marine Sanctuary Program, Northwestern Hawaiian Islands Coral Reef Ecosystem Reserve. Sustainable Resources Group Intn’l, Inc. (SRGII), Honolulu, HI.

Stannard, D. E. 1989. Before the horror: the population of Hawai‘i on the eve of Western contact. Social Science Research Institute, University of Hawai‘i, Honolulu.

State of Hawaii. 1948-2006. Commercial Fish Landings, semi-annual and annual reports and records. State of Hawaii, Department of Land and Natural Resources, Division of Fish and Game, Honolulu.

State of Hawai‘i, National Oceanic and Atmospheric Administration, Office of Hawaiian Affairs, and U.S. Fish and Wildlife Service. 2009. Nomination of Papahānaumokuākea Marine National Monument for Inscription on the World Heritage List. National Oceanic and Atmospheric Administration, Honolulu, Hawai‘i. 280 pgs.

State of Hawaii. n.d. History of Oahu's Harbors. State of Hawaii, Dept of Transportation, Harbors Division.

Steadman, D. W. 1995. Prehistoric extinctions of Pacific Island birds: Biodiversity meets zooarchaeology. Science 267:1123-1131.

Stevens, J. D., R. Bonfil, N. K. Dulvy, and P. A. Walker. 2000. The effects of fishing on sharks, rays, and chimaeras (chondrichthyans), and the implications for marine ecosystems. Journal of Marine Science 57:476-494.

Stewart, C. S. 1828. Journal of a residence in the Sandwich islands during... 1823, 1824, and 1825, with notes by W. Ellis. H. Fisher, Son, & P. Jackson, Newgate Street, London.

Stimson, J. Stimson, Larned, S. Larned, Conklin, and E. Conklin. 2001. Effects of herbivory, nutrient levels, and introduced algae on the distribution and abundance of the invasive macroalga Dictyosphaeria cavernosa in Kaneohe Bay, Hawaii. Coral Reefs 19:343-357.

Stokes, J. F. G. 1908. Walled fish traps of Pearl Harbor. Occasional Papers of Bernice P. Bishop Museum 4:199-212.

Stokes, J. F. G. 1920. Fish-poisoning in the Hawaiian Islands. Bernice P. Bishop Museum, Occasional Papers 7:217-234.

Stokes, J. F. G. 1932. Spaniards and the sweet potato in Hawaii and Hawaiian-American contacts. American Anthropologist 34:594-600.

Stokes, J. F. G. 1978. Iron with the early Hawaiians. Kraus Reprint Co. [Papers of the Hawaiian Historical Society, No. 18], Millwood, NY.

Stokes, J. F. G., and T. Dye. 1991. Heiau of the Island of Hawaii: a historic survey of native Hawaiian temple sites. Bishop Museum Press, Honolulu.

Stokstad, E. 2005. Coral ages show Hawaiian temples sprang from political revolution. Science 307:25.

Storm, B. 1940. Commercial fishing in Hawaii. Pages 14-18. Pan-Pacific, Vol. 4, No.2, Honolulu.

Summers, C. C. 1964. Hawaiian Fishponds. Bernice P. Bishop Museum Special Publication 52, Honolulu.

Summers, C. C. 1971. Moloka‘i: A site survey. Bernice P. Bishop Museum, Pacific Anthropological Records 14, Honolulu.

Svihla, A. 1936. The Hawaiian rat. Reprinted from The Murrelet for January, 1936, Vol. 17.

Svihla, A. 1957a. Dental caries in the Hawaiian dog. Occasional Papers of Bernice P. Bishop Museum 22:7-13.

Svihla, A. 1957b. Observations on French Frigate Shoals, February 1956. Atoll Research Bulletin 51. [online] http://www.botany.hawaii.edu/faculty/duffy/atoll.htm

Tainter, J. A. 1976. Spatial organisation and social patterning in the Kaloko Cemetery, North Kona, Hawaii. Archaeology and Physical Anthropology in Oceania 11:91-105.

Tainter, J. A., and R. H. Cordy. 1977. An archaeological analysis of social ranking and residence groups in prehistoric Hawaii. World Archaeology 9:95-112.

Takeguchi, A., J. Hollyer, W. Koga, M. Hakoda, K. Rohrbach, H. C. S. Bittenbender, B. Buckley, J. B. Friday, R. Bowen, R. Manshardt, J. Leary, G. Teves, E. Herring, H. Zaleski, K. Leonhardt, and B. Eger. 1999. Some history of Hawaii agriculture. Hawaii's Agriculture Gateway, Honolulu, HI.

Tamarin, R. H., and S. R. Malecha. 1972. Reproductive parameters in Rattus rattus and Rattus exulans of Hawaii, 1968 to 1970. Journal of Mammalogy 53:513-528.

Tamaru, C. S., C. Helsley, and C. Carlstrom-Trick. 1997. Aquaculture in Hawai‘i - past, present and future. Pages 257-278 in C. Helsley, editor. Open Ocean Aquaculture '97, Charting the Future of Ocean Farming. University of Hawai‘i Sea Grant College Program, Maui, Hawaii.

Tanaka, W. 2008. Ho‘ohana aku, Ho‘ola aku: first steps to averting the tragedy of the commons in Hawai‘i’s nearshore fisheries. Asian-Pacific Law & Policy Journal 10:235-291.

Taylor, R. H., K. J. Barton, P. R. Wilson, B. W. Thomas, and B. J. Karl. 1995. Population status and breeding of New Zealand fur seals (Arctocephalus forsteri) in the Nelson-northern Marlborough region, 1991-94. New Zealand journal of marine and freshwater research 29:223-234.

Teggart, F. J., editor. 1924. Around the horn to the Sandwich Islands and California 1845-1850, being a personal record kept by Chester S. Lyman. Yale University Press, New Haven.

Thomas, N. 1991. Entangled Objects: Exchange, Material Culture, and Colonialism in the Pacific. Harvard University Press, Cambridge, MA.

Thorsen, M., R. Shorten, R. Lucking, and V. Lucking. 2000. Norway rats (Rattus norvegicus) on Frégate Island, Seychelles: the invasion; subsequent eradication attempts and implications for the island's fauna. Biological Conservation 96:133-138.

Thrum, T. G. 1904. A Historic Tortoise or Land Turtle. Hawaiian Almanac and Annual for 1904. 72 pgs.

Thrum, T. G. 1907. Hawaiian Almanac and Annual for 1908. The Reference Book of Information and Statistics relating to the Territory of Hawaii, of Value to Merchants, Tourists and Others. Thos G. Thrum, Honolulu.

Thrum, T. G. 1925. Hawaiian Almanac and Annual 1925. Thos G. Thrum, Honolulu.

Thurston, L., editor. 1904. The fundamental law of Hawaii. Hawaiian Gazette Company, Honolulu.

Thurston, L. 1925. The fish question in Hawaii. Honolulu Advertiser, article series published July 26, Aug. 2, 9, 16, 23, 30; Sept. 6, 13 on pg. 1, Honolulu.

Thurston, L. 1927. Honolulu faces fish fight to a finish; Fish Trust is arrogant! The Honolulu Advertiser, 29 May 1927, Honolulu.

Tilburg, H. v. 1999. Hawaiian historic shipwrecks: relative positions. Independent Report, Graduate Maritime Archaeology and History Certificate program, Marine Option Program, School of Ocean and Earth Science and Technology, University of Hawai‘i at Mānoa, Honolulu.

Tilden, J. E. 1902. Algae collecting in the Hawaiian Islands. Pages 135-175. Postelsia, The Year book of the Minnesota Seaside Station, 1901, St. Paul, Minnesota.

Titcomb, M. 1972. Native Use of Fish in Hawaii. University of Hawai‘i Press, Honolulu.

Titcomb, M. 1978. Native use of marine invertebrates in old Hawaii. Pacific Science 32:325-377.

Titcomb, M., and M. K. Pukui 1969. Dog and man in the ancient Pacific, with special attention to Hawaii. Bernice P. Bishop Museum Special Publication 59. Printed by Star-Bulletin Print. Co., Honolulu.

Tomich, P. Q. 1969. Mammals in Hawaii: A Synopsis and Notational Bibliography. Bishop Museum Press, Honolulu, HI.

Tomonari-Tuggle, M. J., H. D. Tuggle, and J. S. Athens. 2000. Archaeology on a south coast landscape: Hulopo‘e, Lāna‘i, Hawai‘i. Volumes I-III. Report prepared for Lanai Company, Inc. International Archaeological Research Institute, Inc., Honolulu.

Tsuda, R. T. 1965. Marine algae from Laysan Island with additional notes on the vascular flora. Atoll Research Bulletin 110. [online] http://www.botany.hawaii.edu/faculty/duffy/atoll.htm

Tsuda, R. T. 1966. Marine benthic algae from the leeward Hawaiian group. Atoll Research Bulletin 115. [online] http://www.botany.hawaii.edu/faculty/duffy/atoll.htm

Tsuha, K. 2008. Draft report: Analysis and study of significant alignments on Kaua‘i, Ha‘ena ma (Mokumanamana), Nihoa, Ka‘ula, Ni‘ihau, & Lehua, June 21-29, 2007. Prepared for: Dr. Pualani Kanahele, Ha‘ae Wale Hanauna Lolo, Wailuku.

Tuggle, H. D., R. Cordy, and M. Child. 1978. Volcanic glass hydration-rind age determination for Bellows Dune, Hawaii. New Zealand Archaeological Society Newsletter 21:57-77.

Tuggle, H. D., and P. B. Griffin, editors. 1973. Lapakahi Hawaii: archaeological studies. Asian and Pacific Archaeology Series No. 5, Social Science Research Institute, University of Hawai‘i, Honolulu.

Tuggle, H. D., and L. Olson. 1978. A review of "hydration dating" of Hawaiian volcanic glass. Historic Preservation Division, Department of Land and Natural Resources, State of Hawaii, Honolulu.

Tuggle, H. D., and M. Spriggs. 2000. The age of the Bellows Dune site O18, O‘ahu, Hawai‘i, and the antiquity of Hawaiian colonization. Asian Perspectives 39:165-188.

Tuggle, H. D., and M. J. Tomonari-Tuggle. 1980. Prehistoric agriculture in Kohala, Hawaii. Journal of Field Archaeology 7:297-312.

Turner, A. 1989. Sample selection, schlepp effects and scavenging: the implications of partial recovery for interpretations of the terrestrial mammal assemblage from Klasies River Mouth. Journal of Archaeological Science 16:1-11.

Twain, M. 1866. The Whaling Trade. Sacramento Daily Union, Sacramento, CA. [online] http://www.twainquotes.com/sduindex.html

Twain, M. 1872. Roughing it. Signet Classics.

Uchida, R. N. 1976. Reevaluation of fishing effort and apparent abundance in the Hawaiian fishery for skipjack tuna, Katusuwonus pelamis, 1948-70. Fishery Bulletin 74:59-69.

Udvardy, M. D. F., and R. E. Warner. 1964. Observations on the birds of French Frigate Shoal and Kure Atoll. Atoll Research Bulletin 103. [online] http://www.botany.hawaii.edu/faculty/duffy/atoll.htm

Unger, T. E. 2003. Max Schlemmer: Hawaii’s king of Laysan Island. IUniverse, Inc., Lincoln, NE

U.S. Census Bureau. 2010. National Census Data. [online] http://www.census.gov/

United States Dept. of the Treasury. 1898. The fur seals and fur-seal islands of the North Pacific ocean. pt. I. The history, condition, and needs of the herd of fur seals resorting to the Pribilof islands, by D. S. Jordan and G. A. Clark.--pt. II. Observations on the fur seals of the Pribilof islands, 1872-1897.--pt. III. Special papers relating to the fur seal and to the natural history of the Pribilof islands.--pt. IV. The Asiatic fur-seal islands and fur-seal industry, by Leonhard Stejneger. Govt. Print. Office, Washington, DC.

Van Houtan, K. S., and O. L. Bass. 2007. Stormy oceans are associated with declines in sea turtle hatching. Current Biology 17:R590-R591.

Van Houtan, K. S., S. K. Hargrove, and G. H. Balazs. 2010. Land use, macroalgae, and a tumor-forming disease in marine turtles. PLoS ONE 5:e12900.

Van Tilburg, H., and K. Kikiloi. 2007. Papahānaumokuākea Marine National Monument. Pages 56-59 in B. G. Terrell, editor. Fathoming Our Past: Historical Contexts of the National Marine Sanctuaries. National Oceanic and Atmospheric Administration, Silver Spring, MD.

Vancouver, G. 1798. Views of the Sandwich and other islands. J. Edwards & G. Robinson, London.

Vancouver, G. 1801. A voyage of discovery to the North Pacific ocean, and round the world… in the years 1790, 1791, 1792, 1793, 1794, and 1795, in the Discovery sloop of war, and armed tender Chatham, under the command of Captain George Vancouver. Vol I-IV. Printed for John Stockdale, London.

Vitousek, P. M. 2002. Oceanic islands as model systems for ecological studies. Journal of Biogeography 29:573-582.

Vitousek, P. M., T. N. Ladefoged, P. V. Kirch, A. S. Hartshorn, M. W. Graves, S. C. Hotchkiss, S. Tuljapurkar, and O. A. Chadwick. 2004. Soils, agriculture, and society in precontact Hawaii. Science 304:1665-1669.

Vitousek, P. 2006. Ecosystem science and human-environment interactions in the Hawaiian archipelago. Journal of Ecology 94:510-521.

WPRFMC. N.D. History of the fisheries in the northwestern Hawaiian Islands. Western Pacific Regional Fishery Management Council (WPRFMC), Honolulu.

Wagner, W. L., D. R. Herbst, and S. H. Sohmer 1990. Manual of the Flowering Plants of Hawaii. Bishop Museum Press, Honolulu.

Walker, F. D. 1909. Log of the Kaalokai. The Hawaiian Gazette Co., Ltd., Honolulu.

Walker, F. D. 1913. Experiences of Capt. F.D. Walker, crew and family, wrecked on Midway Island 1889. Paradise of the Pacific, Jan. 1913, p.18, Honolulu.

Walker, P. L., D. J. Kennett, T. L. Jones, and R. DeLong. 2002. Archaeological investigations at the Point Bennett pinniped rookery on San Miguel Island. Pages 628–632 in D. Browne, K. Mitchell, and H. Chaney, editors. Proceedings of the Fifth California Islands Symposium, March 29 - April 1, 1999. Santa Barbara Museum of Natural History, Santa Barbara, CA.

Ward, R. G. 1972. The Pacific bêche-de-mer trade with special reference to Fiji. Man in the Pacific Islands: Essays on Geographical Change in the Pacific Islands.

Warren, S. D. 2004. Degradation and recovery of vegetation on Kaho‘olawe Island, Hawai‘i: a photographic journey. Pacific Science 58:461-495.

Waterhouse, H. 1899. Hawaii deep-sea fishing off Kona. Page 104. All About Hawaii, 1899, v.25 p.104-106, Honolulu.

Webber, J. 1785. Reproductions of prints by John Webber from Captain James Cook’s Voyage to Hawaii, 1778-1779.

Webber, J. 1808. Views in the South seas, from drawings by the late James Webber [sic] draftsman on board the Resolution, Captain James Cooke, from the year 1776 to 1780. With letterpress, descriptive of the various scenery, etc. Boydell and Company, London.

Weisler, M. I., K. D. Collerson, Y. X. Feng, J. X. Zhao, and K. F. Yu. 2006. Thorium-230 coral chronology of a late prehistoric Hawaiian chiefdom. Journal of Archaeological Science 33:273-282.

Westervelt, W. D. 1998. Hawaiian historical legends. Mutual Publishing, Honolulu.

Wetherbee, B. M., C. G. Lowe, and G. L. Crow. 1994. History of shark control in Hawaii: with recommendations for future research. Pacific Science 48:95-115.

Wetmore, A. 1925. Bird life among lava rock and coral sand. National Geographic Magazine 48:77-108.

Wiesler, M. I. 1989. Chronometric dating and the late Holocene prehistory in the Hawaiian Islands: a critical review of radiocarbon dates from Moloka‘i Island. Radiocarbon 31:121-145.

Wilcox, B., K. Duin, J. Shafer, and D. Shafer. 2004. Results of the fishing discussion group process, Fall 2003. Fishing in the proposed Northwestern Hawaiian Islands National Marine Sanctuary. Prepared for U.S. Department of Commerce, National Oceanographic and Atmospheric Administration, National Marine Sanctuary Program, Northwestern Hawaiian Islands Coral Reef Ecosystem Reserve. Sustainable Resources Group Intn’l, Inc., Honolulu, HI.

Wilkes, C. 1852a. Narrative of the United States exploring expedition during the years 1838, 1839, 1840, 1841, 1842. Ingram, Cooke and Co., 227, Strand, London.

Wilkes, C. 1852b. Narrative of the United States exploring expedition during the years 1838, 1839, 1840, 1841, 1842. Sherman, C, Philadelphia, U.S.

Williams, I. D., W. J. Walsh, R. E. Schroeder, A. M. Friedlander, B. L. Richards, and K. A. Stamoulis. 2008. Assessing the importance of fishing impacts on Hawaiian coral reef fish assemblages along regional-scale human population gradients. Environmental Conservation 35:261-272.

Williams, S. H., and J. F. B. Marshall 1846. Report of the Proceedings and Evidence in the Arbitration Between the King and Government of the Hawaiian Islands and Messrs. Ladd & Co., Before Messrs. Stephen H. Williams & James F.B. Marshall, Arbitrators Under Compact 13th July, 1846 By Hawaii. Attorney General's Office, John Ricord, Stephen H. Williams, Office of the Attorney General, Hawaii, Ladd & Co, James F. B. Marshall. Charles E. Hitchcock, Printer, Government Press, Honolulu.

Wing, E. S. 2001. The sustainability of resources used by Native Americans on four Caribbean islands. International Journal of Osteoarchaeology 11:112-126.

Winterhalder, B. and E. A. Smith. 2000. Analyzing adaptive strategies: Human behavioral ecology at twenty-five. Evolutionary Anthropology 9:51-72.

Witzell, W. N. 1994. The origin, evolution, and demise of the U.S. sea turtle fisheries. Marine Fisheries Review 56:8-23.

Woodward, P. W. 1972. The natural history of Kure Atoll, Northwestern Hawaiian Islands. Atoll Research Bulletin 164. [online] http://www.botany.hawaii.edu/faculty/duffy/atoll.htm

Wyllie, R. C. 1845. Notes on the Sandwich, or Hawaiian Islands. Pages 253-268 in P. L. Simmonds, editor. Simmonds's Colonial Magazine and Foreign Miscellany, May-August 1845, Vol. V. Simmonds & Ward, London.

Wyllie, R. C. 1845. Notes on the Sandwich, or Hawaiian Islands. Pages 125-139; 316-328 in P. L. Simmonds, editor. Simmonds's Colonial Magazine and Foreign Miscellany, September-December 1845, Vol. VI. Simmonds & Ward, London.

Yukihira, H., D. W. Klumpp, and J. S. Lucas. 1999. Feeding adaptations of the pearl oysters Pinctada margaritifera and P. maxima to variations in natural particulates. Marine Ecology Progress Series 182:161-173.

Zeller, D., S. Booth, P. Craig, and D. Pauly. 2006. Reconstruction of coral reef fisheries catches in American Samoa, 1950–2002. Coral Reefs 25:144-152.

Zeller, D., S. Booth, and D. Pauly. 2005. Reconstruction of coral reef- and bottom fisheries catches for U.S. flag island areas in the western Pacific, 1950 to 2002. Western Pacific Regional Fishery Management Council, Honolulu, HI.

Zeller, D., S. Booth, G. Davis, and D. Pauly. 2007. Re-estimation of small-scale fishery catches for U.S. flag-associated island areas in the western Pacific: the last 50 years. Fishery Bulletin 105:266-277.

Zeller, D., S. Booth, and D. Pauly. 2007. Fisheries contribution to GDP: underestimating small-scale fisheries in the Pacific. Marine Resource Economics 21:355-374.

Ziegler, A. C. 1990. Search for evidence of early Hawaiian presence on Lisianski Island, Hawaiian Islands National Wildlife Refuge, summer 1990. Unpublished Report, State of Hawai‘i, Office of Hawaiian Affairs, Honolulu. 55 pgs.
