# Supplementary material for: Historical Reconstruction Reveals Recovery in Hawaiian Coral Reefs
Source: PLoS One. 2011 Oct 3;6(10):e25460. doi: 10.1371/journal.pone.0025460 (PMC3184997; doi:10.1371/journal.pone.0025460)
Supplement: Supporting Information S2 — Summary narratives of the primary data used to reconstruct ecological conditions and anthropogenic impacts are presented by guild. At the beginning of each guild subsection, tables summarize a timeline specific to the guild and include descriptions of major events, intensity of proximate stressors and the quantitative EcoState scores assigned to different time periods (Tables A–F). In the summary narratives that follow these tables, a chronological overview synthesizes primary data from multiple data sources and types. These syntheses by guild justify the quantitative scores determined for guild EcoStates and proximate stressor regimes. Ecological changes are reconstructed from multiple lines of evidence. Guild summary narratives are supplemented with additional data in the form of endnotes. In the narratives, terms are used to refer to specific periods in Hawaiian history; these include: 1) prehistoric: AD 1250–1778; 2) historic: AD 1778–1900; and, 3) modern: AD 1900+. (DOCX) [file pone.0025460.s014.docx]

Supporting Information S2: Summary narratives of the primary data used to reconstruct ecological conditions and anthropogenic impacts are presented by guild. At the beginning of each guild subsection, tables summarize a timeline specific to the guild and include descriptions of major events, intensity of proximate stressors and the quantitative EcoState scores assigned to different time periods (Tables S7-18). In the summary narratives that follow these tables, a chronological overview synthesizes primary data from multiple data sources and types. These synthesizes by guild justify the quantitative scores determined for guild EcoStates and proximate stressor regimes. Ecological changes are reconstructed from multiple lines of evidence. Guild summary narratives are supplemented with additional data in the form of endnotes. In the narratives, terms are used to refer to specific periods in Hawaiian history; these include: 1) prehistoric: AD 1250-1778; 2) historic: AD 1778-1900; and, 3) modern: AD 1900+.

1. Data Summaries by Ecological Guild

A. Reef Corals

B. Seagrasses/Algae

C. Suspension Feeders & Detritivores

D. Large Herbivores

E. Large Carnivores

F. Small Herbivores & Small Carnivores

2. Footnotes for Summary Narratives by Guild

Acknowledgments

Several expert reviewers provided comments on these sections of this supporting information, which greatly improved the clarity and content of the manuscript. We thank Cindy Hunter, Kimberly Maison, Bud Antonelis, Kyle Van Houten, Celia Smith and two anonymous reviewers for their expert knowledge and constructive input. Any errors belong to the authors alone.

**1. Data Summaries by Ecological Guild**

A. Reef Corals

A.1 Main Hawaiian Islands

Table A.1: Timeline of major events and proximate stressors (1-6) impacting the EcoState for corals in the Main Hawaiian Islands. Proximate stressors are indicated with regard to their intensity: x = low, X = medium, X [emboldened] = high.

| **Date (AD)** | **EcoState** | **Description** | **1. Overexploitation** | **2. Invasive Species** | **3. Land-Based Pollution** | **4. Disease** | **5. Climate Change** |
| --- | --- | --- | --- | --- | --- | --- | --- |
| <1250 | 1 | Pristine |  |  |  |  |  |
| 1250-1350 | 1.25 | Subsistence harvesting only for implements, religious offerings |  |  |  |  |  |
| 1350-1778 | 2 | Indigenous harvest of coral for fishpond construction; uplands modification | x |  | x |  |  |
| 1778-1815 | 1.5 | No major use; introduction of feral ungulates into uplands |  |  | x |  |  |
| 1815-1830 | 2.5 | Moderate harvesting for use in church & structure building in major towns | x |  | x |  |  |
| 1830-1885 | 3 | Large-scale commercial coral cutting for construction; dredge & fill of harbors & estuaries; sedimentation due to invasive species and human modification of uplands | **X** |  | X |  |  |
| 1885-1935 | 3.5 | Impacts increase from sedimentation due to large-scale upland agriculture and ranching |  |  | **X** |  |  |
| 1935-1980 | 3.5 | Land-based pollution impacts from development of coastal zones; major dredging activities |  |  | **X** |  |  |
| 1980-2009 | 2.5 | Clean Water Act - some improvements in sewage treatment; sedimentation; invasive species; coral disease, bleaching |  | x | x | x | x |

The first colonizing Polynesians in Hawai‘i used coral in ritual activities (e.g. ceremonial offerings) and for tools and implements such as abraders (Kirch 1982a). Archaeological and ethnographic evidence from the early prehistoric period suggests that coral was harvested at very low levels and therefore impacts to coral can be assumed to be very low, or negligible for the earliest prehistoric period in Hawai‘i. Starting in the 1300-1400s, there is evidence in the Hawaiian archipelago of a wave of construction of monumental architecture (e.g. large temples, or heiau) and large-scale fishponds for aquaculture (Burney & Burney 2003; Dixon et al. 1995; Kirch & Sharp 2005). While most of these features are constructed primarily of volcanic basalt, some coral was used in construction activities (Apple & Kikuchi 1975; Costa-Pierce 1987; Deering 1899; Stokes 1908). Records indicate that volcanic basalt was, however, the primary construction material for heiau, and oral traditions describe source areas for volcanic stone that was quarried for construction (Costa-Pierce 1987; Stokes 1908). Of the fishpond types developed in Hawai‘i, the loko kuapā, a type that extended over the reef flat, had the most impact on reef corals, as both coral and basalt were used in wall construction (Costa-Pierce 1987:326; Kikiloi 2003; Kikuchi 1976). Fishponds are believed to have been constructed prior to the 14^th^ century (Kikuchi 1976), and recent dating of fishponds on the island of Kaua‘i shows that construction primarily dates to the period AD 1305–1420 (Burney & Burney 2003). Additional impacts to coral during this late prehistoric period are evidenced in the ethnographic record, including some evidence of chiefs undertaking activities to deepen harbor channels (e.g. Fornander & Stokes 1880:Vol II:48, in Stokes 1908:206), and the archaeological record, which reveals some evidence of sedimentation in coral reef environments due to modification of uplands for agriculture and estuarine fishpond aquaculture.^[[1]](#endnote-1)^ Alteration of terrestrial habitat in the uplands through the introduction of invasive rats, forest clearing or burning may have resulted in sedimentation in the nearshore environment (Athens 1985, 1997, 2009; Culliney 2006; Kirch & Kahn 2007; Drake & Hunt 2008). The available evidence, however, suggests sedimentation impacts were primarily limited to alluvial valleys, estuarine environments and riparian systems in areas of high human population, but some sedimentation may have occurred in nearshore reef zones - more research is needed in this area (Kirch 1982b).

After Western contact (1778), impacts to coral remained negligible and are due to land-based pollution from uplands erosion due to the introduction of feral ungulates (e.g. cows, goats, sheep). In the early 1800s, however, coral began to be harvested as a construction material – both as a raw material for structure building and for burning for lime mortar. During this period, only westerners held in high regard by Hawaiian chiefs were allowed to build homes and own property (Beechert 1991:21). In 1816 the Hawaiian King, Kamehameha I, ordered the construction of the Honolulu Fort, which was built almost entirely from coral harvested from the Honolulu harbor area (Alexander 1899).^[[2]](#endnote-2)^ After the death of Kamehameha I and the subsequent abolishment of the indigenous religious system in 1819, chiefly restrictions on home building were relaxed. The 1820s witnessed a boom in the use of coral for construction in Honolulu, followed shortly thereafter in the other islands, as coral was harvested for use in homes, public buildings, and in the construction of churches by Protestant missionaries. The harvesting required considerable effort, which was largely put on the shoulders of the Native Hawaiians who worked for the Protestant missions and private companies. Hiram Bingham, a Protestant missionary, described in detail the intensive process of coral harvesting and processing for the construction a mission church at Kealakekua Bay, Hawai‘i Island.^[[3]](#endnote-3)^ This process was repeated throughout the archipelago wherever missionaries were stationed. According to a survey of early missionary churches by Gowans (1993), at least 69 individual churches were constructed using coral materials from 1828-1859, with the majority of them situated on Maui, O‘ahu, and Hawai‘i islands. In many cases, the lava stones from Hawaiian heiau were used in conjunction with mortar derived from coral to build the structures but some churches were built solely from coral.

By the late 1830s, commercial enterprises had become established to provide coral to a burgeoning construction industry in Honolulu. The widely held view of coral as a valuable construction material and evidence of large-scale use is described by Bates (1854).^[[4]](#endnote-4)^ By the late 1850s, the majority of homes in Honolulu were constructed with coral materials.^[[5]](#endnote-5)^ The use of coral was so prevalent that even family tombs and the walls surrounding cemeteries were built from coral. In 1857 the Honolulu fort was demolished in order to further develop the land and harbor of Honolulu, which resulted in significant dredging activities over the course of decades in the mid to late 1800s. Eventually, over sixteen acres of coral reef area in the Honolulu harbor area were filled to accommodate a 2000-foot waterfront area (Beechert 1991). The harbor was dredged to a depth of 20-25 feet, and the spoils used as fill for the waterfront (Alexander 1899). By 1860, this new area was finished and the royal residence, Iolani Palace, which before had been a few hundred yards from the ocean, was now several thousand feet from the water. The direct exploitation of coral for construction waned in the early 1900s due to the importation of cheaper and more desirable raw construction materials, but some harvesting continued for use in agricultural fertilizers (Thrum 1907). New impacts to reef corals arose with the commercial agriculture and ranching industries, which began to ramp up considerably in the 1860s. Various crops were introduced for commercialization, but sugar quickly became the focus. Large-scale alteration of upland areas from sugar plantations and ranching activities resulted in increased erosion, the deleterious effects of which were reported by early surveyors of the coral reef and coastal environment (Agassiz 1889; MacCaughey 1918c). The increased delivery of sediment impacted the corals in nearshore marine environments, but detailed surveys are rare, and early surveyors lacked a baseline by which to compare their observations. Despite this, the impacts of sedimentation are evident in historical accounts, including numerous reports of large-scale fishponds being filled in by sedimentation (Beechert 1991; Clifford 1991).

Foreign visitors, or westerners, also brought destructive fishing practices, including the use of bleach and dynamite, which were introduced in the 1870s (Cobb 1902:412; Clifford 1991; Kelly 1925:853). Anecdotal accounts suggest the use of dynamite was widespread by the late 1800s.^[[6]](#endnote-6)^ The eventual outlawing of dynamite fishing prior to 1925 speaks to a general recognition among the public of the destructive nature and pervasive use of these practices, though apparently the laws were not well heeded until the 1930s (Anonymous 1901c, 1905; Thrum 1925).^[[7]](#endnote-7)^ In the early 1900s, increased coastal development and harbor construction altered the land-sea interface, resulting in increased land-based pollution and mechanical habitat destruction of reef environments. Many coastal estuaries were dredged and filled and riparian systems were channelized, further increasing sedimentation and nutrient loading in nearshore reef areas. The impacts of the harbors and filling of wetland, estuarine, and coral habitats on coastal fisheries resources and coral habitat in the early 1900s has been documented in extensive interviews with indigenous fishers and community elders (Maly & Maly 2003c, d)^[[8]](#endnote-8)^. Impacts to coastal marine areas accelerated in the late 1930s and onward due to the increased development of coastal zones, which resulted in increased storm water run-off and other land-based pollution, and the continued impacts from major dredge and fill operations in select areas targeted for development (Banner 1974; Hunter & Evans 1995).

Impacts to corals from coastal development continue today, originating from a variety of sources, including cesspool and septic systems, of which Hawai‘i has the highest number in the US (Friedlander et al. 2008b), channelization of streams, and the increase in impervious surfaces in coastal zones. Some large-scale impacts, however, have been alleviated. In the 1970s, spurred on by the Clean Water Act, significant improvements began to be made to public sewage systems that reduced nutrient and sediment inputs to nearshore reef zones. In the late 1970s, sewage treatment plants were required to raise their treatment standards and outfalls were extended past nearshore environments to reduce the impact and which enabled some recovery in coastal lagoons (e.g. Kane‘ohe Bay) (Grigg 1994; Hunter & Evans 1995). Despite these improvements, there is additional evidence that corals are being impacted by new threats including an increased prevalence of disease, bleaching events and ocean acidification, which are related to human influence (Aeby et al. 2008; Friedlander et al. 2008b; Friedlander et al. 2004; Jokiel et al. 2009; Jokiel et al. 2004). Synthesis reports of the current condition of reef corals in the Hawaiian archipelago point to human impacts from overfishing, land-based pollution, disease, bleaching, recreational over-use, and alien species (Friedlander et al. 2008b; Friedlander et al. 2005; Friedlander et al. 2008c). Over the past 1-3 decades, monitoring programs reveal an average decline in coral cover of 8-12%, with some areas exceeding 30% (Friedlander et al. 2008c). Some areas, however, show increasing coral cover, which has been attributed to amelioration of human impacts as well as natural variability from storm and surf events (Dollar & Grigg 2004).

A.2 Papahānaumokuākea (Northwestern Hawaiian Islands)

Table A.2: Timeline of major events and proximate stressors (1-6) impacting the EcoState for corals in the Northwestern Hawaiian Islands. Proximate stressors are indicated with regard to their intensity: x = low, X = medium, X [emboldened] = high.

| **Date (AD)** | **EcoState** | **Description** | **1. Overexploitation** | **2. Invasive Species** | **3. Land-Based Pollution** | **4. Disease** | **5. Climate Change** |
| --- | --- | --- | --- | --- | --- | --- | --- |
| <1250 | 1 | Pristine |  |  |  |  |  |
| 1250-1870 | 1 | No use; semi-permanent indigenous habitation restricted to Nihoa & Mokumanamana (Necker) Islands after AD 1400 |  |  |  |  |  |
| 1870-1890 | 1.5 | Commercial dredging at Midway & FFS for harbors, runways |  |  | x |  |  |
| 1890-1915 | 2 | Guano mining operations at Laysan w/ modifications for harbor; Minor habitation elsewhere by feather poachers, fishermen |  |  | x |  |  |
| 1915-1937 | 2 | Ephemeral human habitation; some major military exercises |  |  | x |  |  |
| 1937-1941 | 2.5 | Major dredging & construction activities at Midway Atoll |  |  | x |  |  |
| 1941-1970 | 2.5 | Construction of facilities and habitat modification for human habitation for WWII |  |  | x |  |  |
| 1970-2009 | 2 | Ephemeral human habitation; restoration of terrestrial ecosystems |  | x | x |  | x |

The major impacts to reef-building corals in Papahānaumokuākea are the result of construction activities for harbors, anchorage areas, and for the building of runways and other infrastructure. These activities were primarily restricted to French Frigate Shoals and Midway Island. Construction on Midway was authorized by the US Congress in 1870, and the Saginaw was commissioned to blast a 600-foot wide ship channel through the reef rim into the lagoon (Bryan Jr. 1942). Beset by bad weather and other difficulties, the Saginaw only partially completed the project (Read 1912). Major modifications were also made to Midway Island in order to accommodate the personnel and facilities for a station operating the trans-Pacific communication cable, which was installed in the late 1890s. In 1937, after Japan renounced the Washington Treaty, which prohibited the build-up of Pacific naval bases, the Hawaiian Dredging Company was awarded a $50 million dollar contract to improve the harbor and build a naval air station on Midway. After World War II, other major construction activities continued (Apple 1973), but the primary impacts to reef-building corals were probably restricted to the dredging activities that took place in 1870 and in 1937 for the construction of the harbor and airplane runway, respectively. The impact of these dredging activities, however, was not minor. A significant amount of coral was removed to build a deep-water harbor and entryway into the lagoon, and the result of removal impacted the coral community directly, and indirectly through the alteration of biophysical processes that structure these atoll lagoon ecosystems. A similar project was undertaken in 1890 on French Frigate Shoals, and descriptions indicate the severity of dredging impacts on corals.^[[9]](#endnote-9)^ Human population in the NWHI decreased after WWII, and significant efforts were made starting in the 1960s and 1970s to restore the terrestrial habitats of emergent lands, which were highly degraded due to human activities, including introduction of invasive species. Construction of facilities on Midway Island and other areas continued, but other islands began to become uninhabited due to emergence of new communications technologies that did not require manned stations in the NWHI. Short-term monitoring of coral cover in the NWHI (2000-2002; 2006) showed no significant differences in coral cover (Friedlander et al. 2008c), and coral communities in the NWHI currently appear to be in relatively good condition (Kenyon et al. 2008a; Kenyon et al. 2007a; Kenyon et al. 2008b; Kenyon et al. 2007b; Maragos et al. 2004). Disease rates are considerably lower in the NWHI versus the MHI, but bleaching and acidification continue to be of concern (Aeby 2005; Aeby 2006; Aeby et al. 2003; Aeby et al. 2008; Jokiel et al. 2009; Kenyon & Brainard 2006).

B. Seagrasses/Algae

B.1 Main Hawaiian Islands

**Table B.1**: Timeline of major events and proximate stressors (1-6) impacting the EcoState for seagrasses/algae in the Main Hawaiian Islands. Proximate stressors are indicated with regard to their intensity: x = low, X = medium, **X** [emboldened] = high.

| **Date (AD)** | **EcoState** | **Description** | **1. Overexploitation** | **2. Invasive Species** | **3. Land-Based Pollution** | **4. Disease** | **5. Climate Change** |
| --- | --- | --- | --- | --- | --- | --- | --- |
| <1250 | 1 | Pristine |  |  |  |  |  |
| 1250-1825 | 1.25 | Subsistence use with non-lethal harvesting practices; cultivation of edible species in fishponds and reef environments | x |  |  |  |  |
| 1825-1920 | 1.5 | Subsistence use and sustainable commercial harvesting; sedimentation | x |  | x |  |  |
| 1920-1950 | 2.5 | Increased development of uplands, nutrient addition and sedimentation impacts | x |  | x |  |  |
| 1950-1977 | 3 | Introduction of invasive species, rapid spread throughout HI; coastal development increases nutrients to reef zones | x | X | x |  |  |
| 1977-2009 | 3.25 | Increased spread of invasive species; overharvest of herbivores; sewage system upgrades with reduction of nutrient inputs; injection wells, cesspools leach nutrients onto reefs | x | **X** | X |  |  |

The marine flora of the Hawaiian Islands consists of over 300 varieties of native algal species and a few seagrass species (Huisman et al. 2008). In Hawaiian prehistory, Polynesians used limu, or algae, as a food item and preservative, which is described in various ethnographic and scholarly works (Abbott 1984; Huisman et al. 2008; Titcomb 1972). The extent to which limu were harvested in prehistoric times is unknown, but some inferences about the level of impact can be made from Native Hawaiian practices observed in the historic period. The primary harvesting method was non-lethal picking of the top branches of the plant, leaving the base stems to regenerate so the plant could be continually re-harvested. Reef gleaning for limu harvesting was typically undertaken by women and children in nearshore shallow reef flats, and these practices were witnessed by early Westerners in the Hawaiian Archipelago (Bates 1854; Nordhoff 1874). Early surveyors of the marine algae community that arrived in the Hawaiian Archipelago made detailed observations of the methods by which Native Hawaiians harvested, prepared, and consumed limu, and recorded the Linnean style system developed by Hawaiians for algal species names (MacCaughey 1916, 1918a, b; Tilden 1902). Fishponds were also used as an aquaculture mechanism for different species of food algae, and favored species were transplanted in order to cultivate their growth for harvest (MacCaughey 1916, 1918a, 1918b). It is unlikely that Native Hawaiian subsistence harvesting resulted in any significant direct impact on algal communities in prehistoric time periods or in the early historic period. In contrast, substantial anecdotal evidence suggests that the purposeful cultivation of algal species for consumption likely resulted in a higher level of attention to their conservation and maintenance through active ecosystem engineering.

One of the most important issues with respect to seagrasses and algal communities is the historical abundances of large herbivores (green sea turtles) and the role of herbivory in seagrass/algal communities. Studies from Caribbean coral reef systems have suggested that herbivory has played a major role in seagrass systems and historical reductions in green sea turtle populations altered herbivore-seagrass dynamics (Bjorndal & Jackson 2003; Jackson et al. 2001). The relationships between turtle herbivory and algal communities has been altered in Hawai‘i due to the gradual inclusion of invasive algal species observed in turtle diets over the past three decades (Russell & Balazs 2009). It is unknown, however, how the reduction in grazing associated with decreases in turtle populations has impacted seagrass and algal beds in prehistoric time periods and thus the ecological impact is considered to be light.

Starting in the early 1800s, increased sedimentation from large-scale agriculture, overharvesting of herbivores (small & large), and wide-spread invasion of exotic species (e.g. Gracilaria salicornia; Kappaphycus alvarezii; Eucheuma striatum), began to affect reef ecosystem generally and reef flora. Commercial harvesting of marine algae started sometime in the mid- to late-1800s, and a review of the commercial fisheries in Hawai‘i revealed that 42-45,000 lbs of algae were sold per year in the early 1900s in the commercial markets (Cobb 1902; Cobb 1905a). The commercial harvest is unrecorded from 1903-1940, but based on anecdotal reports it is known that edible algae were harvested for markets during this period (Bryan 1915; MacCaughey 1916, 1918b). The commercial harvest was extremely low in the late 1940s, when the State of Hawai‘i began detailed accounting of commercial fisheries, but increased in the 1960s and 1970s to levels higher than observed by Cobb; harvest in the 1970s is estimated at 80,000 lbs per year (Abbott 1978). Commercial harvesting continues to present, and there is no evidence regarding the sustainable limits of harvesting. However, the continued practice of non-lethal harvesting, and some allowances for algae harvesting even in conservation areas points to a low level of impact from both commercial and subsistence harvesting (MacCaughey 1918b).

Currently, major threats to seagrass/algal communities include introduced invasive algae, land-based nutrient pollution, and overexploitation of herbivores (Smith et al. 2002; Stimson et al. 2001). Many of these impacts have been synergistic, and have common origins with the land-based activities impacting corals described above. Of these, the addition of high levels of nutrients and sediments from land-based sources is tied closely to terrestrial activities in the coastal zone, which started in the late 1700s with the large-scale harvesting of sandalwood and through the early to mid 1800s as commercial agriculture and ranching operations expanded. Plantation agricultures was introduced into the archipelago in the 1830s and became a major commercial activity by the mid-1800s (Cooper & Daws 1990; Daws 1968; Kaneshiro et al. 2005). Large-scale impacts from the commercial sugar plantations are known to have rapidly increased sediment to the nearshore environment around the mid-1800s, resulting in degradation of some reef sites and abandonment of fishponds that were filled in by sedimentation (Clifford 1991). The impacts on marine flora, however, are unknown and based on the reports of early observers, no major changes on subsistence harvesting strategies seem to have occurred.

In the past few decades, areas like Kane‘ohe Bay, O‘ahu and portions of the Maui coast have been intensively studied, and high levels of nutrient additions have been identified as a major factor in algal proliferation (Banner 1974; Huisman et al. 2008; Hunter & Evans 1995; Smith et al. 2002; Stimson et al. 2001). Some local impacts have been alleviated through the reduction in nutrient additions associated with the relocation of sewage outfalls in the late 1970s, but these impacts have also been offset by regional increased urbanization, channelization of streams, and proliferation of impervious surfaces, all of which drive nutrients in the coastal zone. In addition, the practice of wastewater injection has resulted in intrusion of nutrients into the nearshore marine environment, stimulating algal blooms (Smith 2003). As a result, the overgrowth of coral reefs by algae has been observed for many sites in Hawai‘i, and major actions have been promulgated to try to alleviate the problem of algal overgrowth, including federal grants for invasive algae removal efforts.

Invasive algal species have become a major problem for Hawaiian seagrass/algal communities in reef ecosystems. Many of the invasive species observed in the MHI were introduced from ship ballast water in the 1950s and 1960s, and these species have dispersed widely (e.g. Gracilaria salicornia; Kappaphycus alvarezii; Eucheuma striatum), fueling algal blooms and overgrowth that started in the 1950s and continue to present (Smith 2003; Smith et al. 2002). The reproductive capacity of many invasive algal species appears to be high, resulting in a rapid spread throughout the archipelago (Smith et al. 2002). Additionally, human harvesting of preferred herbivorous species such as the Yellow Tang (Zebrasoma flavescens) for the aquarium trade, and other finfish species targeted for consumption such as parrotfishes and Acanthurids have undoubtedly had an impact on the dynamics of reef algal communities (Huisman et al. 2008). Grazing has been identified as a critical factor in maintaining the ecological balance between algae and coral species, and the enormous harvesting pressure from subsistence, recreational and commercial fishing in recent decades has resulted in significantly lower levels of herbivores in unprotected areas versus fully protected no-take reserves (Friedlander et al. 2007a; Friedlander et al. 2007b; Friedlander et al. 2006; Friedlander & DeMartini 2002; Williams et al. 2009; Williams et al. 2006; Williams et al. 2008). In addition, herbivores prefer endemic algal species thus favoring the proliferation of invasive algae (Huisman et al. 2008). Herbivore harvesting from commercial, subsistence and recreational fishing sectors is difficult to ascertain, and the lack of a recreational fishing license in Hawai‘i makes estimating the effort and catch of non-commercial fishers difficult (Zeller et al. 2005). The impact of herbivore reduction on coral-algae dynamics is, however, estimated to be significant (Stimson et al. 2001).

B.2 Papahānaumokuākea (Northwestern Hawaiian Islands)

**Table B.2**: Timeline of major events and proximate stressors (1-6) impacting the EcoState for seagrasses/algae in the Northwestern Hawaiian Islands. Proximate stressors are indicated with regard to their intensity: x = low, X = medium, **X** [emboldened] = high.

| **Date (AD)** | **EcoState** | **Description** | **1. Overexploitation** | **2. Invasive Species** | **3. Land-Based Pollution** | **4. Disease** | **5. Climate Change** |
| --- | --- | --- | --- | --- | --- | --- | --- |
| <1250 | 1 | Pristine |  |  |  |  |  |
| 1250-1850 | 1.25 | Subsistence use only by visiting Native Hawaiians |  |  |  |  |  |
| 1850-1950 | 1.5 | Commercial and subsistence harvest of herbivores, impacting grazer-algal community dynamics | x |  |  |  |  |
| 1950-2009 | 1.25 | No commercial harvest, invasive species are limited; intact herbivore community |  | x |  |  |  |

Very little is known of the historic condition of seagrass and algal communities in the NWHI, but it is likely that very little harvesting of algae took place, except for subsistence by the inhabitants of Nihoa and Mokumanamana (Necker) Islands at the SE end of the NWHI chain (AD ~1400-1800). There is no evidence of commercial-scale harvesting of algae in the NWHI. Impacts are restricted, therefore, to introductions of invasive algae, harvesting of herbivores and nutrient additions. Due to the small land area of the emergent lands in the NWHI, very few terrestrial sources of nutrients are available for human modification and introduction into nearshore marine systems, as occurred in the MHI. There was significant terrestrial modification, e.g. due to the introduction of rabbits, goats and other animals, but it is unknown how these dynamics have affected the algal communities. Impacts from nutrient additions, however, are not likely to be major. Intensive harvesting of herbivores occurred in the NWHI from the mid 1800s to the mid-1900s, and it is unknown whether this exploitation released algal communities from grazing. Recent surveys show that in contrast to the MHI, the herbivore community is currently intact in the NWHI (Friedlander & DeMartini 2002). Invasive algae species do not appear to have become established in the NWHI as they have in the MHI, but a high degree of attention is being paid to the issue of invasive species in this area (Friedlander et al. 2008a; Godwin et al. 2006). Comprehensive substrate surveys indicate that corals are prevalent, and no invasive algal overgrowth has been observed (Kenyon et al. 2008a; Kenyon et al. 2007a; Kenyon et al. 2008b; Kenyon et al. 2007b). Natural meadows of endemic algal species occur in the NWHI, and the down-regulation of herbivores by high levels of apex predators appear to maintain these algal beds in this condition (Huisman et al. 2008:28). Additionally, deep reef (<50 m) algal beds appear to provide important nursery habitat for reef fish in the NWHI (Anonymous 2009a).

C. Suspension Feeders & Detritivores

C.1 Main Hawaiian Islands

**Table C.1**: Timeline of major events and proximate stressors (1-6) impacting the EcoState for suspension feeders and detritivores in the Main Hawaiian Islands. Proximate stressors are indicated with regard to their intensity: x = low, X = medium, **X** [emboldened] = high.

| **Date (AD)** | **EcoState** | **Description** | **1. Overexploitation** | **2. Invasive Species** | **3. Land-Based Pollution** | **4. Disease** | **5. Climate Change** |
| --- | --- | --- | --- | --- | --- | --- | --- |
| <1250 | 1 | Pristine |  |  |  |  |  |
| 1250-1800 | 1.5 | Subsistence use with only a few species targeted; growing human population | x |  |  |  |  |
| 1800-1860 | 2 | Commercial harvests of pearl oyster, spatially restricted | x |  |  |  |  |
| 1860-1900 | 2.5 | Commercial exploitation of beche-de-mer; sedimentation in coastal lagoons impact select species, including the pearl oyster | x |  | x |  |  |
| 1900-1950 | 3 | Purposeful introductions of exotic bivalves, crabs; overharvesting of some species; increased sedimentation | x | x | x |  |  |
| 1950-1975 | 3.25 | Ballast water introductions of invasive filter feeders, endemic species out-competed; | x | **X** | x |  |  |
| 1975-2009 | 3.5 | Some improvements in water quality, invasives continue to proliferate; some endemics rare | x | **X** | x |  |  |

In the prehistoric period, Native Hawaiians used various suspension feeders and detritivores for subsistence and for the manufacture of tools and ceremonial items such as religious idols and ceremonial staffs (kahili). Pearl oysters (Pinctada margaritifera), in particular, were highly favored as a food product and for the manufacture of fishing lures and religious items. The Hawaiian name for the pearl oyster, pa, is the same name given to trolling lures, the hooks for which were manufactured from pearl oysters. Pearl oyster lures were used unbaited, and the observers of Native Hawaiian fishing commented that the shimmer and glisten of the shell’s nacreous layer was enough to invite a fish to strike (Beckley 1883). Fishermen were known to treasure fish lures made from pearl oysters that were particularly effective (Coulter 1931). Pearl oyster habitat in Hawai‘i includes most shallow lagoons (Sims 1993). Historically, they were found in abundance at Pearl Harbor, also known as Pu‘uloa (mound of pearl), as well as in a few other areas including the Puna district on Hawai‘i Island and Kane‘ohe Bay, O‘ahu.^[[10]](#endnote-10)^ Pearl oysters must have been either widely distributed or traded in prehistory, however, because pearl shell fishhooks are present in archaeological assemblages from coastal areas throughout the MHI; however, the majority of pearl shell fishhooks from archaeological middens are found on the older islands of O‘ahu and Kaua‘i (Emory et al. 1959:32, Fig 14). Pearl oysters were rare in the MHI by 1901 (Cobb 1902:415). Hooks derived from shell were preferred over those derived from bone which were also common. The use of inferior bone hooks evidences some potential for shell scarcity in pre-contact times, but it is unknown whether scarcity is attributed to natural distribution of pearl shells or overharvest during this period, as has occurred in other Pacific Islands (Allen 2002).

Native Hawaiians also harvested other bivalves from estuarine and coral reef lagoon environments (Deering 1899; Wilkes 1845). Native Hawaiians consumed some species of bêche-de-mer, boiling the large ones, and consuming the small ones raw (Bryan 1915), and shrimp species were harvested from freshwater streams and in estuarine environments for food and as bait for marine fishing (Brassey 1881). It is unknown what other suspension feeding and detritivore species were consumed in a subsistence fashion by pre-contact Hawaiians, but it is likely, given their affinity for seafood and detailed ecological knowledge of marine species, that other species were harvested for subsistence (MacCaughey 1916, Titcomb 1972). Aside from the pearl oyster shell middens observed in focal areas like Pearl Harbor, little evidence exists for impacts to this guild during the prehistoric period.

During the first decade in the 1800s a commercial pearl oyster fishery was developed in Pearl Harbor, near Honolulu, where pearl oysters were still found in dense quantities at the head of the harbor. The fishery was declared a royal monopoly by reigning King Kamehameha I (Campbell 1825; Jarves 1843), who employed divers for harvesting the oysters and was gifted an oyster dredge in 1815 by western visitors, which likely aided in expanding harvesting operations (Corney & Alexander 1896). Pearl oyster exports in 1835 included $6,000 in shells, and $15,000 worth of imported shells from the Society, Friendly, and Caroline islands for export to foreign markets (Ruschenberger 1838) and these exports continued in subsequent years (Niles 1837). The shells were ultimately shipped to China for a few decades until the beds were exhausted, sometime before 1850 (Cobb 1902). Pearl oysters do not tolerate high sediment loads (Yukihira et al. 1999), so it is likely that increased sedimentation after 1860 due to alteration of the uplands from ranching and agriculture activities also impacted this species.

Shortly after the pearl fishery was exhausted, a bêche-de-mer fishery was developed in the Hawaiian Islands. An export market had already developed in other Pacific Islands, with China and Manila being the principal markets, which was fueling Chinese demand for these animals’ supposed medicinal qualities. In 1861 a private firm advertised in the local newspapers that it would purchase bêche-de-mer and Native Hawaiian fishermen began harvesting them. Exports reached 7,135 lbs in 1864, but the commercial fishery was short lived. When Cobb arrived in 1900, he observed that bêche-de-mer were still quite abundant, but it is likely that favored species were preferentially harvested and may have been locally extirpated. This is also supported by current evidence that the species of bêche-de-mer now common on Hawaiian reefs are not preferred species, and some favored species for food and medicinal qualities are now extremely rare (Skillings 2009).

Government agencies explored introductions of exotic clam and oyster species for commercial exploitation in the late 1800s, and exotics were introduced on larger scales in the early 1900s by the territorial government. Introductions included Chesapeake, Australian and Japanese oyster species, which were introduced into a number of shallow lagoon areas, particularly Kane‘ohe Bay, as well as various crab and clam species. Many of these species failed to establish themselves fully, owing largely to the fact that many introduced were temperate rather than tropical species, but remnant populations from these introductions still remain in some areas. Some tropical species, however, thrived and have become commonly sought species by commercial and recreational fishermen (e.g. the Samoan crab, Scylla serrata) (Brock 1960; Edmondson & Wilson 1940). More recently, ballast water and other mechanisms have resulted in the introduction of invasive sponges, including introduced or cryptogenic sponges, polychaetes, bryozoans, tunicates and other suspension feeding invertebrates. In some areas, invasive sponges and other invertebrates have recently been observed to cover almost 100% of surfaces in highly invaded harbor areas (Coles et al. 1999). As in the algal community, introductions have largely occurred from the early 1900s through to the present, with the distributions and likely origins of newly introduced species continually being described in sequential surveys (e.g., for sponges, Bergquist 1967; Coles et al. 1999; De Laubenfels 1950, 1951, 1957).

In summary, there is no evidence of prehistoric over-harvesting of suspension feeders or detritivores. Even the shell middens do not suggest overharvest in Pearl Harbor, as a sufficient stock existed in 1800 to allow for commercial exploitation. The historical evidence suggests that the commercial exploitation was limited to a few select species of bêche-de-mer, a concentrated targeting of pearl oysters for a lucrative export market, and light commercial harvesting of estuarine shrimp, other bivalves and perhaps a few other species in a minor capacity. Unlike in other reef areas, a commercial sponge fishery never developed in Hawai‘i (J. N. Cobb [1902], for example, commented that the sponges he observed in his 1900 survey were of “an inferior quality”). MacCaughey (1918c) commented on the diversity and types of sponges he observed, and also commented that none of the Hawaiian species had been harvested commercially, nor had foreign species been introduced for commercial purposes.

It is doubtful that the large-scale harvesting even of pearl oysters and bêche-de-mer, was sufficient to impact, at the guild level, the diverse populations of suspension feeders and detritivores in the Hawaiian archipelago. Even after these commercial markets had expired, early coral reef surveyors remarked on the abundance of bêche-de-mer, hydroids, and jellyfish species (MacCaughey 1918c). If commercial overharvest had an impact at all, it was likely to be spatially restricted (e.g. pearl oysters at Pearl Harbor), or it was likely rapidly filled by related species (e.g. by congeners among the Holothurians). The introduction of invasive species, however, constitutes a significant impact to the endemic Hawaiian species that comprise this guild, and are judged to be considerable in the post-WWII period. Another major impact to suspension feeders and detritivores is the large-scale alteration of estuarine, riparian and nearshore marine systems from land-based activities, which has degraded reef habitat. For example, oral history interviews with Native Hawaiian elders consistently point out that freshwater shrimp species used traditionally as bait in marine fishing by Hawaiians are not commonly found in streams due to the urbanization of watersheds and riparian systems (Maly & Maly 2003c, d). In the case of this guild, the purposeful and non-purposeful introduction of invasive species and the degradation of habitat due to land-based activities have constituted the major historical impacts.

C.2 Papahānaumokuākea (Northwestern Hawaiian Islands)

**Table C.2**: Timeline of major events and proximate stressors (1-6) impacting the EcoState for suspension feeders and detritivores in the Northwestern Hawaiian Islands. Proximate stressors are indicated with regard to their intensity: x = low, X = medium, **X** [emboldened] = high.

| **Date (AD)** | **EcoState** | **Description** | **1. Overexploitation** | **2. Invasive Species** | **3. Land-Based Pollution** | **4. Disease** | **5. Climate Change** |
| --- | --- | --- | --- | --- | --- | --- | --- |
| <1250 | 1 | Pristine |  |  |  |  |  |
| 1250-1925 | 1 | No impacts |  |  |  |  |  |
| 1925-1930 | 2 | Commercial overexploitation of pearl oysters, spatially restricted | x |  |  |  |  |
| 1930-2009 | 2 | No known harvest; pearl oysters never recover from exploitation | x |  |  |  |  |

Impacts in the NWHI are restricted to the short-lived commercial extraction of pearl oysters at Pearl & Hermes Atoll. Pearl oysters were discovered on July 8, 1825, by Capt. Benjamin Morell Jr. of the ship Tartar (Casserley 1998). Oysters were harvested intensively from 1928 to 1930 by the Lanikai Fishing Company, who employed Filipino divers to harvest oysters from the lagoon. According to Casserley (1998), Capt. William Anderson with the Lanikai Fishing Company rediscovered oysters in the lagoon in 1925 while on a fishing expedition. Anderson and his team removed over 150,000 pearl oysters, constituting some 150 tons, from the lagoon (Galtsoff 1933).^[[11]](#endnote-11)^ Surveys by a government-funded surveying team led by Paul Galtsoff, and employing the same Filipino divers employed by the Hawaiian Sea Products Company, found less than 500 oysters in 1930. Subsequent surveys in 1950, 1969 and 2003 indicate similar abundances, indicating this population has never recovered from exploitation (Apple 1973; Keenan et al. 2006). The only remaining evidence of exploitation (commercial or otherwise) is associated with fishing activities operating in the NWHI in the latter part of the 19^th^ century. In 1882, the fishing schooner Ada visited the NWHI and harvested bêche-de-mer at Pearl & Hermes, Laysan and Lisianski (Casserley 1998); the amount taken is unknown. The ecological impact in the modern period is judged to be light, because only two fisheries focused on suspension feeder/detritivores (pearl oyster & bêche-de-mer). Though the pearl oyster population at Pearl and Hermes Atoll has not recovered (Keenan et al. 2006), the overall impact to the ecological function of this guild is estimated to be light because many other species comprising this guild were not targeted for commercial extraction and there is no evidence supporting loss of ecological function of suspension feeding and detritivory as a result of these human impacts.

D. Large Herbivores

D.1 Main Hawaiian Islands

**Table D.1**: Timeline of major events and proximate stressors (1-6) impacting the EcoState for large herbivores in the Main Hawaiian Islands. Proximate stressors are indicated with regard to their intensity: x = low, X = medium, **X** [emboldened] = high.

| **Date (AD)** | **EcoState** | **Description** | **1. Overexploitation** | **2. Invasive Species** | **3. Land-Based Pollution** | **4. Disease** | **5. Climate Change** |
| --- | --- | --- | --- | --- | --- | --- | --- |
| <1250 | 1 | Pristine |  |  |  |  |  |
| 1250-1450 | 3 | Human predation on adults and eggs; Introduction of species that prey on hatchlings and eggs | X | x |  |  |  |
| 1450-1778 | 2.5 | Turtles reserved for consumption only by males of high social status | x | x |  |  |  |
| 1778-1820 | 2 | Native Hawaiian populations reduced by disease; chiefly re-direction of labor away from traditional fishing | x | x |  |  |  |
| 1820-1850 | 3 | Lifting of indigenous restrictions on consumption; commercial harvesting initiates; attrition of young, able-bodied men to foreign industries | X | x |  |  |  |
| 1850-1975 | 4 | Increased commercial harvest with depletion of adults & juveniles; subsistence use | **X** | x |  |  |  |
| 1975-2009 | 3 | Protected by statute; exploitation ceases; diseases documented; dietary shift to invasive algae species |  | x |  | X |  |

Green sea turtles (Chelonia mydas) are the only large-bodied (> 1 m) herbivore in Hawaiian coral reef ecosystems. In Hawaiian prehistory, two major threats to green sea turtles existed: human predation on adults, juveniles and eggs and predation on hatchlings and eggs by introduced species, including the Polynesian rat, dogs, and ungulates (pigs). Though evidence is equivocal, the combined effects of these pressures are likely to have reduced populations and contributed to large and rapid decreases in green sea turtles during the early prehistoric period (i.e. primarily during the first century of Polynesian habitation of the Hawaiian archipelago). Our estimation of ecostates during the prehistoric period is derived from:

1. Life history traits and high vulnerability of sea turtles to human stressors;^[[12]](#endnote-12)^
2. Archaeological midden data, which shows that turtles comprised a low percentage of midden weight in excavated sites that post-date (>1300-1400 AD) early settlement of the archipelago;^[[13]](#endnote-13)^
3. Ethnographic evidence of cultural restrictions on turtles for consumption only by males in ruling classes, which were likely implemented during a period that witnessed the rise of major polities and the development of socio-political complexity, from 1400-1600 AD;^[[14]](#endnote-14)^
4. Early historic accounts of Native Hawaiians making high-cost trips to peripheral environments in the MHI to harvest turtle;^[[15]](#endnote-15)^
5. Evidence from the late historic period of turtles nesting in the MHI only in sparsely populated areas;^[[16]](#endnote-16)^
6. Contemporary biogeographical distributions of turtle nesting sites in the archipelago showing that over 90% of green sea turtles currently nest in the NWHI, which has functioned as a refuge since prehistoric times;^[[17]](#endnote-17)^
7. The availability of prime turtle habitat in the MHI at the time of Polynesian discovery;^[[18]](#endnote-18)^
8. Differences in observations of first explorers to the NWHI, who commented almost without exception about dense turtle populations in the region, versus first explorers and traders in the MHI, who recorded no observations on wild turtle populations and did not receive them in trade, and;
9. Evidence from other island environments of the impacts of introduced species on turtle populations, including rat predation on turtle eggs and hatchlings,^[[19]](#endnote-19)^ which may have impacted turtle nesting populations in prehistoric and historic times.

Sea turtles represent high-value prey due to their high meat weight and ease of capture, and thus are likely to have been easy targets for the first humans arriving in the archipelago. In Polynesia, a general pattern of early reduction or rapid elimination of turtles on nesting coral sand beaches (a favored habitat for both nesting turtles and first landing sites by voyaging Polynesians) is so often observed that turtle bones in archaeological strata are now considered a proxy for first human habitation of a site (Allen 2007; Anderson 1995; Kirch & Yen 1982; Steadman 1995).^[[20]](#endnote-20)^ The archaeological pattern in Hawai‘i is consistent with ethnographic evidence that turtle in Hawai‘i was reserved for consumption only by males of superior rank (e.g. chiefs, high priests) (Bryan Jr. 1938:67; Kalakaua 1888:32, 303; Malo 1951:29). It is unlikely, however, that sanctions on turtles existed at the time of discovery by Polynesians because resource protections typically develop as a human adaptive response to rarity and require robust human institutions for successful implementation (Allen 2007; Cinner & Aswani 2007; Johannes 2002).

The ethnographic pattern observed in Hawai‘i whereby turtles were reserved for consumption by chiefly classes is thus consistent with other high islands in Polynesia where early reductions made turtles rare and as a consequence, social restrictions on consumption developed (Allen 2007). Allen (2007) has attributed the pattern of cultural protections and population reductions in Pacific Islands to the ratio of turtle to human habitat: in high islands the habitat ratio is skewed toward human hunters and their introduced fauna and turtles become rare and protected; alternatively, in atoll environments turtles have persisted due to the low carrying capacity of terrestrial environments for human habitation versus turtles and cultural protections are typically not imposed. This pattern fits well with Hawai‘i, where nesting beaches in the high islands of the MHI have been serially extirpated (Balazs 1973), but have persisted in the atolls of the NWHI. In summary, the available evidence suggests that combined pressures of human harvesting and invasive species in the early prehistoric period were likely sufficient to significantly reduce turtle populations and nesting beaches in the MHI, and sequester the majority of the nesting population in the NWHI. In the late prehistoric period, subsistence harvest occurred in the MHI but consumption was restricted by cultural sanctions that developed as a response to rarity, and we have conservatively estimated that these sanctions resulted in a 10% population recovery from AD 1450-1778. We believe our 10% recovery estimate is conservative given the small proportion of people that were apparently allowed to consume turtle under these sanctions, and ecological evidence that green sea turtles nesting populations are capable of significant recovery if released from harvesting pressure (e.g. green sea turtle nesting increased from ~100 to ~500 individuals over a 30-year period in the NWHI, Balazs & Chaloupka 2004).

In the post-contact period, anecdotal evidence and modern records of commercial harvesting reveal evidence that green sea turtle has always been a preferred food, among both Hawaiians and foreigners. Turtles were probably released from human harvesting pressures from 1778-1820 due to the combined effects of indigenous depopulation due to disease, chiefly re-direction of labor by chiefs away from traditional fishing to other activities (e.g. sandalwood harvesting; construction of western-style vessels) (Kirch & Sahlins 1992; Mills 2003; Kuykendall 1938:88-90),^[[21]](#endnote-21),^^[[22]](#endnote-22)^ and attrition of able-bodied men to various maritime industries (principally the whaling trade, from the 1820s-1860s), which together removed a substantial percentage of the section of the population typically employed in the professional fishing classes and in subsistence fishing from the Hawaiian Islands^[[23]](#endnote-23)^ (Jones 1948; Kuykendall 1938).

Descriptions of turtles as commonly caught and abundant in the early post-contact period provide some evidence that turtles were abundant in the MHI at the time of western contact (Table S6). Vancouver observed green sea turtle served in ceremonial feasts as early as 1794 (Vancouver 1801), but typically early observers in the MHI did not commonly comment on turtles in reef environments. This stands in stark juxtaposition to comments from first western explorers that visited the NWHI, which are riddled with observations of dense turtle populations (see Section 3.4.2). Later observers remarked on how green sea turtle was a favored food among both royalty and commoners after the abolishment of the indigenous belief system (the kapu system) in 1819, which prescribed the prohibitions on food (including turtle) with respect to both hierarchy and gender (Bryan 1915; Kalakaua 1888; Stewart 1828). The abolishment of consumption sanctions in 1819 allowed turtle to become a commonly consumed food item among all social classes. We have conservatively estimated that these historical events resulted in a 10% recovery from 1778-1820, which is substantially less than the documented recovery of green sea turtles in the NWHI that has occurred from the late 1970s to current (Balazs & Chaloupka 2004).

Starting in the late 1790s, the number of foreign vessels visiting Hawaii began to increase. The first commercial fisheries and markets developed in the port cities, which became the focus for economic activities and trade with foreign vessels (Beechert 1991). Commercial harvesting of turtle to supply these inchoate markets was initiated around ~1830 and continued until 1978 when the species was listed under the Endangered Species Act (ESA). Ships began visiting the NWHI to harvest turtles in the mid-1800s, evidencing rarity or further depletion in MHI turtle populations by at least 1850 (Casserley 1998). In the early 1900s, commercial harvest consisted of just over a hundred individuals per year and records confirm consistent harvest until the 1970s (Cobb 1902; Cobb 1905a; DAR 2007; Witzell 1994). Observers in the early 1900s commented that juveniles were common in commercial markets (Bryan 1915).

After protection under the ESA, green sea turtle populations have recovered, however nesting continues to be concentrated in refugia sites, with the majority of nesting sites still occurring in the NWHI at French Frigate Shoals (Balazs & Chaloupka 2004). An assessment of green sea turtles has suggested that populations may be near 83% of pre-exploitation biomass (Chaloupka & Balazs 2007) but questions have arisen regarding these findings, including doubts about whether the green sea turtle is nearing its ecological carrying capacity (Snover 2008). In the past few decades, disease has become a major problem in the Hawaiian green sea turtle population. The tumor-causing disease fibropapillomatosis has been identified as an inhibiting factor for recovery of the green sea turtle population (Aguirre et al. 1994; Aguirre et al. 1998; Chaloupka et al. 2009), and prevalence rates from capture experiments conducted from 1985-1996 indicate a prevalence rate of ~46% (highest recorded rate: 61%, 1995) (Balazs et al. 1997). This disease is related to increased nitrogen input from developed coastal watersheds, which fuels invasive algae populations and the herpes virus implicated with fibropapillomatosis (Van Houtan et al. 2010). Climate change is also a major threat for the future of green sea turtles, particularly as low-lying nesting beaches in the NWHI are threatened with inundation from sea level rise (Baker et al. 2006).

D.2 Papahānaumokuākea (Northwestern Hawaiian Islands)

**Table D.2**: Timeline of major events and proximate stressors (1-6) impacting the EcoState for large herbivores in the Northwestern Hawaiian Islands. Proximate stressors are indicated with regard to their intensity: x = low, X = medium, **X** [emboldened] = high. FFS = French Frigate Shoals.

| **Date (AD)** | **EcoState** | **Description** | **1. Overexploitation** | **2. Invasive Species** | **3. Land-Based Pollution** | **4. Disease** | **5. Climate Change** |
| --- | --- | --- | --- | --- | --- | --- | --- |
| <1250 | 1 | Pristine |  |  |  |  |  |
| 1250-1790 | 1 | No evidence of impacts; human population restricted to Nihoa and Mokumanamana (Necker); cultural protections for NWHI (> AD 1450) |  |  |  |  |  |
| 1790-1820 | 1 | Reduced visits by Native Hawaiians to NWHI due to depopulation, erosion of cultural knowledge of NWHI; first Western exploration | x |  |  |  |  |
| 1820-1860 | 2 | Subsistence harvest by shipwrecked crews; exploitation of turtles on NWHI islands by Western explorers, traders, whalers, sealers | X |  |  |  |  |
| 1860-1915 | 4 | Depletion of nesting beaches and populations by commercial fishing operations, land-based crews & shipwrecked crews | **X** |  |  |  |  |
| 1915-1950 | 4 | Japanese fishermen excluded; limited Hawai‘i-based fleet continues to harvest | **X** |  |  |  |  |
| 1950-1974 | 3 | Spatially restricted commercial operations at FFS, with air transit to Honolulu | X |  |  |  |  |
| 1974-2009 | 2.5 | Protection under ESA, nesting recovering at FFS; disease |  |  |  | X |  |

The NWHI have always functioned as a geographic refuge for large errant megafauna, due to the isolation of the region, limited human habitability, and dispersed geography of reefs and atolls. Despite is isolation and long distance from the inhabited MHI, archaeological sites confirm Native Hawaiians were accessing the lower portion of the NWHI in prehistoric times (Emory 1928; Cleghorn 1988), probably after AD 1400-1500 (Kikiloi, pers. comm.) which corresponds with the zenith in prehistoric population (Dye and Komori 1992). Anecdotal information from early explorers (Rauzon 2001)^[[24]](#endnote-24)^ is equivocal on whether Native Hawaiians were accessing this region in early historic times, but residents of Ni‘ihau Island did visit Nihoa during summer months until the late 1800s (Iversen et al. 1990).^[[25]](#endnote-25)^ Despite this, Nihoa and Mokumanamana (Necker) contain no sandy beaches and thus did not support nesting turtle populations, but grazing turtles may have been targeted for subsistence, as seabirds were (Cleghorn 1988). Beyond Mokumanamana, French Frigate Shoals is the site of the major nesting beach for green sea turtles in the NWHI. This area is ~650 km (400 miles) from Kaua‘i, and it would have constituted a high-cost trip to visit these islands in voyaging canoes. Regardless, the islands in this zone (beyond Mokumanamana) were subject to cultural protections that arose sometime in prehistory (Kikiloi 2010), and these protections probably ensured that this area remained off-limits to extractive use (Kikiloi, pers. comm.).

Consistent voyaging to the NWHI probably stopped sometime shortly after contact, and analyses of 113 whalers logs visiting the NWHI from 1791-1878 contain no reference to Native Hawaiian fishermen (Iversen et al. 1990:22). The fact that Native Hawaiians were not known to access this area in the early historic period may be the result of disease epidemics, (which claimed Native Hawaiian knowledge holders and their cultural information passed down through oral traditions), may simply reflect the high-cost of trips to this zone, or may evidence the persistence of cultural protections that made the area off-limits. Hawaiians from the island of Kaua‘i, accompanying an exploring expedition in 1788, were themselves unaware of the existence of Nihoa Island in the NWHI, but upon returning back to Kaua‘i and consulting within their communities, discovered a traditional knowledge of the island.^[[26]](#endnote-26)^

Early European explorers of the NWHI commented almost without exception on the high abundances of green sea turtles, which often served as an easily captured and highly prized food item. Some observations from explorers reveal historical nesting locations that are no longer known as nesting habitat, including Laysan Island, Midway Atoll and Pearl and Hermes reef (Anonymous 1859a; Morell 1832). The NWHI also became known for shipwrecks, and many of the shipwrecked crews were forced to survive on locally available fauna, including terrestrial nesting sea birds, and sea turtles and monk seals hauled out on sandy beaches (Bailey 1952). The impact of these shipwrecked crews on sea turtle populations was not likely to be inconsequential. For example, the crew of the Saginaw, upon having been shipwrecked at Kure Atoll, began harvesting turtles immediately.^[[27]](#endnote-27)^ Captain F. D. Walker, himself a survivor of a shipwreck in the NWHI, described a large midden of turtle remains from a shipwreck survivor camp that had been established on Midway.^[[28]](#endnote-28)^ Later on, shipwrecked crews were deprived of abundant sea turtles, due to previous harvesting by other shipwrecked crews and removal by commercial fishing operations. Commercial harvesting of turtle in the NWHI began in earnest sometime in the early to mid 1800s, and consisted of both Hawai‘i- and Japan-based fishing crews. Casserley (1998) described two fishing expeditions to the NWHI in the 1800s.^[[29]](#endnote-29)^

Subsistence harvesting of turtles in the NWHI was also occurring during the historic period by commercial maritime fleets engaged in a variety of industries (e.g. whalers, fur-traders), government vessels and land-based crews of feather poachers and guano miners (Apple 1973; Unger 2003).^[[30]](#endnote-30)^ Observations by voyagers to the NWHI and arrests by US government agents point to a significant presence of Japanese fishing vessels and feather poachers until around the late 1910s. Sustained commercial and subsistence harvesting eliminated large nesting colonies on Laysan, Pearl and Hermes, and Midway Islands, and probably severely depleted colonies on French Frigate Shoals. Notably, there are no historical reports of nesting habitat on Kure Atoll, on which the Polynesian rat became established in prehistoric times, which further evidences the effects of rat predation on turtle nesting. Several Honolulu-based fishing crews worked the NWHI for fish and turtle in the early 1900s (Anonymous 1924; Hamamoto 1928; Iversen et al. 1990; Konishi 1930). After World War II, commercial fishing companies used the runway at French Frigate Shoals to bring green sea turtles to market in Honolulu (Anonymous 1946; Iversen et al. 1990). Apple (1973) described the scope of these operations, which ended in the late 1950s, ^[[31]](#endnote-31)^ and their sale in markets is confirmed in fishery data (DAR 2009). Since the late 1950s, turtle populations have been in a recovery mode in the NWHI. Nesting abundances of green sea turtles in the NWHI have increased from under a 100 individuals to over 500 since being listed under the Endangered Species Act, an increase of more than 500% in three decades (Balazs & Chaloupka 2004).E. Large Carnivores

E.1 Main Hawaiian Islands

**Table E.1**: Timeline of major events and proximate stressors (1-6) impacting the EcoState for large carnivores in the Main Hawaiian Islands. Proximate stressors are indicated with regard to their intensity: x = low, X = medium, **X** [emboldened] = high.

| **Date (AD)** | **EcoState** | **Description** | **1. Overexploitation** | **2. Invasive Species** | **3. Land-Based Pollution** | **4. Disease** | **5. Climate Change** |
| --- | --- | --- | --- | --- | --- | --- | --- |
| <1250 | 1 | Pristine |  |  |  |  |  |
| 1250-1450 | 4 | Human harvesting of monk seal and hawksbill sea turtles; biological invasions; monk seals and hawksbill sea turtles rapidly become rare; Subsistence harvest of carangids, sharks, and large piscivores | X | x |  |  |  |
| 1450-1778 | 3 | Implementation of social status rules for consumption of some large piscivorous fish; traditional fishery management methods are codified, specialization of fishing profession and gear | x | x |  |  |  |
| 1778-1820 | 2.5 | Recovery of piscivorous fish due to release in harvesting pressure due to disease epidemics among Native Hawaiian population, chiefly re-direction of labor away from traditional farming & fishing, and attrition of able-bodied men to foreign commercial trades; chiefly support for professional fishing classes wanes; famines and fish shortages due to lack of effort; monk seal and hawksbills do not recover | x | x |  |  |  |
| 1820-1875 | 4 | Commercial fishery markets initiate and develop; depletion of sharks for fin export market; nearshore fisheries primary zone of exploitation; piscivorous fish a principal target for fisheries | X | x | x |  |  |
| 1875-1939 | 5 | Declines in reef fisheries; investigations by US Bureau of Fisheries; over-harvesting of juveniles; destructive fishing practices | X | x | x |  |  |
| 1939-1945 | 4 | WWII; fishing effort restricted due to martial law; short-lived recovery |  | x | x |  |  |
| 1945-2009 | 5 | State-sponsored shark eradication and introduction of invasive species; overfishing; large predators very rare on reefs in MHI | **X** | x | x | x |  |

Early Polynesian settlers in the Hawaiian island chain must have been greeted with an astonishing array of predators in the coral reef environment. Contemporary studies in uninhabited atolls and reef environments, which have endured some stresses associated with anthropogenic activities, reveal a trophic structure dominated by apex predators (e.g. Sandin et al. 2008). Arriving Polynesians witnessed an environment dominated by apex predators including reef sharks, large carangids, snapper and grouper species, the Hawaiian monk seal, and the hawksbill turtle, all of which have been recoverd in faunal remains at archaeological sites. Of these predators the endemic Hawaiian monk seal is among the largest-bodied apex predators in Hawaiian coral reef ecosystems. With no land predators, monk seals would probably have exhibited little fear of humans, and would have thus been easy to capture. As such, monk seals would have been high value prey, as they provided a significant return on investment in terms of the meat gained per hunting effort.

The majority of the species currently resides in the NWHI, and this distribution has verifiably persisted since the early historic period. In prehistoric times, however, it is highly likely that the monk seal was distributed throughout the Hawaiian archipelago, including the MHI. Monk seal populations and rookery sites probably experienced early and rapid declines in the MHI early after Polynesian settlement, due to harvesting by human hunters and possibly due to the introduction of the Polynesian dog. Early and rapid monk seal population declines are supported by multiple lines of evidence, including:

1. Life history traits and extreme sensitivity and vulnerability of monk seals to human stressors;^[[32]](#endnote-32)^
2. Little to no archaeological evidence of monk seals in middens, which is more likely attributed to early and rapid overharvest and the deleterious effects of invasive species than depositional processes or pre-human rarity in the MHI;^[[33]](#endnote-33)^
3. No known records for a historically recorded Native Hawaiian name for the monk seal, which evidences that monk seals became rare before it developed a cultural identity;^[[34]](#endnote-34)^
4. Anecdotal evidence of Native Hawaiian harvesting of monk seals in the historic period.^[[35]](#endnote-35)^
5. Low estimates for pre-human monk seal populations, contributing to their extreme vulnerability;^[[36]](#endnote-36),^^[[37]](#endnote-37)^
6. Primary habitat requirements for the monk seal and existing MHI habitats at the time of Polynesian discovery; ^[[38]](#endnote-38)^ and,
7. Evidence of high abundances of Polynesian dogs in the Hawaiian archipelago at the time of contact^[[39]](#endnote-39),^^[[40]](#endnote-40)^, historical documentation of wild dog populations in Hawai‘i,^[[41]](#endnote-41)^ and evidence from other coastal settings confirming the deleterious effects of domestic and wild dogs on marine mammal populations in prehistory;^[[42]](#endnote-42)^

In summary, the human colonization of Hawai‘i likely resulted in early and rapid prehistoric reductions in monk seal populations in the MHI. The most likely explanation for scant evidence of monk seals in the archaeological record is best explained by extreme rarity in monk seals in the MHI after the few decades of Polynesian discovery of the archipelago. Competing, but less likely, hypotheses include: 1) prehistoric inhabitants did not harvest monk seal in the MHI; 2) monk seals were never abundant in the MHI, or 3) depositional or taphonomic processes have resulted in monk seal remains not reaching midden deposits. Regardless, at the time of Western discovery, monk seals were either extremely rare or absent in the MHI and the population in the NWHI was subsequently discovered in the early 1800s.^[[43]](#endnote-43)^

Early Hawaiians also harvested hawksbill turtles, or Honu Ea, and used the shell for ceremonial and religious items, and for tools for the manufacture of implements. Even today, the density of hawksbill turtles in Hawai‘i is unknown, but ample anecdotal evidence of hawksbill shell (“tortoise”) used in Hawaiian cultural artifacts points to a widespread availability of these turtles in the archipelago at some point in the past, or at least significant inter-island trade for their shells taken in areas where they were common (Bingham 1849; Ellis 1826; Stewart 1828). Early explorers did, however, note the presence of hawksbills in the NWHI in the early 1800s (Morell 1832), evidencing some constriction in the biogeographic distribution of hawksbills as they are not currently observed in this region.^[[44]](#endnote-44)^ In the historic period, some commercial harvesting of hawksbill turtles did take place. Export charts for commodities in the 1830s indicate some portion of $6,000 attributed to hawksbill shell, with additional shell imported from the Society, Caroline and Friendly Islands, for re-export to foreign markets (Niles 1837; Ruschenberger 1838:380-381). Currently, only 30 female hawksbill turtles nest in the MHI, primarily on remote beaches on the island of Hawai‘i, and none are known to nest in the NWHI (Balazs et al. 2000).

Predatory reef fish, such as reef sharks,^[[45]](#endnote-45)^ large carangid fish such as jacks and trevally, and grouper and snapper species inhabiting the shallow and deep-slope reef areas were prey items for early Polynesian hunters. For large carnivores, ethnographic evidence suggests that large carnivorous fish were reserved for consumption by elite households, while other small herbivores and carnivores were for the most part unregulated (Buck 1957; Titcomb 1972). The stratification in social access to carnivorous fish is supported by the archaeological record. For example, Kirch & O'Day (2003:494) found, “the fish remains from elite households contained more groupers (Epinephelus spp.), jacks (Carangidae) and sharks (Carcharhinidae), all relatively large carnivorous fish.” In Native Hawaiian culture, sharks are revered as part of the Hawaiian pantheon (Beckwith 1917), and early scholars noted that Native Hawaiians had strict rules about which sharks could be harvested (Beckley 1883; Beckwith 1917; Malo 1951).^[[46]](#endnote-46)^ In summary, the evidence from the archaeological and ethnohistoric literature suggest that harvesting on large carnivorous fish species was regulated with respect to social status in prehistory. Like protections on sea turtles, restrictions on large piscivorous fish were probably codified in the late prehistoric period (> AD 1450) as a response to rarity. It is during this period that labor specialization also enabled the rise of a professional fishing class,^[[47]](#endnote-47)^ which provided a mechanism for ruling chiefs to regulate effort by controlling access to valuable gears, materials and related trades (e.g. canoe manufacturing).

After contact, subsistence fishing activities actually decreased due to the combined effects of depopulation due to foreign diseases (Kirch 2007; Nordyke 1989),^[[48]](#endnote-48)^ the attrition of young, able-bodied Hawaiian men (the primary fishers) to burgeoning maritime industries (principally the whaling trade, from the 1820s-1860s) (Jones 1948; Kuykendall 1938), and the waning of chiefly sponsorship of the professional fishing class (Kuykendall 1938; Miller 1989). Supporting evidence for the decrease in fishing is supported by anecdotal accounts, archaeological data, and historical studies on the commercial fisheries of Hawai‘i (Cobb 1902; Cobb 1905a; Cobb 1905b; McCoy 2008; Wilkes 1845). During this period, decreases in the availability of fish are attributed not to overexploitation but paradoxically to lack of fishing effort which is attributed to the combined effects of Native Hawaiian population loss and decreased emphasis among the ruling chiefs on fishing as an economic activity.^[[49]](#endnote-49)^ The introduction of Western influences, particularly trade and export markets, resulted in chiefs reducing their sponsorship and support of the fishing gear and canoe-building trades (including the construction of gears and canoes for offshore fishing activities) vital to the professional fishing class (Miller 1989).^[[50]](#endnote-50)^ The attrition of young men to maritime trades such as whaling also removed a substantial percentage of the section of the population typically employed in the professional fishing classes and in subsistence fishing from the Hawaiian Islands.^[[51]](#endnote-51)^

Commercial harvesting of predatory fish began in the 1820s with the opening of the first commercial fish markets and stalls (Schug 2001), which gradually ramped up from 1820 to the late 1800s, with markets becoming larger and consolidated in the major port cities on each island. In the historic period from the early to mid-1800s, the inchoate commercial industry focused primarily on supplying local demand for fresh fish and markets were supplied completely by Native Hawaiian fishermen. ‘Flashy’ fishing events like shark and porpoise fishing by Native Hawaiians were recorded by early Western observers (Bingham 1849; Colnett & Galois 2004; Olmstead 1841; Wilkes 1845:117). By the mid-1800s, the marketing and resale of fish were primarily handled by foreigners, including primarily the Chinese and Japanese, the majority of whom were brought to Hawai‘i as laborers for the growing sugar economy (Nordyke 1989). Chinese fish peddlers became the primary marketers and sold fresh reef fish from baskets and carts in the major port cities and beyond (Cobb 1902; Schug 2001).

Several export businesses during this early commercial period (1820-1870) initiated, including an export market for shark fins and liver oil. The shark fishery probably depleted shark species in the MHI by the mid 1800s, which is supported by evidence that a number of shark expeditions were undertaken to the NWHI beginning at least by 1859 (Casserley 1998). Anecdotal accounts in the 1880s confirm that large sharks were indeed rare (Beckley 1883). Even so, a sport fishing operation conducted shark hunts for tourists in the MHI at the turn of the century, and anecdotal accounts from this fishery indicate many large sharks were taken near the city’s offshore dump site, though some large expeditions were apparently unsuccessful (Anonymous 1900; Gaffney 2000). Shark hunting continued in the early 1900s.^[[52]](#endnote-52)^ During this early period piscivorous fish and other reef species were also under pressure from the introduction of destructive fishing practices in the 19^th^ century. Dynamite fishing was eventually outlawed in the early 1900s, and anecdotal information from the late 1800s suggests dynamite fishing was both prevalent and productive in catching large reef fish.^[[53]](#endnote-53)^

By the late 1880s, Japanese moved in larger numbers into the commercial fisheries, and by the early 1900s came to dominate most aspects of the commercial fishery trade. The Japanese integrated Native Hawaiian methods of deep reef fishing (primarily targeting demersal snappers and groupers) with their own methods, including longline gears targeting pelagic species.^[[54]](#endnote-54)^ As a result of the Japanese focus on pelagics, commercial fishery markets shifted from nearshore reef species to pelagic and deep-reef species. This shift was also due to Native Hawaiians leaving the commercial fishery trade, which is attributed to market manipulation by Japanese fishing associations, the lowered economic profitability for fishermen, and lack of access to new gears and technologies (e.g. fishing sampans). When Cobb surveyed the fisheries as part of the U.S. Fisheries Commission delegation in 1900 and 1903, over 80% of the commercial catch consisted of reef species, including large catches of jacks, trevallys, snappers, and other predatory fish. However, only a few decades later, the situation completely reversed, with the vast majority of the catch consisting of pelagics, particularly tunas and billfish.^[[55]](#endnote-55)^

The decline in reef fish species in the late 1800s and early 1900s was highlighted in many reports and articles (e.g. Kelly 1925, 1931; Nakuina 1904; Storm 1940), and was the one of the major subjects addressed by a 1938 inquiry by the U.S. Bureau of Fisheries (Bell & Higgens 1939), along with the alleged monopolization and price-fixing by the Japanese, to which some had attributed the high price of fish in Hawai‘i. Bell and Higgens (1939) described the prevailing sentiments regarding reef fisheries in the late 1930s, including the prevailing sentiment that fish prices were too high (up to five times the cost of fish on the mainland) and that market manipulation by fishing associations and vendors were the principal cause.^[[56]](#endnote-56)^ The Bell and Higgens report highlighted a major issue in Hawaiian nearshore fisheries not explained by coarse statistical reporting, namely, that many favored nearshore species had declined considerably, but that these declines were masked by increases in catches of other, less desirable reef fish species.^[[57]](#endnote-57)^ The Bureau of Fisheries investigators further concluded that overfishing was attributed primarily to rampant harvesting of juveniles, primarily through the widespread deployment of small-mesh gill nets and large seine nets. Native Hawaiian perspectives on fisheries decline often differed significantly from Westerners, and indigenous explanations for decline centered primarily on loss of traditional fishery rights (customary marine tenure) and indigenous management methods that were historically implemented to conserve fish species.^[[58]](#endnote-58),^^[[59]](#endnote-59)^

A brief reprieve in fishing occurred during WWII due to the imposition of martial law after the Pearl Harbor attack on Dec. 7^th^, 1941 (Green n.d., Robinson 2009).^[[60]](#endnote-60)^ Nearshore fishing was off-limits due to closures of beach and reef areas, and oral histories collected in the Hawaiian islands indicate some recovery of fish populations during this period (Maly & Maly 2003a, b, c, d), as do published anecdotal accounts (Anonymous 1947b). The nearshore and offshore fisheries were recapitalized slowly after WWII (Pooley 1993b). Major shark eradication efforts were undertaken starting in 1959 with the death of a high school student (Gaffney 2000) and these efforts were chronicled in detail in newspaper articles from 1959-1971. Shark eradication efforts continued until 1976, resulting in approximately 5,000 sharks harvested in the MHI (Wetherbee et al. 1994). In the decades after WWII, major declines occurred in predatory fish in the MHI, except in some fully protected, no-take reserves. Even in these reserves, predators do not reach the levels observed in the NWHI or in other, uninhabited atolls (Friedlander & DeMartini 2002; Sandin et al. 2008). Catches of large carangids (> 60 lbs), commonly observed even in the early 1900s,^[[61]](#endnote-61)^ are now considered to be worthy of publication in popular fishing media (Desha 2009). This decrease in carangid populations continues despite the increase in ciguatera (Gollop & Pon 1992), and the bioaccumulation of ciguatoxin in carangids and other predatory species is well known to local fishermen. Large carnivorous fish continue to be rare outside of protected and rural areas in the MHI due to a combined history of state-sponsored eradication efforts, unregulated fishing overharvest and land-based pollution affecting reef habitat (Friedlander et al. 2008b; Friedlander et al. 2004; Friedlander & DeMartini 2002; Williams et al. 2008).

E.2 Papahānaumokuākea (Northwestern Hawaiian Islands)

**Table E.2**: Timeline of major events and proximate stressors (1-6) impacting the EcoState for large carnivores in the Northwestern Hawaiian Islands. Proximate stressors are indicated with regard to their intensity: x = low, X = medium, **X** [emboldened] = high.

| **Date (AD)** | **EcoState** | **Description** | **1. Overexploitation** | **2. Invasive Species** | **3. Land-Based Pollution** | **4. Disease** | **5. Climate Change** |
| --- | --- | --- | --- | --- | --- | --- | --- |
| <1250 | 1 | Pristine |  |  |  |  |  |
| 1250-1450 | 1 | No impact |  |  |  |  |  |
| 1450-1778 | 1.5 | Ephemeral subsistence harvesting by small groups of voyaging Polynesians only in the lower region of the NWHI | x |  |  |  |  |
| 1778-1820 | 2 | Visits by indigenous fishers decrease or stop; Western explorers discover NWHI with first exploitation | x |  |  |  |  |
| 1820-1875 | 4.5 | Whaler, sealers and traders exploit the Hawaiian monk seal to near extinction; Harvest of hawksbill turtles and other species by foreign ships and shipwrecked crews | **X** |  |  |  |  |
| 1875-1903 | 4 | Shark fishing expeditions; commercial exploitation by Hawai‘i & Japan fleets; first legal protections enacted | **X** |  |  |  |  |
| 1903-1940 | 4 | Commercial fishing by Hawai‘i-based fleets; Scientific exploration, with increased enforcement of legal protections by US government | X |  |  |  |  |
| 1940-1970 | 4 | Commercial fishing operations by small Hawai‘i-based fleet; subsistence fishing by small human population | X |  |  |  |  |
| 1970-2005 | 3 | Commercial fishing focuses on bottomfishing for deep-reef species and lobster; documented declines in monk seal population | x |  |  |  |  |
| 2005-2009 | 2.5 | Full ecosystem protection extended to marine environment with proclamation as National Monument; bottomfishing phases out by 2011; monk seal still declining | x |  |  |  |  |

As discussed previously, Native Hawaiians were probably accessing the lower region of the NWHI (Nihoa and Mokumanamana [Necker] Islands) after AD 1450 for subsistence harvesting on a limited basis during the prehistoric period, but many factors probably precluded these subsistence trips from being common or having a major impact. In the historic period, the NWHI soon became involved in commercial maritime industries in the Pacific due to its location in the middle of major trade routes between fur and seal grounds in Alaska, the Pacific northwest, and the Kamchatka peninsula, markets in Canton (China) and sealing and whaling grounds east of Japan and in the Bering Sea. This strategic location of Hawai‘i ultimately resulted in Hawai‘i becoming a major provisioning station for commercial maritime traders, including the sealing and whaling industries (Beechert 1991; Miller 1989). Foreign ships, including sealers and whalers exploited monk seals, hawksbill turtles and piscivorous fish in the NWHI starting in the early to mid 1800s and continued through the early half of the 20^th^ century. The first major commercial activities focused on harvesting of the Hawaiian monk seal, but hawksbill turtles, sharks and other large carnivorous fish were also targeted (Hau 1984; Iversen et al. 1990; WPRFMC n.d.). The first whalers arrived in Honolulu in the early 1800s and major sealing expeditions were undertaken in the NWHI starting in the 1840s-1850s, taking hundreds to thousands of seals (Anonymous 1859b; Bailey 1952; Brooks 1859; Cobb 1902). Cobb (1902) describes some of the earliest recorded sealing voyages in the NWHI.^[[62]](#endnote-62)^ Of these early described voyages, it is difficult disentangle which cargoes were derived from sealing ventures outside of Hawaiian waters (e.g. Alaska, the Pacific Northwest, and the California coast) and those which were comprised of monk seal populations in Hawai‘i (Kuykendall 1929). At least two voyages are believed to have targeted the monk seal, including the Gambia (Cobb 1902, Bryan 1914) and an unnamed expedition in the 1890s (Bailey 1952).^[[63]](#endnote-63)^ Records from these voyages and others confirm that the monk seal was extremely rare by the mid to late 1800s.^[[64]](#endnote-64)^ Residents on Laysan Island engaged in guano mining at the turn of the century only occasionally saw monk seals and shipwrecked sailors on Midway Atoll saw none in over 14 months (Bailey 1952; Unger 2003). Few individuals survived into the twentieth century; Schultz et al. (2009) estimates a minimum population size of 23 seals at the nadir of the population bottleneck. While the species experienced a partial recovery by 1960, it is now declining at ~4% per year (NMFS 2007).

Hawksbill turtles were a focus for commercial extraction for shell and for subsistence by shipwrecked sailors. Early expeditions described hawksbills in the NWHI (Morell 1832) and according to anecdotal accounts, the exploitative pressures on hawksbills mirrored those on the green sea turtle. Major shark expeditions were undertaken to the NWHI by at least the late 1800s. In his visit in 1900, Cobb described the shark fishery in the NWHI.^[[65]](#endnote-65)^ Japanese commercial fisheries were also operating in the NWHI during the late 1800s and early 1900s, along with subsistence fishing by guano miners, the trans-Pacific cable company operators at Midway, shipwrecked sailors, feather poachers and scientific expeditions.

In 1903, the first protections were extended to the NWHI when President Theodore Roosevelt sent the Navy to Midway Island to expand US Pacific claims and to end the slaughter of millions of seabirds by poachers for the millinery trade (Executive Order 199A). Further protections were advanced in 1909 when Roosevelt proclaimed the NWHI a bird reservation (Executive Order 1019), and increased trips by US warships decreased the presence of foreign commercial harvesters (principally the Japanese) in the NWHI by the 1910s. Domestic commercial and subsistence fishing operations, however, were exploiting the area by the 1920s, targeting inshore reef species, deep-reef groupers and snappers, large carangids and other reef fish (Apple 1973; Hau 1984; Iversen et al. 1990; WPRFMC n.d.). A large military presence built up in the years leading up to WWII, and thousands of individuals inhabited the NWHI during the war. Human presence decreased substantially starting in the 1950s with demilitarization.

A series of commercial operations operated in the NWHI after 1950 including the bottomfish fishery, a handline fishery, a shortline fishery, and a subsistence fishery (Kittinger et al. 2009; SRGII 2004; Wilcox et al. 2004). The number of commercial fishing vessels operating in the NWHI declined to just a few vessels (< 5) during the 1950s due to low fish prices and vessel losses (Hau 1984). The commercial fishery for predatory jacks and trevally (Caranx spp.) declined precipitously in the 1970s-80s due to concerns about ciguatera poisoning (Rick Gaffney and Associates Inc. 2000). Catches at French Frigate Shoals, for example, declined from 60-70,000 lbs annually in the late 1970s to less than 10,000 lbs from the early 1980s-2009 (DAR 2009). The primary fishery impacting large carnivores during the latter half of the 20^th^ century was the bottomfish fishery, which targets deep-reef snappers and groupers. This fishery reached an apex in 1970s and 80s and decreased in the past few decades to a smaller fleet (~8 vessels) (DAR 2009), and the fishery was ended in 2010 as a consequence of the presidential proclamation establishing the Papahānaumokuākea Marine National Monument (Kittinger et al. 2009). Bottomfish populations are still exploited (Coffman & Kim 2009), hawskbill sea turtles are rare and the Hawaiian monk seal population is in a state of decline (NMFS 2007), but large apex predators are now very abundant in NWHI coral reef ecosystems (Friedlander and DeMartini 2002; Holzwarth et al. 2006).

F. Small Carnivores & Small Herbivores

Many of the same proximate factors that affected large carnivores, particularly reef fish, were similar for small herbivores and carnivores in nearshore marine environments. As such, changes to these guilds are described together, but when important differences arise they are differentiated below.

F.1 Main Hawaiian Islands

**Table F.1**: Timeline of major events and proximate stressors (1-6) impacting the EcoState for small herbivores and carnivores in the Main Hawaiian Islands. Proximate stressors are indicated with regard to their intensity: x = low, X = medium, **X** [emboldened] = high.

| Date (AD) | **EcoState** | **Description** | **1. Overexploitation** | **2. Invasive Species** | **3. Land-Based Pollution** | **4. Disease** | **5. Climate Change** |
| --- | --- | --- | --- | --- | --- | --- | --- |
| <1250 | 1 | Pristine |  |  |  |  |  |
| 1250-1450 | 2.5 | Subsistence use with some evidence of resource depression | x |  |  |  |  |
| 1450-1778 | 2 | Development of traditional management measures; social controls on marine resource consumption for some species, access restrictions & time/area closures | x |  |  |  |  |
| 1778-1820 | 1.5 | Population decrease due to disease epidemics, re-direction of labor away from fishing, and attrition of able-bodied men drives down fishing effort, reef fish and shellfish recover | x |  |  |  |  |
| 1820-1880 | 3 | Commercial fishing develops, and is concentrated in nearshore environment; juveniles harvested | X |  |  |  |  |
| 1880-1925 | 3.5 | Overfishing of nearshore species and juveniles rampant; Destructive fishing practices; First fishery laws passed but are largely ineffective | X | x | x |  |  |
| 1925-1939 | 4 | Favored species rare; U.S. Bureau of Fisheries investigation; high prices at fish markets; erosion of traditional fishery rights | **X** | x | x |  |  |
| 1939-1945 | 3 | WWII; martial law restrictions fishing effort; some recovery of reef fish populations |  | x | x |  |  |
| 1945-1975 | 4 | Overexploitation; Habitat degradation from land-based pollution; introduction and spread of exotic species | X | x | x |  |  |
| 1975-2009 | 4.5 | Rampant overfishing due to lack of licensing, enforcement or adequate conservation measures; large fish restricted to small no-take MPAs and geographic refugia; exotic species proliferate; habitat degradation from land-based sources continues with development of coastal zones | **X** | x | x |  |  |

The first Polynesians arriving in the archipelago focused primarily on foraging for marine resources (Burney & Kikuchi 2006; Kirch 1973, 1979b, 1982; Kirch & Kelly 1975; Morrison & Hunt 2007). Archaeological midden data confirm that small carnivorous and herbivorous fish, including juveniles, and reef invertebrates formed a significant portion of the Native Hawaiian diet, and this is supported by evidence from ethnographic works and anecdotal reports. Favored herbivorous invertebrates included octopus (he‘e), intertidal limpets (opihi), gastropods and other small grazers. Numerous reef fish were also targeted, including herbivorous surgeonfish, tangs, and parrotfish, as well as wide assortment of small carnivorous fish such as mackerel scad, wrasses, snappers, groupers, needlefish, scorpionfish, and highly prized bonefish, mullet and milkfish (Titcomb 1972). As discussed above, widespread evidence of changes in prehistoric societies in Hawai‘i from ~ AD 1450-1600 suggest that relationship of human society to marine resources changed during this period. At the same time, population expansion reached its apex (Dye & Komori 1992; Kirch 2007) and thus harvesting of marine resources was probably at its zenith in terms of the geographic extent in Hawaiian prehistory. This is evidenced by the observed transitions of temporarily inhabited fishing camps to permanently inhabited coastal hamlets (e.g. Kirch et al. 2003; O'Day 2002). Archaeological evidence is equivocal on whether marine resources were overexploited during the prehistoric period,^[[66]](#endnote-66)^ but differences in excavation methods, dating procedures, provenience, analysis methods and data reporting among studies confounds comparison between faunal assemblages.

Fishing effort appears to have decreased after European contact due to declining chiefly sponsorship of the professional fishing classes and related trades, indigenous depopulation due to disease, and attrition of able-bodied young men, the principal sector of the population engaged in fishing. Archaeological evidence, for example, shows that recovery in nearshore shellfish populations is due to demographic collapse (McCoy 2008).^[[67]](#endnote-67)^ Similar recoveries are likely to have occurred in nearshore reef fish populations. Starting in the 1820s, Native Hawaiians supplied the developing commercial fishing industry, and most fishing was concentrated in the nearshore reef areas to supply markets emerging in the major port towns (Beechert 1991; Schug 2001). This is evidenced by archaeological data, ethnographic information, and the first studies on commercial fisheries, all of which show that the nearshore reef areas were the principal foraging areas for Native Hawaiian fishers (Beckley 1883; Cobb 1902; Cobb 1905a; Cobb 1905b; Goto 1986; Kirch 1982; Malo 1951; Newman 1970; Titcomb 1972).

The first laws concerning fishing were enacted in the late 1800s in response to over-exploitation from commercial and subsistence fishing practices.^[[68]](#endnote-68)^ By the early 1900s, destructive fishing practices, commercial harvesting in nearshore reef areas, and large subsistence efforts combined to make many small herbivorous and carnivorous species relatively rare. Destructive fishing practices included:

1. Bleach and dynamite fishing introduced in the 1880s, and banned in the early 1900s (Anonymous 1901, 1905);
2. Introduction and pervasive use of new seine nets and gill nets with small mesh size that resulted in rampant harvesting of juveniles, particularly to stock fishponds and procure baitfish for pelagic fisheries (Cobb 1902; Cobb 1905a; Cobb 1905b),
3. Erosion of private marine tenure rights in coastal fisheries, which were traditionally the property of local resource managers that had the right to exclude outsiders (Anders 1987; Kittinger 2009; Kosaki 1954; Meller 1985); and,
4. Erosion of traditional management practices, including seasonal and spatial closures, many of which were designed to protect key species during spawning seasons (Nakuina 1904; Clifford 1991).

Other practices that impacted nearshore species include the overexploitation of small reef-associated planktivorous fish (e.g. herrings, anchovy) for use in pelagic fisheries. In 1899, prior to the rapid development of the skipjack tuna industry, baitfish were still quite common.^[[69]](#endnote-69)^ Native Hawaiians had traditionally fished these schools, many of which arrived either seasonally or exhibited stochastic periodicity, for food fish.^[[70]](#endnote-70)^ Immense schools of planktivorous fish described in anecdotal reports were decreased substantially by the baitfish fishery supplying the skipjack tuna fleet that emerged in the early 1900s. Several more fishery laws were enacted the early nineteen hundreds, for example, protection from harvest of female lobsters in 1917 (Anonymous 1917), which were depleted by the early 1900s. Further evidence of commercial overharvest was evidenced in reviews of commercial fishery products and markets, lamented in public newspapers and magazines (e.g. Buckland 1908; Kelly 1925) and the source of a US Bureau of Fisheries investigation in the 1930s (Bell & Higgens 1939).^[[71]](#endnote-71)^

The first quantitative fisheries evaluations were conducted by the US Fish Commission in 1900 and 1902 (Jordan & Evermann 1902; Jordan et al. 1905). John N. Cobb, a statistician with the commission, compiled detailed reports of the commercial fisheries markets which were located at the principal ports on each of the MHI (Cobb 1902; Cobb 1905a, b). Coarse statistics were reported from 1927-1938 by the Division of Fish and Game, under the Board of Commissioners of Agriculture and Forestry, Territory of Hawai‘i (Board of Fish and Game Commissioners 1927; Division of Fish and Game 1927-1936). Starting in 1948, the Division of Aquatic Resources, under the Board of Land and Natural Resources began to collect more detailed reports on reef organisms (DAR 2009). Analysis of these fisheries records reveals major declines in favored small herbivorous and carnivorous reef species, with some favored species decreasing by over an order of magnitude over the past century (Friedlander 2004; Shomura 1987, 2004). Shomura (1987) found a dramatic decline in inshore species from “embayments, nearshore areas, and reefs,” which decreased from 3.6 million pounds in 1900 to 0.6 million pounds by the mid 1980s. These declines have been attributed to rampant overharvesting with little regulation and weak enforcement, land-based pollution, technological innovations that have decreased natural refugia^[[72]](#endnote-72)^, the introduction of exotic species (e.g. the blue-lined snapper, Ta‘ape; Samoan crab) and shifting social preferences, which have altered the composition of commercial catches over the past century. Currently, reefs supporting the most robust populations for small herbivorous and carnivorous species are now restricted to geographic refugia away from population centers and in some small marine protected areas (Friedlander et al. 2008b; Friedlander et al. 2004; Friedlander & DeMartini 2002; Williams et al. 2008).

F.2 Papahānaumokuākea (Northwestern Hawaiian Islands)

**Table F.2**: Timeline of major events and proximate stressors (1-6) impacting the EcoState for small carnivores and small herbivores in the Northwestern Hawaiian Islands. Proximate stressors are indicated with regard to their intensity: x = low, X = medium, **X** [emboldened] = high. *applies to small carnivores only.

| **Date (AD)** | **EcoState** | **Description** | **1. Overexploitation** | **2. Invasive Species** | **3. Land-Based Pollution** | **4. Disease** | **5. Climate Change** |
| --- | --- | --- | --- | --- | --- | --- | --- |
| <1250 | 1 | Pristine |  |  |  |  |  |
| 1250-1450 | 1 | No impact |  |  |  |  |  |
| 1450-1778 | 1.5 | Mokumanamana (Necker) and Nihoa islands occupied with subsistence fishing | x |  |  |  |  |
| 1778-1820 | 1.25 | Visits decrease or end due to Native Hawaiian depopulation; first Western voyages of exploration and re-discovery | x |  |  |  |  |
| 1820-1880 | 2 | Foreign ships exploit nearshore reef zones; shipwreck survivor camps; occasional visits Mokumanamana and Nihoa by people from Ni‘ihau, Kaua‘i for subsistence harvesting | x |  |  |  |  |
| 1880-1915 | 3 | Commercial fishing by Japan, Hawai‘i-based fleets; subsistence fishing by guano miners, cable operators, feather poachers | X |  |  |  |  |
| 1915-1950 | 3 | Hawai‘i-based commercial fishing operating in NWHI; large human population during WWII | X |  |  |  |  |
| 1950-1975 | 3 | Commercial fishing with air transport to MHI markets | X | x* |  |  |  |
| 1975-2000 | 2.5/3* | Heavy fishing for bottomfish & lobster; lobster stocks crash | x | x* |  |  |  |
| 2000-2009 | 2/2.5* | Commercial bottomfishing only, lobster stocks do not recover; subsistence use by visiting Native Hawaiians, scientists | x | x* |  |  |  |

In the prehistoric period, small human populations living on Nihoa and Mokumanamana (Necker) Islands were probably engaged in subsistence fishing, but it is unlikely these activities had an impact at the regional scale of the NWHI. As discussed earlier, after Western contact the islands were subject to very little use by Native Hawaiians except for summer visits to Mokumanamana and Nihoa by small groups of people from Ni‘ihau and Kaua‘i. Western explorers made re-discoveries of the islands in this region starting in the late 1700s up through the mid-1800s. Commercial exploitation of the area started in the early to mid 19^th^ century, but was primarily restricted to megafauna and a few other key species such as shark and bêche-de-mer. Subsistence fishing by explorers and other visitors, including shipwrecked sailors, occurred from 1820-1880s. Hawai‘i- and Japan-based commercial fishing fleets began exploiting the NWHI in the late 19^th^ century. Reports on species targeted by these fleets are unavailable, but the available evidence suggests they were seeking a wide variety of commercially valuable species, including reef fish, crustaceans and other species (Iversen et al. 1990; Hau 1984; WPRFMC n.d.). During this period, subsistence fishing was also ongoing by shipwrecked sailors in survivor camps, guano miners, feather poachers, and operators of the trans-Pacific cable station at Midway. By 1925, the US Navy had effectively excluded Japanese fleets from the NWHI, but military presence increased and Hawai‘i-based commercial fishing crews were operating in the area (Iversen et al. 1990). Commercial fishing continued after WWII, and included air transport from French Frigate Shoals to the commercial markets in Honolulu (Apple 1973; Iversen et al. 1990), but fleets operating in the NWHI were relatively small after 1950 (Hau 1984).^[[73]](#endnote-73)^ Fleets increased in size in the 1970s and 1980s but were primarily targeting bottomfish species and lobster populations. The lobster fishery was heavily fished in the late 1970s and early 1980s, resulting severe depletion from which it has never recovered (Pooley 1993a, b). The last remaining fishery in the NWHI is the limited-entry bottomfish fishery, which includes less than a dozen vessels that target a complex of deep-reef snappers and grouper species; this fishery will be phased out in 2011 in accordance with the establishment of the Papahānaumokuākea Marine National Monument. Though some species in the NWHI have not recovered from intensive exploitation (e.g. lobster populations at Nihoa & Mokumanamana [Necker] Islands) (DiNardo and Marshall 2001), ecological surveys initiated in the 1970s have shown that most species comprising the small herbivore and small carnivore guilds are intact (Grigg and Pfund 1980; Grigg and Tanoue 1984; Friedlander and Demartini 2002; DiNardo and Parrish 2006).

2. Footnotes for Summary Narratives By Guild

1. “Evidence of erosion has come… from studies on Kaho‘olawe, where burn layers associated with extinct land snails date to the sixteenth century A.D. mark the beginning of a phase of erosion (Hammatt 1978, Hommon 1980). As Hommon writes: ‘During the 1500-1550 period, when the estimated inland population reached its maximum, it is evident that massive erosion was beginning ... extensive [prehistoric] use of the land led directly and indirectly to major erosion’ (1980: 7-63). If, as these cases suggest, erosion was occurring on a fairly widespread scale by the fourteenth through sixteenth centuries, it is probable that deposition of sediments in valley bottoms and along coastlines was correspondingly accelerated.” (Kirch 1982b) [↑](#endnote-ref-1)
2. According to Alexander, the Honolulu fort: “was nearly square, measuring three hundred to four hundred feet on a side, with walls about twelve feet high and twenty feet thick, built of coral rock, with embrasures for cannons” (Alexander 1899) [↑](#endnote-ref-2)
3. “The blocks of coral-rock, somewhat porous, but strong enough to make good walls, being cut out with axes, pried up, trimmed by pattern to their proper form and size, and weighing from two hundred to twelve hundred pounds, were laid up in good lime mortar most of the work being done by native hands... The coral for making the lime they procured by diving in two or three fathom water and detaching blocks or fragments. If these were too heavy for the diver to bring up to his canoe with his hands he ascended to the surface to take breath then descending with a rope attached it to his prize and mounting to his canoe heaved up the mass from the bottom and when the canoe was thus laden rowed it ashore and discharged his freight. By this process they procured about thirty cubic fathoms or 7,776 cubic feet. To burn this mass the church members brought from the mountain side upon their shoulders forty cords of wood. The lime being burned the women took it in calabashes or large gourd shells and bore it on their shoulders to the place of building also sand and water for making the mortar. Thus about 700 barrels each of lime sand and water making about 2,000 barrels equal to 350 wagon loads were carried by women a quarter of a mile to assist the men in building… ” (Bingham 1849:573-574). [↑](#endnote-ref-3)
4. “The coral reefs stretching out from the shore some distance into the ocean are of great value… From these reefs the materials that compose the best and most public buildings in the town are procured simply by hewing them out with axes while in a wet state. It has been estimated that these reefs fronting the town contain materials that would build a city capable of containing 150,000 inhabitants.” (Bates 1854:34). [↑](#endnote-ref-4)
5. Upon visiting the Hawaiian Islands in 1885, the biologist Alexander Agassiz commented, “The elevated reefs of the Sandwich Islands, although not elevated more than twenty to twenty-five feet, are extensively quarried as limestone for building purposes, especially those parts of the reef which evidently formed its inner portion..” (Agassiz 1889). [↑](#endnote-ref-5)
6. “the Hawaii of the good old days was a land of kindly people… of a sea swarming with fish, taken by exploding a stick of dynamite in the water, an expedient that half-filled a boat with fish ranging from sixty-lb ulua [jacks, trevally] to the diminutive ala-lau-wa [aweoweo, Hawaiian big-eye], a little fellow of reddish color, delicious when fried.” (Farrell 1928) [↑](#endnote-ref-6)
7. “This method of fishing [with dynamite] is prohibited by law, but very little attention is paid to this enactment, as nobody seems to bother about enforcing it.” (Cobb 1902:412). [↑](#endnote-ref-7)
8. Oral History Interview with Charles K. Reiny (CR) February 4, 1998 at Mākua on Wai‘anae District Fisheries, O‘ahu, Hawai‘i by Kepā Maly (KM):

   CR: [describing fishery conditions prior to 1940 on the Wai‘anae coast (Mākua-Kahanahāiki area), O‘ahu Island, Hawai‘i] Fishing today is nothing like what it was; the catch has dropped significantly:

   KM: How would you compare the fishing then and now?

   CR: Oh, no comparison. This is nothing. Before, you have one piece of net, you’d get a whole cooler full of all kinds of fish; ‘"weoweo, moi, mullet. Now, you have to lay four or five pieces of net and sometimes, you catch only about eight, nine, ten fish, sometimes. You know what I mean?

   KM: Hmm.

   CR: It’s not like before. They brought this fish over here, the taape [Lutjanus kasmira, introduced blue-line snapper], and they’re eating all the baby eggs. And that’s why, when they bring stuff in, they should study what that thing is going to do.

   (Maly & Maly 2003d:1421)

   Oral History Interview with Walter Keli‘iokekai Paulo (WP) July 14th, 2002 and March 5, 2003 at Miloli‘i, O‘ahu, Hawai‘i by Kepā Maly (KM):

   WP: [describing changes in fisheries since the 1930s and 1940s] “Long line catch has diminished from the 1930s to the present-day…So you can see every fish usually about 150 to 200 pounds. If it’s big eye you probably have about 225 to 300 pounds. That was really good fishing. Today, the long line fisherman might go out, like the ones from Japan, the ones down at Kewalo at present. They would use maybe 2- or 3,000 hooks and their per hundred hook catch now is about two fish per hundred hooks.”

   (Maly & Maly 2003d:258) [↑](#endnote-ref-8)
9. “To accommodate seaplanes, the adjacent coral was dredged to make a seaplane runway 1,000 feet wide and 8,000 feet long. To land supplies, a 12,000 foot long, 2000 foot wide ship channel was dredged to 20 foot depth. About 660,000 cubic yards of dredged coral was added to Tern [Island] to make an airfield 250 feet wide and 3,100 feet long” (Bryan Jr. 1942).; “At FFS [French Frigate Shoals], the 11-acre Tern Island is converted into a 42-acre naval airstrip.” (WPRFMC N.D.) [↑](#endnote-ref-9)
10. Cobb (1902) notes, “The mounds of oyster and other shells found at various places around.. [Pearl] harbor indicate that oysters were a favorite and common article of food many years ago.” [↑](#endnote-ref-10)
11. Anderson formed the Hawaiian Sea Products Company to exploit pearl oysters in the NWHI and, “on Dec. 3, 1928 filed an application with the U.S. government for a permit to build a fishing station and construct several buildings at the atoll. They were granted permission and over the next few years developed pearl beds, and shipped several tons of pearl shells back to Honolulu” (Casserley 1998). [↑](#endnote-ref-11)
12. Sea turtles are slow to mature to reproductive age; they breed only every 3-5 years thereafter. Turtles exhibit a high fidelity to nesting beaches even if threats persist and juveniles experience high mortality. Sea turtle populations can be reduced or eliminated from nesting beaches rather quickly, on the order of decades (Bjorndal & Jackson 2003; Chan & Liew 1996; Musick 1999), but populations can also recover if protected for decades (Balazs & Chaloupka 2004). Because turtles display a high fidelity to both natal nesting beaches and foraging habitat (Avise & Bowen 1994), eradication of nesting beaches renders these sites slow to recover, or becomes permanent, if pressures on nesting females are not removed (McClenachan et al. 2006). [↑](#endnote-ref-12)
13. In Hawai‘i no early archaeological sites contain dense turtle bone in the earliest midden layers, but few data on archaeological midden remains that post-date the earliest settlement period (~AD 1250-1300) exist. Excavations throughout the MHI for later prehistoric periods (> AD 1350) have yielded turtle bones as a low percentage or weight of the total midden (Gordon 1993; Kirch 1979a; Newman 1970; Rosendahl 1994; Soehren n.d.; Tomonari-Tuggle et al. 2000) (Table S4). [↑](#endnote-ref-13)
14. The imposition of cultural controls on resource consumption, including sea turtles, is likely to have developed later in Hawaiian prehistory, from AD 1400-1600, which witnessed the rise of major polities and the development of socio-political complexity, including social stratification. These sanctions included prohibitions on consumption of turtle by women (Bryan Jr. 1938:67; Malo 1951:29) and reservation of turtles for consumption only by chiefs and high priests (Kalakaua 1888:32, 303). [↑](#endnote-ref-14)
15. James Cook, in his visit in 1778, remarked that he was told of some low, uninhabited islands in the vicinity of the MHI called “Tammata pappa” (Beaglehole 1967). Later on, King (Cook’s replacement after his death) provided more clarity: “To the WSW of Teula [Ka‘ula, a small island near Kaua‘i], they visit a low sandy island for sea birds and turtle called Modoo-papapa or Komodoo papapa.” In a similar note, King states, “One canoe belonging to some Atoui [Kaua‘i] Chief…went towards the Island Outoura [Lehua, an island near Kaua‘i]…Their business, they told us, was to catch red birds [frigate & tropic birds], and the next day they intended going to Tomogoopapappa for Turtle” (Beaglehole 1967). The names recorded by Cook and King, now written as moku-pāpapa, translates roughly to mean a low, flat island, as a reef (Pukui & Elbert 1986:318; Pukui et al. 1974:156, in Kikiloi 2010:90). The name moku-pāpapa is a generic name but also refers to a specific place name in some chants and legends. Kikiloi (2010:90) has identified the place name Moku-papapa in the context of indigenous geneaologies of the Hawaiian Islands as “a small submerged seamount” near Kaua‘i that is “a peripheral main Hawaiian Island off Ka‘ula islet” named Five Fathom Pinnacle. It is likely that the Hawaiians encountered by Cook and King were referring to this specific area. Moku-pāpapa may also represent way stations (perhaps in the Line Islands) on the voyaging routes to Kahiki [Tahiti]. The name has also been used to refer to both Nihoa Island (by the archaeologist K. Emory) and Kure Atoll (by King Kālakaua, in 1886) (Rauzon 2001). However, in the manner in which it was used in interactions with Cook and King, Native Hawaiians were almost certainly referring generically to submerged reefs in the vicinity of Kaua‘i or Ni‘ihau as the individuals observed were in a small canoe that was probably not capable of making the long trip to islands beyond Mokumanamana, e.g. French Frigate Shoals, where turtles are known to nest in the NWHI. This is supported by other evidence, including a description in Iversen et al. (1990:22) derived from Chun (1986), in which an informant described a similar area in the vicinity of Ni‘ihau that “abounds in turtle.” [↑](#endnote-ref-15)
16. “Nesting of Green Turtles took place on some of the main Hawaiian Islands just 40 years ago [~1930s]. Documented sightings of turtles coming ashore were made at: Mokapu Peninsula, Kailua and Makapuu Beaches on Oahu; the West coast of Molokai and the North shore of Lanai. It seems reasonable to assume that sites also existed on Kauai, Maui, Kahoolawe and Hawaii, however the author has been unable to obtain substantiated reports of past nestings on these islands. Today no animals are reported nesting on any of the main Hawaiian Islands. If any still do, it would be so infrequent as to best be left unreported because of possible human interference.” (Balazs 1973:129) [↑](#endnote-ref-16)
17. Currently over 90% of green sea turtles now nest at French Frigate Shoals in the NWHI, with the remainder nesting at sparsely inhabited beaches away from urban centers in the MHI (Balazs & Chaloupka 2004). The contemporary ecological data on the distribution of nesting habitat is consistent with expectations of a population reductions and eradication of natal beaches in the MHI. The NWHI and sparsely inhabited areas in the MHI have functioned as geographical refugia for green sea turtle nesting populations due to their isolation and low densities of human foragers, and because many of these sites were not invaded by exotic species, including the Polynesian rat (Hunt & Lipo 2009). Because green sea turtles display a high affinity for natal beaches, it is believed they are slow to colonize new nesting habitat and are unlikely to establish new nesting behaviors if deterring stressors remain. Remaining nesting beaches in the archipelago thus represent the last viable habitats for nesting green sea turtles. [↑](#endnote-ref-17)
18. Prime nesting habitat for green sea turtles, coral sand beaches, is probably not a factor in explaining the current nesting habitats that are currently used by turtle populations. Coral sand beach systems in the MHI were common 2000 years before present and probably represented better habitat than beaches in the NWHI, as these systems were much wider and exhibited active dune systems which are absent today due to the effects of invasive vegetation and other human modification (Fletcher 2009). 2000 years ago sandy beaches were distributed similarly to current locations, whereby beach areas are more common in the MHI than the NWHI due to the larger emergent land areas in the MHI available for beach building, though differences do exist at smaller spatial scales due to volcanic activities, island age, and other geologic processes (Fletcher et al. 2008). [↑](#endnote-ref-18)
19. Introduced species, including the Polynesian rat (Rattus exulans), dog (Canis familiaris) and pig (Sus scrofa), may have played an important role in reducing sea turtles early in Hawaiian prehistory. Rats are known to prey on both sea turtle eggs and hatchlings (Balazs 1983; Caut et al. 2008, 2009), and increase rapidly when introduced into a pristine environment (Drake & Hunt 2009; Holdaway 1999; Thorsen et al. 2000). Population rates of increase for initial colonizing rats would have been much faster than the rate of increase in their human counterparts. The effects, therefore, of rats are much more rapid and geographically dispersed than those of the initial colonizing Polynesians, a pattern that has been observed in the context of terrestrial environmental change in Polynesian prehistory (Athens 1997, 2009; Drake & Hunt 2009; Hunt 2007). [↑](#endnote-ref-19)
20. Observations from other regions, including well-documented prehistoric declines in the Caribbean, indicate that this pattern is a widely observed phenomenon in human prehistory (Frazier 2002; Jackson 1997; McClenachan et al. 2006). Estimates from the Caribbean indicate that even after some recovery following listing under the Endangered Species Act, turtle populations constituted only 3-7% of pre-exploitation abundances (Bjorndal & Jackson 2003; Jackson 1997; Lotze et al. 2006; McClenachan et al. 2006; Van Houtan & Bass 2007). [↑](#endnote-ref-20)
21. “The historian Kamakau states that when Kamehameha learned of the value of [sandalwood], ‘he ordered men to go out in the mountains…to cut sandalwood, and he paid them in cloth and bark for making native cloth, as well as with food and fish…Because the chiefs and commoners in large numbers went out cutting and carrying sandalwood, famine was experienced from Hawaii to Kauai..The pople were forced to eat herbs and fern trunks, because there was no food to be had. When Kamehameha saw that the country was in the grip of a severe famine, he ordered the chiefs and commoners not to devote all their time to cutting sandalwood, and also proclaimed all sandalwood to be the property of the government. Kamehameha then turned and orderd the chiefs and the people under them to farm.’ “ (Kuykendall 1938:88-89) [↑](#endnote-ref-21)
22. After Kamehameha I’s death in 1819, control over sandalwood was decentralized among the ruling chiefs, who re-directed their populations toward sandalwood harvesting to acquire Western goods. This occurred in conjunction with a financial crises in the US, which spurred New England based traders to further develop the sandalwood market in Hawaii for trade with China. Anecdotes compiled by Kuykendall (1938:89-90) confirm how the chiefly re-direction of labor resulted in less attention to traditional farming and fishing, as sandalwood harvesting became the major economic activity promoted by Hawaiian chiefs:

    “March, 1822. A missionary and some assistants returning to Honolulu from Pearl river. They had taken no provisions, expecting to buy from the natives as they went along. ‘But they found the people very poor, and it was with much difficulty that they could obtain any food of the natives, and then only by paying three times its value. The reasons why provisions are so scares on this island is, that the people, for some months past, have been engaged in cutting sandalwood, and have of course neglected the cultivation of the land. Vegetables are sold at a very deer rate.’

    Spring or summer of 1822. Oahu. ‘One one occasion we saw two thousand persons, laden with faggots of sandalwood, coming down from the mountains to deposit their burthens [sic] in the royal store-houses, and then depart to their homes, wearied with their unpaid labours, yet unmurmuring at their bondage.’

    April, 1830. Kauai. From the journal of Mr. Gulick: ‘Felt distressed and grieved for the people who collect sandalwood. They are often driven by hunger to eat wild and bitter herbs, moss, &c. And though the weather is so cold on the hills that my winter clothes will scarcely keep me comfortable, I frequently see men with no clothing except the maro. Were they not remarkably hardy, many of them would certainly perish.’ “ (Kuykendall 1938:89-90). [↑](#endnote-ref-22)
23. The attrition of young men resulted in less fishing effort, as these individuals comprised the primary fishers in professional fishing classes; attrition became such an issue that the Hawaiian monarchy required whaling companies to post bonds with the Hawaiian government for each man that was hired on a whaling vessel. An account from John C. Jones describes the engagement of Hawaiians in the whaling trade, both as sailors on American-owned ships, and as purchasers of U.S. ships: “A practice has for many years existed with the commanders of ships touching the Sandwich Islands either for supplies or trade, to turn on shore all seamen against whom they could allege any trivial misconduct, and employ in the lieu natives of the Islands, by this means lessening their passage bills, but depriving their country of valuable subjects. American vessels have been sold to the natives and their crews discharged, without any means of support, thus left to the protection and mercy of the rude savage…These abuses of the laws of the United States I have been able to remedy.” [emphasis added] (Jones 1948) [↑](#endnote-ref-23)
24. Visits by voyaging Hawaiians to the NWHI may also evidenced by a fresh calabash, or gourd, found in 1805 by the Russian explorer Urey Lisiansky, on a beach of the NWHI island namesake (Lisianski Island), near French Frigate Shoals: “[I] found on the beach a small calabash, which had a round hole cut on one side of it. This could not have been drifted from a great distance, as it was fresh and in good preservation” (Lisiansky 1814). Much has been attributed to the discovery of this calabash, but it may have simply drifted to Lisianski Island from another island in the archipelago. [↑](#endnote-ref-24)
25. “An interview was held with Mr. Bruce Robinson, whose family owns Ni‘ihau Island, in order to locate kupuna [Native Hawaiian elders] who might be able to recount Ni‘ihau bottomfishing practices before the modern fishery era, but Mr. Robinson reported that such kupuna do not exist today. Mr. Robinson reported that during the period from about 1915 to 1925, the oral tradition of past fishing practices carried on by Ni‘ihau residents was broken, and that today’s kupuna on Ni‘ihau do not have a recollection of past fishing practices. He said that Ni‘ihau residents did have the capability to travel to Ka‘ula and Nihoa Islands via canoes, and that a tradition exists that some people from Ni‘ihau would spend three months in the summer on Nihoa Island until the late 1800s.” (Iversen et al. 1990:23) [↑](#endnote-ref-25)
26. “When this rock [Nihoa Island] was first discovered in 1788 there were on board the Prince of Wales some of the natives of Attowai [Kaua‘i] who expressed great surprise that there should be land so near to their islands it lying from Onehow [Ni‘ihau]…at the distance of 39 leagues only and of which not only themselves but all their countrymen were totally ignorant... This intelligence was communicated on their return in the autumn of that year and it excited in the active mind of Taio [a Kaua‘i chief] a strong desire to pay it a visit to establish a colony there and to annex it to his dominions but on his being made thoroughly acquainted with its extent and sterility by the officers of that vessel his project was abandoned. Those people however recognize it under the appellation of Modoo Mannoo [Mokumanu] that is Bird Island and from its great distance from all other land and its proximity to their islands it seems to claim some distant pretensions to be ranked in the group of the Sandwich islands..” (Vancouver 1801) [↑](#endnote-ref-26)
27. “They found some companions that the rough sea had induced to seek the shelter of the lagoon and beach. They were large sea turtles, and he and his crew turned them on their backs to prevent their escape. Today we have them added to our food supply and they are very welcome..” (Read 1912) [↑](#endnote-ref-27)
28. “On one of our explorations we found a plank…which we instantly recognized as part of the Wandering Minstrel wrecked at Midway…The site of the restaurant was unmistakable; they must have enjoyed turtle, to judge from the hundreds of the upper shells we found there. From their account of their experience they had a gay and festive time, and brought away dried fish and shark fins enough to start them in life again.” (Walker 1909) [↑](#endnote-ref-28)
29. “The brig Rodolph under Capt. Perry arrived at [Pearl & Hermes] atoll on Aug. 11, 1850. The Rodolph's purpose was to search for and collected [sic] sea turtles. When Capt Perry and three of his crew landed on one of the small islands, he found only one turtle, but a large number of nesting birds and seals. They killed 12 seals for food, and departed shortly thereafter…In 1882, Laysan was visited by the fishing schooner, Ada. From Jan 26-30, the crew collected 104 turtles, and 207 bêche-de-mer. On this visit the crew found a sign with an ‘appeal to voyagers not to take the turtle away.’ The crew revisited May 3rd and took 26 additional turtles.” [↑](#endnote-ref-29)
30. According to Elschner (1915), “As on all these islands, the meat of the turtles serve as a delicious meal for the few inhabitants of the atolls and the crews of boats.” [↑](#endnote-ref-30)
31. “After World War II, tern islet [on French Frigate Shoals] became the base of joint operations of the Seaside Fishing Company and the Hawaiian-American Fisheries Company. Native Hawaiian fishes were their specialties, but more than 200 turtles were captured and flown to Honolulu from Tern, a major factor in their depletion. The three year profit, 1946-1948, for the company was $19,500 of a net of $73,400. Commercial operations continued intermittently through 1959.” (Apple 1973) [↑](#endnote-ref-31)
32. Contemporary work on the monk seal has shown that monk seals are highly sensitive to the presence of both humans and dogs, which have a significant effect on monk seal behavior: “At Midway, dogs often accompanied their masters when they walked on beaches. Dogs were excted by monk seals and harassed them by biting at the rear flippers until the seals succeeded in escaping into the sea. Seals deserted hauling and pupping beaches where they were frequently disturbed” (Kenyon 1972). Kenyon (1972) showed that repeated disturbance by small groups of humans and dogs resulted in increased juvenile mortality and caused monk seals to desert beaches at preferred habitats in the NWHI. If monk seals were subjected to the synergistic pressures of introduced dogs and human harvesting and harassment, these slow-reproducing, long-lived species would have been locally extirpated rapidly, on the order of a few decades (Musick 1999; Ragen 1999). [↑](#endnote-ref-32)
33. The documented evidence of prehistoric harvesting of monk seal is restricted to one specimen in an upland archaeological site (Rosendahl 1994), but mammal bones in many of the excavated middens in Hawaii have yet to be thoroughly enumerated by species. The scant evidence for monk seals in the archaeological record indicates either early and rapid reduction of monk seals in the MHI, pre-human rarity, lack of human harvesting by Polynesians, or that populations were restricted to the NWHI in prehistoric times. Based on the evidence, it is more likely that the lack of monk seal in middens is due to extreme rarity due to human harvesting and possibly harassment and deterrence from haul out beaches by Polynesian dogs. The monk seal is estimated to have inhabited the archipelago for around 14 million years and thus have adapted to long-term geologic changes in the archipelago. It is unlikely given their life history traits populations were restricted to the NWHI. An alternative explanation is that depositional processes may have obscured seal remains in middens. Monk seals, which weigh hundreds of pounds, may not have been transported to permanent habitation sites before slaughter. Prehistoric hunters were more likely to butcher animals at the kill site and transport the most valuable meat portions back to habitation sites, a phenomenon known in archaeological sciences as the “schlepp effect,” which serves to explain the distribution of large animal bones in midden contents (Turner 1989). Evidence from New Zealand suggests that seal flesh was separated from bones prior to preservation for long distance transport (Smith 1985, 1989). Another potentially confounding factor is that pre-depositional ravaging by Polynesian dogs or pigs raised in animal husbandry may explain the lack of monk seal remains in middens (Marean et al. 1992). The extreme rarity of monk seal in Hawaiian middens is however difficult to reconcile with existing studies in New Zealand and the Pacific Northwest where prehistoric human hunting of seals is recorded in the midden record (Etnier 2007; Nagaoka 2002; Smith 1985, 1989, 2005). This leaves the remaining explanation that perhaps prehistoric inhabitants of the Hawaiian Archipelago did not harvest monk seal, which is why it is not present in middens. This explanation is highly unlikely given the existing evidence of seal bones in middens in New Zealand and the Pacific northwest, which points broadly to early peoples as opportunistic foragers; in addition these studies show widespread evidence of human hunting as a major driver for prehistoric reduction in seal populations in these coastal settings (Anderson & Smith 1996; Etnier 2007; Nagaoka 2002; Rick et al. 2005; Rick et al. 2008; Smith 1979, 1985, 1989, 2005; Torben et al. 2009; but see Etnier [2007], who has suggested that harvest of pinnipeds in the Pacific Northwest may have been sustainable). By the historic period, the remaining monk seals were sequestered in the NWHI and the scant bone found in archaeological sites is evidence that errant individuals occasionally migrated down to the MHI were they were harvested opportunitistically (see anecdote below); this pattern is consistent with current observations of dispersal between the NWHI and MHI (Baker & Johanos 2004). [↑](#endnote-ref-33)
34. It is often noted that there is no known name in Hawaiian for the monk seal (though a modern name, ‘īlioholoikauaua, has been given to the monk seal). Though a pre-contact name may still be waiting to be discovered in extant Hawaiian literature and cultural knowledge, the lack of a cultural identity or knowledge base for monk seals name may also constitute evidence of rapid and early reductions in the MHI, as these animals were likely quickly reduced before they became well known to later generations. [↑](#endnote-ref-34)
35. “a sick or helpless seal was caught by the natives in Hilo Bay, Hawaii, towed ashore, killed and eaten. Unfortunately I was too late to secure any part of the animal for identification, but the natives assured me that solitary seals occurred on the coast about once in 10 years or so. They were very curious and asked many questions as to the habitat of the animal, its nature, food, and habits, about which they knew nothing.” (H.W. Henshaw, quoted in (Bailey 1952:5) [↑](#endnote-ref-35)
36. Prehistoric monk seal populations in Hawaii have been estimated to have only reached numbers in the thousands (Ragen & Lavigne 1999), which is consistent with pre-exploitation estimates for densities of the Caribbean monk seal (McClenachan & Cooper 2008). This small estimated population size contrasts greatly with populations of seals in New Zealand and the Pacific northwest. Seal populations in these areas are estimated to be at least an order of magnitude larger that the estimates for pre-human populations of Hawaiian monk seals, and the prehistoric ranges and habitat of these seals far exceeded that for monk seals in the Hawaiian archipelago. In New Zealand, for example, seals were second only to fish as a source of meat in Maori subsistence diets (Smith 2004). The small population size of Hawaiian monk seals translates to higher vulnerability to human exploitation and stressors from introduced species. [↑](#endnote-ref-36)
37. “We test the hypotheses of selective sweep, historical bottleneck and persistently small population size to explain extremely low genetic diversity in the critically endangered Hawaiian monk seal (Monachus schauinslandi). Of 163 microsatellite loci isolated from the species’ genome, only 17 are polymorphic. Mapping 98 monomorphic and 12 polymorphic loci to 35 chromosomes throughout the dog genome, we reject the selective sweep hypothesis. Genotyping 2423 Hawaiian monk seals at the 17 polymorphic loci plus a locus previously isolated from another pinniped species, we find evidence for a recent bottleneck (P = 0.04). This is consistent with historical records describing intense hunting in the 19th century; however, the bottleneck was not of sufficient severity and duration to explain the genome-wide depletion of genetic diversity (H = 0.05; A = 1.1). Long-term population size restriction is a more likely explanation.” (Schultz et al. 2010) [↑](#endnote-ref-37)
38. Among the primary habitats required by monk seals are shallow water reef habitat for pupping, weaning and foraging, sandy beach areas for hauling out, and deeper reef areas for foraging (Kenyon & Rice 1959; NMFS 2007). These habitats are common in the MHI (Juvik & Juvik 1998), and the higher prevalence of sandy beach areas in the MHI, along with abundant shallow water habitat for reproduction and reef foraging areas in the MHI would have provided prime habitat for Hawaiian monk seals in prehistoric times. [↑](#endnote-ref-38)
39. Upon arrival in 1778, James Cook noted the abundance of dogs in the island: “Judging from what we saw growing and from what was brought to market there can be no doubt that the greatest part of their vegetable food consists of sweet potatoes taro and plantains and that bread fruit and yams are rather to be esteemed rarities. Of animal food they can be in no want as they have abundance of hogs which run without restraint about the houses and if they eat dogs which is not improbable their stock of these seem to be very considerable” (Beaglehole 1967). Similar observations of dense populations of dogs in the archipelago were made by Ellis (1836) and Mariner (1818), as summarized in Titcomb & Pukui (1969). [↑](#endnote-ref-39)
40. Though very little is known about the Polynesian dog, it was probably introduced with the cadre of commensal species that typically accompanied Polynesian voyagers, which also included rats, pigs and chickens (Luomala 1960a; Luomala 1960b). Dog remains are common in middens, and were preferred over pig and chicken as a food item (Titcomb & Pukui 1969). Ethnographic and historic anecdotal evidence confirms that dogs were common in Polynesian households, where they were fed on vegetable matter and may have functioned as food items and as sentinel species. [↑](#endnote-ref-40)
41. At least one observer in the post-contact period confirms the existence of feral dog populations in Hawaii in the 1820s: “I saw many skeletons of some kind of animal, devoid of all flesh, but apparently not long dead, and on rejoining our guide, was informed that the wild dogs had almost exterminated the sheep that Vancouver had brought with the cattle, pursuing them beyond the line of vegetation, where they became bewildered and died for want of food.” (Macrae 1922:56, in Titcomb & Pukui 1969:4). [↑](#endnote-ref-41)
42. In other coastal settings, it has been suggested that dogs played a major role in affecting prehistoric marine mammal populations. In work in the Channel Islands, California, Rick et al. (2008) have posited that dogs were likely to have had a significant effect on seals through the combined effects of harassment and hunting. Rick et al. (2008) have argued that dogs “negatively affected breeding bird and sea mammal populations on the mainland portions of the islands, likely driving these animals to offshore islets or other isolated areas.  If feral dog populations were present, as they were historically, these impacts would have been more pronounced.” [↑](#endnote-ref-42)
43. The monk seal may have survived extinction because of the last remaining rookery on Pearl and Hermes Atoll remained undiscovered until after petroleum products eclipsed marine mammal oil as the primary product for lubricants and lighting (Atkinson & Bryan 1914; Blackman 1941), and because decreases in whale populations in the Pacific led many of the major whaling business interests out of the Hawaiian Islands (Beechert 1991). [↑](#endnote-ref-43)
44. The lack of nesting hawksbill turtles in modern censuses of the NWHI may be a geographical signature of their vulnerability if nesting beaches have always been confined to the MHI, where they may have been impacted by introduced species and human harvesting pressures in pre-historic times (see Large Herbivores section). An alternative explanation is that hawksbill turtles have always been at low densities in the Hawaiian Archipelago. Currently there is no evidence supporting either of these hypotheses, but prehistoric reductions of green sea turtle and monk seal populations in the MHI provides some indication similar processes may have impacted hawksbill populations. [↑](#endnote-ref-44)
45. For sharks, Hawaiians developed several techniques for capturing sharks for food, ornamentation, and weaponry. Native Hawaiians developed a detailed ecological knowledge of these fish species in the inshore reef and deep-slope reef zones, and developed selective fishing technologies for harvesting these fish species (Beckley 1883; Cobb 1902; Cobb 1905a; Cobb 1905b; Titcomb 1972). Large bone and wooden hooks for catching sharks are found in some archaeological sites and sharks teeth were also used in implements, evidencing some prehistoric fishing pressure on shark species. [↑](#endnote-ref-45)
46. “The mano-kihikihi (hammer-headed shark) and the lalakea (white fin) are considered edible. The hammer-headed shark is the one most frequently seen in the markets. The others are the mano kanaka (man shark) the shark god of the ancient Hawaiians; the mano, a large white shark, and the niuhi [Tiger shark and Great White shark], the largest and fiercest of all. The last two are but rarely seen in Hawaiian waters.” (Cobb 1902:421); “In the olden times the catching of the niuhi was made a great event, but there has been no regular fishery for it for nearly one hundred years.” (Cobb 1902:422). [↑](#endnote-ref-46)
47. Though commoners and chiefs alike practiced fishing, there existed a professional class of specialized fishermen that were supported by the chiefly class, particularly for materials for the construction of canoes and fishing gear, which were derived from upland areas (Sinoto & Kelly 1975). Malo (1951), in one of the first written accounts of Native Hawaiian cultural practices, articulates a difference between ‘professional’ and ‘commoner’ fishermen. The po‘o lawai‘a, or expert fishermen, were supported by locally tenured resource managers (konohiki) and at higher levels by regional chiefs, to whom they provided tribute. Observations of this class of fishing experts are evidenced by ethnographic works (Malo 1951) and observed by early western explorers (Kelly 1969; Sinoto & Kelly 1975:97). [↑](#endnote-ref-47)
48. Early explorers, in their visits to the Hawaiian archipelago, noted that diseases had taken a toll on the indigenous population (Cook 1842; La Pérouse 1994; Vancouver 1801). Later on, summary information on epidemics indicates 1. McCoy MD (2008) Hawaiian limpet harvesting in historical perspective: A review of modem and archaeological data on Cellana spp. from the Kalaupapa Peninsula, Moloka'i Island. Pacific Science 62: 21-38. (Anonymous 1897)2. Culliney JL (2006) Islands in a far sea: The fate of nature in Hawaii. Honolulu, HI: University of Hawai'i Press.. [↑](#endnote-ref-48)
49. According to Clifford (1991), “The initial loss of ali‘i [chiefly] interest in most forms of fishing resulted in shortage, and the later abandonment of the kapu [Native Hawaiian religious and resource management system] structure, as it pertained to fishing was another step towards the abuse and further shrinkage of the indigenous fish populations. The shortage became so extreme that for a while, salmon had to be imported from the Pacific Northwest to provide food for the commoners” (Clifford 1991). Information on fish imports (Buckland 1908; Cobb 1902; Cobb 1905a; Cobb 1905b; Thrum 1907) supports the notion that fish shortages existed in the 1800s and continuing through the early part of the 20^th^ century. Several hundred thousand pounds of seafood products were imported into Hawaii in the early 1900s and J. N. Cobb, an early surveyor of Hawaiian fisheries, indicates this had been ongoing at least during the late 1800s, the earliest information in his reports (Cobb 1902; Cobb 1905a; Cobb 1905b). By Cobb’s account, much of this was to satisfy a generally high preference for fish among Hawai‘i residents, but some imports were also aimed at accommodating specific cultural preferences in the multi-ethnic population of Hawai‘i. [↑](#endnote-ref-49)
50. An increased focus on the part of Hawaiian chiefs on Western goods, resulted in chiefly re-direction of the Hawaiian population away from traditional fishing and farming and toward production and harvesting of sandalwood, pork, and other commodities valued by westerners (Kuykendall 1938; Miller 1989). According to Miller (1989), Hawaiian chiefs quickly developed an interest in armaments that conveyed a military advantage over enemies. This became the overriding concern of some chiefs, and resources were diverted away from traditional trades and re-invested in others. For example, animal husbandry became an initial focus after Western contact, since pigs became a major trade item with the Westerners visiting the islands in the first decades after contact; later on, the focus would shift to sandalwood resources. Support for decreases in harvesting pressures are also found in the archaeological record. For example, McCoy (2008) found increases in limpet sizes in the archaeological record during the early historic period, which he attributes to decreases in harvesting pressures from depopulation of the area under study. [↑](#endnote-ref-50)
51. The attrition of young men resulted in less fishing effort, as these individuals comprised the primary fishers in professional fishing classes; attrition became such an issue that the Hawaiian monarchy required whaling companies to post bonds with the Hawaiian government for each man that was hired on a whaling vessel. An account from John C. Jones describes the engagement of Hawaiians in the whaling trade, both as sailors on American-owned ships, and as purchasers of U.S. ships: “A practice has for many years existed with the commanders of ships touching the Sandwich Islands either for supplies or trade, to turn on shore all seamen against whom they could allege any trivial misconduct, and employ in the lieu natives of the Islands, by this means lessening their passage bills, but depriving their country of valuable subjects. American vessels have been sold to the natives and their crews discharged, without any means of support, thus left to the protection and mercy of the rude savage…These abuses of the laws of the United States I have been able to remedy.” [emphasis added] (Jones 1948) [↑](#endnote-ref-51)
52. “The far roaming fisherman told us that the fine edible fish are scarcer around HI than in any island group toward the south. Only sharks and swordfish and ahis [tunas] teem around these isles. The Hawaiian waters have been overfished, he believes. …sharks are sought for their meat, turned into commercial fishcakes, known as kamabuko. ...sharkfin is considered a delicacy among Chinese and when properly dried and seasoned brings as high at $2 per pound at the market. Another valued by-product of shark is its oil, considered superior to olive oil by Oriental epicures and now sells at 80c per quart.” (Inouye 1933) [↑](#endnote-ref-52)
53. “the Hawaii of the good old days was a land of kindly people… of a sea swarming with fish, taken by exploding a stick of dynamite in the water, an expedient that half-filled a boat with fish ranging from sixty-lb ulua [jacks, trevally] to the diminutive ala-lau-wa [aweoweo, Hawaiian big-eye], a little fellow of reddish color, delicious when fried. Only once or twice did I see them in large schools; at such times, no native belief ran, they presaged the death of some high-born Hawaiian” (Farrell 1928) [↑](#endnote-ref-53)
54. Even if waning chiefly sponsorship reduced the fishing effort and capacity post-contact in Native Hawaiian communities, many of the deep reef and other offshore fishing methods developed by Native Hawaiians persisted to the modern period (>AD 1900) (Cobb 1902; Maly & Maly 2004; Waterhouse 1898). [↑](#endnote-ref-54)
55. This reversal was due in part to declining abundances of nearshore reef species, but also to increased effort by the Japanese in the pelagic and deep-reef realm, and an exodus of Native Hawaiians from the industry, forced out by increased numbers of Japanese who formed competitive business associations. A rapid transformation in the fishery occurred in the first decade of the 1900s, when Japanese sampans replaced the largely inshore fishery dominated by Native Hawaiian canoes. The first diesel engine was installed on a sampan in 1905 and shortly thereafter the geographic range of the fleet expanded dramatically. Fisheries also began to become concentrated around the major urban areas in the islands, and Honolulu quickly became the primary venue for commercial fishery catch to be marketed and sold. [↑](#endnote-ref-55)
56. “There is a widespread feeling in Hawaii that fish have declined in abundance during the last quarter century…It is generally believed that depletion from over-fishing has occurred primarly [sic] among the inshore or shallow-water species, no anxiety being expressed over the condition of the offshore fisheries for pelagic or deep-water species…Although the total landings have increased several fold since the beginning of the century, individual opinion is virtually unanimous that certain species of fishes, particularly those most highly prized for food, have been seriously reduced in abundance.” (Bell and Higgens 1939) [↑](#endnote-ref-56)
57. For example, as early as 1900, Cobb (1902) noted that some species of parrotfish had become quite rare due to over-harvesting, and were offered in the commercial markets only at exorbitant prices. Increased production by the Japanese of bottomfish (snappers and groupers) offset declines in preferred reef species such as jacks, trevallys, parrotfish and other species. [↑](#endnote-ref-57)
58. “[describing earlier times] Skillful and daring fishermen, with a thorough knowledge of the habitat and habits of fishes; the seasons of their periodical migrations, spawning, etc.; and they had stringent laws and regulations for the taking of fish, looking towards their preservation. Fish were abundant in the waters surrounding the Hawaiian Islands in those days. Alas! The white man, with his alleged superior knowledge, prevailed on chief and commoner to throw down their wholesome restrictions, as savoring of superstition, with the result that fishes are very scarce in Hawaiian waters, and getting more and more so every year. The tabu of the Hau, which meant the close season for shore fishing, to allow the ama-ama (mullet) and kindred fishes to breed and spawn undisturbed for six months, is no longer kept.” (Nakuina 1904). [↑](#endnote-ref-58)
59. Oral History Interview with Peter Kaikua’ana Park (PP) by Kepā Maly (KM)

    PP: “In the old days they had kapu [restricted or closed] times, and they really observed it. You know why, that was their food. Take only what you need, leave the rest, if you don’t, then you not going have. Even when they hukilau [use a large net for inshore reef fishing] like that, as I understand, they hukilau, the village to come down help and huki [pull] the lau [line], and everything. When they get the fish all corral in one place, they tell everybody you go and help yourselves as much as you want, as much as you can eat and save. They go and take as much as they can use. And if there’s balance, then they open the net and let it go for another day. They no uhauha (act greedy). They take what they need because no ice box those days too. You take and you no can keep in the ice box, then poho!”

    (Maly & Maly 2004:30)

    Oral History Interview with Walter Keli’iokekai Paulo (WP) by Kepā Maly (KM)

    WP: “You malama [take care of] the ko’a [offshore reef fishing area], the ko’a is going to malama you. You malama ka ‘aina [take care of the land], the ‘aina is going to malama you [the land will take care of you].”

    WP: “Those days, there were kapu [restricted times or closed areas], like six months during the season when aku [skipjack tuna] was kapu [tabooed, or off limits], you were fishing ‘opelu [mackerel scad]. And then the aku was open, ‘opelu was closed. And the penalty (for breaking the kapu) was death. And the executioners…well, there was no lawyer to defend you. That’s it! They used the club to bash you on the head…”

    (Maly & Maly 2004:31) [↑](#endnote-ref-59)
60. Japanese fishing sampans returning to port after the attack, the crews of which were unaware of the Pearl Harbor attack, were fired upon by the US navy. As a result, six fishermen lost their lives, after which the US Navy required sampans to paint American flags on their hulls in order to be easily identified. [↑](#endnote-ref-60)
61. Oral History Interview of July 21st, 1998 of Kahu John “Kumukāhi” Makuakāne (JM) at ‘Opihikao by Kepā Maly (KM):

    JM [describing his childhood, ~1940s-1950s]: Once, we caught one that was so big. My sister had come down and visit us, and her friend had a car. We didn’t have a car yet, those days. And we had to go home, tell the guy to come down. We tied the rope to the car to pull the fish out.

    KM: Wow! How big would you say that ‘ulua was?

    JM: My golly, if it wasn’t close to 200 pounds. There was no way you could pull it up. And we pulled 80, over 100 pounds. The average weight that we used to catch was 60 to 80 pounds. That was the typical kind of size. (Maly & Maly 2003d: 127) [↑](#endnote-ref-61)
62. “There are occasional notices of sealers in the maritime notes of the newspapers of the islands after this date, as in 1859, when the bark Gambia, 249 tons, is reported as having been sealing. She left Honolulu on April 26, and cruised among the islands to the westward of this group, returning on August 7 with 240 barrels of seal oil, 1,500 skins, a quantity of shark fins and oil, etc.” [↑](#endnote-ref-62)
63. [describing an unnamed ship and voyage in 1893]: “an expedition killing sixty or seventy in Laysan” (Bailey 1952) [↑](#endnote-ref-63)
64. 1. [describing Kure Atoll, 1825]: “The shores were lined with sea elephants” (Morrell 1832);
    2. [describing Kure Atoll 1870]: "The seal… are growing less in numbers…I commenced by sending out parties to kill seals… but after about a month I found that, owing to the rapid diminution of the seal, I was obliged to cut the allowance down, and only killed one seal... per day for the whole crew…. Also, we have not lately attempted fishing on the reef, for fear of reducing their food.” (Read 1912);
    3. [describing Laysan Island, May 1857]: “Seal … were numerous on the beach and might be easily taken.” (Paty 1857);
    4. [describing Laysan Island, 1886]: Shark fishing boat Wanderer killed seals for bait (Farrell 1928); [describing Laysan Island, 1891]: No seals seen by Kaalokai (Rothschild 1893)

    [↑](#endnote-ref-64)
65. “During the latter half of the last century particularly, considerable shark fishing was done among the chain of islands to the westward of the main group, and these islands in time came to achieve an unenviable notoriety from the number of wrecks which occurred upon their shores. The first record we have of this fishery was in 1859 when the bark Gambia returned from a three and one-half months’ cruise amongst these islands with, among other things, a quantity of sharks’ fins and oil. In 1872 the Henrietta made a cruise among the islands for the same purpose. In 1886 the schooner General Seigel, while on a shark-fishing cruise, parted her cables and went ashore at Midway…. Very little shark fishing has been done of late years owing to the lack of a profitable market for the products obtained.” (Cobb 1902) [↑](#endnote-ref-65)
66. Archaeological investigations into shellfish assemblages have shown decreases in size and switches in fauna from higher to lower value prey, evidencing some resource depression (Hammatt et al. 1978; Morrison & Hunt 2007). At some sites in Hawai‘i, notably Halawa Valley on the island of Moloka‘i, the weight of midden fishbone decreases while pig and dog increase, coincident with the development of agricultural systems (Kirch & Kelly 1975; Kirch & McCoy 2007). At other sites the proportion and weight of bones of fish, mammal and other remains more or less stable through time, with weak trends suggesting shifts from small carnivorous fish to small herbivorous fish which may have been spurred by the development of fishing technologies (Goto 1986; Kirch 1979b). [↑](#endnote-ref-66)
67. McCoy (2008) found that shellfish in archaeological deposits increased in average size over time: “increases in average limpet size and decreases in population over the period between 1650 to the present are closely matched … In the Proto-Historic Period [AD 1650-1795], population and harvest pressure are assumed to have been at their height, and shellfish were roughly one-quarter to one-third smaller than their current sizes. By the Early Historic Period [AD 1795-1866], human population was conservatively 17% lower than in the Proto-Historic Period, and average Cellana lengths jumped 18–19% over the previous period… when one accounts for the rates of demographic decline and shellfish recovery it appears that ‘bounce back’ [recovery] was swiftest during the most extreme phase of the demographic crash.” (McCoy 2008:34-35) [↑](#endnote-ref-67)
68. “A few of the more public spirited owners of fishery rights made every possible effort to conserve and increase the supply of fish and through the medium of the provision in the law allowing such owners in lieu of setting apart some peculiar fish to their exclusive use to prohibit during certain indicated months of the year all fishing of every description upon their fisheries they placed tabooes [restrictions] on certain fish notably the ama ama [mullet] during their spawning seasons and thus gave a measure of protection which is entirely lacking at present. The only species now protected are the young of the ama ama [mullet] and the awa [milkfish] it being unlawful to take these fishes under 4 inches in length. So far as the ama ama [mullet] is concerned this law is disregarded in all but a few places. Thousands of young mullet from 1 to 2 inches in length and known as pua are taken by the fishermen of Molokai and Maui in fine meshed nets and sold. Large quantities are taken in the fisheries of other islands, also, particularly Oahu and sold to the workmen on the sugar plantations…” (Cobb 1905b:437-438, emphasis added) [↑](#endnote-ref-68)
69. “...noticed in the harbor of Honolulu large schools of nehu [anchovy] and iau [Hawaiian silverside] which were used as live baits for aku [skipjack tuna] fishing. So thick were the schools that the water appeared red with them” (Konishi 1930:4) [↑](#endnote-ref-69)
70. As described by Bryan, the arriving schools of bigeye were greeted with both superstition and celebration for harvest: “The Catalufas (Hawaiian Bigeye)..[or] aweoweo is the famous red fish which during the month of September 1873 entered Honolulu harbor in shoals. They were evidently young fish as the largest were not more than three and a half inches long. This shoaling has occurred from time to time at irregular intervals. In the mind of the native the coming of the red fish presages the sickness and death of some member of the royal family. On several occasions there has been a singular sequence of events of this nature which has left its impress on the beliefs of the more superstitious among the people. The fish are esteemed as food by the natives however who regard their coming in large numbers in the nature of a windfall as the fish can be readily dried and saved. The species is of wide distribution and among English speaking people is known as the big eye” (Bryan 1915:354) [↑](#endnote-ref-70)
71. Cobb (1903) commented, “the fine meshed nets are used almost entirely by the Japanese who throw away probably one fourth of the catch in some localities notably in Pearl Harbor in order to keep up the present high prices of fish.”

    Drs. Jordan and Evermann led the U.S. Fisheries Commission investigation in the early 1900s and published many of the first taxonomic surveys using reef fish obtained from commercial markets. Their experience in commercial fisheries and fish biology heightens the value of their observations of declining nearshore fisheries in the 1920s: “The fauna of the reefs is much less abundant than in the period of the first extensive explorations, those of Dr. Oliver P. Jenkins in 1889 and Jordan and Evermann in 1901. Probably no species has been actually exterminated by overfishing, but many once common have now become rare.” (Jordan et al. 1927) [↑](#endnote-ref-71)
72. Oral History Interview of February 14th and June 21st, 2003 of Valentine K. Ako (VA) at Wailua, Kaua‘i by Kepā Maly (KM):

    VA: [describing changes since his early fishing days 1932-1950 on the Island of Hawai‘i] Technology, has improved the ability to take, but led to depletion of fisheries:

    VA: The bad part about these depth recorders. Whereas we, during our days we know a certain ko‘a [offshore reef fishing area], you don’t go fish. If you fish today you no go fish over there for two or three months. You leave them alone to replenish, then you go back.

    KM: Yes.

    VA: Whereas these guys they go week after week.

    KM: And not only them, plenty guys, right?

    VA: Yes. Four or five boats at one time. Naturally, you’re going to fish out that ko‘a, and they

    blame each other.

    KM: Yes. Do you think that the improved and I use quotes, “improved” technology. You said depth recorder, the GPS [global positioning system], the FADS [fish aggregation devices], the amount that you can take. Do you think that these things have also impacted the well-being of the fish?

    VA: Yes, yes! All this modern technology has screwed up everything. It’s improved the ability to take.

    (Maly & Maly 2003d:435) [↑](#endnote-ref-72)
73. “The number of large vessels fishing in the NWHI declined during the 1950s because of vessel losses and low fish prices, and only a single vessel remainted in operation in the 1960s.” (Hau 1984:266) [↑](#endnote-ref-73)
